# First Generation Proteolysis Targeting Chimeras (PROTACs) for the Treatment of Progeria

**DOI:** 10.1002/advs.202521608

**Published:** 2026-03-23

**Authors:** Jon Macicior‐Michelena, Marcelino Telechea, Daniel Fernández, Alba García‐Martín, Ángeles Canales, Silvia Ortega‐Gutiérrez

**Affiliations:** ^1^ Department of Organic Chemistry Universidad Complutense de Madrid Madrid Spain

**Keywords:** lamin A, progeria, progerin, proteolysis targeting chimeras (PROTACs), senescence

## Abstract

Hutchinson‐Gilford progeria syndrome (HGPS) is a rare genetic disorder caused by a mutation in the LMNA gene, leading to the production of progerin, an aberrant and toxic form of lamin A. Due to its hydrophobic nature, progerin accumulates at the nuclear membrane, disrupting nuclear architecture, impairing cellular functions, and ultimately resulting in death around adolescence. Reducing progerin levels is considered the most effective strategy to improve disease progression. To date, small‐molecule approaches have primarily targeted progerin upstream mechanisms to indirectly reduce its levels. In this study, we report the development of first‐generation proteolysis targeting chimeras (PROTACs) designed to directly degrade progerin, establishing a novel therapeutic paradigm for HGPS. We identify UCM‐18142 (compound **2**) as the first PROTAC capable of selectively degrading progerin. Treatment with UCM‐18142 results in significant improvements in cellular phenotype in both HGPS patient‐derived cells and a murine model, including enhanced proliferation, reduced senescence markers, and normalization of nuclear and mitochondrial abnormalities. Additionally, transcriptomic analysis of treated human cells reveals the cellular pathways modulated by compound. Remarkably, PROTAC **2** reduces progerin levels in vivo, supporting the therapeutic potential of this direct‐targeting approach and opening new avenues for intervention in HGPS and related laminopathies.

## Graphical abstract

We report the first PROTACs designed to degrade progerin, introducing a novel therapeutic approach for progeria. The best compound, UCM‐18142, significantly reduces progerin levels and improves key disease phenotypes in patient‐derived cells and in the *Lmna^G609G/G609G^* mouse model, paving the way for new treatment strategies targeting the root cause of the disease.

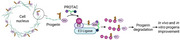

## 1 Introduction

**FIGURE 1:**
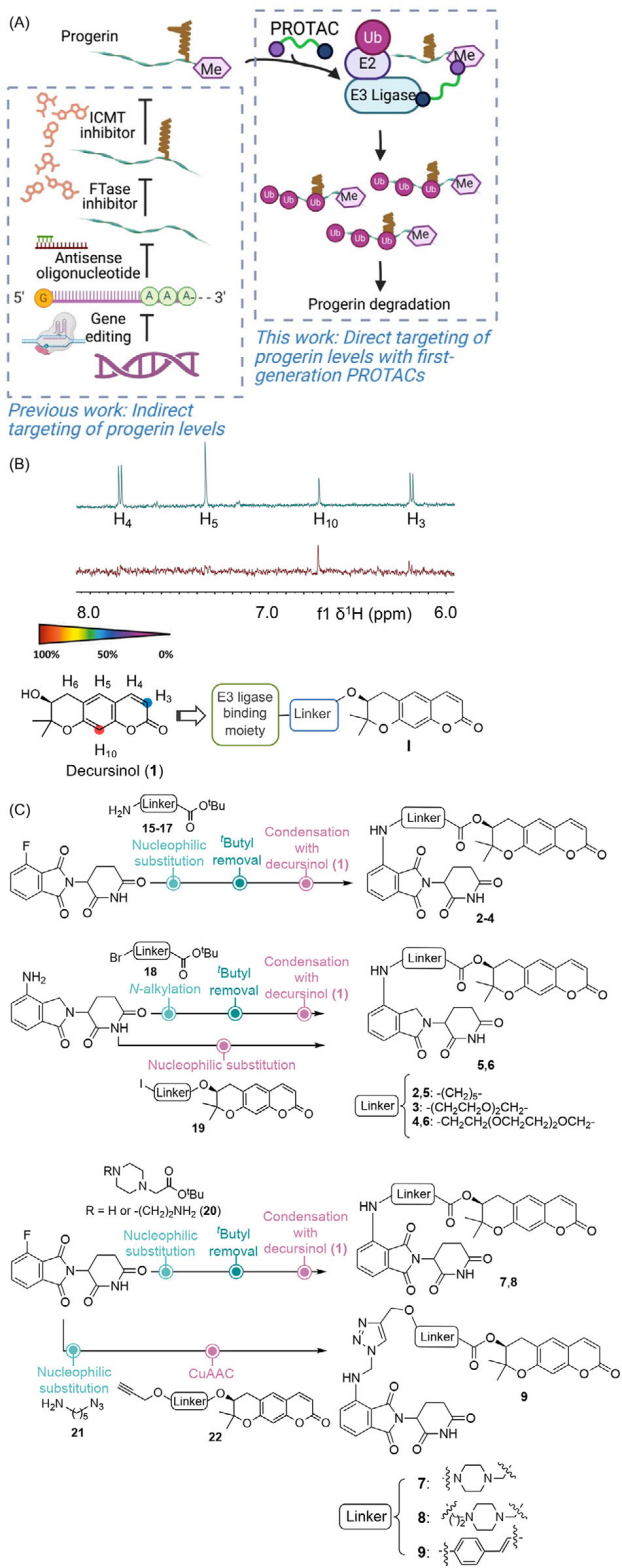
(A) Schematic comparison of previous and current strategies to reduce progerin levels. (B) Off‐resonance (top, green) and STD (bottom, red) spectra of the aromatic region of decursinol. Highest STD intensities correlate with the closest ligand–protein interaction points in the bound state. Protons with an STD value greater than 50% are coloured and represent different degrees of contact as shown in the scale. (C) Synthesis of designed PROTACs based on decursinol and cereblon E3 ligase ligands pomalidomide (**2‐4** and **7‐9**) and lenalidomide (**5,6**). Synthetic details are provided in the Supporting Information.

Hutchinson‐Gilford Progeria Syndrome (HGPS) or progeria, is a rare genetic disease that affects children. It is characterized by an accelerated aging phenotype that leads to a premature death at around the age of 14.5 years [1, 2, 3, 4]. This average life expectancy can be extended up to 33% with the administration of lonafarnib, the only drug currently approved for the treatment of progeria [5]. This moderate efficacy, together with the fact that lonafarnib does not improve some of the key phenotypic hallmarks of progeria [6], has stimulated the interest in the search of new therapeutic strategies [7, 8]. In addition, and taking into account that progeria resembles to physiological aging, especially in relation with cardiovascular disease [9, 10], correction of these phenotypes could also be applicable in normal aging, a factor that enormously broadens the relevance of the treatments for progeria. The molecular cause of the disease is a point mutation (1824C>T, G608G) in the lamin A encoding gene [11, 12], which produces a splice variant transcript that cannot undergo the usual post‐translational modifications required to convert prelamin A into mature normal lamin A after translation [13]. Prelamin A belongs to the CAAX family of proteins, where this C‐terminal tetrapeptide is a signal for a four‐step post‐translational processing involving (i) proteolysis of the three terminal residues; (ii) farnesylation and (iii) carboxymethylation of the terminal cysteine and (iv) removal of the 15 terminal amino acids. When the G608G mutation occurs, the translated protein lacks the signal for this final step, resulting in a methylated and farnesylated lamin A variant called progerin. Progerin accumulates in the nucleus and affects virtually all cellular functions. Recent research suggests that reducing progerin accumulation is the single most important factor to significantly improve the phenotype of progeria [14]. Reducing progerin accumulation has been tried in indirect ways, either by inhibiting the enzymes responsible for farnesylation (the mechanism of action of lonafarnib) or methylation; [15] by blocking its interaction with lamin A; [16] by using antisense oligonucleotides (ASOs) [17, 18, 19] or by genetic approaches [20, 21]. However, a direct reduction strategy based on small molecules has not been explored (Figure 1A), even though such an approach would overcome the significant limitations of ASOs and gene therapies to become approved drugs, while taking advantage of the drug‐like nature of small molecules and the specificity that results from directly targeting progerin.

In this context, targeted protein degradation stands out as a promising strategy to promote the specific removal of progerin using the existing cellular machinery. Proteolysis targeting chimeras (PROTACs) are heterobifunctional small molecules consisting of two moieties usually separated by a linker. One moiety binds to the protein to be degraded, or protein of interest (POI), and the other one recruits an E3 ligase which, when brought into close proximity to the target protein, catalyzes its ubiquitination, thus tagging it for destruction by the proteasome [22, 23, 24, 25]. This field has experienced an exponential growth over the last two decades, culminating in the advancement of more than 20 PROTACs into clinical trials, including vepdegestrant (ARV‐471), which has just received FDA approval under the fast track designation for the treatment of locally advanced or metastatic breast cancer; [26, 27] ARV‐102, the first PROTAC with activity in the central nervous system to enter Phase 1 clinical trials; and KT‐474, the first heterobifunctional degrader evaluated in a non‐oncology indication and currently in Phase 2 clinical trials [28]. These milestones confirm the potential of PROTACs to become new drugs. In this context, progerin is an excellent candidate to be targeted by PROTACs, since it is a structural protein lacking defined binding pockets or active catalytic sites. In this work, we present the development of first‐generation PROTACs designed to selectively degrade progerin, introducing a novel therapeutic strategy for progeria. Among the synthesized compounds, UCM‐18142 (**2**) emerged as the most potent candidate, effectively reducing progerin levels and improving hallmark cellular phenotypes associated with the disease, including enhanced cell proliferation, reduced senescence markers, and normalization of nuclear and mitochondrial alterations. Notably, UCM‐18142 demonstrates efficacy in HGPS patient‐derived cells and in the *Lmna^G609G/G609G^* mouse model, underscoring its translational potential and opening new avenues for the treatment of progeria through direct progerin targeting.

## 2 Results and Discussion

### 2.1 Design and Synthesis of Progerin‐Directed PROTACs

PROTACs are composed of three structural subunits: (i) a ligand with affinity for the POI (progerin in this case); (ii) an E3 ligase binding subunit; and (iii) a linker that separates both moieties. While several E3 ligase binding motifs have been described [29, 30, 31] and a wide variety of spacers have been reported [32, 33], there is a lack of available progerin ligands. As the ones described so far contain the scaffold of the natural product decursinol [16, 34, 35], we selected this fragment as the progerin‐binding moiety (**1**, Figure 1B). To identify the optimal exit‐vector we carried out saturation transfer difference (STD) NMR studies. Incubation of decursinol in the presence of progerin allowed us to calculate the percentage of STD, by comparing the signal intensity of each proton in the STD spectrum to the signal of the same proton in the off‐resonance spectrum. Two of the protons of decursinol (H_3_ and H_10_) were identified as the closest ones to the protein, with the highest STD signals (Figure 1B). Considering the lack of other significant interactions, we selected the free alcohol in position 7 as the exit‐vector for attaching the linker and the E3 ligase recruiting moiety, thus leading to the design of PROTACs of general structure **I**. Initially, we chose pomalidomide and lenalidomide, two widely used ligands of the E3 ligase cereblon (CRBN), and explored a variety of flexible linkers of different lengths (**2‐6**, Figure 1C).

The ability of compounds **2**‐**6** to degrade progerin was evaluated in *Lmna^G609G/G609G^* fibroblasts, derived from the progeria mouse model that most closely replicates the human disease phenotype [19]. Lamin A/C antibody was used for immunodetection due to its prevalent use in this context, enabling simultaneous visualization of both lamins and progerin [15].

**FIGURE 2:**
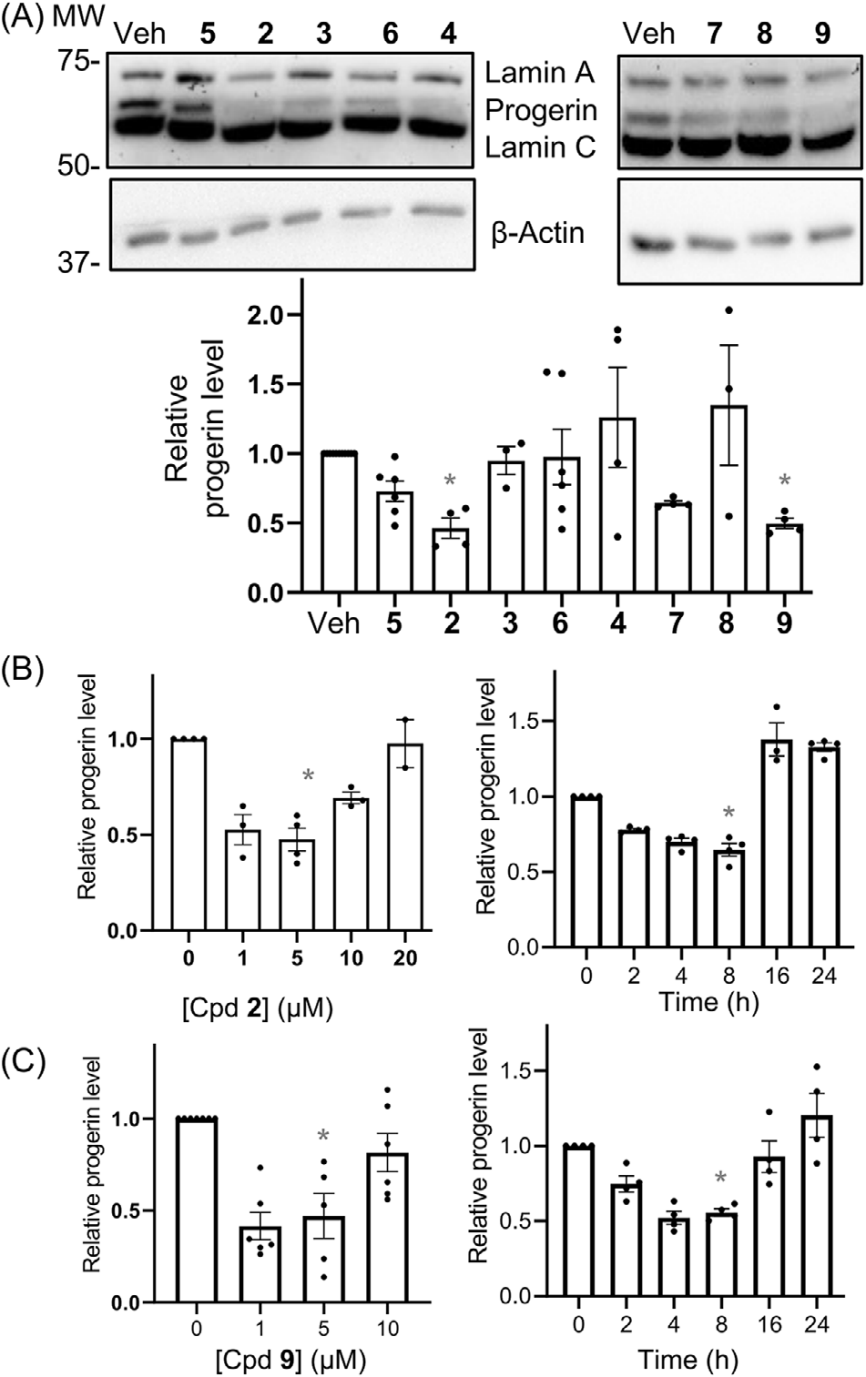
(A) Representative cropped immunoblots and quantification of progerin from *Lmna^G609G/G609G^* fibroblasts. Cells were incubated with vehicle or indicated compound at 5 µm for 8 h. MW, molecular weight (in kDa) markers. (B, C) Concentration and time‐dependency of the reduction of progerin levels induced by compounds **2** and **9**. Graphs show quantification of progerin in *Lmna^G609G/G609G^* fibroblasts (immunoblots are shown in Supporting Figure S1). Cells were incubated with vehicle or the indicated compound concentration for 8 h. For time‐course experiments, cells were incubated with vehicle or indicated compound at 5 µM. Bar graphs show mean±sem of 3 independent experiments performed in duplicate. Data were analysed using one‐way ANOVA followed by Dunnett's post–hoc test comparing all groups to the vehicle; ^*^, *p* < 0.05.

The results demonstrated superior activity of pomalidomide‐containing analogues over lenalidomide derivatives (Figure 2A). Hence, pomalidomide was retained in compounds **7–9**, in which different linkers were explored (Figure 1C). Progerin degradation assays identified compounds **2** and **9** as the most effective in reducing progerin levels (Figure 2A), so they were selected for time‐ and concentration‐dependency studies (Figure 2B,C).

Our findings indicate that maximum degradation occurs after 8 h and at a compound concentration of 5 µm. At higher concentrations a noticeable hook effect can be observed, a phenomenon commonly observed in PROTACs [36]. The fact that progerin removal is not complete likely reflects the intrinsic properties of this protein. Progerin displays an unusual long lifetime and reduced turnover, as it forms poorly extractable and densely assembled nuclear lamina polymers [37]. These features, together with the decreased proteasome activity observed in fibroblasts from progeria patients [38], render a substantial fraction of the cellular progerin pool less accessible to complete proteasomal degradation, which is consistent with the moderate D_max_ observed.

**FIGURE 3:**
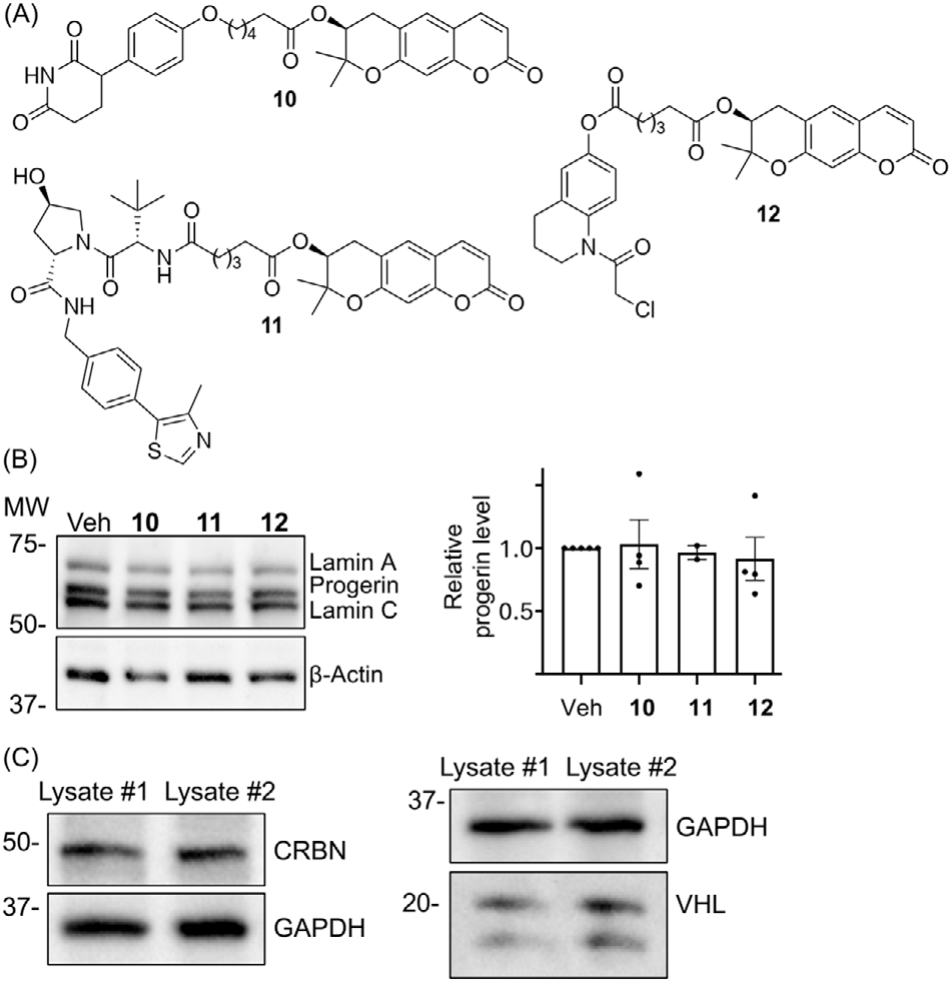
(A) Structure of compounds **10‐12**. Synthetic details are provided in the Supporting Information. (B) Representative cropped immunoblots and quantification of progerin in the presence of compounds **10‐12**. *Lmna^G609G/G609G^* fibroblasts were incubated with vehicle or indicated compound at 5 µm for 8 h (bar graph shows mean±sem of 3 independent experiments performed in duplicate). One‐way ANOVA was performed, and no statistically significant differences were found. MW, molecular weight (in kDa) markers. (C) CRBN and VHL expression in *Lmna^G609G/G609G^* fibroblasts from 2 independent experiments (lysates #1 and #2).

To investigate the impact of different E3 ligase binding subunits beyond pomalidomide and lenalidomide, we considered phenyl glutarimide, a recently described structural motif that retains a high affinity for CRBN [39]. We also explored how recruiting different E3 ligases could impact the activity of the PROTACs. For this, we selected VHL and DCAF16, the former being one of the most frequently recruited E3 ligases in successful PROTACs and the latter being a recently described E3 ligase that is highly conserved in mammals but absent in rodents [40]. This compound would provide an additional negative control in mouse *Lmna^G609G/G609G^* fibroblasts, while representing a new PROTAC for assays conducted in human patient cells. Thus, compounds **10‐12** (Figure 3A) were synthesized and their ability to reduce progerin levels determined. As anticipated, DCAF16 recruiting PROTAC **12** failed to reduce progerin levels. Similarly, neither the glutarimide derivative **10** nor the VHL‐ligand‐containing compound **11** led to a significant reduction in progerin levels (Figure 3B). These findings underscore the critical role of pomalidomide in progerin‐targeting PROTACs, as substituting it with alternative CRBN ligands significantly diminishes activity. Additionally, we confirmed the expression of both E3 ligases, CRBN and VHL, in *Lmna^G609G/G609G^* fibroblasts (Figure 3C).

**FIGURE 4:**
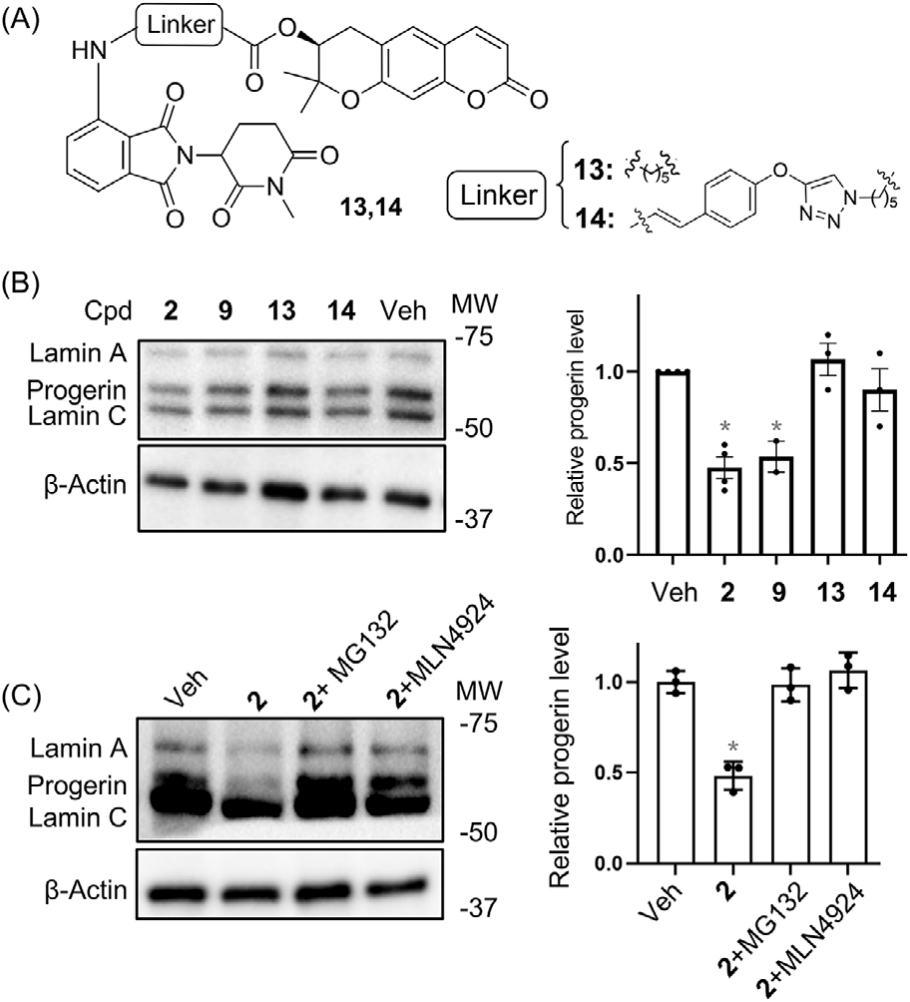
(A) Structure of compounds **13** and **14**. Synthetic details are provided in the Supporting Information. (B) Representative cropped immunoblots and quantification of progerin in the presence of compounds **2**, **9**, **13**, and **14**; (C) Proteosome and cullin E3‐ligase dependency of compound **2**. *Lmna^G609G/G609G^* fibroblasts were incubated with vehicle or indicated compounds at 5 µm for 8 h. Bar graphs show mean±sem of 2 independent experiments performed in duplicate. Data were analysed using one‐way ANOVA followed by Dunnett's post–hoc test comparing all groups to the vehicle; ^*^, *p* < 0.05. MW, molecular weight (in kDa) markers.

To confirm that the effects of compounds **2** and **9** were consistent with a PROTAC mechanism, we synthesized analogues **13** and **14** (Figure 4A; see Supporting Information for the synthetic details) bearing a methylated glutarimide, a modification known to abolish CRBN binding. As anticipated, these analogues were ineffective in lowering progerin levels (Figure 4B). Additionally, the activity of compound **2** was abolished in the presence of the proteasome inhibitor MG132 and the neddylation inhibitor MLN4924 (Figure 4C) supporting the proteosome and cullin E3 ligase dependency. We also confirmed the formation of the ternary complex between progerin, compound **2** and CRBN by immunoprecipitation as well as the direct binding between compound **2** and progerin by cellular thermal shift assays (Figure S2). Obtained curves allowed us to estimate the binding affinity by isothermal dose‐response fingerprint (IC_50_ = 7±1 µm). This value is consistent with the observation that 1 and 5 µm are the most efficacious concentrations, as the catalytic mechanism of PROTACs enables activity below the IC_50_ of direct binding affinity. Global proteomic analysis did not identify significant alterations in overall protein abundance (Figure S2), which is consistent with compound **2** exhibiting a degree of selectivity. It should be noted, however, that progerin cannot be detected within this workflow, as its detection requires a targeted mass‐spectrometry strategy [41]. This methodological constrain limits the interpretability of the global proteomic data with respect to progerin degradation. We also confirmed that compounds **2** and **9** did not induce significant cytotoxicity in *Lmna^G609G/G609G^* or *Lmna^+/+^* fibroblasts up to 50 µm (Figure S3).

Pharmacokinetic properties, including solubility and membrane permeability, are critical in drug discovery but can pose challenges in PROTAC development [42, 43] In our study, PROTAC **2** yielded a solubility value of 0.5 µm in phosphate buffer solution (PBS, pH = 7.5), which is comparable to the solubility values of other recently described potent degraders [44]. The calculated logarithm of the partition coefficient between *n*‐octanol and water (clogP), determined using ACDLabs Percepta Software, indicated an acceptable lipophilicity for PROTAC **2** (clogP = 3.94), aligning with Lipinski's “rule of 5” (clogP<5). In contrast, compound **9** displayed fivefold lower solubility in PBS (0.1 µm) and a significantly higher clogP value of 5.49, reflecting its increased lipophilicity. Compound permeability was assessed using the parallel artificial membrane permeability assay (PAMPA), which measures the ability of compounds to diffuse through a lipid‐infused artificial membrane. Compound **2** exhibited good permeability (P = 6.7 × 10^−6^ cm/s). In contrast, compound **9** was below the detection limit under the same conditions, indicating its lower capacity to passively cross biological membranes. The cellular uptake of compound **2** was further confirmed by fluorescence microscopy, leveraging the intrinsic fluorescence of pomalidomide‐bearing derivatives (Figure S4). Additionally, compound **2** exhibited favorable stability and human serum albumin (HSA) binding (Table 1), making it a suitable candidate for in‐depth cellular characterization.

**TABLE 1:** Pharmacokinetic parameters of PROTAC **2**.

P (cm/s)^a^	Stability (hours)^b^			HSA binding^c^
	h‐serum	m‐serum	Cell culture	
6.7·10^−6^	>24	0.28±0.02	>24	99% *K*_D_ = 5.8·10^−6^ m

^a^ P, permeability;

^b^ h, human; m, mouse;

^c^ HSA, human serum albumin.

### 2.2 Cellular Characterization of PROTAC 2 in Mouse Progeroid Cells

**FIGURE 5:**
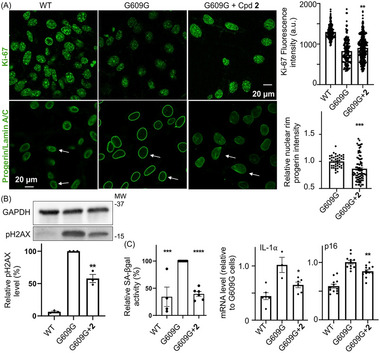
(A) PROTAC **2** increases cell proliferation (upper panels) and decreases nuclear cell membrane thickness and progerin accumulation (lower panels). Confocal fluorescence microscopy images are representative of 2 independent experiments. Bar graph shows mean±sem of at least 3 fields from 2 independent experiments. (B) PROTAC **2** reduces the levels of phosphorylated histone 2AX (pH2AX). Representative cropped immunoblots are shown. Bar graph shows mean±sem levels of pH2AX relative to GAPDH from 3 independent experiments. MW, molecular weight (in kDa) markers. (C) PROTAC **2** decreases senescence‐associated β‐galactosidase (SA‐βgal) activity, interleukin (IL) 1α and p16 senescent markers. *Lmna^G609G/G609G^* (G609G) or *Lmna^+/+^* (WT) fibroblasts were incubated with vehicle or compound **2** (5 µm). Bar graph shows mean±sem of 3 independent experiments performed in duplicate (SA‐βgal and IL‐1α) or 4 independent experiments performed in triplicate (p16). Data were analysed using one‐way ANOVA followed by Dunnett's post–hoc test. ^*^, *p* < 0.05; ^**^, *p* < 0.01, ^***^, *p* < 0.001, ^****^, *p* < 0.0001 vs G609G cells.

We sought to determine whether the reduction in progerin levels induced by PROTAC **2** could significantly improve the progeria phenotype. Progeroid cells exhibit a globally senescent phenotype, characterized by impaired proliferation, mitochondrial disfunction, increased DNA damage, nuclear defects, and elevated expression of senescence markers [15, 45, 46]. Our results show that PROTAC **2** significantly increases cell proliferation, as assessed by the proliferation marker Ki‐67, and decreases progerin accumulation in the nuclear membrane (Figure 5A).

Remarkably, the compound also reduces the levels of phosphorylated histone 2AX (pH2AX), a marker of nuclear damage (Figure 5B). Well‐known senescent markers such as the senescence‐associated β‐galactosidase (SA‐βgal) activity, interleukin (IL) 1α and p16 were also significantly reduced after treatment of progeroid cells with compound **2** (Figure 5C).

### 2.3 Cellular Characterization of PROTAC 2 in Human Progeroid Cells

**FIGURE 6:**
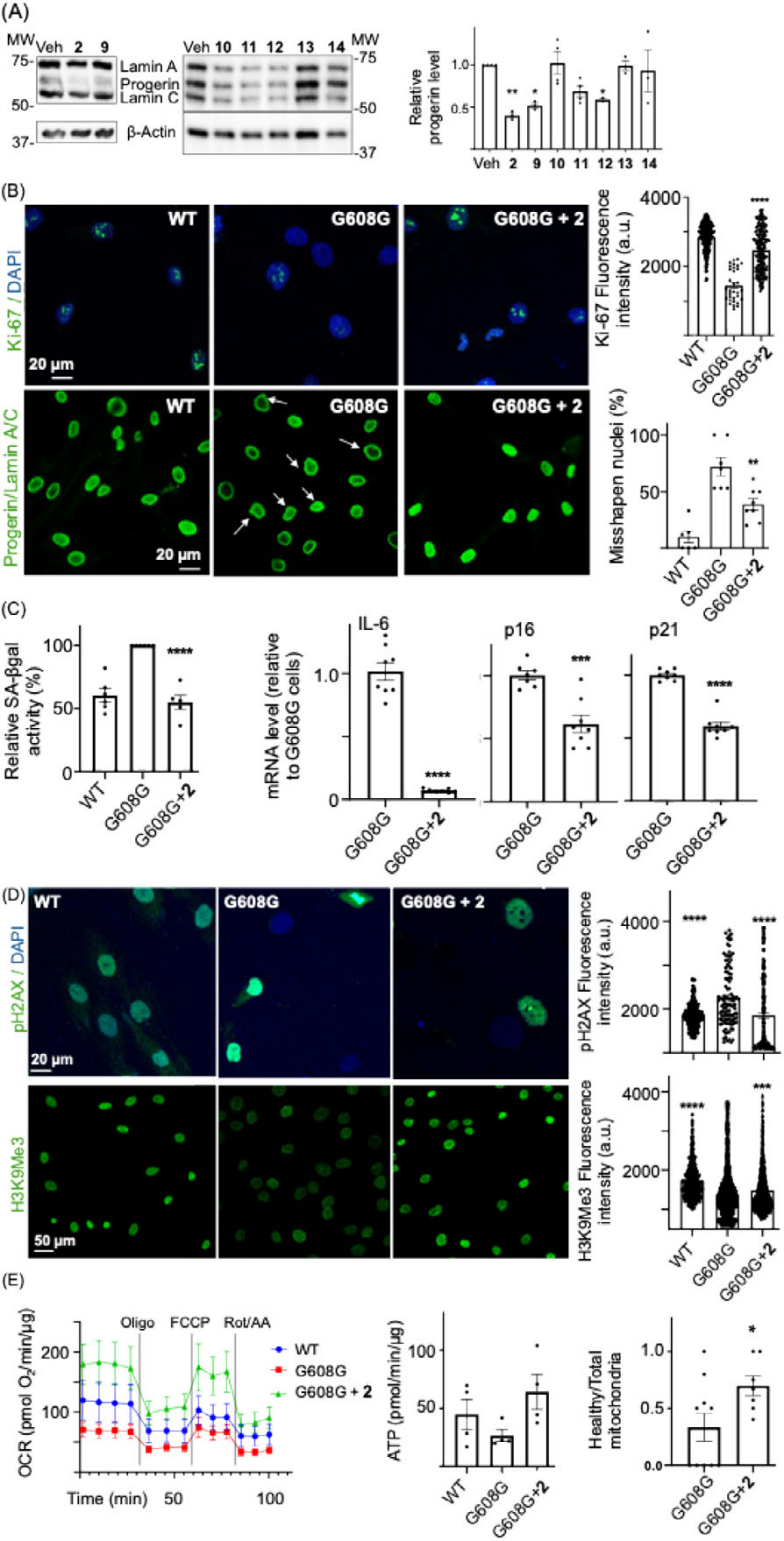
(A) Representative cropped immunoblots and progerin quantification in human *Lmna^G608G/G608G^* fibroblasts. MW, molecular weight (in kDa) markers. Bar graph shows mean±sem of 2 independent experiments performed in duplicate. (B) PROTAC **2** increases cell proliferation (upper panels) and decreases the number of misshapen nuclei (lower panels). White arrows highlight selected misshapen nuclei. (C) PROTAC **2** decreases senescence‐associated β‐galactosidase (SA‐βgal) activity, interleukin (IL) 6, p16 and p21 senescent markers. Bar graphs show mean±sem of 2 (SA‐βgal and p21) or 3 (IL‐6 and p16) independent experiments performed in triplicate. (D) PROTAC **2** decreases phosphorylated histone 2AX (pH2AX) and increases trimethylated histone 3 (H3K9Me3) levels. (E) PROTAC **2** increases the oxygen consumption rate (OCR), ATP production and the number of healthy mitochondria in human progeroid cells. Bar graphs show mean±sem of 2 independent experiments performed in duplicate. In all cases, HGADFN167 *Lmna^G608G/G608G^* (G608G) or HGFDFN168 *Lmna^+/+^* (WT) fibroblasts, provided by the Progeria Research Foundation, were incubated with vehicle or **2** (5 µm) for 20 days. Data were analysed using one‐way ANOVA followed by Dunnett's post–hoc test vs vehicle (panel A) or vs G608G cells (panels B‐E). ^*^, *p* < 0.05; ^**^, *p* < 0.01; ^***^, *p* < 0.001; ^****^, *p* < 0.0001. Microscopy images are representative of 2 independent experiments and corresponding bar graphs show mean±sem of 4 fields from 2 independent experiments.

Encouraged by the promising results obtained in the progeria mouse model, we next investigated a more clinically relevant phenotype using cells derived from progeria patients, generously provided by the Progeria Research Foundation. We evaluated the efficacy of the synthesized compounds in reducing progerin levels (Figure 6A) and showed that the synthesized compounds effectively reduce progerin levels in patient‐derived cells. Overall, most compounds retained a similar response profile to that observed in mouse cells, except for the DCAF16 recruiting PROTAC **12**, which exhibited significant activity in human cells. This result aligns with its reported nuclear efficacy [40]. Among the tested compounds, PROTAC **2** was the most potent in human cells, so it was prioritized for further studies to evaluate its potential to improve the global senescent phenotype in human progeroid cells.

HGPS fibroblasts exhibit a variety of cellular defects, including impaired proliferation, nuclear defects, premature senescence phenotype and mitochondrial dysfunction [45, 47, 48]. Since these defects result from the abnormal accumulation of progerin, we aimed to determine whether reducing progerin levels through treatment with PROTAC **2** could mitigate these alterations.

Accordingly, we treated fibroblasts derived from progeria patients with PROTAC **2**. Our results indicate that this treatment enhances cell proliferation, as evidenced by an increase in the expression of Ki‐67 (Figure 6B) and in the percentage of cells undergoing replication (phases S/G2/M of the cell cycle, Figure S5). Additionally, the compound significantly reduced the number of misshapen nuclei (Figure 6B) and the levels of important senescent markers such as SA‐βgal, IL‐6, p16 and p21 (Figure 6C). HGPS cells typically exhibit elevated levels of pH2AX [49], a well‐established marker of nuclear damage, specifically of DNA double‐strand breaks. Treatment with PROTAC **2** reduced pH2AX levels to those observed in WT cells (Figure 6D). In addition, HGPS cells often show a marked loss of histone H3 lysine 9 trimethylation (H3K9Me3) [50], a feature that was reversed by treatment with PROTAC **2**, leading to significantly increased H3K9Me3 levels (Figure 6D). HGPS cells exhibit significant mitochondrial dysfunction, characterized by increased oxidative stress and impaired basal mitochondrial respiration [45]. To further investigate mitochondrial function, we measured cellular oxygen consumption rates (OCR) as an indicator of overall respiratory chain activity. Our results demonstrate that treatment with PROTAC **2** enhances OCR in human progeroid cells (Figure 6E, left), basal respiration (Figure S6), ATP production (Figure 6E, middle) and the number of healthy mitochondria as assessed by electron microscopy (Figure 6E, right; Figure S5 for representative images). Collectively, the global improvement in mitochondrial function, along with the previously observed restoration of nuclear integrity and proliferation capacity, underscores the potential of PROTAC **2** to induce a significant and overall improvement of the cellular phenotype of patient‐derived cells. In this context, and to gain deeper insights into the molecular mechanisms underlying the observed improvements in the HGPS cellular phenotype following treatment, we performed global transcriptomic analysis.

### 2.4 Global Transcriptomic Analysis of PROTAC 2‐Treated Cells

**FIGURE 7:**
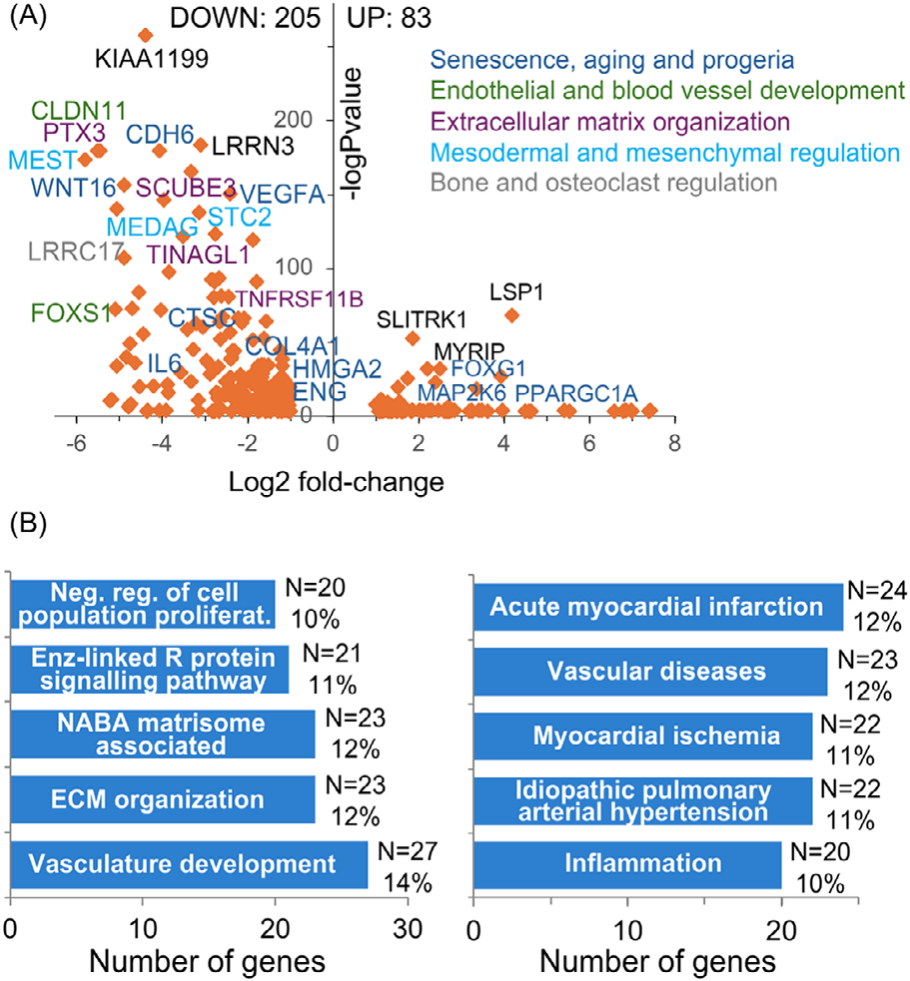
(A) Volcano plot of differentially expressed transcripts in HGADNF167 *Lmna^G608G/G608G^* fibroblasts treated with **2** vs non‐treated. (B) Top five clusters identified in pathway and process (left panel, *p* value<10^−8^) and DISGENET (right panel, *p* value<10^−6^) enrichment analysis using Metascape.

To establish the broader impact of PROTAC **2** at the molecular level, we performed detailed gene expression and pathway‐centric enrichment analysis. HGADFN167 cells were treated with either vehicle or compound **2** (5 µm) for 20 days before RNA was extracted for RNA‐seq analysis (3 independent biological samples per condition). Defining an adjusted *p*‐value<0.01 and a log2 fold‐change (FC)>1.0 or <−1.0, which represents those genes which expression has increased or decreased by more than two‐fold (upregulated and downregulated genes, respectively), we found 288 differentially expressed genes in treated HGPS fibroblasts vs non‐treated cells. Of these, 83 were upregulated and 205 were downregulated (Figure 7A). Among the most significantly downregulated genes, we could identify some genes involved in progeria (WNT16, VEGFA and HMGA2) and in senescence and aging (IL6, CTSC, PTGS2, SLC25A4, COL4A1, ENG, TBX2, MAP2K6, FOXS1, PPARGC1A). Remarkably, IL6 identification supports the qPCR results previously obtained (Figure 6C). Previous genome‐scale expression profiling of HGPS reveals widespread transcriptional misregulation related to DNA repair, vascular and bone development, extracellular matrix (ECM) organization, mesodermal/mesenchymal defects and lipid homeostasis alterations [51, 52] In line with these findings, we identified several genes associated with endothelial and blood vessel development (CLDN11 and FOXS1), ECM organization (PTX3, SCUBE3, TINAGL1, and TNFRSF11B), and mesodermal and mesenchymal regulation (MEST, MEDAG, and STC2). Pathway and process enrichment analysis using Metascape [53] revealed significant enrichment of Gene Ontology (GO) terms related to ECM organization and the matrisome (term that encompasses genes encoding ECM‐associated proteins, including ECM‐affiliated proteins, ECM regulators, secreted factors), and those involved in vasculature development (Figure 7B, left panel, and Figure S7).

Interestingly, several genes associated with the negative regulation of cell proliferation were also identified, supporting the observed increase in proliferation indicated by the upregulation of Ki‐67 expression (Figures 5A and 6B). Furthermore, enrichment analysis using the gene‐disease association database DISGENET [54] suggests the specific downregulation of genes linked to cardiac and vascular diseases (Figure 7B, right panel), which represent the most critical health issue in progeria.

### 2.5 In Vivo Studies

**FIGURE 8:**
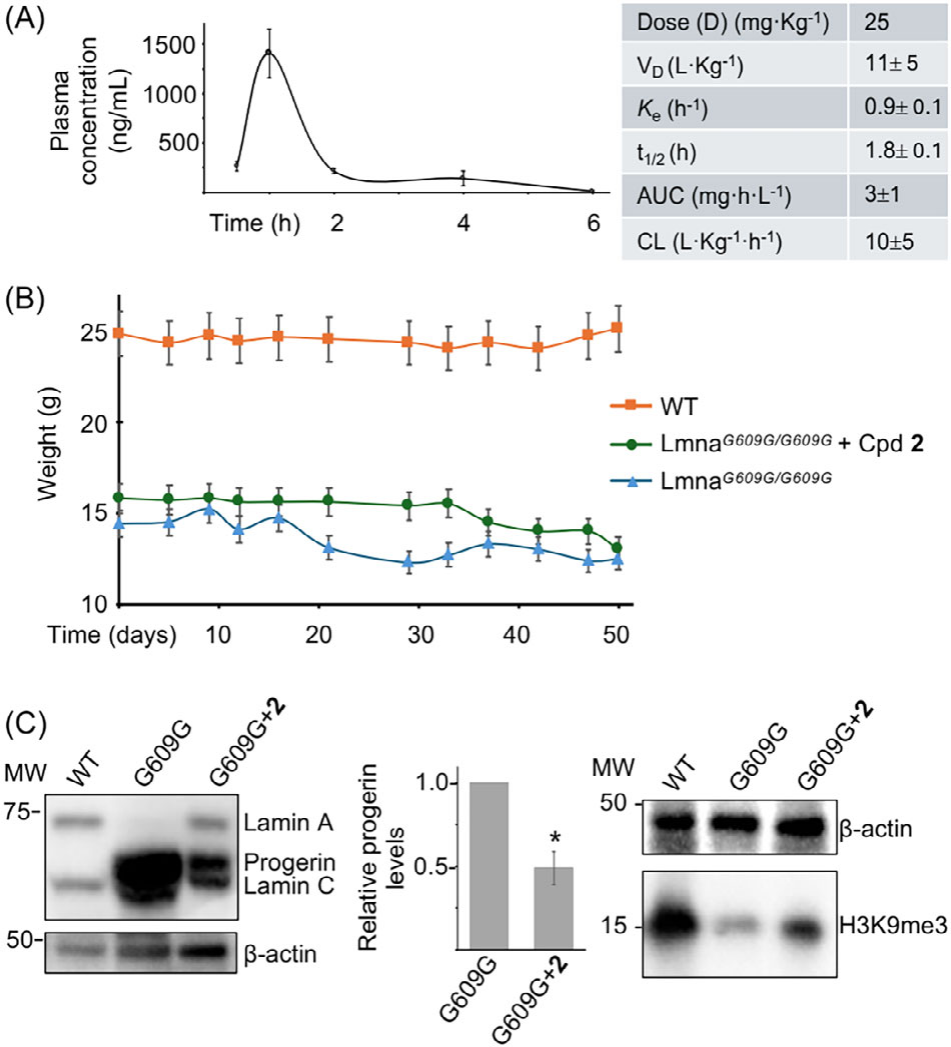
(A) In vivo pharmacokinetic (PK) analysis of compound **2** (mean±sem, n = 2 per time point). (B) Body weight throughout the 7‐week treatment. Mice of the indicated genotypes (mean±sem, n = 2) were treated with compound **2** or vehicle, starting at 6 weeks of age. (C) Progerin and H3K9Me3 levels in lung and liver, respectively, of mice treated for 7 weeks. Representative cropped immunoblots are shown. MW, molecular weight (in kDa) markers. Bar graph shows mean±sem of 2 independent experiments performed in duplicate. ^*^, *p* < 0.05; two‐sided Student's *t*‐test.

Having demonstrated the efficacy of compound **2** in mouse and human progeroid cells, we evaluated its in vivo efficacy in progeroid *Lmna^G609G/G609G^* mice. The in vivo pharmacokinetic parameters showed that an intraperitoneal (i.p.) dose of 25 mg/kg was enough to reach significant levels of the compound 1 h after administration, close to 1500 ng/mL, and a half‐life of almost 2 h (Figure 8A). As no evident signs of toxicity were detected, this dose was subsequently administered three times per week during seven weeks. Weight was monitored during this time and at the end of the period mice were sacrificed and tissue was harvested to determine if PROTAC administration induced a decrease in the levels of progerin in vivo. Progeroid mice treated with compound **2** exhibited a trend toward improved body weight (Figure 8B) and, importantly, they exhibited reduced levels of progerin and increased levels of H3K9Me3 (Figure 8C), clearly supporting the in vivo efficacy of PROTAC **2**.

## 3 Conclusion

In summary, we report the first PROTACs specifically designed to target and degrade progerin, establishing a novel proof‐of‐concept therapeutic strategy for progeria. PROTAC **2** demonstrates that direct targeting of progerin is not only feasible, but also therapeutically relevant. Treatment with this compound significantly reduces progerin levels and improves the main phenotypic hallmarks of the disease not only in the mouse cellular models but also in HGPS patient‐derived cells. These include enhanced cellular proliferation, reduced expression of senescence markers, and improvement of nuclear and mitochondrial alterations. Moreover, transcriptomic profiling of treated patient‐derived cells reveals a partial normalization of gene expression patterns associated with the disease phenotype. Importantly, PROTAC **2** shows in vivo efficacy in progeroid mice, reducing the levels of progerin and increasing H3K9Me3 expression. Although current degradation levels are not yet maximal, our results provide compelling proof of concept for a PROTAC‐based approach to directly target disease‐driving protein aggregates. By targeting the root cause of HGPS, this approach may have significant translational relevance for future treatments aimed at correcting the main hallmarks of the disease and mitigating its progression.

## 4 Experimental Section

### 4.1 Compound Synthesis

The starting materials, reagents, and solvents were purchased as high‐grade commercial products from Sigma–Aldrich (Merck), Acros, ABCR, Fluorochem, Scharlab, or Panreac. For all final compounds, a purity of at least 95% was determined by HPLC‐MS using an Agilent 1200LC‐MSD VL instrument. Detailed synthetic protocols and characterization data are provided in the Supporting information.

### 4.2 Cell Lines

Mouse fibroblasts were grown in Dulbecco's modified eagle medium (DMEM, Gibco) supplemented with 10% heat inactivated fetal bovine serum (FBS, Gibco), 50 U/mL penicillin, and 50 µg/mL streptomycin (Invitrogen). Human progeroid HGADFN167 *Lmna^G608G/G608G^* (G608G) or healthy HGADFN168 *Lmna^+/+^* (WT) fibroblasts, were obtained from The Progeria Research Foundation (RRIDs CVCL_1Y92 and CVCL_1Y93, respectively) and cultured in DMEM with 20% heat inactivated FBS, 50 U/mL penicillin and 50 µg/mL streptomycin. Cells were incubated in a humidified atmosphere at 37°C in the presence of 5% CO_2_.

### 4.3 Ethic Statement for Animal Experiments

All scientific procedures with animals were conformed to EU Directive 2010/63 EU and Recommendation 2007/526/EC, enforced in Spanish law under Real Decreto 53/2013. Animal protocols were approved by the Committee of Animal Experimentation of Universidad Complutense de Madrid and the Animal Protection Area of the Comunidad Autónoma de Madrid (PROEX 114.4/24).

### 4.4 Statistical Analysis

GraphPad Prism (version 10.4.2) was used for statistical analysis. For data pre‐processing, normalization was applied where appropriate as indicated in the corresponding figure legends, and data were inspected for potential outliers. Results are presented as mean±standard error of the mean (SEM). The sample size (n) for each experimental group and statistical comparison is reported in the figure legends. Assumptions of normality and homogeneity of variance were confirmed before applying parametric tests. Significant differences were evaluated using two‐sided Student's *t*‐test (t‐test) or one‐way analysis of variance (ANOVA) followed by Dunnett's post–hoc multiple comparisons test. Statistical significance was assessed at an alpha level of 0.05. *p*‐values are presented as ^*^, *p* < 0.05; ^**^, *p* < 0.01; ^***^, *p* < 0.001; and ^****^, *p* < 0.0001.

## Funding

Comunidad de Madrid, SYG‐2024/SAL‐GL‐812; Ministerio de Universidades FPU18/05620; Ministerio de Ciencia e Innovación, PID2023‐151109OB‐I00, PID2022‐138797OB‐I00; Progeria Research Foundation PRF 2022‐84.

## Conflicts of Interest

The authors declare no conflicts of interest.

## Supporting information




**Supporting file**: advs74965‐sup‐0001‐SuppMat.pdf

## Data Availability

The data that support the findings of this study are available in the supplementary material of this article.

## References

[1] L. B. Gordon , W. T. Brown , F. S. Collins Hutchinson‐Gilford Progeria Syndrome. In editors: MP Adam , S. Bick , G. M. Mirzaa , et al., GeneReviews® [Internet]. Seattle (WA): University of Washington, Seattle; 2003) 1993–2026, https://www.ncbi.nlm.nih.gov/books/NBK1121/. 20301300

[2] N. J. Ullrich and L. B. Gordon , “Hutchinson‐Gilford Progeria Syndrome,” Handbook of Clinical Neurology 132 (2015): 249–264. 26564085 10.1016/B978-0-444-62702-5.00018-4

[3] L. B. Gordon , F. G. Rothman , C. Lopez‐Otin , and T. Misteli , “Progeria: A Paradigm for Translational Medicine,” Cell 156 (2014): 400–407. 24485450 10.1016/j.cell.2013.12.028 PMC6318797

[4] M. S. Ahmed , S. Ikram , N. Bibi , and A. Mir , “Hutchinson‐Gilford Progeria Syndrome: A Premature aging Disease,” Molecular Neurobiology 55 (2018): 4417–4427. 28660486 10.1007/s12035-017-0610-7

[5] M. Suzuki , L. J. B. Jeng , S. Chefo , et al., “FDA Approval Summary for Lonafarnib (Zokinvy) for the Treatment of Hutchinson‐Gilford Progeria Syndrome and Processing‐deficient Progeroid Laminopathies,” Genetics in Medicine 25 (2023): 100335. 36507973 10.1016/j.gim.2022.11.003

[6] L. B. Gordon , H. Shappell , J. Massaro , et al., “Association of Lonafarnib Treatment vs no Treatment with Mortality Rate in Patients with Hutchinson‐Gilford Progeria Syndrome,” Jama 319 (2018): 1687–1695. 29710166 10.1001/jama.2018.3264 PMC5933395

[7] X. Chen , H. Yao , V. Andrés , M. O. Bergo , and M. Kashif , “Status of Treatment Strategies for Hutchinson–Gilford Progeria Syndrome with a Focus on Prelamin: A Posttranslational Modification,” Basic & Clinical Pharmacology & Toxicology 131 (2022): 217–223. 35790078 10.1111/bcpt.13770 PMC9795874

[8] J. Macicior , B. Marcos‐Ramiro , and S. Ortega‐Gutiérrez , “Small‐Molecule Therapeutic Perspectives for the Treatment of Progeria,” International Journal of Molecular Sciences 22 (2021): 7190. 34281245 10.3390/ijms22137190 PMC8267806

[9] M. Olive , I. Harten , R. Mitchell , et al., “Cardiovascular pathology in Hutchinson‐Gilford progeria: Correlation with the vascular pathology of aging,” Arteriosclerosis, Thrombosis, and Vascular Biology 30 (2010): 2301–2309. 20798379 10.1161/ATVBAHA.110.209460 PMC2965471

[10] D. McClintock , D. Ratner , M. Lokuge , et al., “The Mutant form of Lamin A that Causes Hutchinson‐Gilford Progeria is a Biomarker of Cellular aging in Human Skin,” PLoS ONE 2 (2007): 1269. 10.1371/journal.pone.0001269 PMC2092390 18060063

[11] A. De Sandre‐Giovannoli , R. Bernard , P. Cau , et al., “Lamin A Truncation in Hutchinson‐Gilford Progeria,” Science 300 (2003): 2055. 12702809 10.1126/science.1084125

[12] M. Eriksson , W. T. Brown , L. B. Gordon , et al., “Recurrent de Novo Point Mutations in Lamin A Cause Hutchinson–Gilford Progeria Syndrome,” Nature 423 (2003): 293–298. 12714972 10.1038/nature01629 PMC10540076

[13] S. H. Yang , M. O. Bergo , J. I. Toth , et al., “Blocking Protein Farnesyltransferase improves Nuclear Blebbing in Mouse Fibroblasts with a Targeted Hutchinson–Gilford Progeria Syndrome Mutation,” Proceedings of the National Academy of Sciences 102 (2005): 10291–10296. 10.1073/pnas.0504641102 PMC1174929 16014412

[14] B. H. Kim , Y. H. Chung , T. G. Woo , S. M. Kang , S. Park , and B. J. Park , “Progerin, an Aberrant Spliced form of Lamin A, is a Potential Therapeutic Target for HGPS,” Cells 12 (2023): 2299. 37759521 10.3390/cells12182299 PMC10527460

[15] B. Marcos‐Ramiro , A. Gil‐Ordóñez , N. I. Marín‐Ramos , et al., “Isoprenylcysteine Carboxylmethyltransferase‐Based Therapy for Hutchinson–Gilford Progeria Syndrome,” ACS Central Science 7 (2021): 1300–1310. 34471675 10.1021/acscentsci.0c01698 PMC8393201

[16] S. M. Kang , M. H. Yoon , J. Ahn , et al., “Progerinin, an Optimized Progerin‐Lamin A Binding Inhibitor, Ameliorates Premature Senescence Phenotypes of Hutchinson‐Gilford Progeria Syndrome,” Communications Biology 4 (2021): 5, 10.1038/s42003-42020-01540-w. 33398110 PMC7782499

[17] M. R. Erdos , W. A. Cabral , U. L. Tavarez , et al., “A Targeted Antisense Therapeutic Approach for Hutchinson–Gilford Progeria Syndrome,” Nature Medicine 27 (2021): 536–545. 10.1038/s41591-021-01274-0 PMC10158310 33707773

[18] M. Puttaraju , M. Jackson , S. Klein , et al., “Systematic Screening Identifies Therapeutic Antisense Oligonucleotides for Hutchinson–Gilford Progeria Syndrome,” Nature Medicine 27 (2021): 526–535. 10.1038/s41591-021-01262-4 PMC10167920 33707772

[19] F. G. Osorio , C. L. Navarro , J. Cadinanos , et al., “Splicing‐Directed Therapy in a new Mouse Model of Human Accelerated Aging,” Science Translational Medicine 3 (2011): 106ra107. 10.1126/scitranslmed.3002847 22030750

[20] O. Santiago‐Fernandez , F. G. Osorio , V. Quesada , et al., “Development of a CRISPR/Cas9‐based therapy for Hutchinson–Gilford progeria syndrome,” Nature Medicine 25 (2019): 423–426. 10.1038/s41591-018-0338-6 PMC6546610 30778239

[21] L. W. Koblan , M. R. Erdos , C. Wilson , et al., “In vivo base editing rescues Hutchinson–Gilford progeria syndrome in mice,” Nature 589 (2021): 608–614. 33408413 10.1038/s41586-020-03086-7 PMC7872200

[22] G. M. Burslem and C. M. Crews , “Proteolysis‐targeting Chimeras as Therapeutics and Tools for Biological Discovery,” Cell 181 (2020): 102–114. 31955850 10.1016/j.cell.2019.11.031 PMC7319047

[23] M. Békés , D. R. Langley , and C. M. Crews , “PROTAC Targeted Protein Degraders: The Past is Prologue,” Nature Reviews Drug Discovery 21 (2022): 181–200. 35042991 10.1038/s41573-021-00371-6 PMC8765495

[24] X. Liu and A. Ciulli , “Proximity‐based Modalities for Biology and Medicine,” ACS Central Science 9 (2023): 1269–1284. 37521793 10.1021/acscentsci.3c00395 PMC10375889

[25] X. Zhang , Z. Song , X. Zhang , et al., “Unconventional PROTACs for Targeted Protein Degradation in Cancer Therapy,” Angewandte Chemie International Edition 64 (2025): 202507702. 10.1002/anie.202507702 40492592

[26] S. M. Gough , J. J. Flanagan , J. Teh , et al., “Oral Estrogen Receptor PROTAC Vepdegestrant (ARV‐471) is highly Efficacious as Monotherapy and in Combination with CDK4/6 or PI3K/mTOR Pathway Inhibitors in Preclinical ER+ breast Cancer Models,” Clinical Cancer Research 30 (2024): 3549–3563. 38819400 10.1158/1078-0432.CCR-23-3465 PMC11325148

[27] M. Campone , M. De Laurentiis , K. Jhaveri , et al., “Vepdegestrant, a PROTAC Estrogen Receptor Degrader, in Advanced Breast Cancer, in advanced breast cancer,” New England Journal of Medicine 393 (2025): 556–568. 40454645 10.1056/NEJMoa2505725

[28] X. Zheng , N. Ji , V. Campbell , et al., “Discovery of KT‐474─a Potent, Selective, and Orally Bioavailable IRAK4 Degrader for the Treatment of Autoimmune Diseases,” Journal of Medicinal Chemistry 67 (2024): 18022–18037. 39151120 10.1021/acs.jmedchem.4c01305 PMC11513890

[29] Y. Xiao , Y. Yuan , Y. Liu , et al., “Targeted protein Degradation: Current and Emerging Approaches for E3 Ligase Deconvolution,” Journal of Medicinal Chemistry 67 (2024): 11580–11596. 38981094 10.1021/acs.jmedchem.4c00723 PMC12912787

[30] T. Ishida and A. Ciulli , “E3 ligase ligands for PROTACs: How they were found and how to Discover new ones,” SLAS Discovery 26 (2021): 484–502. 33143537 10.1177/2472555220965528 PMC8013866

[31] M. J. Bond and C. M. Crews , “Proteolysis Targeting Chimeras (PROTACs) come of age: Entering the Third Decade of Targeted Protein Degradation,” RSC Chemical Biology 2 (2021): 725–742. 34212149 10.1039/d1cb00011j PMC8190915

[32] T. A. Bemis , J. J. La Clair , and M. D. Burkart , “Unraveling the role of Linker Design in Proteolysis Targeting Chimeras,” Journal of Medicinal Chemistry 64 (2021): 8042–8052. 34106704 10.1021/acs.jmedchem.1c00482 PMC10790565

[33] C. E. Hendrick , J. R. Jorgensen , C. Chaudhry , et al., “Direct‐to‐Biology Accelerates PROTAC Synthesis and the Evaluation of Linker effects on Permeability and Degradation,” ACS Medicinal Chemistry Letters 13 (2022): 1182–1190. 35859867 10.1021/acsmedchemlett.2c00124 PMC9290060

[34] S. J. Lee , Y. S. Jung , M. H. Yoon , et al., “Interruption of Progerin–Lamin A/C Binding Ameliorates Hutchinson‐Gilford Progeria Syndrome Phenotype,” Journal of Clinical Investigation 126 (2016): 3879–3893. 27617860 10.1172/JCI84164 PMC5096810

[35] J. Macicior , D. Fernández , and S. Ortega‐Gutiérrez , “A new Fluorescent Probe for the Visualization of Progerin,” Bioorganic Chemistry 142 (2024): 106967. 37979321 10.1016/j.bioorg.2023.106967

[36] E. F. Douglass , C. J. Miller , G. Sparer , H. Shapiro , and D. A. Spiegel , “A Comprehensive Mathematical Model for Three‐Body Binding Equilibria,” Journal of the American Chemical Society 135 (2013): 6092–6099. 23544844 10.1021/ja311795d PMC3717292

[37] J. Hasper , K. Welle , K. Swovick , J. Hryhorenko , S. Ghaemmaghami , and A. Buchwalter , “Long Lifetime and Tissue‐Specific Accumulation of Lamin A/C in Hutchinson‐Gilford Progeria Syndrome,” Journal of Cell Biology 223 (2024): e202307049, 10.1083/jcb.202307049. 37966721 PMC10651395

[38] D. Gabriel , D. Roedl , L. B. Gordon , and K. Djabali , “Sulforaphane Enhances Progerin Clearance in Hutchinson– Gilford Progeria Fibroblasts,” Aging Cell 14 (2015): 78–91. 25510262 10.1111/acel.12300 PMC4326906

[39] J. Min , A. Mayasundari , F. Keramatnia , et al., “Phenyl‐Glutarimides: Alternative Cereblon Binders for the Design of PROTACs,” Angewandte Chemie International Edition 60 (2021): 26663–26670. 34614283 10.1002/anie.202108848 PMC8648984

[40] X. Zhang , V. M. Crowley , T. G. Wucherpfennig , M. M. Dix , and B. F. Cravatt , “Electrophilic PROTACs that Degrade Nuclear Proteins by Engaging DCAF16,” Nature Chemical Biology 15 (2019): 737–746. 31209349 10.1038/s41589-019-0279-5 PMC6592777

[41] E. Camafeita , I. Jorge , J. Rivera‐Torres , V. Andrés , and J. Vázquez , “Quantification of Farnesylated Progerin in Hutchinson‐Gilford Progeria Patient Cells by Mass Spectrometry,” International Journal of Molecular Sciences 23 (2022): 11733, 10.3390/ijms231911733. 36233036 PMC9569443

[42] A. Pike , B. Williamson , S. Harlfinger , S. Martin , and D. F. McGinnity , “Optimising Proteolysis‐Targeting Chimeras (PROTACs) for Oral Drug Delivery: A Drug Metabolism and Pharmacokinetics Perspective,” Drug Discovery Today 25 (2020): 1793–1800. 32693163 10.1016/j.drudis.2020.07.013

[43] L. M. Luh , U. Scheib , K. Juenemann , L. Wortmann , M. Brands , and P. M. Cromm , “Prey for the Proteasome: Targeted Protein Degradation—A Medicinal Chemist's Perspective,” Angewandte Chemie International Edition 59 (2020): 15448–15466. 32428344 10.1002/anie.202004310 PMC7496094

[44] X. Liu , A. F. Kalogeropulou , S. Domingos , et al., “Discovery of XL01126: A Potent, Fast, Cooperative, Selective, Orally Bioavailable, and Blood–Brain Barrier Penetrant PROTAC Degrader of Leucine‐Rich Repeat Kinase 2,” Journal of the American Chemical Society 144 (2022): 16930–16952. 36007011 10.1021/jacs.2c05499 PMC9501899

[45] J. Rivera‐Torres , R. Acin‐Perez , P. Cabezas‐Sanchez , et al., “Identification of Mitochondrial Dysfunction in Hutchinson–Gilford Progeria Syndrome through use of Stable isotope Labeling with Amino Acids in Cell Culture,” Journal of Proteomics 91 (2013): 466–477. 23969228 10.1016/j.jprot.2013.08.008

[46] P. Scaffidi and T. Misteli , “Lamin A‐Dependent Nuclear Defects in Human Aging,” Science 312 (2006): 1059–1063. 16645051 10.1126/science.1127168 PMC1855250

[47] P. Scaffidi and T. Misteli , “Reversal of the Cellular Phenotype in the Premature Aging Disease Hutchinson‐Gilford Progeria Syndrome,” Nature Medicine 11 (2005): 440–445. 10.1038/nm1204 PMC1351119 15750600

[48] R. D. Goldman , D. K. Shumaker , M. R. Erdos , et al., “Accumulation of Mutant Lamin A Causes Progressive changes in Nuclear Architecture in Hutchinson–Gilford Progeria Syndrome,” Proceedings of the National Academy of Sciences 101 (2004): 8963–8968. 10.1073/pnas.0402943101 PMC428455 15184648

[49] P. R. Musich and Y. Zou , “DNA‐Damage Accumulation and Replicative Arrest in Hutchinson–Gilford Progeria Syndrome,” Biochemical Society Transactions 39 (2011): 1764–1769. 22103522 10.1042/BST20110687 PMC4271832

[50] D. K. Shumaker , T. Dechat , A. Kohlmaier , et al., “Mutant Nuclear Lamin A Leads to Progressive Alterations of Epigenetic Control in Premature Aging,” Proceedings of the National Academy of Sciences 103 (2006): 8703–8708. 10.1073/pnas.0602569103 PMC1472659 16738054

[51] A. B. Csoka , S. B. English , C. P. Simkevich , et al., “Genome‐Scale Expression Profiling of Hutchinson–Gilford Progeria Syndrome Reveals Widespread Transcriptional Misregulation Leading to Mesodermal/Mesenchymal Defects and Accelerated Atherosclerosis,” Aging Cell 3 (2004): 235–243. 15268757 10.1111/j.1474-9728.2004.00105.x

[52] R. San Martin , P. Das , J. T. Sanders , A. M. Hill , and R. P. McCord , “Transcriptional Profiling of Hutchinson‐Gilford Progeria Syndrome Fibroblasts Reveals Deficits in Mesenchymal Stem Cell Commitment to differentiation Related to Early Events in Endochondral Ossification,” eLife 11 (2022): 81290. 10.7554/eLife.81290 PMC9833827 36579892

[53] Y. Zhou , B. Zhou , L. Pache , et al., “Metascape provides a Biologist‐Oriented Resource for the Analysis of Systems‐Level Datasets,” Nature communications 10 (2019): 1523. 10.1038/s41467-019-09234-6 PMC6447622 30944313

[54] J. Piñero , J. M. Ramírez‐Anguita , J. Saüch‐Pitarch , et al., “The DisGeNET knowledge Platform for Disease Genomics: 2019 Update,” Nucleic Acids Research 48 (2020): D845. 31680165 10.1093/nar/gkz1021 PMC7145631

[55] C. Steinebach , H. Kehm , S. Lindner , et al., “PROTAC‐Mediated Crosstalk between E3 Ligases,” Chemical Communications 55 (2019): 1821–1824. 30672516 10.1039/c8cc09541h

[56] T. Pinkert , D. Furkert , T. Korte , A. Herrmann , and C. Arenz , “Amplification of a FRET Probe by Lipid–Water Partition for the Detection of Acid Sphingomyelinase in Live Cells,” Angewandte Chemie International Edition 56 (2017): 2790–2794. 28156033 10.1002/anie.201611706

[57] X. Qiu , N. Sun , Y. Kong , Y. Li , X. Yang , and B. Jiang , “Chemoselective Synthesis of Lenalidomide‐based PROTAC Library using Alkylation Reaction,” Organic Letters 21 (2019): 3838–3841. 31066567 10.1021/acs.orglett.9b01326

[58] A. Bricelj , Y. L. Dora Ng , D. Ferber , et al., “Influence of Linker Attachment Points on the Stability and Neosubstrate Degradation of Cereblon Ligands,” ACS Medicinal Chemistry Letters 12 (2021): 1733–1738. 34795861 10.1021/acsmedchemlett.1c00368 PMC8591746

[59] G. Lamanna , C. R. Smulski , N. Chekkat , et al., “Multimerization of an Apoptogenic TRAIL‐Mimicking Peptide by Using Adamantane‐Based Dendrons,” Chemistry – A European Journal 19 (2013): 1762–1768. 23239456 10.1002/chem.201202415

[60] I. S. Shchelik and K. Gademann , “Thiol‐ and disulfide‐Containing Vancomycin Derivatives against Bacterial Resistance and Biofilm Formation,” ACS Medicinal Chemistry Letters 12 (2021): 1898–1904. 34917252 10.1021/acsmedchemlett.1c00455 PMC8667304

[61] M. X. Li , Y. Yang , Q. Zhao , et al., “Degradation versus Inhibition: Development of Proteolysis‐Targeting Chimeras for Overcoming Statin‐induced Compensatory Upregulation of 3‐hydroxy‐3‐methylglutaryl Coenzyme A Reductase,” Journal of Medicinal Chemistry 63 (2020): 4908–4928. 32321253 10.1021/acs.jmedchem.0c00339

[62] C. Steinebach , S. Lindner , N. D. Udeshi , et al., “Homo‐PROTACs for the Chemical Knockdown of Cereblon,” ACS Chemical Biology 13 (2018): 2771–2782. 30118587 10.1021/acschembio.8b00693

[63] C. Steinebach , Y. L. D. Ng , I. Sosič , et al., “Systematic Exploration of different E3 Ubiquitin Ligases: An approach towards Potent and Selective CDK6 Degraders,” Chemical Science 11 (2020): 3474–3486. 33133483 10.1039/d0sc00167h PMC7552917

[64] H. Vázquez‐Villa , A. Rueda‐Zubiaurre , D. Fernández , et al., “Chemical Probes for the Identification of the Molecular Targets of Honokiol,” European Journal of Medicinal Chemistry 283 (2025): 117102. 39616692 10.1016/j.ejmech.2024.117102

[65] R. Kreienkamp , S. Graziano , N. Coll‐Bonfill , et al., “A Cell‐Intrinsic Interferon‐like Response links Replication Stress to Cellular aging Caused by Progerin,” Cell Reports 22 (2018): 2006–2015. 29466729 10.1016/j.celrep.2018.01.090 PMC5848491

[66] Y. Kono , C. G. Pack , T. Ichikawa , et al., “Roles of the Lamin A‐Specific Tail Region in the Localization to sites of Nuclear Envelope Rupture,” PNAS Nexus 3 (2024): 527. 10.1093/pnasnexus/pgae527 PMC11645434 39677369

