# Supplementary material for: First Generation Proteolysis Targeting Chimeras (PROTACs) for the Treatment of Progeria
Source: Adv Sci (Weinh). 2026 Mar 23;13(32):e21608. doi: 10.1002/advs.202521608 (PMC13252604; doi:10.1002/advs.202521608)

First Generation Proteolysis Targeting Chimeras (PROTACs) for the Treatment of Progeria

Jon Macicior-Michelena[a,#], Marcelino Telechea[a,#], Daniel Fernández[a], Alba García-Martín[a], Ángeles Canales[a], and Silvia Ortega-Gutiérrez[a,*]

[a] Departamento de Química Orgánica, Facultad de Ciencias Químicas, Plaza de las Ciencias s/n, Universidad Complutense de Madrid, E-28040 Madrid, Spain

[#]Equal contribution

*Correspondence: siortega@ucm.es

Supporting Information

Table of Contents

1. Supporting Figures S1-S7	S3
2. Chemistry: synthetic procedures and compound characterization	S10
2.1. General synthetic procedures	S11
2.2. Synthesis of PROTAC **2**	S12
2.3. Synthesis of PROTAC **3**	S14
2.4. Synthesis of PROTAC **4**	S18
2.5. Synthesis of PROTAC **5**	S22
2.6. Synthesis of PROTAC **6**	S24
2.7. Synthesis of PROTAC **7**	S26
2.8. Synthesis of PROTAC **8**	S28
2.9. Synthesis of PROTAC **9**	S31
2.10. Synthesis of PROTAC **10**	S33
2.11. Synthesis of PROTAC **11**	S36
2.12. Synthesis of PROTAC **12**	S38
2.13. Synthesis of CRBN-based negative control **13** and **14**	S39
3. Saturation Transfer Difference (STD) NMR	S43
4. Cell lines and culture	S44

5. Cell viability assay S44
6. Immunoblot and immunocytofluorescence analysis S44
7. Senescence-associated (SA) β-galactosidase activity S45
8. RNA isolation and real time RT-qPCR analysis S46
9. Oxygen consumption rates (OCR) measurement S46
10. Cell cycle S47
11. Transmission electron microscopy S47
12. RNA-seq analysis S47
13. Immunoprecipitation S47
14. Cellular thermal shift assay S48
15. Animal experiments S48
16. ^{1}H-NMR and ^{13}C-NMR spectra of final compounds **2-14** S50
17. HPLC and MS data of final compounds **2-14** S63

1. Supporting Figures S1-S7.

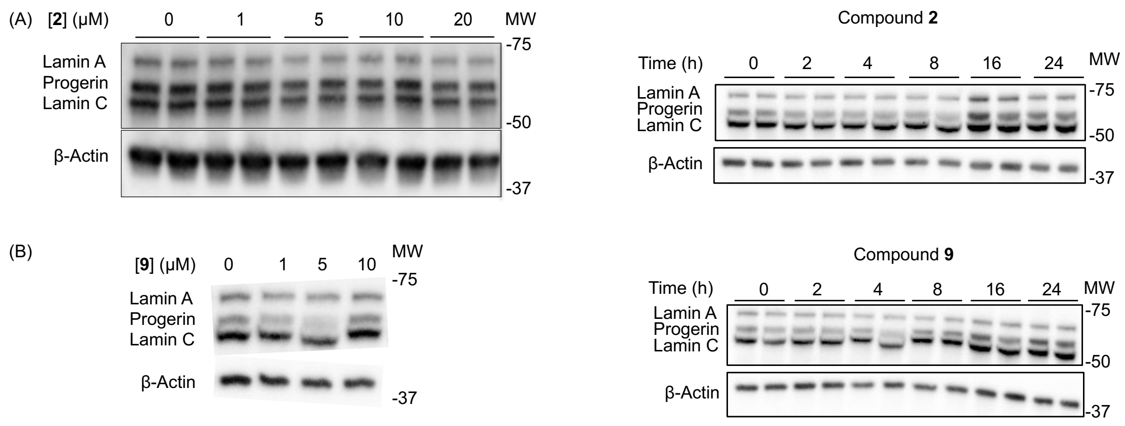

Supporting Figure S1. **Dose- and time-dependency studies.** Representative cropped immunoblots from *Lmna*$^{G609G/G609G}$ fibroblasts. (A) Cells were incubated with compound **2** at the indicated concentrations for 8 h (left panel) or with compound **2** at 5 μM for the indicated time (right panel). (B) Cells were incubated with compound **9** at the indicated concentrations for 8 h (left panel) or with compound **9** at 5 μM for the indicated time (right panel). MW, molecular weight (in kDa) markers.

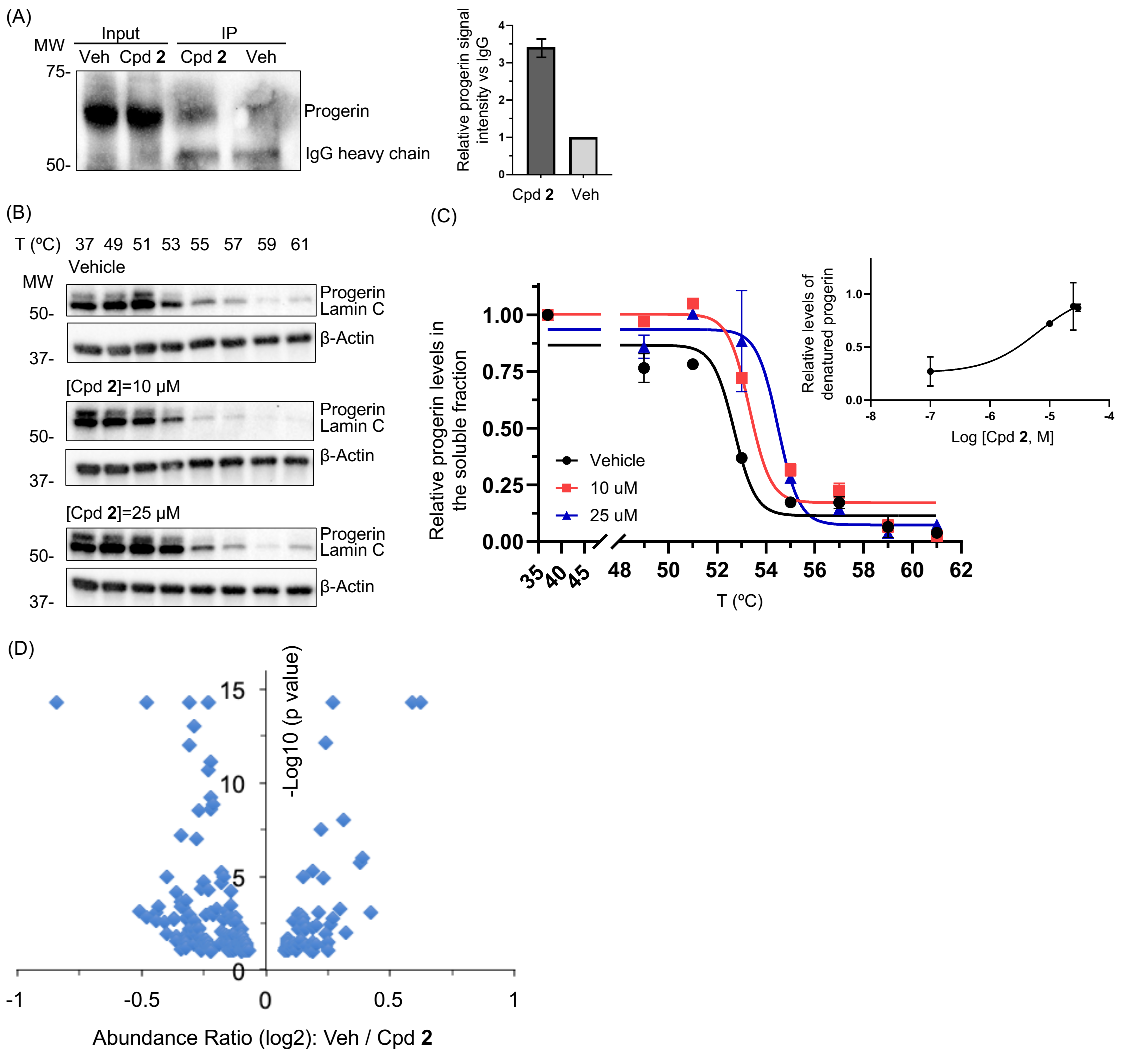

Supporting Figure S2. **Ternary complex formation, progerin engagement and global selectivity of compound 2.** (A) Progeroid *Lmna*$^{G606G/G609G}$ fibroblasts treated with vehicle (0.1% DMSO) or compound **2** (5 µM) were subjected to immunoprecipitation with an anti-CRBN antibody and then immunoblotted with an anti-progerin antibody. Bar graph represents mean±sem of 2 independent experiments. (B) Representative progerin immunoblotting of the soluble fraction of *Lmna*$^{G609G/G609G}$ cell homogenates treated with vehicle (0.1% DMSO) or compound **2** as indicated in the figure and heat-shocked at the indicated temperature. (C) Thermal denaturation curves of progerin in the presence of vehicle (black) or compound **2** at 10 µM (red, ΔTm=0.7±0.1 ºC) or 25 µM (blue, ΔTm=2.0±0.3 ºC). Inset shows the isothermal (at 53 ºC) dose-response fingerprint curve of compound **2** (IC_{50}=7±1 µM). Curves are generated and fitted using sigmoidal dose-response non-linear regression curve fit in GraphPad Prism. Symbols and associated error bars (when not enclosed by the symbol) represent average±SD from two independent experiments carried out in duplicate. (D) Vulcano plot shows 200 proteins identified with more than 1 unique peptide (total proteins identified in the experiment: >4000; experiment shows the obtained results from three independent samples treated with vehicle and three independent samples treated with 5 µM compound **2**).

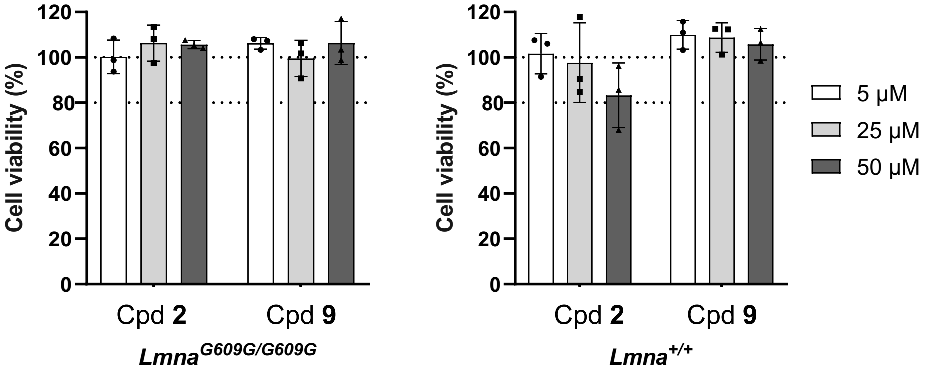

Supporting Figure S3. **Cellular cytotoxicity of compounds 2 and 9.** Viability of progeroid *Lmna*$^{G606G/G609G}$ and wild type *Lmna*$^{+/+}$ fibroblasts treated with compounds at 5-50 µM for 24 h. Viability is expressed as the percentage relative to cells treated with vehicle (0.1% DMSO) which are considered 100% viable. Error bars represent the average±SD from three independent experiments carried out in duplicate.

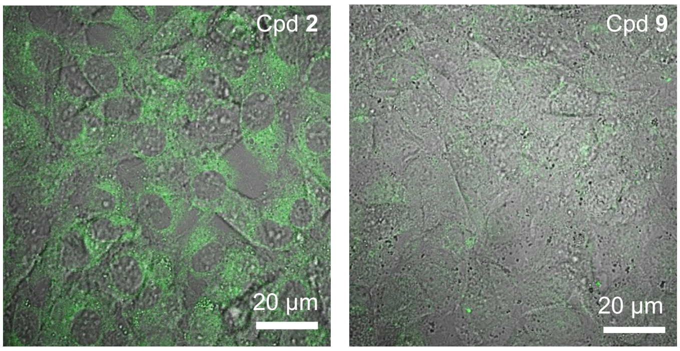

Supporting Figure S4. Cellular internalization of compounds 2 and 9. Live progeroid *Lmna*$^{G609G/G609G}$ mouse fibroblasts were incubated with 5 μM of indicated compound and images were taken every 5 minutes in a confocal fluorescence microscopy Olympus lx83 (λexc = 420 nm; λem = 530 nm). Shown image corresponds to 100 min incubation. Scale bar: 20 μm.

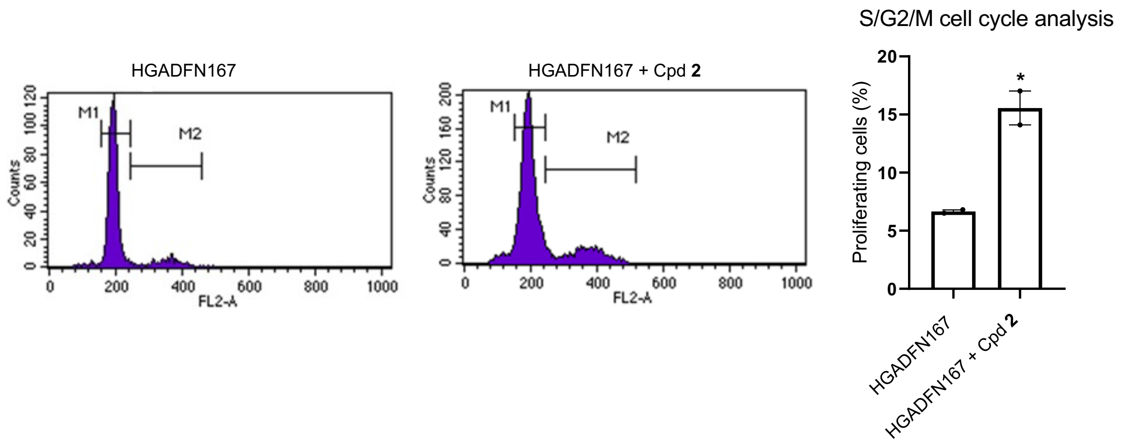

Supporting Figure S5. Cell cycle analysis histogram. Flow cytometry analysis of DNA content showing cell cycle distribution with peaks corresponding to G0/G1 (labeled as M1) and G2/M phases, and an intermediate region representing S phase (S/G2/M are labeled as M2). Human progeroid *Lmna*$^{G608G/G608G}$ fibroblasts (HGADFN167 cells, from The Progeria Research Foundation) were incubated with 5 µM of compound **2** for 20 days and analyzed for DNA content by flow cytometry in a FACSCalibur Flow cytometer (Becton Dickinson). Images shown correspond to one representative analysis and bar graph represents mean±sem of 2 independent experiments performed in duplicate. *p<0.05 vs HGADFN167 cells, one-way ANOVA.

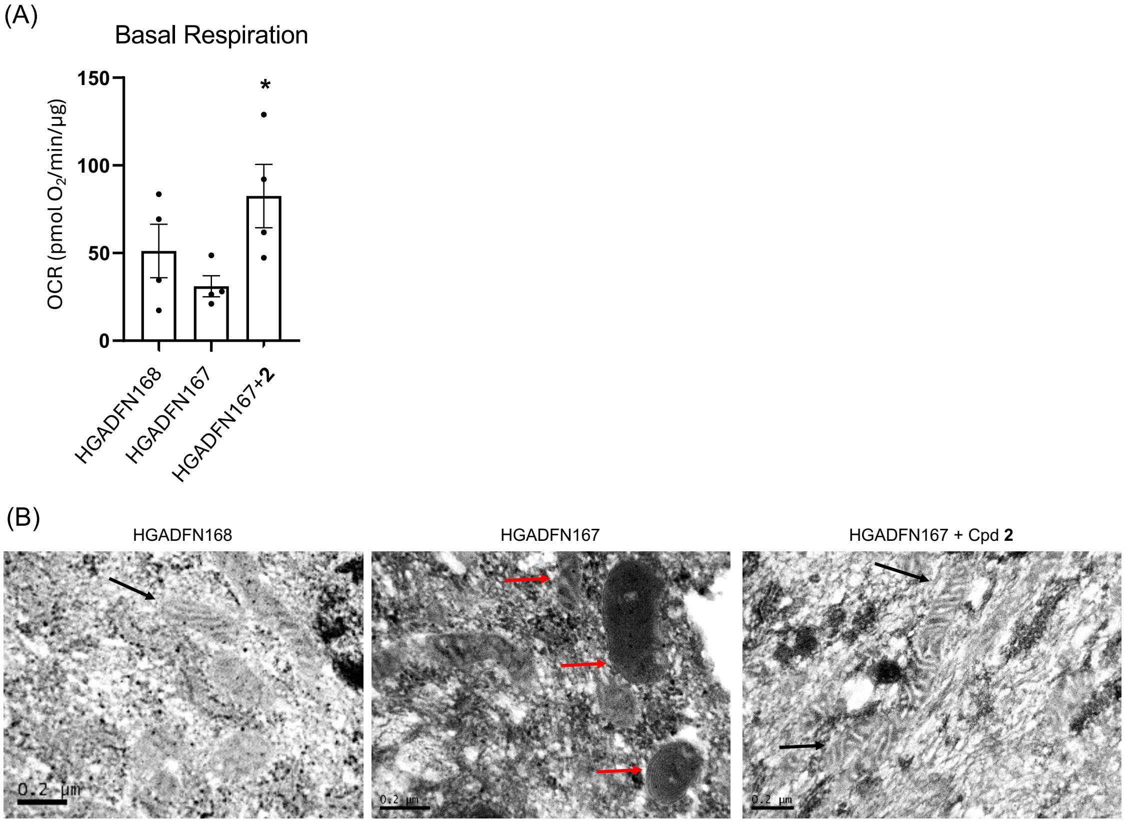

Supporting Figure S6. Basal respiration and electronic microscopy analysis of mitochondria in wild type and HGPS cells. (A) PROTAC **2** increases basal respiration (OCR, oxygen consumption rate) in human progeroid cells. Bar graphs show mean±sem of 2 independent experiments performed in duplicate. HGADFN167 *Lmna*$^{G608G/G608G}$ (progeroid) and HGADFN168 *Lmna*$^{+/+}$ (wild type) fibroblasts, provided by the Progeria Research Foundation, were incubated with vehicle or **2** (5 µM) for 20 days. *, p<0.05 vs G608G cells, one-way ANOVA. (B) HGADFN168 *Lmna*$^{+/+}$ (wild type) or HGADFN167 *Lmna*$^{G608G/G608G}$ (progeroid) fibroblasts provided by the Progeria Research Foundation were incubated with vehicle or compound **2** (5 µM) for 20 days before imaging. Mitochondria were evaluated for the integrity of the membrane, matrix and cristae, and were classified as mitochondria with normal morphology (black arrows) or damaged mitochondria (red arrows, with alterations in the membrane, presence of inclusions in the matrix and concentric cristae or "onion-like" mitochondria). Images correspond to representative transmission electron micrographs (scale bar, 200 nm) of two independent experiments.

(A)

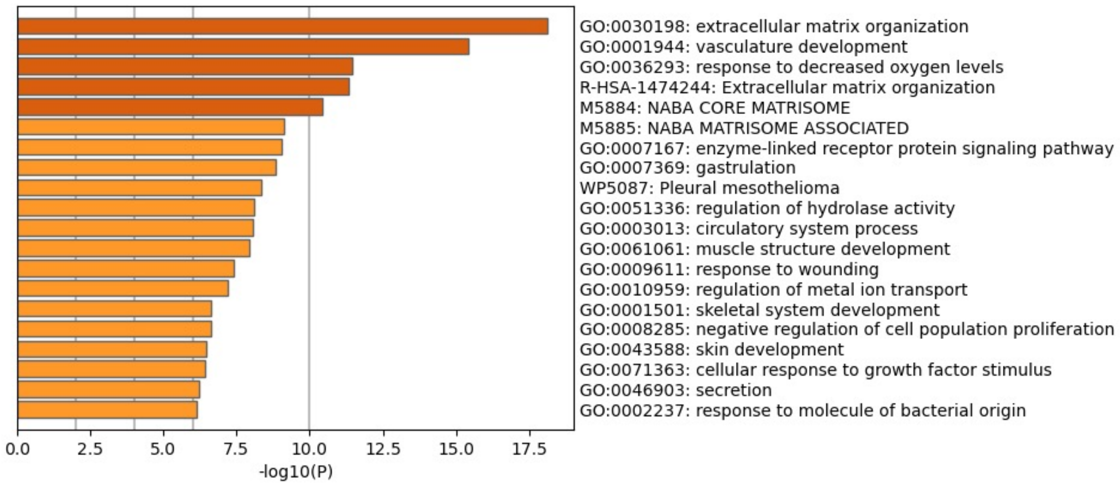

(B)

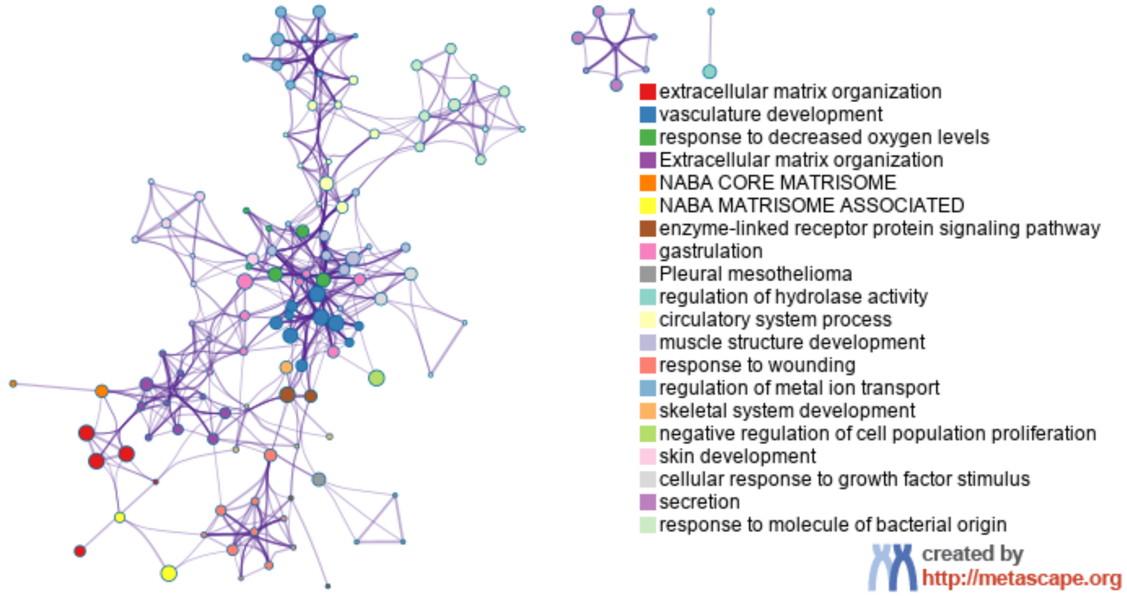

Supporting Figure S7. Statistically enriched ontology clusters. (A) Statistically enriched terms (GO/KEGG terms and canonical pathways) were identified, and accumulative hypergeometric p-values and enrichment factors were calculated and used for filtering. Remaining significant terms were then hierarchically clustered into a tree based on Kappa-statistical similarities among their gene memberships. Then 0.3 kappa score was applied as the threshold to cast the tree into term clusters. (B) Selection of a subset of representative terms from the full cluster and conversion into a network layout. Each term is represented by a circle node, where its size is proportional to the number of input genes fall under that term, and its color represent its cluster identity (i.e., nodes of the same color belong to the same cluster). Terms with a similarity score > 0.3 are linked by an edge (the thickness of the edge represents the similarity score). The network is visualized with Cytoscape with "force-directed" layout and with edge bundled for clarity. One term from each cluster is selected to have its term description shown as label. Analysis was carried out using Metascape.[29]

2. Chemistry: synthetic procedures and compound characterization.

The starting materials, reagents, and solvents were purchased as high-grade commercial products from Sigma-Aldrich (Merck), Acros, ABCR, Fluorochem, Scharlab, or Panreac. DCM (DCM), tetrahydrofuran (THF) and diethyl ether were dried using a Pure Solv^{TM} Micro 100 Liter solvent purification system. Analytical thin-layer chromatography (TLC) was run on Merck silica gel plates (Kieselgel 60 F254), with detection by UV light (λ = 254 nm), 5% ninhydrin solution in EtOH, or 10% vanillin solution in EtOH. Unless otherwise stated, compounds were purified in a Biotage® Selekt flash purification system using silica gel cartridges (Biotage Sfär, size particle 60 μM). MW irradiation reactions were carried out on a Biotage Initiator 2.5 reactor, using Biotage vials sealed with Biotage aluminum caps with septum. Hydrogenation reactions were carried out in a Thales Nano H-Cube® flow reactor, unless otherwise stated.

Melting points (mp) were determined on a Stuart Scientific electrothermal apparatus. Infrared (IR) spectra were measured on a Bruker Tensor 27 instrument equipped with a Specac ATR accessory of 4000-600 cm^{-1} transmission range; frequencies (ν) are expressed in cm^{-1}. IUPAC rules have been followed to name all organic compounds.

^{1}H-, and ^{13}C-NMR spectra were recorded on a Bruker Avance III 700 MHz (^{1}H, 700 MHz; ^{13}C, 175 MHz) or Bruker DPX 300 MHz (^{1}H, 300 MHz; ^{13}C, 75 MHz) at rt at the Universidad Complutense de Madrid (UCM) NMR core facility. Chemical shifts (δ) are expressed in parts per million (ppm), relative to the residual solvent peak for ^{1}H and ^{13}C nuclei ($CDCl_3$: δ_H = 7.26, δ_C = 77.16; Acetone-d_6: δ_H = 2.05, δ_C = 29.84, 206.26; MeOD-d_4: δ_H = 3.31, δ_C = 49.00); coupling constants (J) are shown in hertz (Hz). The following abbreviations are used to describe peak patterns when appropriate: s (singlet), d (doublet), t (triplet), q (quadruplet), quint (quintuplet), sext (sextuplet), m (multiplet) and br (broad). 2D NMR experiments -homonuclear correlation spectroscopy (H,H-COSY), heteronuclear multiple quantum correlation (HMQC), and heteronuclear multiple bond correlation (HMBC)- of all compounds were acquired to assign protons and carbons of structures. The following abbreviations have been used for the peak assignment: Ar (aryl), Cbz (carboxybenzyl), Bn (benzyl), Trz (triazole), Cy (cyclohexyl) and Ph (phenyl). If necessary, the relative configuration of the compounds was confirmed by 1D ^{1}H-NMR nuclear Overhauser effect (NOE) experiments, in which the signal of interest was irradiated with a selective pulse and NOE interactions were observed.

For all final compounds, a purity of at least 95% was determined by HPLC-MS using an Agilent 1200LC-MSD VL instrument. LC separation was achieved with an Eclipse XDB-C18 column (5 μm, 4.6 mm x 150 mm), together with a guard column (5 μm, 4.6 mm x 12.5 mm). The mobile phase consisted of water and acetonitrile (ACN) with 0.1% formic acid as solvent modifier, using the gradient indicated in Table 1. MS analysis was performed using an electrospray irradiation source. The capillary voltage was set to 3.0 kV and the fragmentor voltage to 72 eV. The drying gas temperature was 350 ^{o}C, the drying gas flow was 10 L/min, and the nebulizer pressure was 20 psi. Spectra were acquired in positive or negative ionization mode from 100 to 1200 *m/z* and in UV-mode at four different wavelengths (210, 230, 254, and 280 nm). High resolution mass

spectrometry (HRMS) was carried out in a Bruker Impact LC-MS QTOF instrument in positive mode with an ESI source at the UCM MS core facility.

Table 1. HPLC gradient for compound characterization.

t (min)	% B
0	0
2	0
8	80
10	100
18	100
22	0
25	0

2.1. General synthetic procedures

General procedure A: Nucleophilic aromatic substitution reaction. To a stirred solution of lenalidomide or 4-fluoro-thalidomide (1.0 eq) in DMF (5.0 mL/mmol) were added DIPEA (3.0 eq) and the protected linker (1.5 eq) and the resulting mixture was stirred at 180 ºC for 40 min in a MW reactor. After adding EtOAc, the mixture was washed with water (x6) and extracted with EtOAc (x3). After evaporating the solvent under reduced pressure, the crude was purified by flash chromatography.

General procedure B: *tert*-butyl ester removal. To a solution of the corresponding *tert*-butyl ester (1.0 eq) in DCM (10 mL/mmol), TFA (100 eq) was added and stirred at 25 ºC for 2 h. The reaction was monitored by TLC and when completed, the solvent was evaporated under reduced pressure. The crude was then dissolved in diethyl ether and the solvent evaporated under reduced pressure. This procedure was repeated three times to obtain the free carboxylic acid as a solid.

General procedure C: Steglich esterification. A mixture of the corresponding carboxylic acid (1.2 eq), DCC (1.2 eq) and DMAP (0.40 eq) were dissolved in anhydrous DCM (10 mL/mmol of alcohol). Then, the appropriate alcohol (1.0 eq) was added, and the reaction mixture was stirred at rt for 12 h. The crude was filtered and concentrated under reduced pressure, and the residue was purified by flash or column chromatography.

General procedure D for *O*-alkylation. The corresponding alcohol (1.0 eq) was dissolved in dry THF (2.5 mL/mmol) and the solution was cooled to 0 ºC. A mixture of potassium *tert*-butoxide (1.0 eq) in dry THF (2.0 mL/mmol) was added. The reaction mixture was stirred for 30 min at 0 ºC before *tert*-butyl bromoacetate (1.2 eq) was added. Stirring continued for 3 h at 0 ºC and for 18 h at rt. Subsequently, water was added, and the mixture was evaporated under reduced pressure. The residue was partitioned between EtOAc, and water and the aqueous layer was extracted with EtOAc (x3). The combined

organic layers were washed with brine, dried over Na_2SO_4, filtered and evaporated. The residue was purified by flash chromatography.

General procedure E for *O*-alkylation. The corresponding alcohol (1.0 eq) was dissolved in dry toluene (2.6 mL/mmol) and was cooled to 0 °C. A mixture of potassium *tert*-butoxide (1.5 eq) in *tert*-butanol (1.0 mL/mmol) was then added. The reaction mixture was stirred for 30 min at 0 °C before ethyl bromoacetate (2.0 eq) was added. Stirring of the suspension continued for 3 h at 0 °C and for 15 h at rt. Subsequently, 1 M HCl (10 mL) was added, and the aqueous layer was discarded. The organic layer was further washed with brine, dried over Na_2SO_4, filtered and evaporated. The residue was purified by flash chromatography.

General procedure F: Cbz group removal. The deprotection reaction was conducted in a Thales Nano H-Cube® dissolving the corresponding Cbz protected amine in EtOH (0.05 M). Pd/C-10% cartridges were used at 50 °C, Full H_2 and 1 mL/min. The solvent evaporated under reduced pressure.

2.2. Synthesis of PROTAC 2

***Tert*-butyl 6-aminohexanoate (15).** 6-aminohexanoic acid (1.0 eq, 200 mg, 1.52 mmol) and thionyl chloride (4.5 eq, 0.50 mL, 6.86 mmol) were added to a round bottom flask and under nitrogen atmosphere. This solution was stirred at 25 °C for 2 h, the mixture was concentrated under reduced pressure and a slurry of $NaHCO_3$ (2.0 eq, 256 mg, 3.05 mmol) in *tert*-butanol (5.7 eq, 0.80 mL, 8.69 mmol) was added. After stirring at rt for 2 h, *tert*-butanol was removed under reduced pressure at 40 °C and the crude was diluted with EtOAc and washed with 1 M NaOH (x3), water (x3) and brine. The organic phases were combined, dried over Na_2SO_4, filtered and concentrated to obtain compound **15** (200 mg, 1.07 mmol, 70% yield) as an oil.

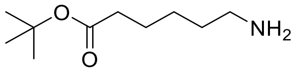

R_f: 0.25 (DCM/MeOH, 9:1). **IR (ATR, cm^{-1})**: ν 2976 (C-H), 2932 (C-H), 1724 (C=O), 1147 (C-O). **^{1}H-NMR (300 MHz, $CDCl_3$)**: δ 1.29 – 1.39 (m, 2H, H_4), 1.43 (s, 9H, $(CH_3)_3$), 1.41 – 1.51 (m, 2H, H_3), 1.52 – 1.65 (m, 2H, H_5), 1.92 (s, 2H, NH_2), 2.21 (t, *J* = 7.4 Hz, 2H, H_2), 2.69 (t, *J* = 6.9 Hz, 2H, H_6). **^{13}C-NMR (75 MHz, $CDCl_3$)**: δ 25.0 (C5), 26.5 (C4), 28.3 ($(CH_3)_3$), 33.2 (C3), 35.6 (C2), 42.0 (C6), 80.1 (<u>C</u>$(CH_3)_3$), 173.3 (CO). **HPLC (t_R, min):** 9.20. **MS (ESI, *m/z*, %):** 188.2 ($[M+H]^+$, 100), 132.2 ($[M+H-{}^tBu]^+$, 58).

***Tert*-butyl 6-{[2-(2,6-dioxopiperidin-3-yl)-1,3-dioxo-2,3-dihydro-1*H*-isoindol-4-yl]-amino}hexanoate (23).** Following general procedure A, 4-fluoro-thalidomide (100 mg, 0.362 mmol) was dissolved in DMF (1.8 mL) and DIPEA (0.20 mL, 1.09 mmol) and *tert*-butyl 6-aminohexanoate (101 mg, 0.540 mmol) were added. The resulting residue

was purified by flash chromatography (hex to hex/EtOAc, 4:6) to obtain compound **23** as a solid (67.0 mg, 0.148 mmol, 42% yield).

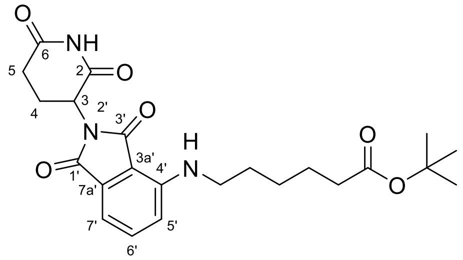

Mp: 80-82 ºC. **R$_f$**: 0.56 (Hex/EtOAc, 6:4). **IR (ATR, cm^{-1})**: ν 3395 (N-H), 3242 (=C-H), 2976 (C-H), 2932 (C-H), 1693 (C=O), 1150 (C-O). **^{1}H-NMR (300 MHz, CDCl$_3$)**: δ 1.37 – 1.48 (m, 2H, $C\underline{H}_2CH_2CH_2NH$), 1.43 (s, 9H, $(CH_3)_3$), 1.55 – 1.76 (m, 4H, $C\underline{H}_2CH_2C\underline{H}_2CH_2NH$), 2.00 – 2.19 (m, 1H, H_4), 2.23 (t, J = 7.3 Hz, 2H, $C\underline{H}_2COO$), 2.62 – 2.93 (m, 3H, H_4, H_5), 3.26 (t, J = 7.1 Hz, 2H, $C\underline{H}_2NH$), 4.84 – 4.97 (m, 1H, H_3), 6.22 (br s, 1H, ArN$\underline{H}$), 6.86 (dd, J = 8.6, 0.6 Hz, 1H, $H_{5'}$), 7.07 (dd, J = 7.1, 0.6 Hz, 1H, $H_{7'}$), 7.48 (dd, J = 8.5, 7.1 Hz, 1H, $H_{6'}$), 8.29 (s, 1H, NHCO). **^{13}C-NMR (75 MHz, CDCl$_3$)**: δ 22.9 (C4), 24.8 ($\underline{C}H_2CH_2CH_2CH_2NH$), 26.5 ($\underline{C}H_2CH_2CH_2NH$), 28.2 ($(CH_3)_3$), 29.2 ($\underline{C}H_2CH_2NH$), 31.5 (C5), 35.4 ($\underline{C}H_2CO$), 42.6 ($CH_2NH$), 49.0 (C3), 80.3 ($\underline{C}(CH_3)_3$), 110.0 (C3a'), 111.5 (C7'), 116.7 (C5'), 132.6 (7a'), 136.2 (C6'), 147.1 (C4'), 167.7 (C3'), 168.5 (C1'), 169.6 (C2), 171.3 (C6), 173.0 (COO). **HPLC (t$_R$, min):** 14.16. **MS (ESI, *m/z*, %):** 388.2 ([M+H-^{t}Bu]$^+$, 100).

6-{[2-(2,6-Dioxopiperidin-3-yl)-1,3-dioxo-2,3-dihydro-1*H*-isoindol-4-yl]amino}-hexanoic acid (24). Following general procedure B, *tert*-butyl ester **23** (60.0 mg, 0.143 mmol) was dissolved in DCM (1.4 mL) and TFA (1.0 mL, 13.5 mmol) was added. Compound **24** was obtained as a solid (48.0 mg, 0.121 mmol, 91% yield).

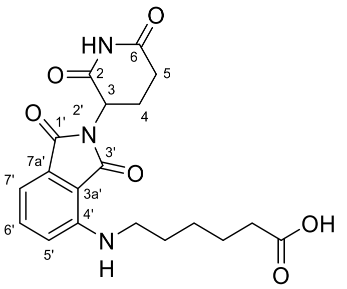

Mp: 164-166 ºC. **R$_f$**: 0.25 (DCM/MeOH, 9:1). **IR (ATR, cm^{-1})**: ν 3384 (N-H), 3218 (=C-H), 2932 (C-H), 1692 (C=O), 1623 (C=O), 1196 (C-O). **^{1}H-NMR (300 MHz, Acetone-*d$_6$*)**: δ 1.38 – 1.57 (m, 2H, $C\underline{H}_2CH_2CH_2NH$), 1.59 – 1.81 (m, 4H, $C\underline{H}_2CH_2C\underline{H}_2CH_2NH$), 2.14 – 2.26 (m, 1H, H_4), 2.31 (d, J = 7.4 Hz, 2H, $C\underline{H}_2CO$), 2.64 – 2.85 (m, 2H, H_4, H_5), 2.84 – 3.16 (m, 1H, H_5), 3.39 (t, J = 7.1 Hz, 2H, $C\underline{H}_2NH$), 5.06 (dd, J = 12.5, 5.4 Hz, 1H, H_3), 7.03 (d, J = 7.1 Hz, 1H, $H_{5'}$), 7.10 (d, J = 8.5 Hz, 1H, $H_{7'}$), 7.58 (dd, J = 8.6, 7.1 Hz, 1H, $H_{6'}$), 9.88 (s, 1H, NHCO). **^{13}C-NMR (75 MHz, Acetone-*d$_6$*)**: δ 23.4 (C4), 25.3 ($\underline{C}H_2CH_2CH_2CH_2NH$), 27.1 ($\underline{C}H_2CH_2CH_2NH$), 29.7 ($\underline{C}H_2CH_2NH$), 32.0 (C5), 34.0

($\underline{C}H_2CO$), 42.9 (CH_2NH), 49.8 (C3), 110.9 (C3a'), 111.2 (C5'), 117.6 (C7'), 133.7 (C7a'), 136.9 (C6'), 147.8 (C4'), 168.3 (C3'), 170.1 (C1'), 170.3 (C2), 172.6 (C6), 174.5 (CO). **HPLC (t_R, min):** 11.15. **MS (ESI, *m/z*, %):** 388.2 ($[M+H]^+$, 100).

(7*S*)-8,8-Dimethyl-2-oxo-7,8-dihydro-2*H*,6*H*-benzo[1,2-*b*:5,4-*b'*]dipyran-7-yl 6-{[2-(2,6-dioxopiperidin-3-yl)-1,3-dioxo-2,3-dihydro-1*H*-isoindol-4-yl]amino}hexanoate (2). Following general procedure C, compound **24** (30.0 mg, 84.0 μmol), DCC (16.0 mg, 77.0 μmol) and DMAP (3.80 mg, 31.0 μmol) were dissolved in anhydrous DCM (0.80 mL). Decursinol (**1**, synthesized as previously described[35]) (19.1 mg, 77.0 μmol) was then added. The residue was purified by column chromatography (DCM to DCM/MeOH, 98:2) to obtain compound **2** as a solid (30.0 mg, 49.0 μmol, 63% yield).

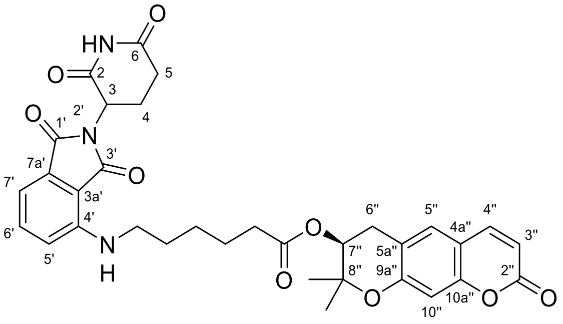

Mp: 112-114 °C. **R_f**: 0.78 (DCM/MeOH, 9:1). **IR (ATR, cm^{-1})**: ν 2925 (C-H), 1695 (C=O), 1624 (C=C), 1357 (C-H), 1134 (C-O). **^{1}H-NMR (300 MHz, $CDCl_3$)**: δ 1.34 (s, 3H, CH_3), 1.36 (s, 3H, CH_3), 1.37 – 1.44 (m, 2 H, $C\underline{H}_2CH_2CH_2NH$), 1.64 (m, 4H, $C\underline{H}_2CH_2C\underline{H}_2CH_2NH$), 2.12 (dt, *J* = 10.8, 3.4 Hz, 1H, H_4), 2.34 (t, *J* = 7.3 Hz, 2H, $C\underline{H}_2COO$), 2.67 – 2.98 (m, 4H, $1H_{6''}$, $1H_4$, $2H_5$), 3.08 – 3.29 (m, 3H, $1H_{6''}$, $C\underline{H}_2NH$), 4.79 – 4.99 (m, 1H, H_3), 5.05 (t, *J* = 4.7 Hz, 1H, $H_{7''}$), 6.20 (d, *J* = 9.5, 1H, $H_{3''}$), 6.78 (s, 1H, $H_{10''}$), 6.83 (d, *J* = 8.5 Hz, 1H, $H_{5'}$), 7.08 (d, *J* = 7.1 Hz, 1H, $H_{7'}$), 7.14 (s, 1H, $H_{5''}$), 7.48 (dd, *J* = 8.5, 7.1 Hz, 1H, $H_{6'}$), 7.55 (d, *J* = 9.5 Hz, 1H, $H_{4''}$), 8.17 (s, 1H, NHCO). **^{13}C-NMR (75 MHz, $CDCl_3$)**: δ 22.9 (C4), 23.4 ($\underline{C}H_2CH_2CH_2NH$), 24.7 ($\underline{C}H_2CH_2COO$), 24.9 ($CH_3$), 25.1 ($CH_3$), 27.9 (C6''), 29.0 ($\underline{C}H_2CH_2NH$), 31.5 (C5), 34.2 ($\underline{C}H_2CO$), 42.5 ($\underline{C}H_2NH$), 49.0 (C3), 70.3 (C7''), 76.6 (C8''), 104.9 (C10''), 110.1 (C3a'), 111.6 (C7'), 113.0 (C4a''), 113.5 (C3''), 115.7 (C5a''), 116.6 (C5'), 128.7 (C5''), 132.6 (C7a'), 136.3 (C6'), 143.2 (C4''), 147.0 (C4'), 154.3 (C10a''), 156.4 (C9a''), 161.3 (C2''), 167.7 (C1'), 168.5 (C2), 169.6 (C3'), 171.1 (C6), 172.9 ($CH_2\underline{C}OO$). **HPLC (t_R, min):** 13.49. **MS (ESI, *m/z*, %):** 616.3 ($[M+H]^+$, 100), 676.3 ($[M+H+{}^iPrOH]^+$, 100). HRMS (ESI) *m/z* calcd for $C_{33}H_{33}N_3O_9+Na^+$: 638.2114 $[M+Na]^+$; found, 638.2092.

2.3. Synthesis of PROTAC 3

Benzyl [2-(2-hydroxyethoxy)ethyl]carbamate (25). 2-(2-aminoethoxy)ethanol (1.0 eq, 1.9 mL, 19.0 mmol) was dissolved in dry DCM (20 mL), Et_3N (1.0 eq, 2.6 mL, 19.0 mmol) was added and the mixture was cooled to 0 °C. A solution of benzyl chloroformate (1.0 eq, 2.7 mL, 19.0 mmol) in dry DCM (20 mL) was slowly added and the reaction

mixture was stirred at 0 °C for 2 h and then at rt for 18 h. The reaction crude was washed with a saturated aqueous solution of $NaHCO_3$ (x3) and the aqueous layer was extracted with DCM (x3). The combined organic layers were washed with brine, dried over Na_2SO_4, filtered and evaporated. The residue was purified by flash chromatography (EtOAc) to obtain compound **25** as a colorless oil (2.70 g, 11.3 mmol, 60% yield).

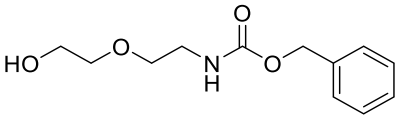

R_f: 0.42 (EtOAc). **IR (ATR, cm^{-1})**: ν 3341 (O-H), 3065 (=C-H), 2937 (C-H), 2872 (C-H), 1698 (C=O), 1249 (C-O), 1122 (C-O). **^{1}H-NMR (300 MHz, $CDCl_3$)**: δ 1.96 (s, br, 1H, OH), 3.36 – 3.45 (m, 2H, C$\underline{H}_2$NH), 3.53 – 3.58 (m, 4H, C$\underline{H}_2$OC$\underline{H}_2$), 3.68 – 3.77 (m, 2H, C$\underline{H}_2$OH), 5.11 (s, 2H, CH_{2Cbz}), 7.28 – 7.42 (m, 5H, $5CH_{Cbz}$). **^{13}C-NMR (75 MHz, $CDCl_3$)**: δ 41.0 (CH_2NH), 62.0 (CH_2OH), 66.9 (CH_{2Cbz}), 70.2 (CH_2O), 72.3 (CH_2O), 128.3 ($3CH_{Cbz}$), 128.7 ($2CH_{Cbz}$), 136.6 (C_{Cbz}), 156.6 (CO). **HPLC (t_R, min):** 10.58. **MS (ESI, *m/z*, %):** 196.2 ($[M+H-CH_2CH_2OH]^+$, 100). The spectroscopic data are consistent with those reported in the bibliography.[55]

***Tert*-butyl 2,2-dimethyl-4-oxo-3,8,11-trioxa-5-azatridecan-13-oate (26).** Following general procedure D, compound **25** (470 mg, 1.96 mmol) was dissolved in dry THF (5.0 mL) and was cooled to 0 °C. A mixture of potassium *tert*-butoxide (220 mg, 1.96 mmol) in dry THF (4.5 mL) was added. The reaction mixture was stirred for 30 min at 0 °C before *tert*-butyl bromoacetate (1.2 eq, 0.35 mL, 2.36 mmol) was added. The residue was purified by flash chromatography (hex/EtOAc, 2:1) to obtain compound **26** as a colorless oil (268 mg, 0.759 mmol, 39% yield).

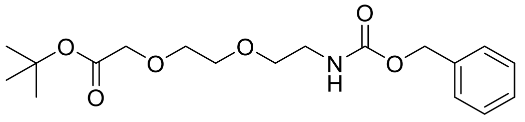

R_f: 0.36 (Hex/EtOAc, 1:1). **IR (ATR, cm^{-1})**: ν 2976 (C-H), 2932 (C-H), 1744 (C=O), 1717 (C=O), 1228 (C-O), 1142 (C-O), 1116 (C-O). **^{1}H-NMR (300 MHz, $CDCl_3$)**: δ 1.45 (s, 9H, $(CH_3)_3$), 3.39 (q, J = 5.3 Hz, 2H, C$\underline{H}_2$NH), 3.57 (t, J = 5.0 Hz, 2H, C$\underline{H}_2$OCH_2COO), 3.59 – 3.74 (m, 4H, C$\underline{H}_2$OC$\underline{H}_2$$CH_2$NH), 3.98 (s, 2H, CH_2COO), 5.09 (s, 2H, CH_{2Cbz}), 5.38 – 5.44 (m, 1H, NH), 7.17 – 7.41 (m, 5H, $5CH_{Cbz}$). **^{13}C-NMR (75 MHz, $CDCl_3$)**: δ 28.2 ($(CH_3)_3$), 41.0 (CH_2NH), 66.7 (CH_{2Cbz}), 69.0 (CH_2CO), 70.2 ($\underline{C}H_2$OCH_2COO), 70.3 (CH_2O$CH_2$$\underline{C}H_2$NH), 70.8 ($\underline{C}H_2OCH_2$$CH_2$NH), 81.8 ($\underline{C}(CH_3)_3$), 128.1 (2C), 128.3, 128.5 (2C) ($5CH_{Cbz}$), 136.7 (C_{Cbz}), 156.6 (NHCOO), 169.7 (COO). **HPLC (t_R, min):** 13.19. The spectroscopic data are consistent with those reported in the bibliography.[55]

***Tert*-butyl [2-(2-aminoethoxy)ethoxy]acetate (16).** Following general procedure F, protected amine **26** (100 mg, 0.283 mmol) was dissolved in EtOH and hydrogenated in a

Thales Nano H-Cube® flux reactor. The solvent was evaporated under reduced pressure to obtain compound **16** as a colorless oil (57.0 mg, 0.261 mmol, 92% yield).

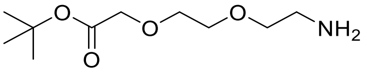

R$_f$: 0.1 (DCM/MeOH, 9:1). **IR (ATR, cm^{-1})**: ν 3338 (N-H), 2977 (C-H), 2873 (C-H), 1745 (C=O), 1669 (C=O), 1118 (C-O). **^{1}H-NMR (300 MHz, CDCl$_3$)**: δ 1.44 (s, 9H, (C<u>H</u>$_3$)$_3$), 2.11 (s, 2H, N<u>H</u>$_2$), 2.84 (t, *J* = 5.2 Hz, 1H, C<u>H</u>$_2$NH$_2$), 3.49 (t, *J* = 5.2 Hz, 2H, C<u>H</u>$_2$CH$_2$NH$_2$), 3.56 – 3.82 (m, 4H, OC<u>H</u>$_2$C<u>H</u>$_2$O), 3.98 (s, 2H, C<u>H</u>$_2$COO). **^{13}C-NMR (75 MHz, CDCl$_3$)**: δ 28.2 ((CH$_3$)$_3$), 41.7 (<u>C</u>H$_2$CH$_2$NH$_2$), 69.1 (<u>C</u>H$_2$COO), 70.4 (<u>C</u>H$_2$OCH$_2$CO), 70.8 (<u>C</u>H$_2$CH$_2$OCH$_2$COO), 73.3 (<u>C</u>H$_2$NH$_2$), 81.7 (<u>C</u>(CH$_3$)$_3$), 169.7 (COO).

***Tert*-butyl [2-(2-{[2-(2,6-dioxopiperidin-3-yl)-1,3-dioxo-2,3-dihydro-1*H*-isoindol-4-yl]amino}ethoxy)ethoxy]acetate (27).** Following general procedure A, 4-fluoro-thalidomide (150 mg, 0.541 mmol) was dissolved in DMF (2.7 mL) and DIPEA (0.19 mL, 1.09 mmol) and compound **16** (143 mg, 0.647 mmol) were added. The resulting residue was purified by flash chromatography (DCM/MeOH, 99:1) to obtain compound **27** as a solid (144 mg, 0.303 mmol, 56% yield).

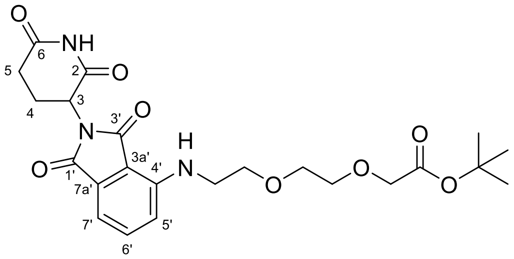

Mp: 70-72 °C. **R$_f$**: 0.25 (hex/EtOAc, 1:1). **IR (ATR, cm^{-1})**: ν 3395 (N-H), 2977 (C-H), 2909 (C-H), 1695 (C=O), 1114 (C-O). **^{1}H-NMR (300 MHz, CDCl$_3$)**: δ 1.47 (s, 9H, (CH$_3$)$_3$), 2.02 – 2.20 (m, 1H, H$_4$), 2.55 – 3.03 (m, 3H, H$_4$, H$_5$), 3.49 (t, *J* = 5.5 Hz, 2H, C<u>H</u>$_2$NH), 3.64 – 3.83 (m, 6H, 3CH$_2$O), 4.02 (s, 2H, C<u>H</u>$_2$COO), 4.91 (dd, *J* = 12.1, 5.3 Hz, 1H, H$_3$), 6.93 (dd, *J* = 8.6, 0.6 Hz, 1H, H$_{5'}$), 7.10 (dd, *J* = 7.1, 0.7 Hz, 1H, H$_{7'}$), 7.49 (dd, *J* = 8.6, 7.1 Hz, 1H, H$_{6'}$), 8.04 (s, 1H, NHCO). **^{13}C-NMR (75 MHz, CDCl$_3$)**: δ 22.9 (C4), 28.2 ((CH$_3$)$_3$), 31.5 (C5), 42.5 (CH$_2$NH), 49.0 (C3), 69.2 (<u>C</u>H$_2$COO), 69.7 (NHCH$_2$<u>C</u>H$_2$), 70.8 (CH$_2$O), 71.0 (CH$_2$O), 81.7 (<u>C</u>(CH$_3$)$_3$), 110.4 (C3a'), 111.8 (C7'), 117.0 (C5'), 132.6 (C7a'), 136.2 (C6'), 147.0 (C4'), 167.7 (C1'), 168.4 (C3'), 169.4 (COO), 169.8 (C2), 171.1 (C6). **HPLC (t$_R$, min):** 12.52. **MS (ESI, *m/z*, %):** 420.0 ([M+H-^{t}Bu]$^+$, 100). The spectroscopic data are consistent with those reported in the bibliography.[55]

[2-(2-{[2-(2,6-Dioxopiperidin-3-yl)-1,3-dioxo-2,3-dihydro-1*H*-isoindol-4-yl]amino}-ethoxy)ethoxy]acetic acid (28). Following general procedure B, *tert*-butyl ester **27** (30.0

mg, 63.0 μmol) was dissolved in DCM (0.63 mL) and TFA (0.48 mL, 6.31 mmol) was added. Compound **28** was obtained as a solid (25.0 mg, 60.0 μmol, 95% yield).

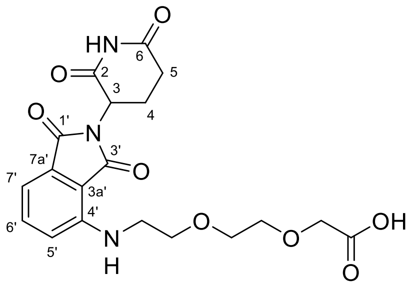

Mp: 86-88 °C. **Rf**: 0.38 (DCM/MeOH, 9:1). **IR (ATR, cm^{-1})**: ν 3384 (N-H), 3219 (O-H), 3115 (=C-H), 2911 (C-H), 1693 (C=O), 1350 (C-O), 1141 (C-O). **^{1}H-NMR (300 MHz, Acetone-*d$_6$*)**: δ 2.12 – 2.28 (m, 1H, H_4), 2.67 – 3.05 (m, 3H, H_4, H_5), 3.55 (t, *J* = 5.4 Hz, 2H, C<u>H</u>$_2$NH), 3.61 – 3.82 (m, 6H, 3CH_2O), 4.13 (s, 2H, C<u>H</u>$_2$COOH), 5.06 (dd, *J* = 12.5, 5.4 Hz, 1H, H_3), 7.04 (dd, *J* = 7.1, 0.6 Hz, 1H, $H_{5'}$), 7.14 (dd, *J* = 8.5, 0.6 Hz, 1H, $H_{7'}$), 7.58 (dd, *J* = 8.6, 7.1 Hz, 1H, $H_{6'}$). **^{13}C-NMR (75 MHz, Acetone-*d$_6$*)**: δ 23.4 (C4), 32.0 (C5), 42.9 (CH_2NH), 49.8 (C3), 68.6 (<u>C</u>H_2COOH), 70.2 (NHCH_2<u>C</u>H_2O), 71.0, 71.4 (2CH_2O), 111.1 (C3a’), 111.2 (C5’), 117.8 (C7’), 133.6 (C7a’), 147.7 (C4’), 168.2 (C3’), 170.1 (C1’), 170.2 (C2), 171.7 (COO), 172.6 (C6). **HPLC (t_R, min):** 10.24.

(7*S*)-8,8-Dimethyl-2-oxo-7,8-dihydro-2*H*,6*H*-benzo[1,2-*b*:5,4-*b'*]dipyran-7-yl [2-(2-{[2-(2,6-dioxopiperidin-3-yl)-1,3-dioxo-2,3-dihydro-1*H*-isoindol-4-yl]amino}ethoxy)-ethoxy]acetate (3). Following general procedure C, compound **28** (17.0 mg, 41.0 μmol), DCC (8.40 mg, 41.0 μmol) and DMAP (1.00 mg, 8.00 μmol) were dissolved in anhydrous DCM (0.30 mL). Decursinol (**1**) (5.00 mg, 20.0 μmol) was then added. The residue was purified by column chromatography (DCM to DCM/MeOH, 98:2) to obtain compound **3** as a solid (8.10 mg, 13.0 mmol, 62% yield).

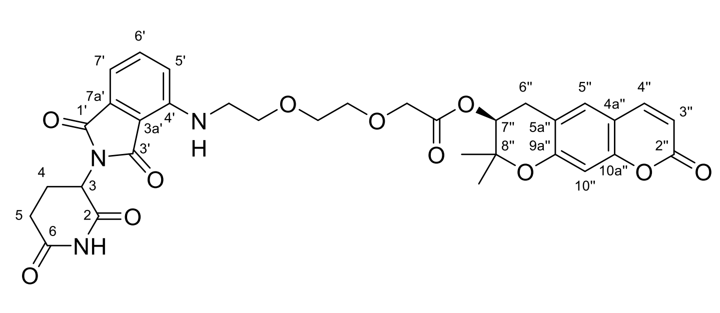

Mp: 103-105 °C. **R_f**: 0.62 (EtOAc). **IR (ATR, cm^{-1})**: ν 2923 (C-H), 1695 (C=O), 1624 (C=C), 1357 (C-H), 1134 (C-O). **^{1}H-NMR (300 MHz, $CDCl_3$)**: δ 1.34 (s, 3H, CH_3), 1.37 (s, 3H, CH_3), 2.04 – 2.19 (m, 1H, 1H_4), 2.69 – 2.94 (m, 4H, 1H_4, 2H_5, 1$H_{6''}$), 3.18 (ddd, *J* = 17.3, 4.8, 1.2 Hz, 1H, 1$H_{6''}$), 3.42 (m, 2H, C<u>H</u>$_2$NH), 3.58 – 3.77 (m, 6H, 3CH_2O), 4.05 – 4.26 (m, 2H, C<u>H</u>$_2$COO), 4.90 (m, 1H, H_3), 5.13 (t, *J* = 4.6 Hz, 1H, $H_{7''}$), 6.19 (d, *J* = 9.5, 1H, $H_{3''}$), 6.42 (s, 1H, CH_2N<u>H</u>), 6.75 (s, 1H, $H_{10''}$), 6.87 (d, *J* = 8.5 Hz, 1H, $H_{5'}$), 7.06 (dd, *J* = 7.0, 3.4 Hz, 1H, $H_{7'}$), 7.13 (d, *J* = 1.5 Hz, 1H, $H_{5''}$), 7.46 (dd, *J* = 8.4, 7.1

Hz, 1H, $H_{6'}$), 7.53 (d, J = 9.4, 1H, $H_{4''}$), 8.24 (s, 1H, N<u>H</u>CO). **^{13}C-NMR (75 MHz, $CDCl_3$):** δ 22.8 (C4), 23.5 (CH_3), 24.9 (CH_3), 27.82 (C6''), 31. (C5), 42.4 (CH_2NH), 48.9 (C3), 68.7 (CH_2COO), 69.5 ($NHCH_2\underline{C}H_2$), 70.5 (CH_2O), 70.9 (C7''), 71.2 (CH_2O), 76.4 (C8''), 104.80 (C10''), 110. (C3a'), 111.7 (C7'), 113.1 (C4a''), 113.5 (C3''), 115.4 (C5a''), 116.8 (C5'), 128.8 (C5''), 132.6 (C7a'), 136.2 (C6'), 143.2 (C4''), 146.9 (C4'), 154.3 (C10a''), 156.3 (C9a''), 161.3 (C2''), 167.7 (C1'), 168.5 (C2), 169.3 (C3'), 170.1 (C6), 171.2 (COO). **HPLC (t_R, min):** 12.47. **MS (ESI, *m/z*, %):** 648.3 ($[M+H]^+$, 50). HRMS (ESI) *m/z* calcd for $C_{33}H_{33}N_3O_{11}+Na^+$: 670.2013 $[M+Na]^+$; found, 670.1986.

2.4. Synthesis of PROTAC 4

Ethyl 3-oxo-1-phenyl-2,7,10-trioxa-4-azadodecan-12-oate (29). Following general procedure E, compound **25** (1.50 g, 6.27 mmol) was dissolved in dry toluene (8.0 mL) and the mixture was cooled to 0 ºC. A mixture of potassium *tert*-butoxide (1.05 g, 9.50 mmol) in *tert*-butanol (9.0 mL) was then added. The reaction mixture was stirred for 30 min at 0 ºC before ethyl bromoacetate (1.4 mL, 12.5 mmol) was added. The residue was purified by flash chromatography (hex/EtOAc, 1:1 to EtOAc) to obtain compound **29** as a colorless oil (820 mg, 2.52 mmol, 40% yield).

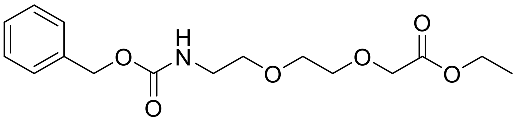

R_f: 0.27 (Hex/EtOAc, 1:1). **IR (ATR, cm^{-1})**: ν 2938 (C-H), 1726 (C=O), 1584 (N-H), 1249 (C-O), 1151 (C-O). **^{1}H-NMR (300 MHz, $CDCl_3$)**: δ 1.26 (t, J = 7.2 Hz, 3H, C<u>H</u>$_3$), 3.40 (q, J = 5.3 Hz, 2H, C<u>H</u>$_2$NH), 3.57 (t, J = 5.1 Hz, 2H, C<u>H</u>$_2$CH$_2$NH), 3.60 – 3.75 (m, 4H, $2CH_2O$), 4.12 (s, 2H, C<u>H</u>$_2$COOEt), 4.20 (q, J = 7.1 Hz, 2H, OC<u>H</u>$_2$CH$_3$), 5.10 (s, 2H, CH_{2Cbz}), 5.32 (s, 1H, NHCOO), 7.27 – 7.41 (m, 5H, $5CH_{Cbz}$). **^{13}C-NMR (75 MHz, $CDCl_3$):** δ 14.3 (CH_3), 41.0 (CH_2NH), 61.0 (O<u>C</u>H$_2$CH$_3$), 66.8 (CH_{2Cbz}), 68.8 (<u>C</u>H$_2$COOEt), 70.3, 70.5, 71.0 ($3CH_2O$), 128.2 (3C, $3CH_{Cbz}$), 128.6 (2C, $2CH_{Cbz}$), 136.8 (C_{Cbz}), 151.5 (NHCOO), 170.6 (COO). **HPLC (t_R, min):** 12.17. **MS (ESI, *m/z*, %):** 326.2 ($[M+H]^+$, 100). The spectroscopic data are consistent with those reported in the bibliography.[55]

Benzyl {2-[2-(2-hydroxyethoxy)ethoxy]ethyl}carbamate (39). Finely powdered $NaBH_4$ (6.5 eq, 604 mg, 15.9 mmol) was added over a period of 15 min to a stirred solution of compound **40** (1.0 eq, 800 mg, 2.16 mmol) in THF (12 mL) at reflux. Subsequently, MeOH (12 mL) was carefully added dropwise to the stirred suspension for over 30 min. The resulting mixture was refluxed for a further 60 min. After this time, the colorless solution was allowed to cool to rt before the reaction was quenched with a saturated aqueous solution of NH_4Cl (12 mL). The organic layer was separated, and the aqueous phase was extracted with EtOAc (x2). The combined organic layers were washed with brine, dried over Na_2SO_4, filtered and the solvent was removed under reduced

pressure. Purification by flash chromatography (hex/EtOAc 1:2) yielded compound **39** as a colorless oil (650 mg, 2.29 mmol, 93%).

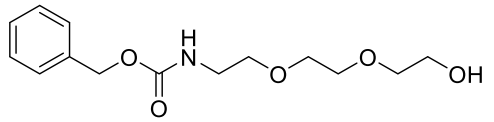

R

R_f: 0.28 (EtOAc). **IR (ATR, cm^{-1})**: ν 3340 (O-H), 3066 (=C-H), 2873 (C-H), 1700 (C=O), 1532 (N-H), 1250 (C-O), 1102 (C-O). **^{1}H-NMR (300 MHz, $CDCl_3$)**: δ 3.39 (q, *J* = 5.4 Hz, 2H, C<u>H</u>$_2$NH), 3.52 – 3.66 (m, 8H, 4CH_2O), 3.66 – 3.76 (m, 2H, C<u>H</u>$_2$OH), 5.10 (s, 2H, CH_{2Cbz}), 5.43 (s, 1H, NH), 7.27 – 7.41 (m, 5H, 5CH_{Cbz}). **^{13}C-NMR (75 MHz, $CDCl_3$):** δ 41.0 (CH_2NH), 61.8 (CH_2OH), 66.8 (CH_{2Cbz}), 70.2, 70.5, 70.5, 72.6 (4CH_2O), 128.2 (3C, 3CH_{Cbz}), 128.6 (2C, 2CH_{Cbz}), 136.7 (C_{Cbz}), 156.6 (CO). **HPLC (t_R, min):** 10.59. **MS (ESI, *m/z*, %):** 284.0 ([M+H]$^+$, 100). The spectroscopic data are consistent with those reported in the bibliography.[55]

***Tert*-butyl 3-oxo-1-phenyl-2,7,10,13-tetraoxa-4-azapentadecan-15-oate (31).** Following general procedure E, compound **30** (650 mg, 2.29 mmol) was dissolved in dry toluene (6.0 mL) and the mixture was cooled to 0 ºC. A mixture of potassium *tert*-butoxide (386 mg, 3.44 mmol) in *tert*-butanol (3.2 mL) was then added. The reaction mixture was stirred for 30 min at 0 ºC before *tert*-butyl bromoacetate (0.68 mL, 4.59 mmol) was added. The residue was purified by flash chromatography (hex/EtOAc, 1:1 to EtOAc) to obtain compound **31** as a colorless oil (270 mg, 0.679 mmol, 30% yield).

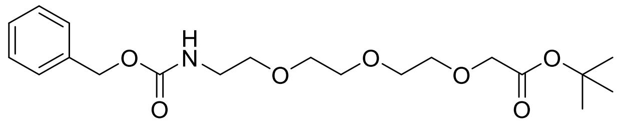

R_f: 0.24 (Hex/EtOAc, 1:1). **IR (ATR, cm^{-1})**: ν 2932 (C-H), 1744 (C=O), 1720 (C=O), 1527 (N-H), 1248 (C-O), 1142 (C-O). **^{1}H-NMR (300 MHz, $CDCl_3$):** δ 1.46 (s, 9H, $(CH_3)_3$), 3.39 (q, *J* = 5.3 Hz, 2H, H_2), 3.56 (t, *J* = 5.0 Hz, 2H, NHCH$_2$C<u>H</u>$_2$O), 3.60 – 3.72 (m, 8H, 4CH_2O), 3.98 (s, 2H, CH_2COO), 5.10 (s, 2H, CH_{2Cbz}), 5.38 (s, 1H, NH), 7.27 – 7.46 (m, 5H, 5CH_{Cbz}). **^{13}C-NMR (75 MHz, $CDCl_3$):** δ 28.2 ($(CH_3)_3$), 41.0 (CH_2NH), 66.8 (CH_{2Cbz}), 69.1 (<u>C</u>H$_2$COO), 70.2, 70.4, 70.7 (2C), 70.8 (5CH_2O), 81.7 (<u>C</u>$(CH_3)_3$), 128.2 (2C, 2CH_{Cbz}), 128.2 (CH_{Cbz}), 128.6 (2C, 2CH_{Cbz}), 136.7 (C_{Cbz}), 156.5 (NHCOO), 169.8 (COO). **HPLC (t_R, min):** 13.09. **MS (ESI, *m/z*, %):** 458.1 ([M+H+^{i}PrOH]$^+$, 100). The spectroscopic data are consistent with those reported in the bibliography.[55]

***Tert*-butyl {2-[2-(2-aminoethoxy)ethoxy]ethoxy}acetate (17).** Following general procedure F, protected amine **31** (270 mg, 0.704 mmol) was dissolved in EtOH and was run through a Thales Nano H-Cube® flux reactor. The solvent was evaporated under reduced pressure to obtain compound **17** as a colorless oil (106 mg, 0.403 mmol, 57% yield).

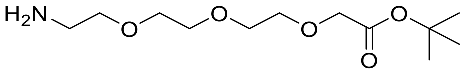

R$_f$: 0.28 (EtOAc). **IR (ATR, cm^{-1})**: ν 3339 (N-H), 2975 (C-H), 2930 (C-H), 1725 (C=O), 1146 (C-O). **^{1}H-NMR (300 MHz, CDCl$_3$):** δ 1.47 (s, 9H, (CH$_3$)$_3$), 2.86 (t, *J* = 4.9 Hz, 2H, C$\underline{H}_2$NH$_2$), 3.51 (t, *J* = 4.9 Hz, 2H, NH$_2$CH$_2$C$\underline{H}_2$O), 3.56 – 3.77 (m, 8H, 4CH$_2$O), 4.02 (s, 2H, OCH$_2$COO). **^{13}C-NMR (75 MHz, CDCl$_3$):** δ 28.2 ((CH$_3$)$_3$), 41.9 (CH$_2$NH$_2$), 69.2 ($\underline{C}$H$_2$COO), 70.4, 70.7, 70.8, 70.9 (4CH$_2$O), 73.6 (NH$_2$CH$_2$C$\underline{H}_2$O), 81.7 ($\underline{C}$(CH$_3$)$_3$), 169.8 (COO).

***Tert*-butyl {2-[2-(2-{[2-(2,6-dioxopiperidin-3-yl)-1,3-dioxo-2,3-dihydro-1*H*-isoindol-4-yl]amino}ethoxy)ethoxy]ethoxy}acetate (32).** Following general procedure A, 4-fluoro-thalidomide (95.0 mg, 0.344 mmol) was dissolved in DMF (1.7 mL) and DIPEA (0.12 mL, 0.688 mmol) and compound **17** (108 mg, 0.413 mmol) were added. The resulting residue was purified by flash chromatography (DCM/MeOH, 99:1) to obtain compound **32** (80.0 mg, 0.154 mmol, 45% yield).

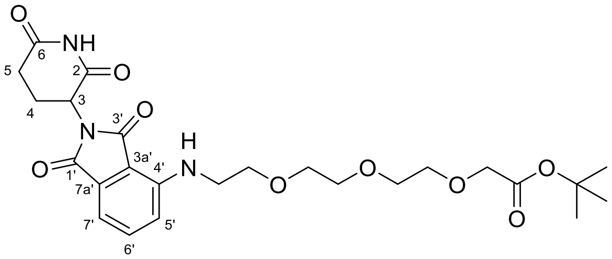

R$_f$: 0.73 (EtOAc). **IR (ATR, cm^{-1}):** ν 3395 (N-H), 2921 (C-H), 1694 (C=O), 1141 (C-O), 1111 (C-O). **^{1}H-NMR (300 MHz, CDCl$_3$):** δ 1.47 (s, 9H, (CH$_3$)$_3$), 2.06 – 2.20 (m, 1H, 1H$_4$), 2.65 – 2.94 (m, 3H, 1H$_4$, 2H$_5$), 3.47 (q, *J* = 5.5 Hz, 2H, CH$_2$NH), 3.64 – 3.75 (m, 10H, 5CH$_2$O), 4.01 (s, 2H, CH$_2$COO), 4.84 – 5.01 (m, 1H, H$_3$), 6.49 (t, *J* = 5.7 Hz, 1H, NHCH$_2$), 6.92 (d, *J* = 8.5 Hz, 1H, H$_{5'}$), 7.10 (dd, *J* = 7.1, 0.6 Hz, 1H, H$_{7'}$), 7.49 (dd, *J* = 8.5, 7.2 Hz, 1H, H$_{6'}$), 8.15 (s, 1H, NHCO). **^{13}C-NMR (75 MHz, CDCl$_3$):** δ 22.9 (C4), 28.2 ((CH$_3$)$_3$), 31.6 (C5), 42.6 (CH$_2$NH), 49.0 (C3), 69.2 (CH$_2$COO), 69.2, 69.6, 70.8 (2C), 70.9 (5CH$_2$O), 81.8 (C(CH$_3$)$_3$), 110.5 (C3a'), 111.8 (C7'), 117.0 (C5'), 132.7 (C7a'), 136.2 (C6'), 147.0 (C4'), 167.7 (C1'), 168.4 (C2), 169.3 (C3'), 169.9 (C6), 171.1 (COO). **HPLC (t$_R$, min):** 12.45. **MS (ESI, *m/z*, %):** 464.23 ([M+H-^{t}Bu]$^+$, 100), 520.3 ([M+H]$^+$, 40). The spectroscopic data are consistent with those reported in the bibliography.[55]

{2-[2-(2-{[2-(2,6-Dioxopiperidin-3-yl)-1,3-dioxo-2,3-dihydro-1*H*-isoindol-4-yl]amino}ethoxy)ethoxy]ethoxy}acetic acid (33). Following general procedure B, *tert*-butyl ester **32** (50.0 mg, 96.9 μmol) was dissolved in DCM (0.48 mL) and TFA (0.74 mL, 9.62 mmol) was added. Compound **33** was obtained as a solid (40.0 mg, 86.0 μmol, 90% yield).

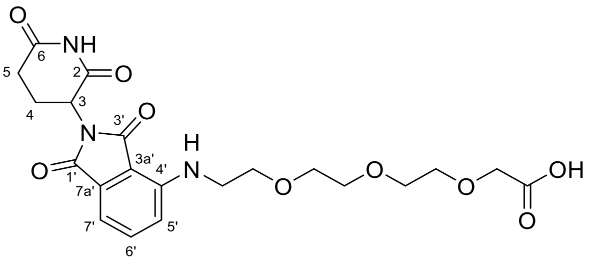

Mp: 102-104 °C. **R$_f$**: 0.23 (DCM/MeOH, 9:1). **IR (ATR, cm^{-1})**: ν 3387 (N-H), 3219 (O-H), 3104 (=C-H), 2914 (C-H), 1754 (C=O), 1694 (C=O), 1143 (C-O). **^{1}H-NMR (300 MHz, CDCl$_3$):** δ 2.05 – 2.21 (m, 1H, H_4), 2.62 – 3.01 (m, 3H, H_4, H_5), 3.48 (t, *J* = 5.2 Hz, 2H, C$\underline{H}_2$NH), 3.63 – 3.79 (m, 10H, 5CH$_2$O), 4.14 (s, 2H, CH$_2$COO), 4.86 – 4.98 (m, 1H, H_3), 6.92 (d, *J* = 8.6 Hz, 1H, $H_{5'}$), 7.10 (d, *J* = 7.1 Hz, 1H, $H_{7'}$), 7.49 (dd, *J* = 8.5, 7.1 Hz, 1H, $H_{6'}$), 8.42 (s, 1H, NHCO). **^{13}C-NMR (75 MHz, CDCl$_3$):** δ 22.9 (C4), 31.5 (C5), 42.5 (CH$_2$NH), 49.0 (C3), 68.9 ($\underline{C}$H$_2$COO), 69.6, 70.4, 70.6, 70.7, 71.6 (5CH$_2$O), 110.4 (C3a’), 111.9 (C7’), 117.0 (C5’), 132.6 (C7a’), 136.2 (C6’), 147.0 (C4’), 167.8 (C1’), 168.9 (C3’), 169.5 (C2), 171.6 (C6), 172.3 (COO). **HPLC (t$_R$, min):** 9.82. **MS (ESI, *m/z*, %):** 464.2 ([M+H]$^+$, 100).

(7*S*)-8,8-Dimethyl-2-oxo-7,8-dihydro-2*H*,6*H*-benzo[1,2-*b*:5,4-*b′*]dipyran-7-yl {2-[2-(2-{[2-(2,6-dioxopiperidin-3-yl)-1,3-dioxo-2,3-dihydro-1*H*-isoindol-4-yl]amino}ethoxy)-ethoxy]ethoxy}acetate (4). Following general procedure C, compound **33** (35.2 mg, 61.0 µmol), DCC (12.6 mg, 61.0 µmol) and DMAP (2.00 mg, 16.0 µmol) were dissolved in anhydrous DCM (0.20 mL). Decursinol (**1**) (10.0 mg, 41.0 µmol) was then added. The residue was purified by column chromatography (DCM to DCM/MeOH, 98:2) to obtain compound **4** as a solid (15.0 mg, 22.0 µmol, 53% yield).

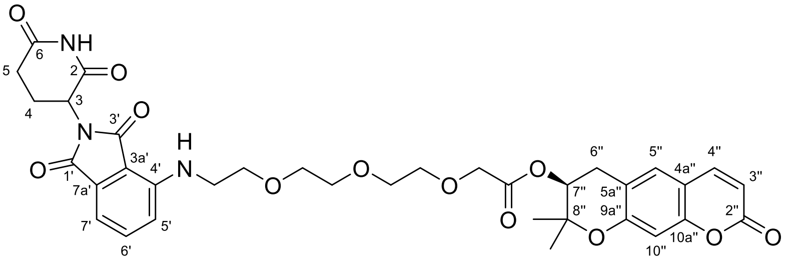

Mp: 83-85 °C. **R$_f$**: 0.59 (DCM/MeOH, 9:1). **IR (ATR, cm^{-1})**: ν 2922 (C-H), 1696 (C=O), 1625 (C=C), 1358 (C-H), 1195, 1134 (C-O). **^{1}H-NMR (300 MHz, CDCl$_3$):** δ 1.34 (s, 3H, CH$_3$), 1.37 (s, 3H, CH$_3$), 2.06 – 2.20 (m, 1H, 1H_4), 2.65 – 2.94 (m, 4H, 1H_4, H_5, 1$H_{6''}$), 3.19 (dd, *J* = 17.5, 4.7 Hz, 1H, 1$H_{6''}$), 3.43 (q, *J* = 5.4 Hz, 2H, C$\underline{H}_2$NH), 3.59 – 3.73 (m, 10H, 5CH$_2$O), 4.05 – 4.31 (m, 2H, CH$_2$COO), 4.85 – 4.97 (m, 1H, H_3), 5.14 (t, *J* = 4.6 Hz, 1H, $H_{7''}$), 6.20 (d, *J* = 9.5 Hz, 1H, $H_{3''}$), 6.45 (t, *J* = 5.7 Hz, 1H, N$\underline{H}$CH$_2$), 6.77 (s, 1H, $H_{10''}$), 6.89 (d, *J* = 8.5 Hz, 1H, $H_{5'}$), 7.08 (dd, *J* = 7.1, 2.2 Hz, 1H, $H_{7'}$), 7.14 (s, 1H, $H_{5''}$), 7.42 – 7.53 (m, 1H, $H_{6'}$), 7.55 (d, *J* = 9.4, 1H, $H_{4''}$), 8.19 (s, 1H, NHCO). **^{13}C-NMR (75 MHz, CDCl$_3$):** δ 22.9 (C4), 23.5 (CH$_3$), 25.0 (CH$_3$), 27.9 (C6’’), 31.5 (C5), 42.5 (CH$_2$NH), 49.0 (C3), 68.6 ($\underline{C}$H$_2$COO), 69.6, 70.7, 70.8 (3C), 71.0 (C7’’, 5CH$_2$O), 76.5

(C8''), 104.9 (C10''), 110.4 (C3a'), 111.8 (C7'), 113.1 (C4a''), 113.6 (C3''), 115.4 (C5a''), 116.9 (C5'), 128.8 (C5''), 132.6 (C7a'), 136.2 (C6'), 143.2 (C4''), 146.9 (C4'), 154.3 (C10a''), 156.3 (C9a''), 161.3 (C2''), 167.7 (C1'), 168.5 (C2), 169.3 (C3'), 170.3 (C6), 171.1 (COO). **HPLC (t_R, min):** 10.83. **MS (ESI, *m/z*, %):** 692.2 ($[M+H]^+$, 100). HRMS (ESI) *m/z* calcd for $C_{35}H_{37}N_3O_{12}+Na^+$: 714.2275 $[M+Na]^+$; found, 714.2256.

2.5. Synthesis of PROTAC 5

***Tert*-butyl 6-{[2-(2,6-dioxopiperidin-3-yl)-1-oxo-2,3-dihydro-1*H*-isoindol-4-yl]-amino}hexanoate (34).** Following general procedure A, lenalidomide (200 mg, 0.771 mmol) was dissolved in DMF (4.0 mL) and DIPEA (0.40 mL, 2.31 mmol) and *tert*-butyl 6-bromohexanoate **18**, synthesized as previously reported[53] (232 mg, 0.928 mmol) were added. The crude was purified by flash chromatography (DCM to DCM/MeOH, 98:2) to obtain compound **34** as a pale white solid (100 mg, 0.231 mmol, 30% yield).

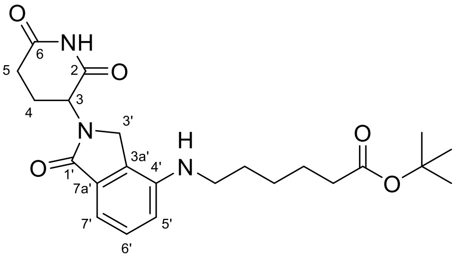

Mp: 213-215 ºC. **R$_f$**: 0.69 (DCM/MeOH, 9:1). **IR (ATR, cm^{-1})**: ν 3072 (=C-H), 2974 (C-H), 2928 (C-H), 1709 (C=O), 1663 (C=O), 1609 (C=O), 1150 (C-O). **^{1}H-NMR (300 MHz, $CDCl_3$)**: δ 1.43 (s, 9H, $(CH_3)_3$), 1.45 (m, 3H, $C\underline{H}_2CH_2CH_2N\underline{H}$), 1.53 – 1.75 (m, 4H, $C\underline{H}_2CH_2C\underline{H}_2CH_2NH$), 2.08 – 2.18 (m, 2H, H_4), 2.23 (t, J = 7.3 Hz, 2H, CH_2COO), 2.74 – 2.86 (m, 2H, H_5), 3.21 (t, J = 7.0 Hz, 2H, $C\underline{H}_2NH$), 4.13 (d, J = 15.7 Hz, 1H, $H_{3'}$), 4.28 (d, J = 15.7 Hz, 1H, $H_{3'}$), 5.19 (dd, J = 13.1, 5.3 Hz, 1H, H_3), 6.79 (dd, J = 8.0, 0.9 Hz, 1H, $H_{5'}$), 7.24 (dd, J = 7.5, 0.9 Hz, 1H, $H_{7'}$), 7.34 (t, J = 7.7 Hz, 1H, $H_{6'}$), 8.47 (s, 1H, NHCO). **^{13}C-NMR (75 MHz, $CDCl_3$)**: δ 23.5 (C4), 24.7 ($\underline{C}H_2CH_2COO$), 26.5 ($C\underline{H}_2CH_2CH_2NH$), 28.2 ($(CH_3)_3$), 29.0 ($\underline{C}H_2CH_2NH$), 31.6 (C5), 35.4 ($\underline{C}H_2COO$), 43.7 ($CH_2NH$), 45.2 (C3'), 51.9 (C3), 80.3 ($\underline{C}(CH_3)_3$), 112.9 (C7'), 113.5 (C5'), 126.6 (C3a'), 129.8 (C6'), 132.0 (C7a'), 143.0 (C4'), 170.0 (C1'), 170.1 (C2), 171.6 (C6), 173.2 (COO). **HPLC (t_R, min):** 13.10. **MS (ESI, *m/z*, %):** 374.2 ($[M+H-{}^tBu]^+$, 100).

6-{[2-(2,6-Dioxopiperidin-3-yl)-1-oxo-2,3-dihydro-1*H*-isoindol-4-yl]amino}hexanoic acid (35). Following general procedure B, *tert*-butyl ester **34** (80.0 mg, 0.190 mmol) was dissolved in DCM (1.9 mL) and TFA (1.4 mL, 18.6 mmol) was added. Compound **35** was obtained as a solid (32.6 mg, 87.0 µmol, 46% yield).

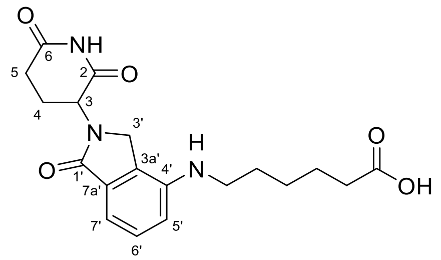

Mp: 202-204 ºC. **R**$_f$: 0.44 (DCM/MeOH, 9:1). **IR (ATR, cm^{-1})**: ν 3069 (=C-H), 2974 (C-H), 2928 (C-H), 1709 (C=O), 1663 (C=O), 1609 (C=O), 1150 (C-O). **^{1}H-NMR (300 MHz, DMSO)**: δ 1.30 – 1.42 (m, 2H, C$\underline{H}_2$CH$_2$CH$_2$NH), 1.45 – 1.66 (m, 4H, C$\underline{H}_2$CH$_2$C$\underline{H}_2$CH$_2$NH), 1.96 – 2.11 (m, 1H, H$_4$), 2.22 (t, *J* = 7.3 Hz, 2H, C$\underline{H}_2$COO), 2.25 – 2.37 (m, 1H, H$_4$), 2.53 – 2.67 (m, 1H, H$_5$), 2.83 – 3.01 (m, 1H, 1H$_5$), 3.11 (t, *J* = 7.0 Hz, 2H, C$\underline{H}_2$NH), 4.12 (d, *J* = 17.2 Hz, 1H, 1H$_{3'}$), 4.23 (d, *J* = 17.2 Hz, 1H, 1H$_{3'}$), 5.11 (dd, *J* = 13.2, 5.1 Hz, 1H, H$_3$), 6.74 (d, *J* = 8.0 Hz, 1H, H$_{5'}$), 6.93 (d, *J* = 7.4 Hz, 1H, H$_{7'}$), 7.28 (t, *J* = 7.7 Hz, 1H, H$_{6'}$), 11.00 (s, 1H, NHCO). **^{13}C-NMR (75 MHz, DMSO)**: δ 22.8 (C4), 24.4 ($\underline{C}$H$_2$CH$_2$CH$_2$CH$_2$NH), 26.2 ($\underline{C}$H$_2$CH$_2$CH$_2$NH), 28.3 ($\underline{C}$H$_2$CH$_2$NH), 31.3 (C5), 33.7 ($\underline{C}$H$_2$COO), 42.7 (CH$_2$NH), 45.7 (C3'), 51.5 (C3), 110.0 (C7'), 111.8 (C5'), 126.5 (C3a'), 129.25 (C6'), 132.1 (C7a'), 143.7 (C4'), 168.9 (C1'), 171.3 (C2), 172.9 (C6), 174.5 (COO). **HPLC (t$_R$, min):** 12.82. **MS (ESI, *m/z*, %):** 374.3 ([M+H]$^+$, 100). The spectroscopic data are consistent with those reported in the bibliography.[57]

(7*S*)-8,8-Dimethyl-2-oxo-7,8-dihydro-2*H*,6*H*-benzo[1,2-*b*:5,4-*b'*]dipyran-7-yl-6-{[2-(2,6-dioxopiperidin-3-yl)-1-oxo-2,3-dihydro-1*H*-isoindol-4-yl]amino}hexanoate (5). Following general procedure C, compound **35** (9.10 mg, 24.0 µmol), DCC (5.00 mg, 24.0 µmol) and DMAP (1.00 mg, 8.00 µmol) were dissolved in anhydrous DCM (0.50 mL) and a few drops of DMF were added to improve solubility. Decursinol (**1**) (5.00 mg, 20.0 µmol) was then added. The residue was purified by column chromatography (DCM to DCM/MeOH, 98:2) to obtain compound **5** as a solid (7.60 mg, 13.0 µmol, 62% yield).

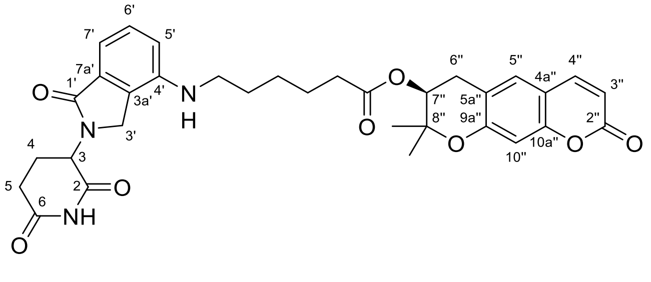

Mp: 114-116 ºC. **R**$_f$: 0.40 (DCM/MeOH, 9:1). **IR (ATR, cm^{-1})**: ν 2926 (C-H), 1708 (C=O), 1626 (C=C), 1193, 1136 (C-O). **^{1}H-NMR (300 MHz, Acetone-*d$_6$*)**: δ 1.36 (s, 6H, 2CH$_3$), 1.42 (m, 2H, C$\underline{H}_2$CH$_2$CH$_2$NH), 1.56 – 1.66 (m, 4H, C$\underline{H}_2$CH$_2$C$\underline{H}_2$CH$_2$NH), 2.14 – 2.26 (m, 1H, 1H$_4$), 2.29 – 2.36 (m, 2H, C$\underline{H}_2$CO), 2.40 – 2.54 (m, 1H, 1H$_4$), 2.72 – 2.79 (m, 1H, 1H$_5$), 2.82 – 2.90 (m, 1H, 1H$_{6''}$), 2.92 – 3.04 (m, 1H, 1H$_5$), 3.18 (q, *J* = 7.0 Hz, 2H, C$\underline{H}_2$NH), 3.24 (dd, *J* = 17.0, 5.1 Hz, 1H, 1H$_{6''}$), 4.17 – 4.35 (m, 2H, H$_{3'}$), 5.08 (t, *J* = 4.6 Hz, 1H, H$_{7''}$), 5.14 – 5.20 (m, 1H, H$_3$), 6.16 (d, *J* = 9.5 Hz, 1H, H$_{3''}$), 6.70 (s, 1H,

$H_{10''}$), 6.78 (d, *J* = 8.0, 1H, $H_{5'}$), 7.04 (d, *J* = 7.4 Hz, 1H, $H_{7'}$), 7.31 (t, *J* = 7.7 Hz, 1H, $H_{6'}$), 7.36 (s, 1H, $H_{5''}$), 7.80 (d, *J* = 9.5 Hz, 1H, $H_{4''}$), 9.81 (s, 1H, NHCO). **^{13}C-NMR (75 MHz, Acetone-*d₆*)**: δ 23.6 (CH_3), 24.2 (C4), 25.0 (CH_3), 25.4 ($\underline{C}H_2CH_2COO$), 27.1 ($\underline{C}H_2CH_2CH_2NH$), 28.3 (C6''), 29.5 ($\underline{C}H_2CH_2NH$), 32.2 (C5), 34.6 ($\underline{C}H_2CO$), 43.8 ($CH_2NH$), 46.1 (C3'), 52.7 (C3), 70.7 (C7''), 77.5 (C8''), 104.6 (C5''), 111.7 (C7'), 112.9 (C5'), 113.8 (C4a''), 113.9 (C3''), 116.8 (5a''), 127.7 (C3a'), 130.1 (C6'), 130.2 (C10''), 133.4 (C7a'), 144.3 (C4''), 144.7 (C4'), 155.1 (10a''), 157.2 (9a''), 161.0 (C2''), 170.1 (C1'), 171.5 (C2), 172.8 (C6), 173.2 (COO). **HPLC (t_R, min):** 12.59. **MS (ESI, *m/z*, %):** 602.3 ($[M+H]^+$, 100). HRMS (ESI) *m/z* calcd for $C_{33}H_{35}N_3O_8+Na^+$: 624.2322 $[M+Na]^+$; found, 624.2299.

2.6. Synthesis of PROTAC 6

***Tert*-butyl {2-[2-(2-chloroethoxy)ethoxy]ethoxy}acetate (36).** Following general procedure D, 2-[2-(2-chloroethoxy)ethoxy]ethanol (0.43 mL, 2.97 mmol) was dissolved in dry THF (10 mL) and was cooled to 0 °C. A mixture of potassium *tert*-butoxide (366 mg, 3.26 mmol) in dry THF (4.5 mL) was added, and after stirring the reaction mixture for 30 min at 0 °C, *tert*-butyl bromoacetate (0.53 mL, 3.56 mmol) was added. The residue was purified by flash chromatography (hex/EtOAc, 3:1) to obtain compound **36** as a colorless oil (210 mg, 0.743 mmol, 25% yield).

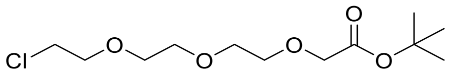

R_f: 0.40 (Hex/EtOAc, 6:4). **IR (ATR, cm^{-1})**: ν 2977 (C-H), 2873 (C-H), 1744 (C=O), 1113 (C-O). **^{1}H-NMR (300 MHz, $CDCl_3$)**: δ 1.47 (s, 9H, $(CH_3)_3$), 3.59 – 3.65 (m, 2H, CH_2Cl), 3.64-3.72 (m, 4H, $2CH_2O$), 3.70 (m, 4H, $2CH_2O$), 3.73 – 3.78 (m, 2H, CH_2O), 4.02 (s, 2H, CH_2COO). **^{13}C-NMR (75 MHz, $CDCl_3$):** δ 28.3 ($(CH_3)_3$), 42.8 (CH_2Cl), 69.2 ($\underline{C}H_2COO$), 70.7, 70.8, 70.8, 70.8, 71.5 ($5CH_2O$), 81.7 ($\underline{C}(CH_3)_3$), 169.8 (COO). The spectroscopic data are consistent with those reported in the bibliography.[58]

***Tert*-butyl {2-[2-(2-iodoethoxy)ethoxy]ethoxy}acetate (37).** Compound **36** (1.0 eq, 500 mg, 1.77 mmol) was dissolved in THF (2.8 mL) and NaI (2.0 eq, 530 mg) was added. The resulting mixture was stirred at 150 °C for 30 min in a MW reactor. The solvent was then evaporated under reduced pressure, DCM was added and the mixture was washed with $Na_2S_2O_3$ (x3) and brine. The organic solvent was then evaporated under reduced pressure to obtain compound **37** as a yellow oil (553 mg, 1.48 mmol, 84% yield).

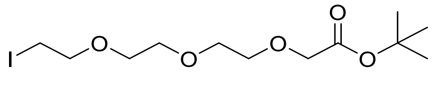

R_f: 0.48 (Hex/EtOAc, 6:4). **IR (ATR, cm^{-1}):** ν 2976 (C-H), 2873 (C-H), 1746 (C=O), 1123 (C-O). **^{1}H-NMR (300 MHz, $CDCl_3$):** δ 1.47 (s, 9H, $(CH_3)_3$), 3.26 (t, *J* = 6.5 Hz, 2H, CH_2I), 3.63-3.70 (m, 4H, $2CH_2O$), 3.69 – 3.73 (m, 4H, $2CH_2O$), 3.75 (t, *J* = 6.5 Hz,

2H, CH_2O), 4.02 (s, 2H, CH_2COO). **^{13}C-NMR (75 MHz, $CDCl_3$):** δ 3.0 (CH_2I), 28.3 ($(CH_3)_3$), 69.2 (CH_2COO), 70.3, 70.7, 70.8, 70.9, 72.1 ($5CH_2O$), 81.7 ($\underline{C}(CH_3)_3$), 169.8 (COO). **HPLC (t_R, min):** 13.61. **MS (ESI, *m/z*, %):** 435.2 ($[M+H+^iPrOH]^+$, 100).

{2-[2-(2-Iodoethoxy)ethoxy]ethoxy}acetic acid (38). Following general procedure B, *tert*-butyl ester **37** (100 mg, 0.267 mmol) was dissolved in DCM (2.7 mL) and TFA (2.0 mL, 26.7 mmol) was added. Compound **38** was obtained as a white solid (57.0 mg, 0.179 mmol, 67% yield).

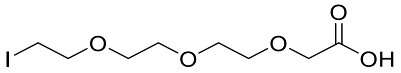

R_f: 0.10 (Hex/EtOAc, 1:1). **IR (ATR, cm^{-1})**: ν 2874 (C-H), 1738 (C=O), 1091 (C-O). **^{1}H-NMR (300 MHz, $CDCl_3$)**: δ 3.26 (t, *J* = 6.5 Hz, 2H, CH_2I), 3.63 – 3.80 (m, 10H, $5CH_2O$), 4.18 (s, 2H, CH_2COO), 8.12 (s, 1H, COOH). **^{13}C-NMR (75 MHz, $CDCl_3$):** δ 2.9 (CH_2I), 68.8 ($\underline{C}H_2COO$), 70.1, 70.4, 70.7, 71.5, 72.1 ($5CH_2O$), 173.4 (COO). **HPLC (t_R, min):** 10.49. **MS (ESI, *m/z*, %):** 319.1 ($[M+H]^+$, 100).

(7*S*)-8,8-Dimethyl-2-oxo-7,8-dihydro-2*H*,6*H*-benzo[1,2-*b*:5,4-*b'*]dipyran-7-yl {2-[2-(2-iodoethoxy)ethoxy]ethoxy}acetate (19). Following general procedure C, compound **38** (12.9 mg, 41.0 µmol), DCC (8.40 mg, 41.0 µmol) and DMAP (1.20 mg, 10.0 µmol) were dissolved in anhydrous DCM (0.20 mL). Decursinol (**1**) (5.00 mg, 20.0 µmol) was then added. The residue was purified by column chromatography (hex/EtOAc, 4:1 to 1:1) to obtain compound **19** (7.00 mg, 13.0 µmol, 63% yield).

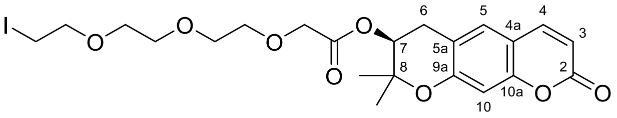

R_f: 0.65 (EtOAc). **IR (ATR, cm^{-1}):** ν 2930 (C-H), 1701, 1697 (C=O), 1626 (C=C), 1357 (C-H), 1089 (C-O). **^{1}H-NMR (300 MHz, $CDCl_3$):** δ 1.35 (s, 3H, CH_3), 1.37 (s, 3H, CH_3), 2.87 (dd, *J* = 17.1, 4.6 Hz, 1H, $1H_6$), 3.18 (dd, *J* = 17.1, 4.6 Hz, 1H, $1H_6$), 3.24 (t, *J* = 7.3 Hz, 2H, CH_2I), 3.60 – 3.79 (m, 10H, $5CH_2O$), 4.16 (d, *J* = 2.9 Hz, 2H, CH_2COO), 5.14 (t, *J* = 4.8 Hz, 1H, H_7), 6.24 (d, *J* = 9.5 Hz, 1H, H_3), 6.79 (s, 1H, H_{10}), 7.15 (s, 1H, H_5), 7.59 (d, *J* = 9.5 Hz, 1H, H_4). **^{13}C-NMR (75 MHz, $CDCl_3$):** δ 3.2 (CH_2I), 23.2 (CH_3), 24.1 (CH_3), 28.6 (C6), 68.0, 68.4, 69.6, 70.0, 70.6, 72.2 ($6CH_2O$), 74.2 (C7), 76.3 (C8), 104.5 (C10), 112.4 (C3), 113.1 (C4a), 117.8 (C5a), 128.2 (C5), 143.3 (C4), 155.7 (C10a), 156.2 (C9a), 161.4 (C2), 170.7 (COO).

(7*S*)-8,8-Dimethyl-2-oxo-7,8-dihydro-2*H*,6*H*-benzo[1,2-*b*:5,4-*b'*]dipyran-7-yl {2-[2-(2-{[2-(2,6-dioxopiperidin-3-yl)-1-oxo-2,3-dihydro-1*H*-isoindol-4-yl]amino}ethoxy)-ethoxy]ethoxy}acetate (6). Following general procedure A, lenalidomide (9.50 mg, 37.0 µmol) was dissolved in DMF (0.18 mL) and DIPEA (20 µL, 0.110 mmol) and compound

19 (10.0 mg, 18.0 μmol) were added. The resulting residue was purified by column chromatography (DCM to DCM/MeOH 98:2) to obtain compound **6** as a solid (4.00 mg, 6.00 μmol, 32% yield).

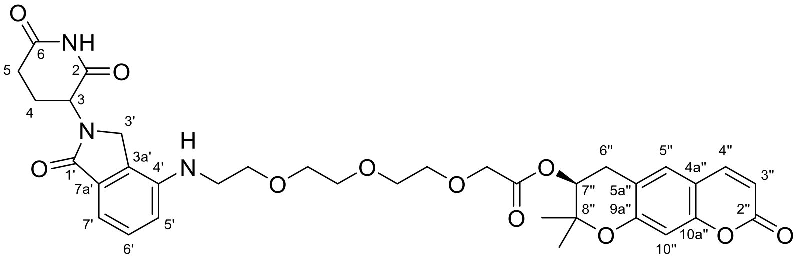

Mp: 86-88 °C. **R_f:** 0.83 (DCM/MeOH, 9:1). **IR (ATR, cm^{-1}):** ν 2922 (C-H), 1699, 1694 (C=O), 1624 (C=C), 1196, 1134 (C-O). **^{1}H-NMR (500 MHz, Acetone):** δ 1.37 (s, 6H, $2CH_3$), 2.19 (m, 1H, $1H_4$), 2.50 (m, 1H, $1H_4$), 2.88 – 3.04 (m, 2H, $1H_5$, $1H_{6''}$), 3.27 (m, 2H, $1H_5$, $1H_{6''}$), 3.40 (q, J = 5.5 Hz, 2H, C<u>H</u>$_2$NH), 3.56 (m, 6H, $3CH_2O$), 3.62 (m, 2H, CH_2O), 3.68 (t, J = 5.5 Hz, 2H, CH_2O), 4.07 – 4.22 (m, 2H, CH_2COO), 4.23 – 4.35 (m, 2H, $H_{3'}$), 4.84 (s, 1H, ArNH), 5.17 (m, 2H, $H_{7''}$, H_3), 6.19 (d, J = 9.5 Hz, 1H, $H_{3''}$), 6.70 (s, 1H, $H_{10''}$), 6.85 (d, J = 8.1, 1H, $H_{5'}$), 7.05 (d, J = 7.6 Hz, 1H, $H_{7'}$), 7.31 (t, J = 7.7 Hz, 1H, $H_{6'}$), 7.38 (s, 1H, $H_{5''}$), 7.82 (d, J = 9.6 Hz, 1H, $H_{4''}$), 9.74 (s, 1H, NHCO). **^{13}C-NMR (126 MHz, Acetone):** δ 23.7 (CH_3), 24.1 (C4), 25.0 (CH_3), 29.2 (C6’’), 32.3 (C5), 44.0 (CH_2NH), 46.0 (C3’), 52.7 (C3), 68.8, 70.0, 71.0 ($3CH_2O$), 71.1 (2C, C7’’ and CH_2O), 71.2, 71.3, ($2CH_2O$), 77.4 (C8’’), 104.6 (C5’’), 112.1 (C7’), 113.3 (C5’), 113.8 (C4a’’), 113.9 (C3’’), 116.7 (C5a’’), 128.0 (C3a’), 130.1 (C6’), 130.3 (C10’’), 133.5 (C7a’), 144.3 (C4’’), 144.7 (C4’), 155.1 (C10a’’), 157.2 (C9a’’), 160.9 (C2’’), 169.9 (C1’), 170.7, 171.5 (C2, C6), 172.8 (COO). **HPLC (t_R, min):** 11.71. **MS (ESI, *m/z*, %):** 678.2 ($[M+H]^+$, 100). HRMS (ESI) m/z: calcd for $C_{35}H_{39}N_3O_{11}{}^+[M+H]^+$, 678.2662; found, 678.2654.

2.7. Synthesis of PROTAC 7

***Tert*-butyl 2-(4-(2-(2,6-dioxopiperidin-3-yl)-1,3-dioxoisoindolin-4-yl)piperazin-1-yl)acetate (39).** *Tert*-butyl 2-(piperazin-1-yl)acetate (1.0 eq, 86 mg, 0.43 mmol) was dissolved in DMF (2.1 mL) and DIPEA (4.0 eq, 0.30 mL) and 4-fluoro-thalidomide were added. The mixture was stirred at 180 °C for 40 min in a MW reactor. When finished, the mixture was diluted with EtOAc, washed with water (x5), dried over anhydrous Na_2SO_4 and concentrated under reduced pressure. The crude was purified by flash chromatography (Hex to Hex:EtOAc 3:2) to afford compound **39** as an oil (169 mg, 0.37 mmol, 86% yield).

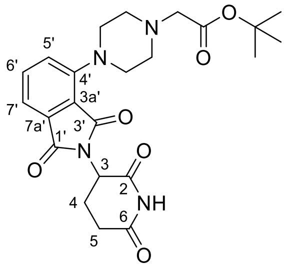

Rf: 0.38 (EtOAc). **IR (ATR, cm^{-1}):** 3240 (ν =C-H), 2926 (C-H), 2854 (C-H), 1699 (ν C=O), 1204 (ν C-O). **^{1}H-NMR (700 MHz, $CDCl_3$):** δ 1.48 (s, 9H, C(C<u>H</u>$_3$)$_3$), 2.12 (dt, *J* = 12.8, 3.8 Hz, 1H, H_4), 2.72 (ddd, *J* = 18.3, 13.7, 3.1 Hz, 1H, H_5), 2.78-2.92 (m, 6H, piperazine, H_4, H_5), 3.23 (s, 2H, CH_2COO), 3.44 (m, 4H, piperazine), 4.95 (dd , 1H, 12.7, 5.4 Hz, H_3), 7.18 (d, *J* = 8.3 Hz, 1H, $H_{5'}$), 7.42 (d, *J* = 7.1 Hz, 1H, $H_{7'}$), 7.60 (t, *J* = 7.8 Hz, 1H, $H_{6'}$), 7.98 (s, 1H, NHCO). **^{13}C-NMR (175 MHz, $CDCl_3$):** δ 22.8 (C4), 28.3 (3C, C(<u>C</u>H$_3$)$_3$), 31.6 (C5), 49.3 (C3), 51.0 (2C, piperazine), 53.0 (2C, piperazine), 59.8 (<u>C</u>H$_2$COO), 81.6 (<u>C</u>(CH$_3$)$_3$), 116.1 (C7'), 117.7 (C3a'), 123.6 (C5'), 134.2 (C7a'), 135.9 (C6'), 150.5 (C4'), 166.8 (C2), 167.6 (C1'), 167.4 (C3'), 168.2 (COO), 170.9 (C6). **MS (ESI, *m/z*, %):** 457.1 ([M+H]$^+$, 100). **R_t:** 9.48 min.

2-(4-(2-(2,6-dioxopiperidin-3-yl)-1,3-dioxoisoindolin-4-yl)piperazin-1-yl)acetic acid (40). Following general procedure B, *tert*-butyl ester **39** (35 mg, 0.08 mmol) was dissolved in DCM (0.4 mL) and TFA (0.59 mL, 7.67 mmol) was added. Compound **40** was obtained as a solid (24 mg, 0.06 mmol, 78% yield).

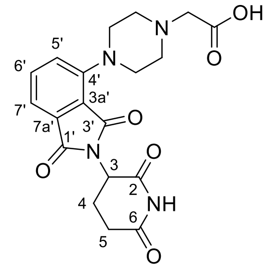

Mp: 108-110 ºC. **Rf**: 0.10 (EtOAc). **IR (ATR, cm^{-1}):** 3273 (ν =C-H), 2926 (C-H), 2855 (C-H), 1696 (ν C=O), 1199 (ν C-O). **^{1}H-NMR (700 MHz, DMSO- δ_6):** δ 1.99-2.05 (m, 1H, 1H_4), 2.51-2.62 (m, 3H, 1H_4, 2H_5), 2.94 (m, 4H, piperazine), 3.40 (m, 6H, 4H piperazine, CH_2COO), 5.09 (dd, *J* = 12.8, 5.4 Hz, 1H, H_3), 7.37 (d, *J* = 8.5 Hz, 1H, $H_{5'}$), 7.39 (d, *J* = 7.1 Hz, 1H, $H_{7'}$), 7.72 (t, *J* = 7.8 Hz, 1H, $H_{6'}$), 11.1 (s, 1H, COOH). **^{13}C-NMR (175 MHz, DMSO- δ_6):** δ 22.5 (C4), 31.4 (C5), 49.3 (C3), 50.1 (2C, piperazine), 52.3 (2C, piperazine), 58.2 (CH_2COO), 117.3 (C3a'), 115.7 (C7'), 124.4 (C5'), 134.1 (C7a'), 136.4 (C6'), 149.7 (C4'), 166.8 (C3'), 167.5 (C1'), 170.5 (C2), 170.6 (COO), 173.3 (C6). **(ESI, *m/z*, %):** 401.1 ([M+H]$^+$, 100). **R_t:** 8.78 min.

(*S*)-8,8-dimethyl-2-oxo-7,8-dihydro-2*H*,6*H*-pyrano[3,2-*g*]chromen-7-yl 2-(4-(2-(2,6-dioxopiperidin-3-yl)-1,3-dioxoisoindolin-4-yl)piperazin-1-yl)acetate (7). Following general procedure C, compound **40** (15 mg, 37 μmol), DCC (7.7 mg, 37 μmol) and DMAP

(1.8 mg, 15 µmol) were dissolved in DCM (0.25 mL). Decursinol (**1**) (9.2 mg, 37 µmol) dissolved in DCM (100 µL) was then added. The residue was dissolved in the minimum amount of DCM/MeOH (95:5) and purified by preparative TLC using the same solvent mixture as eluent to obtain compound **7** as a solid (12 mg, 19 µmol, 51% yield).

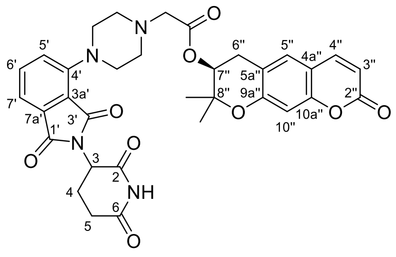

Mp: 113-115 °C. **Rf**: 0.29 (EtOAc). **IR (ATR, cm^{-1}):** ν 2926 (C-H), 2854 (C-H), 1704 (ν C=O), 1195 (ν C-O). **^{1}H-NMR (500 MHz, $CDCl_3$):** δ 1.36 (s, 3H, $C\underline{H}_3$), 1.39 (s, 3H, $C\underline{H}_3$), 2.07-2.15 (m, 1H, H_4), 2.70-2.94 (m, 8H, 4H, piperazine, H_4, H_5), 3.20 (dd, J = 17.2, 4.5 Hz, 1H, $H_{6''}$), 3.33 (s, 2H, $C\underline{H}_2$COO), 3.34-3.45 (m, 4H, piperazine), 4.95 (dd, J = 12.0, 5.3 Hz, 1H, H_3), 5.13 (t, J = 4.6 Hz, 1H, $H_{7''}$), 6.19 (dd, J = 9.5, 5.2 Hz, 1H, $H_{3''}$), 6.78 (s, 1H, $H_{10''}$), 7.10-7.16 (m, 2H, $H_{5'}$, $H_{5''}$), 7.40 (d, J = 7.1 Hz, 1H, $H_{7'}$), 7.56 (d, J = 9.5 Hz, 1H, $H_{4''}$), 7.59 (dd, J = 8.4, 7.2 Hz, 1H, $H_{6'}$), 8.22 (s, 1H, NHCO). **^{13}C-NMR (125 MHz, $CDCl_3$):** δ 22.8 (C4), 23.6 ($\underline{C}H_3$), 25.0 ($\underline{C}H_3$), 27.9 (C6''), 31.5 (C5), 49.3 (C3), 50.8 (2C, piperazine), 52.9 (2C, piperazine), 59.0 ($\underline{C}H_2$COO), 70.9 (C7''), 76.5 ($\underline{C}(CH_3)_2$), 104.9 (C10''), 113.1 (C4a''), 113.6 (C3''), 115.4 (C5a''), 116.1 (C7'), 117.7 (C3a'), 123.5 (C5'), 128.9 (C5''), 134.2 (C7a'), 135.9 (C6'), 143.2 (C4''), 150.2 (C4'), 154.4 (C10a''), 156.4 (C9a''), 161.3 (C2''), 166.8 (C3'), 167.4 (C2), 168.3 (C1'), 169.6 ($CH_2\underline{C}OO$), 171.1 (C6). **(ESI, *m/z*, %):** 629.1 ($[M+H]^+$, 100). **R_t:** 10.22 min. HRMS (ESI) m/z: calcd for $C_{33}H_{32}N_4O_9^+[M+H]^+$, 629.2247; found, 629.2226.

2.8. Synthesis of PROTAC 8

***Tert*-butyl 2-(4-(2-(2,6-dioxopiperidin-3-yl)-1,3-dioxoisoindolin-4-yl)piperazin-1-yl)acetate (41).** *Tert*-butyl 2-(piperazin-1-yl)acetate (1.0 eq, 195 mg, 0.78 mmol) was dissolved in DMF (4.9 mL) and Et_3N (1.2 eq, 0.16 mL) and 2-(2-bromoethyl)isoindoline-1,3-dione (1.1 eq, 272 mg, 1.07 mmol) were added. The mixture was stirred at 180 °C for 40 min in a MW reactor. When finished, the mixture was diluted with EtOAc, washed with water (x5), dried over anhydrous Na_2SO_4, and concentrated under reduced pressure. The crude was purified by flash chromatography (Hex to Hex/EtOAc 1:1), to afford compound **41** as a solid (149 mg, 0.40 mmol, 41% yield).

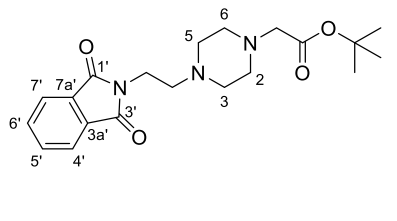

Mp: 69-72 °C. **Rf:** 0.10 (Hex/EtOAc, 7:3). **IR (ATR, cm^{-1}):** ν 1772, 1740, 1708 (C=O), 1438 (C=C), 1393 (C-N), 1153 (O-C-C). **^{1}H-NMR (300 MHz, $CDCl_3$):** δ 1.43 (s, 9H, $C(CH_3)_3$), 2.53 (s, br, 4H, H_2, H_6), 2.58 (s, br, 4H, H_3, H_5), 2.63 (t, *J* = 6.6 Hz, 2H, $NH_2CH_2C\underline{H}_2$), 3.04 (s, 2H, CH_2COO), 3.80 (t, *J* = 6.6 Hz, 2H, $NH_2C\underline{H}_2CH_2$), 7.66-7.73 (m, 2H, $H_{5'}$, $H_{6'}$), 7.79-7.86 (m, 2H, $H_{4'}$, $H_{7'}$). **^{13}C-NMR (75 MHz, $CDCl_3$):** δ 28.2 (3C, $C(\underline{C}H_3)_3$), 35.4 ($N\underline{C}H_2CH_2$), 52.9 (2C, piperazine ring), 53.0 (2C, piperazine ring), 55.7 ($NCH_2\underline{C}H_2$), 60.8 ($\underline{C}H_2COO$), 81.1 ($\underline{C}(CH_3)_3$), 123.3 (C4', C7'), 132.3 (2C, C3a', C7a'), 134.0 (C5', C6'), 168.5 (2C, C1', C3'), 169.6 (COO). **(ESI, *m/z*, %):** 374.2 ($[M+H]^+$, 100). **R_t:** 9.58 min.

***Tert*-butyl-[4-(2-aminoethyl)piperazin-1-yl]acetate (20).** To a solution of piperazine **41** (1 eq, 145 mg, 0.39 mmol) in EtOH (3.1 mL) was added a solution of commercial hydrazine hydrate 60-65% (6.5 eq, 0.2 mL) and was stirred at 80 ºC for 2 h. The reaction crude was filtered off and triturated with Et_2O, and the filtrate was collected and concentrated under vacuum yielding compound **20** as an oil (94.5 mg, 0.39 mmol, 99% yield), which was used in the next step without further purification.

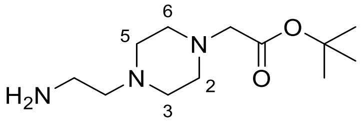

IR (ATR, cm^{-1}): ν 3293 (N-H), 1738 (C=O), 1659 (C-N), 1154 (O-C-C). **^{1}H-NMR (300 MHz, $CDCl_3$):** δ 1.40 (s, 9H, $C(CH_3)_3$), 2.07 (s, br, 2H, NH_2), 2.37 (t, *J* = 6.2 Hz, 2H, $NH_2CH_2C\underline{H}_2$), 2.47 (m, 4H, H_2, H_6), 2.53 (m, 4H, H_3, H_5), 2.73 (t, *J* = 6.1 Hz, 2H, $NH_2C\underline{H}_2CH_2$), 3.04 (s, 2H, CH_2COO). **^{13}C-NMR (75 MHz, $CDCl_3$):** δ 28.2 (3C, $C(\underline{C}H_3)_3$), 38.7 ($NH_2\underline{C}H_2CH_2$), 53.0 (2C, piperazine), 53.1 (2C, piperazine ring), 60.0 ($\underline{C}H_2COO$), 60.9 ($NH_2CH_2\underline{C}H_2$), 81.0 ($\underline{C}(CH_3)_3$), 169.6 (COO). **(ESI, *m/z*, %):** 244.2 ($[M+H]^+$, 100). **R_t:** 9.18 min.

***Tert*-butyl[4-(2-{[2-(2,6-dioxopiperidin-3-yl)-1,3-dioxo-2,3-dihydro-1*H*-isoindol-4-yl]amino}ethyl)piperazin-1-yl]acetate (42)**. To a stirred solution of **20** (1 eq, 88 mg, 0.32 mmol) in NMP (1 mL) a solution of 4-fluoro-thalidomide (1.2 eq, 93 mg, 0.38 mmol) and DIPEA (3 eq, 0.17 mL) in NMP (0.6 mL) was added. The mixture was stirred at 110 °C for 3 h under MW. After adding EtOAc (50 mL), the crude was washed with water (x6) and extracted with EtOAc (x3). The organic phases were combined, dried over Na_2SO_4, filtered and concentrated under vacuum. The resulting residue was purified by flash chromatography (hex/EtOAc, 3:2 to EtOAc) to obtain compound **42** as an oil (35 mg, 0.07 mmol, 22% yield).

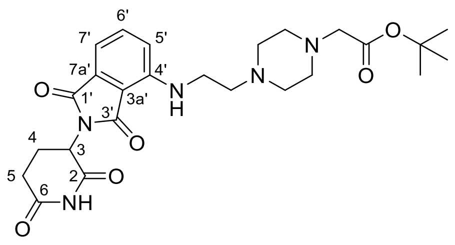

Rf: 0.40 (EtOAc/MeOH, 95:5). **IR (ATR, cm^{-1}):** ν 3406 (N-H), 1737, 1693 (C=O), 1624 (C=C), 1363 (C-N), 1160 (O-C-C).**^{1}H-NMR (300 MHz, CDCl$_3$):** δ 1.45 (s, 9H, C(C$\underline{H}_3$)$_3$), 2.06-2.16 (m, 1H, H$_4$), 2.60 (m, 8H, piperazine), 2.64-2.71 (m, 2H, NHCH$_2$C$\underline{H}_2$), 2.73-2.90 (m, 3H, H$_4$, H$_5$), 3.11 (s, 2H, CH$_2$COO), 3.33 (q, *J* = 5.9 Hz, 2H, NHC$\underline{H}_2$CH$_2$), 4.85-4.97 (m , 1H, H$_3$), 6.64 (t, *J* = 5.0 Hz, 1H, N$\underline{H}$CH$_2$CH$_2$), 6.85 (d, *J* = 8.5 Hz, 1H, H$_{5'}$), 7.07 (d, *J* = 7.2 Hz, 1H, H$_{7'}$), 7.47 (dd, *J* = 8.5, 7.1 Hz, 1H, H$_{6'}$), 8.70 (s, 1H, NHCO). **^{13}C-NMR (75 MHz, CDCl$_3$):** δ 22.9 (C4), 28.7 (3C, C($\underline{C}$H$_3$)$_3$), 31.6 (C5), 39.4 (NH$_2\underline{C}$H$_2$CH$_2$), 49.0 (C3), 52.8 (2C, piperazine), 53.0 (2C, piperazine), 56.2 (NHCH$_2\underline{C}$H$_2$), 60.1 ($\underline{C}$H$_2$COO), 81.2 (O$\underline{C}$(CH$_3$)$_3$), 110.3 (C3a'), 111.5 (C7'), 116.9 (C5'), 132.7 (C7a'), 136.2 (C6'), 146.8 (C4'), 167.8 (C1'), 168.7 (C2), 169.2 (C3'), 169.7 (COO), 171.4 (C6). **(ESI, *m/z*, %):** 500.2 ([M+H]$^+$, 100). **R$_t$:** 9.58 min.

1-(carboxymethyl)-4-(2-{[2-(2,6-dioxopiperidin-3-yl)-1,3-dioxo-2,3-dihydro-1*H*-isoindol-4-yl]amino}ethyl)piperazine-1,4-diium bis(trifluoroacetate) (43). Following general procedure B, *tert*-butyl ester **42** (30 mg, 0.06 mmol) was dissolved in DCM (0.6 mL) and TFA (0.46 mL, 6.00 mmol) was added to obtain compound **43** as the trifluoroacetate (TFA) salt (38 mg, 0.06 mmol, 94 %).

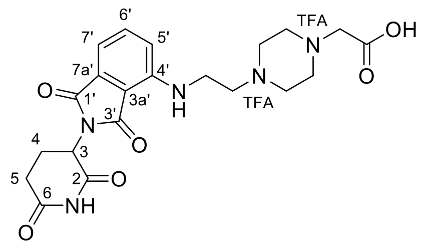

^{1}H-NMR (300 MHz, Acetone-*δ$_6$*): δ 2.11-2.26 (m, 1H, H$_4$), 2.67-3.00 (m, 3H, H$_4$, H$_5$), 3.30 (m, 4H, piperazine), 3.46 (m, 2H, NHC$\underline{H}_2$CH$_2$), 3.51-3.61 (m, 4H, piperazine), 3.68 (s, 2H, CH$_2$COO), 3.94 (m, 2H, NHCH$_2$C$\underline{H}_2$), 5.07 (dd, *J* = 12.2, 5.2 Hz, 1H, H$_3$), 7.10 (d, *J* = 7.0 Hz, 1H, H$_{5'}$), 7.26 (d, *J* = 8.3 Hz, 1H, H7'), 7.61 (t, *J* = 7.6 Hz, 1H, H6'), 9.93 (s, 1H, NHCO). **^{13}C-NMR (75 MHz, Acetone-*δ$_6$*):** δ 23.5 (C4), 32.0 (C5), 38.2 (NH$_2$CH$_2\underline{C}$H$_2$), 49.3 (C3), 50.3 (2C, piperazine), 52.0 (2C, piperazine), 55.7 ($\underline{C}$H$_2$COO), 58.0 (NH$\underline{C}$H$_2$CH$_2$), 111.9 (C3a'), 112.3 (C5'), 118.0 (C7'), 133.7 (C7a'), 137.2 (C6'), 146.9 (C4'), 168.2 (C3'), 170.0 (C2), 170.2 (C1), 170.8 (COO), 172.6 (C6). **(ESI, *m/z*, %):** 441.0 ([M-2]$^{+\cdot}$, 100). **R$_t$:** 8.94 min.

(*S*)-8,8-dimethyl-2-oxo-7,8-dihydro-2H,6H-pyrano[3,2-g]chromen-7-yl 2-(4-(2-((2-(2,6-dioxopiperidin-3-yl)-1,3-dioxoisoindolin-4-yl)amino)ethyl)piperazin-1-

yl)acetate (8). Following general procedure C, compound **43** (13.6 mg, 20 µmol), DCC (6.3 mg, 30 µmol) and DMAP (1 mg, 8 µmol) were dissolved in DCM (0.25 mL). Decursinol (**1**) (5 mg, 20 µmol) dissolved in DCM (100 µL) was then added. The residue was dissolved in the minimum amount of EtOAc/EtOH (9:1) and purified by preparative TLC using the same solvent mixture as eluent to obtain compound **8** as a solid (2.2 mg, 3 µmol, 16% yield).

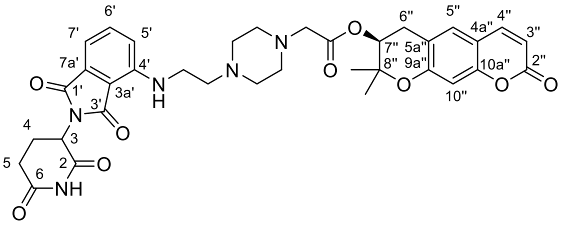

Mp: 122-124 °C. **Rf**: 0.36 (EtOAc/EtOH 9:1). **IR (ATR, cm^{-1}):** ν 3406 (N-H), 1737, 1693 (C=O), 1624 (ν C=C), 1363 (C-N), 1160 (O-C-C). **^{1}H-NMR (700 MHz, $CDCl_3$):** δ 1.35 (s, 3H, CH_3), 1.37 (s, 3H, CH_3), 2.10-2.13 (m, 1H, H_4), 2.54-2.75 (m, 10H, piperazine, NHCH2C<u>H</u>2), 2.76-2.92 (m, 4H, H_4, H_5, $H_{6''}$), 3.19 (dd, *J* = 17.0, 4.8 Hz, 1H, $H_{6''}$), 3.24 (d, *J* = 5.8 Hz, 2H, CH_2COO), 3.33 (m, 2H, NHC<u>H</u>2CH2), 4.91 (dd, *J* = 12.8, 5.5 Hz, 1H, H_3), 5.11 (t, *J* = 4.8 Hz, 1H, $H_{7''}$), 6.23 (d, *J* = 9.5 Hz, 1H, $H_{3''}$), 6.65 (s br, 1H, N<u>H</u>CH2CH2), 6.79 (s, 1H, H10''), 6.87 (s, 1H, $H_{5''}$), 7.09 (d, *J* = 7.1 Hz, 1H, $H_{7'}$), 7.15 (s, 1H, $H_{5'}$), 7.49 (t, *J* = 7.8 Hz, 1H, $H_{6'}$), 7.57 (d, *J* = 9.5 Hz, 1H, $H_{4''}$), 8.19 (s, 1H, NHCO). **^{13}C-NMR (175 MHz, $CDCl_3$):** δ 22.9 (C4), 23.4 (CH_3), 25.1 (CH_3), 27.9 (C6''), 31.6 (C5), 39.3 (NH2<u>C</u>H2CH2), 49.0 (C3), 52.6 (4C, piperazine), 56.0 (NHCH2<u>C</u>H2), 59.1 (<u>C</u>H2COO), 70.6 (C7''), 76.6 (C8''), 104.9 (C10''), 110.4 (C3a'), 111.7 (C7'), 113.6 (C3''), 113.1 (C4a'), 115.5 (C5a'), 117.0 (C5'), 128.8 (C5''), 132.7 (C7a'), 136.3 (C6'), 143.2 (C4''), 146.8 (C4'), 154.4 (C10a''), 156.4 (C9a''), 161.4 (C2''), 167.8 (C1'), 168.5 (C2), 169.3 (C3'), 169.9 (CH2<u>C</u>OO), 171.2 (C6). **(ESI, *m/z*, %):** 672.2 ([M+H]$^+$, 100). **R_t:** 9.86 min. HRMS (ESI) *m/z* calcd for $C_{35}H_{37}N_5O_9^+$: 672.2669 [M+H]$^+$; found, 672.2647.

2.9. Synthesis of PROTAC 9

5-Azidopentan-1-amine (21). 5-bromopentan-1-aminium bromide, synthesized as previously described[60] (1.0 eq, 600 mg, 2.43 mmol) and NaN_3 (3.0 eq, 474 mg, 7.29 mmol) were dissolved in water (3.0 mL) and heated in a MW reactor at 130 °C for 30 min. The reaction was then cooled to 0 °C and 1M KOH (3.0 mL) and diethyl ether (3.0 mL) were added. The aqueous layer was extracted twice with diethyl ether, and the organic phase was washed with brine and dried with anhydrous Na_2SO_4. The solvent was removed under reduced pressure and the obtained residue was purified by flash chromatography (DCM/MeOH, 29:1) to obtain compound **21** as an oil (200 mg, 1.56 mmol, 64% yield).

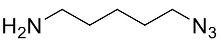

R_f: 0.15 (DCM/MeOH/NH_3, 90:10:1). **IR (ATR, cm^{-1})**: ν 2933, 2861 (C-H), 2090 (N=N). **^{1}H-NMR (300 MHz, Acetone-*d₆*):** δ 1.35 – 1.50 (m, 2H, C<u>H</u>2CH2CH2NH2), 1.51 – 1.68 (m, 4H, C<u>H</u>2CH2C<u>H</u>2CH2NH2), 2.07 – 2.09 (m, 2H, CH2N<u>H</u>2), 3.17 (t, *J* = 6.7 Hz, 2H,

$C\underline{H}_2N_3$), 3.33 (t, *J* = 6.8 Hz, 2H, $C\underline{H}_2NH_2$). **^{13}C-NMR (75 MHz, Acetone-*d*$_6$):** δ 25.4 ($\underline{C}H_2CH_2CH_2NH_2$), 29.5 ($\underline{C}H_2CH_2NH_2$), 31.2 ($\underline{C}H_2CH_2N_3$), 51.6 ($CH_2NH_2$), 52.0 ($CH_2N_3$). **HPLC ($t_R$, min):** 3.37. **MS (ESI, *m/z*, %):** 129.2 ($[M+H]^+$, 100). The spectroscopic data are consistent with those reported in the bibliography.[59]

4-[(5-Azidopentyl)amino]-2-(2,6-dioxopiperidin-3-yl)-1*H*-isoindole-1,3(2*H*)-dione (44). Following general procedure A, 4-fluoro-thalidomide (150 mg, 0.543 mmol) was dissolved in DMF (2.7 mL) and DIPEA (0.28 mL, 1.63 mmol) and compound **21** (104 mg, 0.815 mmol) were added. The resulting residue was purified by flash chromatography (DCM/MeOH, 99:1) to obtain compound **44** as a solid (81.0 mg, 0.211 mmol, 39% yield).

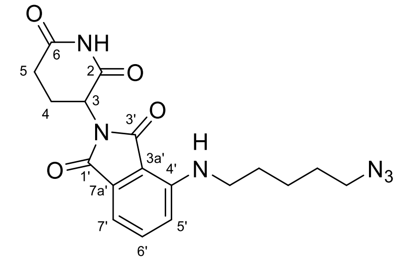

Mp: 126-128 °C. **R$_f$**: 0.52 (Hex/EtOAc, 4:6). **IR (ATR, cm^{-1})**: ν 3240 (N-H), 2935 (C-H), 2860 (C-H), 2096 (N=N), 1694 (C=O). **^{1}H-NMR (300 MHz, CDCl$_3$):** δ 1.50 (d, *J* = 6.6 Hz, 2H, $NHCH_2CH_2C\underline{H}_2$), 1.55 – 1.77 (m, 4H, $NHCH_2C\underline{H}_2CH_2C\underline{H}_2$), 2.00 – 2.19 (m, 1H, $1H_4$), 2.62 – 2.93 (m, 3H, $1H_4$, H_5), 3.29 (td, *J* = 6.8, 1.5 Hz, 4H, $NHC\underline{H}_2$, $C\underline{H}_2N_3$), 4.84 – 4.97 (m, 1H, H_3), 6.24 (t, *J* = 5.7 Hz, 1H, ArNH), 6.86 (d, *J* = 8.5 Hz, 1H, $H_{5'}$), 7.08 (dd, *J* = 7.2, 0.6 Hz, 1H, $H_{7'}$), 7.48 (dd, *J* = 8.5, 7.1 Hz, 1H, $H_{6'}$), 8.44 (s, 1H, NHCO). **^{13}C-NMR (75 MHz, CDCl$_3$):** δ 22.9 (C4), 24.2 ($NHCH_2CH_2\underline{C}H_2$), 28.7 ($NHCH_2\underline{C}H_2$), 28.9 ($\underline{C}H_2CH_2N_3$), 31.5 (C5), 42.5 ($NHCH_2$), 49.0 (C3), 51.3 ($CH_2N_3$), 110.1 (C3a’), 111.6 (C7’), 116.7 (C5’), 132.6 (C7a’), 136.3 (C6’), 147.0 (C4’), 167.7 (C3’), 168.6 (C1’), 169.6 (C2), 171.4 (C6). **HPLC (t_R, min):** 13.24. **MS (ESI, *m/z*, %):** 385.2 ($[M+H]^+$, 100). The spectroscopic data are consistent with those reported in the bibliography.[61]

(7*S*)-8,8-Dimethyl-2-oxo-7,8-dihydro-2*H*,6*H*-benzo[1,2-*b*:5,4-*b'*]dipyran-7-yl (2*E*)-3-(4-{[1-(5-{[2-(2,6-dioxopiperidin-3-yl)-1,3-dioxo-2,3-dihydro-1*H*-isoindol-4-yl]amino}-pentyl)-1*H*-1,2,3-triazol-4-yl]methoxy}phenyl)prop-2-enoate (9). To a mixture of azide **44** (1.0 eq, 13.4 mg, 35.0 μmol), copper(II) sulfate (0.50 eq, 4.40 mg, 17.0 μmol) and sodium ascorbate (1.0 eq, 6.90 mg, 35.0 μmol) in DMF (0.14 mL) and water (0.39 mL) was added alkyne **22** synthesized as previously reported[35] (1.0 eq, 15.0 mg, 35.0 μmol) and the mixture was stirred at rt for 16 h. When finished, the mixture was diluted with water and extracted with EtOAc (x3). The crude product was purified by column chromatography (DCM to DCM/MeOH, 98:2) to obtain compound **9** as a solid (20.0 mg, 24.5 μmol, 70% yield).

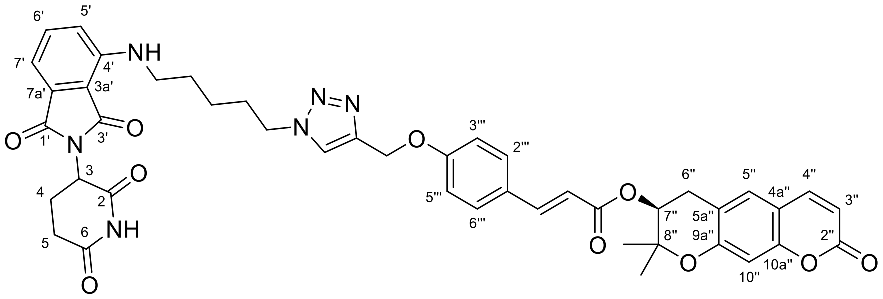

Mp: 138-140 ºC. **R$_f$**: 0.40 (DCM/MeOH, 9:1). **IR (ATR, cm^{-1})**: ν 2923 (C-H), 1697 (C=O), 1625 (C=C), 1601 (C=C), 1161 (C-O), 1134 (C-O). **^{1}H-NMR (300 MHz, CDCl$_3$):** δ 1.38 (s, 3H, CH_3), 1.42 (s, 3H, CH_3), 1.47 (dd, *J* = 9.4, 6.0 Hz, 2H, $NHCH_2CH_2C\underline{H}_2$), 1.61 – 1.77 (m, 2H, $NHCH_2C\underline{H}_2$), 1.89 – 2.05 (m, 2H, $C\underline{H}_2CH_2N_{Trz}$), 2.06 – 2.20 (m, 1H, $1H_4$), 2.62 – 2.99 (m, 4H, $1H_4$, $2H_5$, $1H_{6''}$), 3.16 – 3.30 (m, 3H, $1H_{6''}$, $NHC\underline{H}_2$), 4.37 (t, *J* = 7.1 Hz, 2H, CH_2N_{Trz}), 4.91 (dd, *J* = 12.0, 5.4 Hz, 1H, H_3), 5.18 (t, *J* = 4.7 Hz, 1H, $H_{7''}$), 5.22 (s, 2H, CH_2O), 6.22 (d, *J* = 9.5 Hz, 1H, $H_{3''}$), 6.27 (d, *J* = 16.0 Hz, 1H, CHCOO), 6.81 (s, 1H, $H_{10''}$), 6.84 (d, *J* = 8.5 Hz, 1H, $H_{5'}$), 6.97 (d, *J* = 8.8 Hz, 2H, $H_{3'''}$, $H_{5'''}$), 7.08 (d, *J* = 7.1 Hz, 1H, $H_{7'}$), 7.16 (s, 1H, $H_{5''}$), 7.44 (d, *J* = 8.7 Hz, 2H, $H_{2'''}$, $H_{6'''}$), 7.49 (t, *J* = 7 Hz, 1H, $H_{6'}$), 7.54 – 7.67 (m, 2H, $N\underline{H}CH_2$, CH_{Trz}), 7.58 (d, *J* = 9.6 Hz, 1H, $H_{4''}$), 7.61 (d, *J* = 15.0 Hz, 1H, $C\underline{H}CHCOO$), 8.22 (s, 1H, NHCO). **^{13}C-NMR (75 MHz, CDCl$_3$):** δ 22.9 (C4), 23.5 (CH_3), 24.0 ($\underline{C}H_2CH_2CH_2N_{Trz}$), 25.1 ($CH_3$), 28.0 (C6’’), 28.7 ($C\underline{H}_2CH_2N_{Trz}$), 30.1 ($NHCH_2\underline{C}H_2$), 31.5 (C5), 42.4 ($CH_2NH$), 49.0 (C3), 50.3 ($CH_2N_{Trz}$), 62.2 ($CH_2O$), 70.2 (C7’’), 77.8 (C8’’), 104.9 (C10’’), 110.2 (C3a’), 111.8 (C7’), 113.0 (C4a’’), 113.5 (C3’’), 115.3 (C3’’’, C5’’’), 115.4 ($CH\underline{C}HCOO$), 115.9 (C5a’’), 116.7 (C5’), 122.8 (CH_{Trz}), 127.5 ($\underline{C}CHCHCOO$), 128.8 (C5’’), 130.0 (C2’’’, C6’’’), 132.6 (C7a’), 136.3 (C6’), 143.3 (C4’’), 143.8 ($\underline{C}N_{Trz}$), 145.4 ($\underline{C}HCHCOO$), 146.9 (C4’), 154.3 (C10a’’), 156.5 (C9a’’), 160.3 ($CH_2O\underline{C}$), 161.4 (C2’’), 166.7 (COO), 167.6 (C1’), 168.5 (C2), 169.6 (C3’), 171.1 (C6). **HPLC (t$_R$, min):** 13.52. **MS (ESI, *m/z*, %):** 815.3 ($[M+H]^+$, 100). HRMS (ESI) m/z: calcd for $C_{44}H_{42}N_6O_{10}{}^+[M+H]^+$, 815.3040; found, 815.3001.

2.10. Synthesis of PROTAC 10

***Tert*-butyl 6-{4-[2,6-bis(benzyloxy)pyridin-3-yl]phenoxy}hexanoate (45).** To 4-[2,6-bis(benzyloxy)pyridin-3-yl]phenol (1.0 eq, 200 mg, 0.522 mmol), synthesized as previously described,[37] and potassium carbonate (1.5 eq, 108 mg, 0.782 mmol) in DMF (5.2 mL) was added *tert*-butyl 6-bromohexanoate (1.5 eq, 196 mg, 0.782 mmol) and the reaction mixture was stirred for 30 min at 150 ºC under MW conditions. Then, water was added to the reaction mixture and the aqueous layer was extracted with EtOAc (x3). The organic layer was washed with brine, dried over anhydrous Na_2SO_4 and concentrated under reduced pressure. The crude was purified by flash chromatography (hex to hex/EtOAc, 85:15) to afford compound **45** as an oil (170 mg, 0.307 mmol, 59% yield).

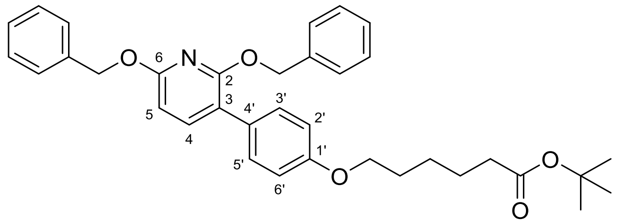

R$_f$: 0.50 (Hex/EtOAc, 8:2). **IR (ATR, cm^{-1})**: ν 3031 (=C-H), 2975 (C-H), 2937 (C-H), 1724 (C=O), 1242 (C-O), 1148 (C-O). **^{1}H-NMR (300 MHz, $CDCl_3$):** δ 1.45 (s, 9H, $(CH_3)_3$), 1.47 – 1.57 (m, 2H, $C\underline{H}_2CH_2CH_2COO$), 1.68 (quint, *J* = 7.2 Hz, 2H, $C\underline{H}_2CH_2COO$), 1.82 (quint, *J* = 6.6 Hz, 2H, $C\underline{H}_2CH_2OAr$), 2.26 (t, *J* = 7.4 Hz, 2H, CH_2COO), 3.99 (t, *J* = 6.4 Hz, 2H, $C\underline{H}_2O$), 5.36 (s, 2H, CH_2OAr), 5.42 (s, 2H, CH_2OAr), 6.46 (dd, *J* = 8.0, 0.7 Hz, 1H, H_5), 6.86 – 6.98 (m, 2H, $H_{2'}$, $H_{6'}$), 7.24 – 7.53 (m, 12H, $10CH_{Ph}$, $H_{3'}$, $H_{5'}$), 7.57 (d, *J* = 8.0 Hz, 1H, H_4). **^{13}C-NMR (75 MHz, $CDCl_3$):** δ 25.0 ($\underline{C}H_2CH_2COO$), 25.8 ($\underline{C}H_2CH_2CH_2COO$), 28.3 ($(\underline{C}H_3)_3$), 29.2 ($\underline{C}H_2CH_2OAr$), 35.6 ($\underline{C}H_2COO$), 67.7 ($CH_2OAr$), 67.8 ($CH_2OAr$), 68.0 ($CH_2O$), 80.2 ($\underline{C}(CH_3)_3$), 102.4 (C5), 114.3 (C2', C6'), 115.9 (C3), 127.4 (2C), 127.5 (2C), 127.9 (2C), 128.5 (2C), 128.6 (2C) ($10CH_{Ph}$), 129.2 (C4'), 130.2 (C3', C5'), 137.8 (C_{Ph}), 138.1 (C_{Ph}), 141.6 (C4), 158.1 (C1'), 158.3 (C2), 161.0 (C6), 173.2 (COO).

***Tert*-butyl 6-[4-(2,6-dioxopiperidin-3-yl)phenoxy]hexanoate (46).** Compound **45** (120 mg, 0.217 mmol) was dissolved in EtOH and hydrogenated using a Thales Nano H-Cube® flux reactor at 1 mL/min, full H_2 and 60 ºC. The solvent was evaporated under reduced pressure to obtain compound **46** as a solid (75.0 mg, 0.200 mmol, 92% yield).

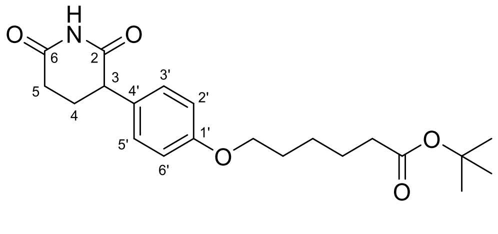

Mp: 75-77 ºC. **R$_f$**: 0.64 (Hex/EtOAc, 4:6). **IR (ATR, cm^{-1})**: ν 3219 (N-H), 2977 (C-H), 2936 (C-H), 1726 (C=O), 1238 (C-O), 1179 (C-O), 1156 (C-O). **^{1}H-NMR (300 MHz, $CDCl_3$):** δ 1.44 (s, 9H, $(CH_3)_3$), 1.46 – 1.53 (m, 2H, $C\underline{H}_2CH_2CH_2OAr$), 1.60 – 1.71 (m, 2H, $C\underline{H}_2CH_2COO$), 1.79 (m, 2H, $C\underline{H}_2CH_2OAr$), 2.13 – 2.35 (m, 4H, CH_2COO, H_4), 2.54 – 2.81 (m, 2H, H_5), 3.73 (dd, *J* = 9.3, 5.4 Hz, 1H, H_3), 3.94 (t, *J* = 6.4 Hz, 2H, $C\underline{H}_2OAr$), 6.88 (d, *J* = 8.7 Hz, 2H, $H_{2'}$, $H_{6'}$), 7.11 (d, *J* = 8.6 Hz, 2H, $H_{3'}$, $H_{5'}$), 8.05 (s, 1H, NHCO). **^{13}C-NMR (75 MHz, $CDCl_3$):** δ 25.0 ($\underline{C}H_2CH_2COO$), 25.7 ($\underline{C}H_2CH_2CH_2OAr$), 26.5 (C4), 28.3 ($(CH_3)_3$), 29.1 ($\underline{C}H_2CH_2OAr$), 31.0 (C5), 35.6 ($\underline{C}H_2COO$), 47.3 (C3), 67.9 ($\underline{C}H_2OAr$), 80.2 ($\underline{C}(CH_3)_3$), 115.0 (2C, C2' and C6'), 128.9 (C4'), 129.2 (2C, C3' and C5'), 158.7 (C1'), 172.4 (COO), 173.2 (C2), 173.5 (C6).

6-[4-(2,6-Dioxopiperidin-3-yl)phenoxy]hexanoic acid (47). Following general procedure B, *tert*-butyl ester **46** (60.0 mg, 0.160 mmol) was dissolved in DCM (0.80 mL) and TFA (1.2 mL, 16.0 mmol) was added. Compound **47** was obtained as a purple solid (50.0 mg, 0.157 mmol, 98% yield).

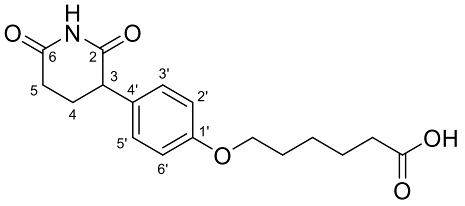

Mp: 137-139 °C. **R$_f$**: 0.36 (Hex/EtOAc, 4:6). **IR (ATR, cm^{-1})**: ν 3218 (N-H), 3098 (=C-H), 2936 (C-H), 2869 (C-H), 1700 (C=O), 1238 (C-O), 1194 (C-O). **^{1}H-NMR (300 MHz, Acetone-*d$_6$*):** δ 1.45 – 1.60 (m, 2H, $C\underline{H}_2CH_2CH_2OAr$), 1.60 – 1.73 (m, 2H, $C\underline{H}_2CH_2COO$), 1.73 – 1.92 (m, 2H, $C\underline{H}_2CH_2OAr$), 2.15 – 2.31 (m, 2H, H_4), 2.33 (t, *J* = 7.3 Hz, 2H, CH_2COO), 2.49 – 2.82 (m, 2H, H_5), 3.83 (dd, *J* = 10.0, 5.6 Hz, 1H, H_3), 3.99 (t, *J* = 6.4 Hz, 2H, $C\underline{H}_2OAr$), 6.90 (d, *J* = 8.6 Hz, 2H, $H_{2'}$, $H_{6'}$), 7.18 (d, *J* = 8.7 Hz, 2H, $H_{3'}$, $H_{5'}$), 9.56 (s, 1H, NHCO), 10.52 (s, 1H, COOH). **^{13}C-NMR (75 MHz, Acetone-*d$_6$*):** δ 25.4 ($\underline{C}H_2CH_2COO$), 26.4 ($\underline{C}H_2CH_2CH_2OAr$), 27.4 (C4), 29.7 ($\underline{C}H_2CH_2OAr$), 32.0 (C5), 34.1 ($\underline{C}H_2COO$), 47.9 (C3), 68.3 ($\underline{C}H_2OAr$), 115.2 (2C, C2' and C6'), 130.3 (2C, C3'and C5'), 131.7 (C4'), 159.2 (C1'), 173.4 (C2), 174.5 (C6), 174.6 (COO). **HPLC (t$_R$, min):** 10.66. **MS (ESI, *m/z*, %):** 317.1 ([M-2]$^{+\cdot}$, 100).

(7*S*)-8,8-Dimethyl-2-oxo-7,8-dihydro-2*H*,6*H*-benzo[1,2-*b*:5,4-*b'*]dipyran-7-yl 6-[4-(2,6-dioxopiperidin-3-yl)phenoxy]hexanoate (10). Following general procedure C, compound **47** (15.6 mg, 49.0 µmol), DCC (10.1 mg, 49.0 µmol) and DMAP (1.60 mg, 13.0 µmol) were dissolved in anhydrous DCM (0.16 mL). Decursinol (**1**) (8.00 mg, 32.0 µmol) was then added. The residue was purified by column chromatography (DCM to DCM/MeOH, 98:2) to obtain compound **10** as a solid (5.00 mg, 9.00 µmol, 28% yield).

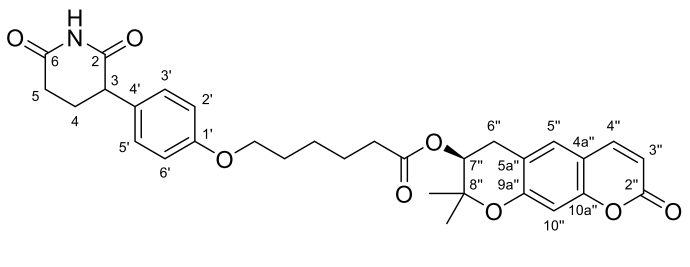

Mp: 98-100 °C. **R$_f$**: 0.70 (EtOAc). **IR (ATR, cm^{-1})**: ν 2928 (C-H), 1725, 1706 (C=O), 1625 (C=C), 1134 (C-O). **^{1}H-NMR (300 MHz, CDCl$_3$):** δ 1.35 (s, 3H, CH_3), 1.37 (s, 3H, CH_3), 1.45 (ddd, *J* = 9.5, 8.0, 4.4 Hz, 2H, $C\underline{H}_2CH_2CH_2OAr$), 1.57 – 1.82 (m, 4H, $C\underline{H}_2CH_2C\underline{H}_2CH_2OAr$), 2.17 – 2.30 (m, 2H, H_4), 2.35 (t, *J* = 7.3 Hz, 2H, CH_2COO), 2.56 – 2.78 (m, 2H, H_5), 2.84 (dd, *J* = 17.3, 4.8 Hz, 1H, 1$H_{6''}$), 3.18 (dd, *J* = 17.2, 4.7 Hz, 1H, 1$H_{6''}$), 3.73 (dd, *J* = 9.5, 5.5 Hz, 1H, H_3), 3.89 (t, *J* = 6.3 Hz, 2H, $C\underline{H}_2OAr$), 5.05 (t, *J* = 4.8 Hz, 1H, $H_{7''}$), 6.21 (d, *J* = 9.4 Hz, 1H, $H_{3''}$), 6.79 (s, 1H, $H_{10''}$), 6.85 (d, *J* = 8.6 Hz, 2H, $H_{2'}$, $H_{6'}$), 7.11 (d, *J* = 8.6 Hz, 2H, $H_{3'}$, $H_{5'}$), 7.13 (s, 1H, $H_{5''}$), 7.54 (d, *J* = 9.5 Hz, 1H,

$H_{4''}$), 7.99 (s, 1H, NHCO). **^{13}C-NMR (75 MHz, CDCl$_3$):** δ 23.3 (CH_3), 24.8 (<u>C</u>H_2CH_2COO), 25.1 (CH_3), 25.7 (<u>C</u>$H_2CH_2CH_2$OAr), 26.5 (C4), 27.9 (C6''), 29.0 (<u>C</u>H_2CH_2OAr), 31.1 (C5), 34.3 (<u>C</u>H_2COO), 47.3 (C3), 67.7 (<u>C</u>H_2OAr), 70.2 (C7''), 77.4 (C8''), 104.9 (C10''), 113.0 (C4a''), 113.5 (C3''), 115.0 (C2', C6'), 115.7 (C5a''), 128.8 (C5''), 129.0 (C4'), 129.2 (C3', C5'), 143.2 (C4''), 154.3 (C10a''), 156.5 (C9a''), 158.6 (C1'), 161.4 (C2''), 172.4 (COO), 173.1 (C2), 173.5 (C6). **HPLC (t_R, min):** 13.33. **MS (ESI, *m/z*, %):** 548.3 ([M+H]$^+$, 45). HRMS (ESI) *m/z* calcd for $C_{31}H_{33}NO_8^+$: 548.2284 [M+H]$^+$; found, 548.2268.

2.11. Synthesis of PROTAC 11

***Tert*-butyl (7*S*)-8,8-dimethyl-2-oxo-7,8-dihydro-2*H*,6*H*-benzo[1,2-*b*:5,4-*b'*]dipyran-7-yl hexanedioate (48).** Following general procedure C, 6-*tert*-butoxy-6-oxohexanoic acid (1.1 eq, 27.1 mg, 0.134 mmol), DCC (1.1 eq, 27.6 mg, 0.134 mmol) and DMAP (6.00 mg, 49.0 μmol) were dissolved in anhydrous DCM (1.2 mL). Decursinol (**1**) (1.0 eq, 30.0 mg, 0.122 mmol) was then added. The residue was purified by flash chromatography (hex/EtOAc, 1:1) to obtain compound **48** as an off-white solid (30.0 mg, 70.0 μmol, 57% yield).

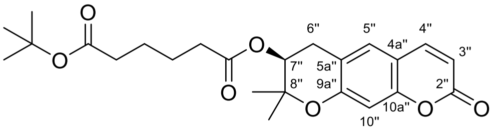

Mp: 99-101 °C. **R$_f$**: 0.57 (Hex/EtOAc, 1:1). **IR (ATR, cm^{-1})**: ν 2978 (C-H), 2933 (C-H), 1728 (C=O), 1626 (C=C), 1135 (C-O). **^{1}H-NMR (300 MHz, CDCl$_3$):** δ 1.35 (s, 3H, CH_3), 1.36 (s, 3H, CH_3), 1.42 (s, 9H, $(CH_3)_3$), 1.55 – 1.64 (m, 4H, C<u>H$_2$</u>C<u>H$_2$</u>CH_2COO), 2.19 (t, *J* = 6.9 Hz, 2H, C<u>H$_2$</u>COO), 2.33 (t, *J* = 7.0 Hz, 2H, C<u>H$_2$</u>COO), 2.84 (dd, *J* = 17.1, 4.9 Hz, 1H, 1$H_{6''}$), 3.18 (ddd, *J* = 17.1, 4.9, 1.2 Hz, 1H, 1$H_{6''}$), 5.05 (t, *J* = 4.9 Hz, 1H, $H_{7''}$), 6.23 (d, *J* = 9.5 Hz, 1H, $H_{3''}$), 6.79 (s, 1H, $H_{10''}$), 7.15 (d, *J* = 1.1 Hz, 1H, $H_{5''}$), 7.58 (d, *J* = 9.6 Hz, 1H, $H_{4''}$). **^{13}C-NMR (75 MHz, CDCl$_3$):** δ 23.2 (CH_3), 24.4, 24.6 (2<u>C</u>H_2CH_2COO), 25.1 (CH_3), 27.9 (C6''), 28.2 (C(<u>C</u>H_3)$_3$), 34.1 (<u>C</u>H_2COO), 35.2 (<u>C</u>H_2COO), 70.3 (C7''), 76.6 (C8''), 80.4 (<u>C</u>(CH_3)$_3$), 104.9 (C10''), 113.0 (C4a''), 113.5 (C3''), 115.8 (C5a''), 128.8 (C5''), 143.3 (C4''), 154.3 (C10a''), 156.5 (C9a''), 161.4 (C2''), 172.7 (COO), 172.9 (COO). **HPLC (t_R, min):** 14.99. **MS (ESI, *m/z*, %):** 375.2 ([M+H-^{t}Bu]$^+$, 100).

6-{[(7*S*)-8,8-Dimethyl-2-oxo-7,8-dihydro-2*H*,6*H*-benzo[1,2-*b*:5,4-*b'*]dipyran-7-yl]-oxy}-6-oxohexanoic acid (49). Following general procedure B, *tert*-butyl ester **48** (30.0 mg, 70.0 μmol) was dissolved in DCM (0.70 mL) and TFA (0.53 mL, 6.97 mmol) was added. Compound **49** was obtained as an off-white solid (22.0 mg, 59.0 μmol, 84% yield).

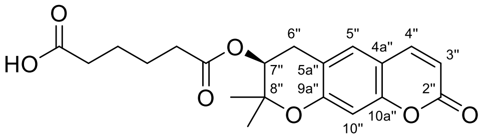

Mp: 132-134 °C. **R_f**: 0.17 (Hex/EtOAc, 1:1). **IR (ATR, cm^{-1})**: ν 3060 (O-H), 2930 (C-H), 1728 (C=O), 1707 (C=O), 1625 (C=C), 1133 (C-O). **^{1}H-NMR (300 MHz, $CDCl_3$):** δ 1.35 (s, 3H, CH_3), 1.37 (s, 3H, CH_3), 1.53 – 1.73 (m, 4H, C$\underline{H}_2$C$\underline{H}_2$$CH_2$COO), 2.28 – 2.42 (m, 4H, 2C$\underline{H}_2$COO), 2.84 (dd, J = 17.3, 4.9 Hz, 1H, $1H_{6''}$), 3.18 (ddd, J = 17.2, 4.8, 1.2 Hz, 1H, $1H_{6''}$), 5.05 (t, J = 4.8 Hz, 1H, $H_{7''}$), 6.23 (d, J = 9.4 Hz, 1H, $H_{3''}$), 6.79 (s, 1H, $H_{10''}$), 7.15 (s, 1H, $H_{5''}$), 7.58 (d, J = 9.5 Hz, 1H, $H_{4''}$). **^{13}C-NMR (75 MHz, $CDCl_3$):** δ 23.3 (CH_3), 24.1, 24.4 (2$\underline{C}H_2CH_2$COO), 25.1 (CH_3), 27.9 (C6''), 33.4 ($\underline{C}H_2$COO), 34.0 ($\underline{C}H_2$COO), 70.4 (C7''), 76.6 (C8''), 104.9 (C10''), 113.0 (C4a''), 113.5 (C3''), 115.7 (C5a''), 128.8 (C5''), 143.3 (C4''), 154.4 (C10a''), 156.5 (C9a''), 161.4 (C2''), 172.8 ($COO_{Decursinol}$), 177.9 (COOH). **HPLC (t_R, min):** 12.01. **MS (ESI, *m/z*, %):** 375.2 ([M+H]$^+$, 100).

***N*-(6-{[(7*S*)-8,8-Dimethyl-2-oxo-7,8-dihydro-2*H*,6*H*-benzo[1,2-*b*:5,4-*b'*]dipyran-7-yl]oxy}-6-oxohexanoyl)-3-methyl-L-valyl-(4*R*)-4-hydroxy-*N*-{[4-(4-methyl-1,3-thiazol-5-yl)phenyl]methyl}-L-prolinamide (11).** To a solution of **49** (1.0 eq, 13.0 mg, 35.0 μmol) in DCM (0.70 mL), HATU (1.1 eq, 14.5 mg, 38.0 μmol) was added and the resulting solution was stirred for 10 minutes at rt. Then, ((2*S*,4*R*)-1-[(2*S*)-2-amino-3,3-dimethyl-butanoyl]-4-hydroxy-*N*-[[4-(4-methylthiazol-5-yl)phenyl]methyl]pyrrolidine-2-carboxamide hydrochloride (1.1 eq, 17.8 mg, 38.0 μmol) and DIPEA (3.0 eq, 18 μL, 0.104 mmol) were added. The resulting mixture was stirred at rt for 12 h. The product was washed with water (x2) and then purified by column chromatography (DCM/MeOH, 95:5) to obtain compound **11** as a white solid (12.0 mg, 15.0 μmol, 44% yield).

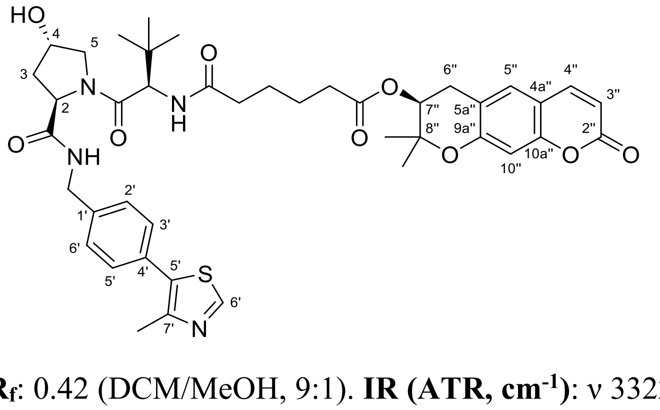

Mp: 130-132 °C. **R_f**: 0.42 (DCM/MeOH, 9:1). **IR (ATR, cm^{-1})**: ν 3322 (O-H), 2950 (C-H), 1730 (C=O), 1626 (C=C), 1136 (C-O). **^{1}H-NMR (300 MHz, $CDCl_3$):** δ 0.91 (s, 9H, $(CH_3)_3$), 1.33 (s, 3H, CH_3), 1.35 (s, 3H, CH_3), 1.53 – 1.64 (m, 4H, 2C$\underline{H}_2$$CH_2$COO), 2.06 – 2.15 (m, 1H, $1H_3$), 2.13 – 2.20 (m, 2H, C$\underline{H}_2$CONH), 2.31 (d, J = 5.6 Hz, 2H, C$\underline{H}_2$COO), 2.52 (s, 3H, $CH_3C_{Thiazole}$), 2.53 – 2.63 (m, 1H, $1H_3$), 2.83 (dd, J = 17.2, 4.8 Hz, 1H, $1H_{6''}$), 3.16 (dd, J = 17.2, 4.8 Hz, 1H, $1H_{6''}$), 3.60 (ddd, J = 11.3, 3.7, 1.7 Hz, 1H, $1H_5$), 4.07 (d, J = 11.4 Hz, 1H, $1H_5$), 4.33 (dd, J = 14.9, 5.2 Hz, 1H, C$\underline{H}_2$NHCO), 4.48 (d, J = 8.7, Hz,

1H, C$\underline{H}^t$Bu), 4.54 (m, 1H, H_4), 4.58 (dd, J = 14.7, 5.0 Hz, 1H, C$\underline{H}_2$NHCO), 4.72 (td, J = 8.0, 2.3 Hz, 1H, H_2), 5.03 (t, J = 4.8 Hz, 1H, $H_{7''}$), 6.09 (d, J = 8.1 Hz, 1H, NH), 6.22 (d, J = 9.5 Hz, 1H, $H_{3''}$), 6.78 (s, 1H, $H_{10''}$), 7.14 (s, 1H, $H_{5''}$), 7.24 – 7.33 (m, 1H, NH), 7.29 – 7.41 (m, 4H, $H_{2'}$, $H_{3'}$, $H_{5'}$, $H_{6'}$), 7.57 (d, J = 9.5 Hz, 1H, $H_{4''}$), 8.70 (s, 1H, $H_{6'}$). **^{13}C-NMR (75 MHz, $CDCl_3$):** δ 16.1 ($\underline{C}H_3C_{Thiazole}$), 23.3 ($CH_3$), 24.4 ($\underline{C}H_2CH_2COO$), 24.9 ($\underline{C}H_2CH_2CONH$), 25.1 ($CH_3$), 26.5 ($(CH_3)_3$), 27.9 (C6''), 34.0 ($\underline{C}H_2COO$), 34.9 ($\underline{C}(CH_3)_3$), 35.9 (C3), 35.9 ($C\underline{H}_2CONH$), 43.4 ($\underline{C}H_2NHCO$), 56.8 (C5), 57.7 ($\underline{C}H^tBu$), 58.5 (C2), 70.2 (C7''), 70.4 (C4), 76.6 (C8''), 104.9 (C10''), 113.0 (C4a''), 113.5 (C3''), 115.7 (C5a''), 128.3 (C3', C5'), 128.8 (C5''), 129.7 (C2', C6'), 131.0 (C4'), 131.6 (C5'), 138.3 (C1'), 143.3 (C4''), 148.4 (C7'), 150.5 (C6'), 154.3 (C10a''), 156.4 (C9a''), 161.4 (C2''), 170.7 ($\underline{C}OCH^tBu$), 172.1 ($CH_2NH\underline{C}O$), 173.0 (COO), 173.2 ($NHCO_{linker}$). **HPLC (t_R, min):** 12.07. **MS (ESI, *m/z*, %):** 787.4 ([M+H]$^+$, 100). HRMS (ESI) *m/z* calcd for $C_{42}H_{50}N_4O_9S^+$: 787.3376 [M+H]$^+$; found, 787.3332.

2.12. Synthesis of PROTAC 12

2-Chloro-1-(6-hydroxy-3,4-dihydroquinolin-1(2*H*)-yl)ethan-1-one (50). 6-Hydroxy-1,2,3,4-tetrahydroquinoline (1.0 eq, 500 mg, 3.35 mmol) and NaOH (1.2 eq, 179 mg, 4.02 mmol) were dissolved in water (5.0 mL) and dioxane (5.0 mL) at 0 ºC. Chloroacetyl chloride (1.1 eq, 416 mg, 3.69 mmol) was added dropwise over 5 minutes and the reaction was stirred at rt for 4 h. The reaction mixture was acidified with 1M HCl (until pH <4) and extracted with EtOAc (x3). The organic phase was washed with brine, dried over anhydrous Na_2SO_4, filtered, and concentrated under reduced pressure. The resulting residue was purified by flash chromatography (DCM to DCM/MeOH, 96:4) to obtain compound **50** as a solid (300 mg, 1.33 mmol, 40% yield).

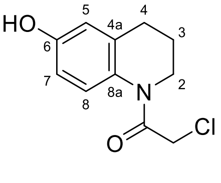

Mp: 150-152 ºC. **R$_f$**: 0.45 (DCM/MeOH, 9:1). **IR (ATR, cm^{-1})**: ν 3279 (O-H), 3032 (=C-H), 2949 (C-H), 1628 (C=O), 1499 (C=C). **^{1}H-NMR (300 MHz, Acetone-*d$_6$*):** δ 1.93 (t, J = 6.7 Hz, 2H, H_3), 2.66 (d, J = 7.0 Hz, 2H, H_4), 3.73 (t, J = 6.5 Hz, 2H, H_2), 4.33 (s, 2H, $C\underline{H}_2Cl$), 6.69 (d, J = 6.5 Hz, 2H, H_7, H_8), 7.20 (s, 1H, H_5). **^{13}C-NMR (75 MHz, Acetone-*d$_6$*):** δ 24.5 (C3), 27.2 (C4), 43.0 (CH_2Cl), 43.7 (C2), 113.8 (C7), 115.7 (C8), 125.8 (C5), 131.5 (C8a), 136.8 (C4a),156.2 (C6), 166.1 ($\underline{C}OCH_2Cl$). **HPLC (t_R, min):** 10.66. **MS (ESI, *m/z*, %):** 226.1 ([M+H]$^+$, 100).

1-(Chloroacetyl)-1,2,3,4-tetrahydroquinolin-6-yl (7*S*)-8,8-dimethyl-2-oxo-7,8-dihydro-2*H*,6*H*-benzo[1,2-*b*:5,4-*b'*]dipyran-7-yl hexanedioate (12). Following general procedure C, compound **49** (18.0 mg, 48.0 μmol), DCC (9.90 mg, 48.0 μmol) and DMAP (2.30 mg, 19.0 μmol) were dissolved in anhydrous DCM (0.48 mL). DCAF16

binding ligand **50** (13.0 mg, 58.0 µmol) was then added. The residue was purified by column chromatography (DCM to DCM/MeOH, 98:2) to obtain compound **12** as an off-white solid (3.00 mg, 5.00 µmol, 11% yield).

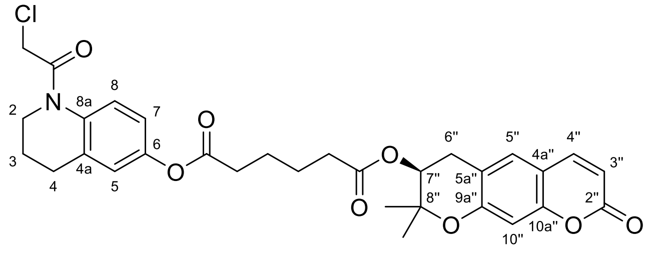

Mp: 121-123 °C. **R$_f$**: 0.77 (DCM/MeOH, 9:1). **IR (ATR, cm^{-1})**: ν 2925 (C-H), 1730 (C=O), 1626 (C=C), 1133 (C-O). **^{1}H-NMR (700 MHz, CDCl$_3$)** δ 1.35 (s, 3H, CH$_3$), 1.38 (s, 3H, CH$_3$), 1.71 – 1.75 (m, 4H, 2C<u>H</u>$_2$CH$_2$COO), 1.99 – 2.02 (m, 2H, H$_3$), 2.36 – 2.40 (m, 2H, CH$_2$COO), 2.52 – 2.55 (m, 2H, CH$_2$COO), 2.73 – 2.75 (m, 2H, H$_4$), 2.86 (dd, *J* = 17.1, 4.7 Hz, 1H, 1H$_{6''}$), 3.19 (dd, *J* = 16.9, 4.7 Hz, 1H, 1H$_{6''}$), 3.81 – 3.83 (m, 2H, H$_2$), 4.21 (s, 2H, CH$_2$Cl), 5.07 (t, *J* = 4.8 Hz, 1H, H$_{7''}$), 6.23 (d, *J* = 9.4 Hz, 1H, H$_{3''}$), 6.80 (s, 1H, H$_{10''}$), 6.92 (s, 2H, H$_7$, H$_8$), 7.15 (s, 1H, H$_{5''}$), 7.24 (s, 1H, H$_5$), 7.57 (d, *J* = 9.1 Hz, 1H, H$_{4''}$). **^{13}C-NMR (176 MHz, CDCl$_3$)** δ 23.3 (CH$_3$), 23.5 (C3), 24.2 (2<u>C</u>H$_2$CH$_2$COO), 24.9 (CH$_3$), 26.8 (C4), 27.8 (C6''), 33.8, 33.9 (2<u>C</u>H$_2$COO), 41.5 (CH$_2$Cl), 43.3 (C2), 70.2 (C7''), 76.5 (C8''), 104.8 (C10''), 112.9 (C4a''), 113.4 (C3''), 115.5 (C5a''), 119.6, 121.7 (C7, C8), 125.8 (C5), 128.7 (C5''), 131.5 (C8a), 136.8 (C4a), 143.2 (C4''), 154.2 (C10a''), 156.3 (C9a'', C6), 161.3 (C2''), 165.9 (ClCH$_2$<u>C</u>O), 171.7, 172.7 (2COO). **HPLC (t$_R$, min):** 14.05. **MS (ESI, *m/z*, %):** 582.1 ([M+H]$^+$, 100). HRMS (ESI) *m/z* calcd for $C_{31}H_{32}ClNO_8+Na^+$: 604.1714 [M+Na]$^+$; found, 604.1699.

2.13. Synthesis of CRBN-based negative control 13

4-Fluoro-2-(1-methyl-2,6-dioxopiperidin-3-yl)-1*H*-isoindole-1,3(2*H*)-dione (51). To a round bottom flask containing 4-fluoro-thalidomide (1.0 eq, 200 mg, 0.724 mmol) in anhydrous DMF (1.4 mL), methyl iodide (1.3 eq, 56 µL, 0.905 mmol) and K_2CO_3 (1.3 eq, 125 mg, 0.905 mmol) were added. The suspension was stirred at rt for 12 h. The resulting mixture was diluted with water to precipitate the product, which was filtered and washed with water (x3) to obtain compound **51** as a solid (155 mg, 0.534 mmol, 89% yield).

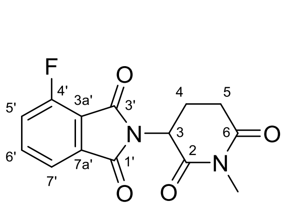

Mp: 194-196 °C. **R$_f$**: 0.80 (DCM/MeOH, 95:5). **IR (ATR, cm^{-1})**: ν 2959 (C-H), 2921 (C-H), 1715, 1679 (C=O). **^{1}H-NMR (300 MHz, CDCl$_3$):** δ 2.15 – 2.32 (m, 1H, H$_4$), 2.65 – 2.83 (m, 1H, H$_4$), 2.86 – 3.07 (m, 2H, H$_5$), 3.10 (s, 3H, CH$_3$), 5.18 (dd, *J* = 13.0, 5.4 Hz, 1H, H$_3$), 7.64 (ddd, *J* = 9.2, 8.4, 0.7 Hz, 1H H$_{5'}$), 7.77 (dt, *J* = 7.4, 0.7 Hz, 1H, H$_{7'}$), 7.97

(ddd, *J* = 8.4, 7.4, 4.5 Hz, 1H, $H_{6'}$). **^{13}C-NMR (75 MHz, $CDCl_3$):** δ 22.4 (C4), 27.1 (CH_3), 32.2 (C5), 51.0 (C3), 118.5 (d, *J* = 12.6 Hz, C3a'), 120.6 (d, *J* = 3.8 Hz, C7'), 123.5 (d, *J* = 19.9 Hz, C5'), 135.1 (d, *J* = 1.7 Hz, C7a'), 138.5 (d, *J* = 7.9 Hz, C6'), 158.4 (d, *J* = 263.2 Hz, C4'), 164.8 (d, *J* = 1.2 Hz, C3'), 167.0 (d, *J* = 3.0 Hz, C1'), 170.0 (C2), 172.1 (C6). **HPLC (t_R, min):** 11.64. **MS (ESI, *m/z*, %):** 291.1 ([M+H]$^+$, 100). The spectroscopic data are consistent with those reported in the bibliography.[62]

***Tert*-butyl 6-{[2-(1-Methyl-2,6-dioxopiperidin-3-yl)-1,3-dioxo-2,3-dihydro-1*H*-isoindol-4-yl]amino}hexanoate (52).** Following general procedure A, compound **51** (60.0 mg, 0.207 mmol) was dissolved in DMF (0.51 mL) and DIPEA (72 µL, 0.413 mmol) and *tert*-butyl 6-aminohexanoate (46.5 mg, 0.248 mmol) were added. The resulting residue was purified by flash chromatography (hex to hex/EtOAc, 4:6) to obtain compound **52** as a solid (35.0 mg, 76.0 µmol, 37% yield).

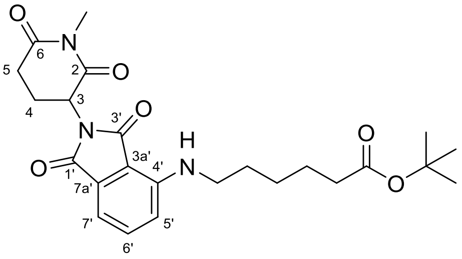

Mp: 70-72 ºC. **R_f**: 0.40 (Hex/EtOAc, 3:2). **IR (ATR, cm^{-1})**: ν 3396 (N-H), 2933 (C-H), 1721, 1682 (C=O), 1624 (C=C), 1118 (C-O). **^{1}H-NMR (300 MHz, $CDCl_3$):** δ 1.43 (s, 9H, $(CH_3)_3$), 1.44 – 1.51 (m, 2H, C$\underline{H}_2$CH$_2$CH$_2$NH), 1.66 (m, 4H, C$\underline{H}_2$CH$_2$C$\underline{H}_2$CH$_2$NH), 2.00 – 2.17 (m, 1H, 1H_4), 2.23 (t, *J* = 7.4 Hz, 2H, C$\underline{H}_2$COO), 2.69 – 2.85 (m, 2H, 1H_4, 1H_5), 2.91 – 3.04 (m, 1H, 1H_5), 3.21 (s, 3H, NCH_3), 3.26 (q, *J* = 6.7 Hz, 2H, C$\underline{H}_2$NH), 4.83 – 4.98 (m, 1H, H_3), 6.22 (br s, 1H, ArN$\underline{H}$), 6.87 (d, *J* = 8.5 Hz, 1H, $H_{5'}$), 7.08 (d, *J* = 7.1 Hz, 1H, $H_{7'}$), 7.48 (dd, *J* = 8.6, 7.1 Hz, 1H, $H_{6'}$). **^{13}C-NMR (75 MHz, $CDCl_3$):** δ 22.3 (C4), 24.8 ($\underline{C}$H$_2$CH$_2$CH$_2$CH$_2$NH), 26.5 ($\underline{C}$H$_2$CH$_2$CH$_2$NH), 27.4 (NCH_3), 28.2 (($CH_3)_3$), 29.1 ($\underline{C}$H$_2$CH$_2$NH), 32.1 (C5), 35.5 ($\underline{C}$H$_2$COO), 42.6 (CH_2NH), 49.7 (C3), 80.3 ($\underline{C}(CH_3)_3$), 110.1 (C3a'), 111.5 (C7'), 116.7 (C5'), 132.7 (7a'), 136.2 (C6'), 147.0 (C4'), 167.9 (C3'), 169.1 (C1'), 169.8 (C2), 171.4 (C6), 173.0 (COO). **HPLC (t_R, min):** 14.88. **MS (ESI, *m/z*, %):** 402.2 ([M+H-^{t}Bu]$^+$, 100). The spectroscopic data are consistent with those reported in the bibliography.[63]

6-{[2-(1-Methyl-2,6-dioxopiperidin-3-yl)-1,3-dioxo-2,3-dihydro-1*H*-isoindol-4-yl]-amino}hexanoic acid (53). Following general procedure B, *tert*-butyl ester **52** (18.0 mg, 39.0 µmol) was dissolved in DCM (0.39 mL) and TFA (0.30 mL, 3.93 mmol) was added. Compound **53** was obtained as a solid (15.0 mg, 37.0 µmol, 95% yield).

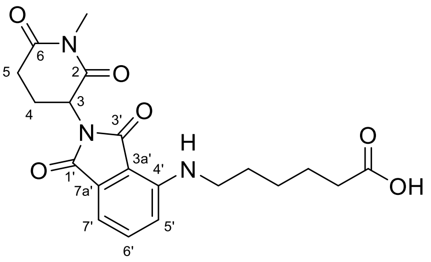

Mp: 121-123 °C. **R$_f$**: 0.11 (Hex/EtOAc, 3:2). **IR (ATR, cm^{-1})**: ν 3395 (N-H), 2930 (C-H), 2858 (C-H), 1728 (C=O), 1678 (C=O), 1623 (C=C), 1116 (C-O). **^{1}H-NMR (300 MHz, CDCl$_3$):** δ 1.39 – 1.45 (m, 2H, $C\underline{H}_2CH_2CH_2NH$), 1.60 – 1.66 (m, 4H, $C\underline{H}_2CH_2C\underline{H}_2CH_2NH$), 1.97 – 2.08 (m, 1H, 1H$_4$), 2.32 (t, *J* = 7.3 Hz, 2H, $C\underline{H}_2COO$), 2.64 – 2.79 (m, 2H, 1H$_4$, 1H$_5$), 2.80 – 2.96 (m, 1H, 1H$_5$), 3.14 (s, 3H, CH$_3$), 3.21 (t, *J* = 7.0 Hz, 2H, $C\underline{H}_2NH$), 4.69 – 4.99 (m, 1H, H$_3$), 6.81 (d, *J* = 8.5 Hz, 1H, H$_{5'}$), 7.02 (d, *J* = 7.0 Hz, 1H, H$_{7'}$), 7.42 (dd, *J* = 8.5, 7.1 Hz, 1H, H$_{6'}$). **^{13}C-NMR (75 MHz, CDCl$_3$):** δ 22.1 (C4), 24.3 ($\underline{C}H_2CH_2CH_2CH_2NH$), 26.3 ($\underline{C}H_2CH_2CH_2NH$), 27.3 (CH$_3$), 28.9 ($\underline{C}H_2CH_2NH$), 31.9 (C5), 33.6 ($\underline{C}H_2COO$), 42.4 (CH$_2$NH), 49.6 (C3), 110.1 (C3a’), 111.5 (C7’), 116.6 (C5’), 132.6 (7a’), 136.1 (C6’), 146.9 (C4’), 167.8 (C3’), 169.1 (C1’), 169.7 (C2), 171.3 (C6), 178.2 (COO). **HPLC (t$_R$, min):** 11.74. **MS (ESI, *m/z*, %):** 402.1 ([M+H]$^+$, 100).

(7*S*)-8,8-Dimethyl-2-oxo-7,8-dihydro-2*H*,6*H*-benzo[1,2-*b*:5,4-*b'*]dipyran-7-yl 6-{[2-(1-methyl-2,6-dioxopiperidin-3-yl)-1,3-dioxo-2,3-dihydro-1*H*-isoindol-4-yl]amino}-hexanoate (13). Following general procedure C, compound **53** (12.0 mg, 30.0 µmol), DCC (6.80 mg, 33.0 µmol) and DMAP (1.50 mg, 12.0 µmol) were dissolved in anhydrous DCM (0.30 mL). Decursinol (**1**) (8.80 mg, 36.0 µmol) was then added. The residue was purified by column chromatography (DCM to DCM/MeOH, 98:2) to obtain compound **13** as a solid (9.50 mg, 15.0 µmol, 51% yield).

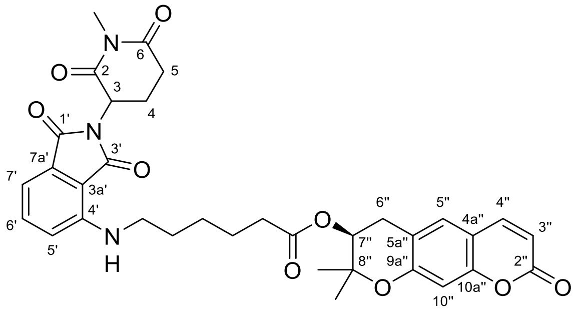

Mp: 106-108 °C. **R$_f$**: 0.83 (DCM/MeOH, 9:1). **IR (ATR, cm^{-1})**: ν 2921 (C-H), 1727, 1697, 1682 (C=O), 1625 (C=C), 1358 (C-H), 1134 (C-O). **^{1}H-NMR (300 MHz, CDCl$_3$):** δ 1.34 (s, 3H, CH$_3$), 1.37 (s, 3H, CH$_3$), 1.43 (dd, *J* = 9.3, 6.1 Hz, 2H, $C\underline{H}_2CH_2CH_2NH$), 1.64 (m, 4H, $C\underline{H}_2CH_2C\underline{H}_2CH_2NH$), 2.00 – 2.20 (m, 1H, 1H$_4$), 2.34 (t, *J* = 7.4 Hz, 2H, $C\underline{H}_2COO$), 2.68 – 2.89 (m, 3H, 1H$_{6''}$, 1H$_4$, 1H$_5$), 2.89 – 3.03 (m, 1H, 1H$_5$), 3.11 – 3.19 (m, 3H, 1H$_{6''}$, $C\underline{H}_2NH$), 3.20 (s, 3H, CH$_3$), 4.84 – 4.98 (m, 1H, H$_3$), 5.05 (t, *J* = 4.8 Hz, 1H, H$_{7''}$), 6.21 (d, *J* = 9.5 Hz, 1H, H$_{3''}$), 6.79 (s, 1H, H$_{10''}$), 6.83 (d, *J* = 8.5 Hz, 1H, H$_{5'}$),

7.08 (d, *J* = 7.1 Hz, 1H, $H_{7'}$), 7.14 (s, 1H, $H_{5''}$), 7.48 (dd, *J* = 8.5, 7.1 Hz, 1H, $H_{6'}$), 7.56 (d, *J* = 9.5 Hz, 1H, $H_{4''}$). **^{13}C-NMR (75 MHz, $CDCl_3$):** δ 22.3 (C4), 23.4 (CH_3), 24.7 ($\underline{C}H_2CH_2COO$), 25.0 (CH_3), 26.5 ($\underline{C}H_2CH_2CH_2COO$), 27.4 ($NCH_3$), 27.9 (C6''), 29.0 ($\underline{C}H_2CH_2NH$), 32.0 (C5), 34.2 ($\underline{C}H_2COO$), 42.5 ($\underline{C}H_2NH$), 49.8 (C3), 70.3 (C7''), 76.6 (C8''), 104.9 (C10''), 110.2 (C3a'), 111.6 (C7'), 113.0 (C4a''), 113.6 (C3''), 115.7 (C5a''), 116.6 (C5'), 128.7 (C5''), 132.7 (C7a'), 136.2 (C6'), 143.2 (C4''), 146.9 (C4'), 154.3 (C10a''), 156.4 (C9a''), 161.3 (C2''), 167.9 (C1'), 169.2 (C2), 169.8 (C3'), 171.4 (C6), 172.9 (COO). **HPLC (t_R, min):** 14.49. **MS (ESI, *m/z*, %):** 630.3 ($[M+H]^+$, 100). HRMS (ESI) *m/z* calcd for $C_{34}H_{35}N_3O_9+Na^+$: 652.2271 $[M+Na]^+$; found, 652.2251.

2.14. Synthesis of VHL-based negative control 14

4-[(5-Azidopentyl)amino]-2-(1-methyl-2,6-dioxopiperidin-3-yl)-1*H*-isoindole-1,3 (2*H*)-dione (54). Following general procedure A, **51** (60.0 mg, 0.207 mmol) was dissolved in DMF (1.0 mL) and DIPEA (0.11 mL, 0.620 mmol) and amine **21** (39.7 mg, 0.310 mmol) were added. The resulting residue was purified by flash chromatography (DCM/MeOH, 99:1) to obtain compound **54** as a solid (51.0 mg, 0.128 mmol, 62% yield).

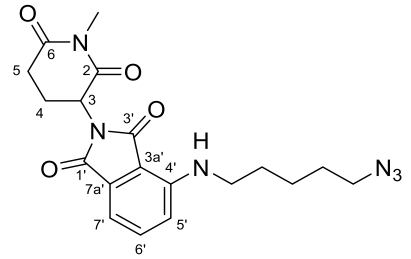

Mp: 103-105 °C. **R_f**: 0.61 (Hex/EtOAc, 4:6). **IR (ATR, cm^{-1})**: ν 3398 (N-H), 2931 (C-H), 2858 (C-H), 2097 (N=N), 1696 (C=O), 1682 (C=O), 1119 (C-O). **^{1}H-NMR (300 MHz, $CDCl_3$):** δ 1.44 – 1.59 (m, 2H, $NHCH_2CH_2C\underline{H}_2$), 1.59 – 1.84 (m, 4H, $NHCH_2C\underline{H}_2CH_2C\underline{H}_2$), 1.99 – 2.19 (m, 1H, $1H_4$), 2.65 – 2.86 (m, 2H, $1H_4$, $1H_5$), 2.86 – 3.04 (m, 1H, $1H_5$), 3.20 (s, 3H, CH_3), 3.25 – 3.36 (m, 4H, $NHC\underline{H}_2$, $C\underline{H}_2N_3$), 4.78 – 5.01 (m, 1H, H_3), 6.23 (t, *J* = 5.6 Hz, 1H, ArN$\underline{H}$), 6.87 (d, *J* = 8.5 Hz, 1H, $H_{5'}$), 7.08 (dd, *J* = 7.1, 0.6 Hz, 1H, $H_{7'}$), 7.49 (dd, *J* = 8.5, 7.1 Hz, 1H, $H_{6'}$). **^{13}C-NMR (75 MHz, $CDCl_3$):** δ 22.2 (C4), 24.3 ($NHCH_2CH_2\underline{C}H_2$), 27.4 ($CH_3$), 28.7 ($NHCH_2\underline{C}H_2$), 29.0 ($NHCH_2CH_2CH_2\underline{C}H_2$), 32.0 (C5), 42.6 ($CH_2NH$), 49.7 (C3), 51.3 ($CH_2N_3$), 110.2 (C3a'), 111.6 (C7'), 116.6 (C5'), 132.7 (C7a'), 136.2 (C6'), 146.9 (C4'), 167.8 (C3'), 169.1 (C1'), 169.8 (C2), 171.3 (C6). **HPLC (t_R, min):** 13.99. **MS (ESI, *m/z*, %):** 399.1 ($[M+H]^+$, 100).

(7*S*)-8,8-Dimethyl-2-oxo-7,8-dihydro-2*H*,6*H*-benzo[1,2-*b*:5,4-*b'*]dipyran-7-yl (2*E*)-3-(4-{[1-(5-{[2-(1-methyl-2,6-dioxopiperidin-3-yl)-1,3-dioxo-2,3-dihydro-1*H*-isoindol-4-yl]amino}pentyl)-1*H*-1,2,3-triazol-4-yl]methoxy}phenyl)prop-2-enoate (14). To a mixture of azide **55** (1.0 eq, 13.9 mg, 35.0 μmol), copper(II) sulfate (0.50 eq,

4.40 mg, 17.0 μmol) and sodium ascorbate (1.0 eq, 6.90 mg, 35.0 μmol) in DMF (0.14 mL) and water (0.38 mL) was added alkyne **22** synthesized as previously reported[35] (1.0 eq, 15.0 mg, 35.0 μmol) and the mixture was stirred at rt for 16 h. Then, the mixture was diluted with water and extracted with EtOAc (x3). The crude product was purified by column chromatography (DCM to DCM/MeOH, 98:2) to obtain compound **14** as a solid (18.0 mg, 22.0 μmol, 61% yield).

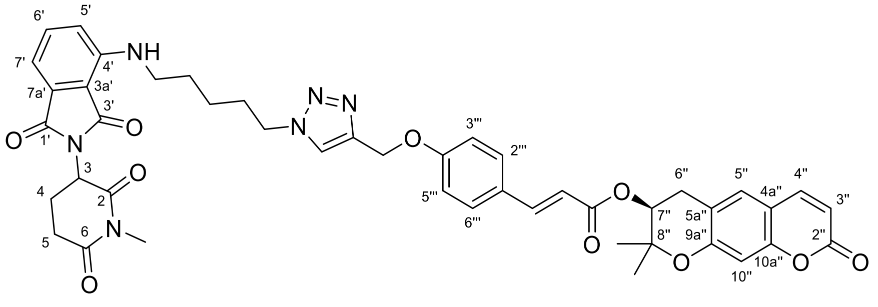

Mp: 134-135 °C. **R$_f$**: 0.61 (EtOAc). **IR (ATR, cm^{-1})**: ν 2925 (C-H), 1698, 1681 (C=O), 1626 (C=C), 1359 (C-H), 1161, 1134 (C-O). **^{1}H-NMR (300 MHz, CDCl$_3$)**: δ 1.38 (s, 3H, CH_3), 1.42 (s, 3H, CH_3), 1.44 – 1.53 (m, 2H, $NHCH_2CH_2C\underline{H}_2$), 1.70 (quint, *J* = 7.1 Hz, 2H, $NHCH_2C\underline{H}_2$), 1.98 (quint, *J* = 7.1 Hz, 2H, $C\underline{H}_2CH_2N_{Trz}$), 2.04 – 2.15 (m, 1H, $1H_4$), 2.68 – 2.83 (m, 2H, $1H_4$, $1H_5$), 2.87 – 3.02 (m, 2H, $1H_5$, $1H_{6''}$), 3.20 (s, 3H, NCH_3), 3.16 – 3.30 (m, 3H, $1H_{6''}$, $NHC\underline{H}_2$), 4.38 (t, *J* = 7.0 Hz, 2H, CH_2N_{Trz}), 4.83 – 4.96 (m, 1H, H_3), 5.15 – 5.20 (m, 1H, $H_{7''}$), 5.23 (s, 2H, CH_2O), 6.23 (d, *J* = 9.5 Hz, 1H, $H_{3''}$), 6.28 (d, *J* = 15.9 Hz, 1H, $C\underline{H}COO$), 6.82 (s, 1H, $H_{10''}$), 6.84 (d, *J* = 8.3 Hz, 1H, $H_{5'}$), 6.97 (d, *J* = 8.5 Hz, 2H, $H_{3'''}$, $H_{5'''}$), 7.09 (d, *J* = 7.0 Hz, 1H, $H_{7'}$), 7.16 (s, 1H, $H_{5''}$), 7.44 (d, *J* = 8.7 Hz, 2H, $H_{2'''}$, $H_{6'''}$), 7.53 – 7.47 (m, 1H, $H_{6'}$), 7.55 – 7.67 (m, 3H, CH_{Trz}, $H_{4''}$, $C\underline{H}CHCOO$). **^{13}C-NMR (75 MHz, CDCl$_3$):** δ 22.3 (C4), 23.5 (CH_3), 24.0 ($\underline{C}H_2CH_2CH_2N_{Trz}$), 25.1 ($CH_3$), 27.4 ($NCH_3$), 28.0 (C6’’), 28.8 ($C\underline{H}_2CH_2N_{Trz}$), 30.1 ($NHCH_2\underline{C}H_2$), 32.0 (C5), 42.4 ($CH_2NH$), 49.8 (C3), 50.3 ($CH_2N_{Trz}$), 62.2 ($CH_2O$), 70.2 (C7’’), 76.8 (C8’’), 104.9 (C10’’), 110.3 (C3a’), 111.7 (C7’), 113.0 (C4a’’), 113.5 (C3’’), 115.3 (2C, C3’’’ and C5’’’), 115.3 ($\underline{C}HCOO$), 115.9 (C5a’’), 116.6 (C5’), 122.8 (CH_{Trz}), 127.5 ($\underline{C}CHCHCOO$), 128.9 (C5’’), 130.1 (2C, C2’’’ and C6’’’), 132.7 (C7a’), 136.3 (C6’), 143.3 (C4’’), 143.8 ($\underline{C}N_{Trz}$), 145.4 ($\underline{C}HCHCOO$), 146.9 (C4’), 154.4 (C10a’’), 156.5 (C9a’’), 160.3 ($CH_2O\underline{C}$), 161.4 (C2’’), 166.7 (COO), 167.8 (C1’), 169.2 (C2), 169.8 (C3’), 171.3 (C6). **HPLC (t$_R$, min):** 14.41. **MS (ESI, *m/z*, %):** 829.3 ($[M+H]^+$, 100). HRMS (ESI) *m/z* calcd for $C_{45}H_{44}N_6O_{10}+Na^+$: 851.3017 $[M+Na]^+$; found, 851.2963.

3. Saturation Transfer Difference (STD) NMR

All NMR spectra were acquired at 298K using a Bruker AVANCE 600 MHz spectrometer equipped with a cryo-probe and processed with TopSpin 3.5 (Bruker BioSpin GmbH) and MestReNova 14.2 software (Mestrelab Research, SL). Progerin (50 µg, Diatheva, REP0050) was buffer exchanged by using a Millipore Amicon Ultra Centrifugal Filter to remove glycerol present in the buffer and to change the buffer to deuterated Tris 50mM, NaCl 50mM, pH 7.4. STD-NMR experiments were performed with a 10:1 ligand-protein molar ratio (30 μM protein and 300 μM ligand) and a saturation time of 2 s. The protein was saturated at -0.3 ppm (on-resonance experiment), and the off-resonance experiment

was saturated at 100 ppm. Relative STD effects were calculated by comparing the intensity of the signals in the STD-NMR spectrum with signal intensities of the reference spectrum (off-resonance). The STD signal with the highest intensity was set to 100%, and other STD signals were calculated accordingly. Control STD-NMR experiments were performed using an identical experimental setup and the same ligand concentration but in the absence of the protein.

4. Cell lines and culture.

Progeroid mouse fibroblasts, kindly donated by Prof. Carlos López Otín (Oviedo University, Spain), were isolated from *Lmna$^{G609G/G609G}$* mice[19] and wild-type (WT) littermates and immortalized by retroviral transduction with SV40 large T antigen (SV40-LT). Since *Lmna$^{G609G/G609G}$* mouse represents the most suitable and widely accepted model of HGPS, as it most faithfully recapitulates the molecular and phenotypic features of the human disease, its use in this study is fully justified. Moreover, although this cell model does not currently have an assigned Research Resource Identifier (RRID), these cells have been extensively validated by additional independent studies.[15, 65, 66] Accordingly, its use does not compromise the reproducibility or validity of the experiments, nor does it affect the conclusions presented in our study.

Mouse fibroblasts were grown in Dulbecco's modified eagle medium (DMEM, Gibco) supplemented with 10% heat inactivated fetal bovine serum (FBS, Gibco), 50 U/mL penicillin, and 50 μg/mL streptomycin (Invitrogen). Human progeroid HGADFN167 *Lmna$^{G608G/G608G}$* (G608G) or or healthy HGADFN168 *Lmna$^{+/+}$* (WT) fibroblasts, were obtained from The Progeria Research Foundation (RRIDs CVCL_1Y92 and CVCL_1Y93, respectively) and cultured in DMEM with 20% heat inactivated FBS, 50 U/mL penicillin and 50 μg/mL streptomycin. All cells were incubated in a humidified atmosphere at 37 °C in the presence of 5% CO_2 and were confirmed to be free of mycoplasma contamination.

5. Cell viability assay.

Cell viability was assessed as previously described.[64] Cells were seeded in 96-well plates at a density of 2 x 10^3 cells per well in the corresponding medium with 10% FBS for 24 h prior to treatments. The medium was then replaced by fresh medium containing different concentrations of compounds tested or by medium containing the equivalent volume of dimethylsulfoxide (DMSO, vehicle control). Cells were treated for 24 h, and then medium was replaced by fresh medium with 2 mg/mL of MTT (3-(4,5-dimethylthiazol-2-yl)-2,5-diphenyltetrazolium bromide, Sigma-Aldrich) and cells were incubated for 4 h at 37 ºC in the dark. Once supernatants were removed, formazan crystals previously formed by viable cells were dissolved in DMSO (100 μL/ well) and absorbance was measured at 570 nm (OD570-630) using an Asys UVM 340 (Biochrom Ltd., Cambridge, UK) microplate reader. Background absorbance from blank wells containing only media with compound or vehicle were subtracted from each test well. Measurements were performed in triplicate, and each experiment was repeated at least three times. The viability values were calculated from the percentage of viable cells with respect to 100% viability of the vehicle-treated cells.

6. Immunoblot and immunocytofluorescence analysis.

Mouse or human progeroid cells were seeded at density of 10^5 or 5 x 10^4 cells respectively on 12-well plates and incubated for 24 h in DMEM medium. For proteosome-dependency assays, cells were pre-treated either with the proteasome inhibitor (S)-MG132 (20 μM) or with the NEDD8-activating enzyme inhibitor MLN4924 (5 μM) for 2 h prior to

compound exposure. Then, cells were incubated with compound of interest or DMSO for the appropriate time and lysed with cold laemmli buffer (50 mM Tris-HCl pH 7.4, 150 mM NaCl, 1% Igepal) with a mixture of protease and phosphatase inhibitors (1 mM PMSF, 10 mM NaF, 0.3 mg/mL CalyculinA, 1 mM sodium orthovanadate, 10 mM β-glycerol-phosphate; all from Sigma-Aldrich and complete protease inhibitor cocktail, from Roche). Proteins were denatured at 95 °C for 5 min and the lysates were immediately used or stored at -20 °C until use. Samples were analysed by electrophoresis in polyacrylamide gels (All Kd SDS-PAGE, BioRad) and the proteins transferred to nitrocellulose membranes (GE Healthcare, Amersham). After 1 h of incubation in blocking buffer [20 mM Tris pH 7.6, 150 mM NaCl and 0.1% Tween-20 (TBS-T) with 5% of bovine serum albumin (BSA)], blots were incubated overnight at 4 °C with the corresponding primary antibody: anti-phospho-histone 2AX (Ser139) (EMD Millipore, 05-636, 1:1000), anti-lamin A/C (Santa Cruz Biotechnology, sc-47778, 1:1000), anti-GAPDH (Cell Signaling, 2118S, 1:5000) and anti-actin (Santa Cruz Biotechnology, sc-376248, 1:500) as antibodies for loading control. Then, membranes were washed (3x10 min) with TBS-T and incubated with the corresponding secondary antibody conjugated with peroxidase (Sigma- Aldrich) for 1 h at rt. Membranes were washed (6x5 min) with TBS-T and proteins were visualized by quimioluminiscence (GE Healthcare Amersham™ ECL™ Western Blotting Detection Reagents, Fisher Scientific) in a Fujifilm LAS-3000 imager. The bands were quantified by densitometry using the program ImageJ (NIH).

For immunocytofluorescence studies, mouse or human progeroid cells previously incubated with compound of interest or DMSO were seeded at a density of 3×10^4 or 1.5×10^4 cells/well over 12 mm diameter slide covers respectively in a 12-well plate and were incubated for 24 h at 37 °C with compound of interest. Cells were fixed with 4% formaldehyde (Ki67, pH2AX) or cold methanol (Lamin A/C, H3K9Me3) from Sigma-Aldrich during 20 min and permeabilized with PBS containing 0.2% Triton X-100 (PBS-T, Sigma-Aldrich). Then, cells were blocked with TBS 2% BSA during 30 min at rt, followed by incubation o.n. with anti-lamin A/C (Santa Cruz Biotechnology, SC-376248, 1:50), anti-Ki67 (Novus Biological, NB110-89717SS, 1:100), anti-H3K9Me3 (Abcam, ab8898, 1:1000) and anti-pH2AX (R&D Systems, AF2288, 1:20) in TBS with 2% BSA at 4 °C. After that, cells were washed with TBS (1x) and incubated in absence of light for 1 h with fluorescent goat anti-mouse (1:500, Alexa Fluor 488, Life Technologies) diluted in TBS with 2% BSA. Finally, cells were washed with TBS (2x) and the slide cover was carefully mounted with Immumount (Thermo Scientific). The visualization was carried out using confocal microscopy (Olympus IX83) equipped with a 40X oil immersion lens and the appropriate excitation and emission filters at the microscopy UCM facilities.
Quantification of different proteins intensity was performed using ImageJ (NIH) except for nuclear rim progerin intensity with Cell Profiler (Broad Institute).

7. Senescence-associated (SA) β-galactosidase activity.

SA β-galactosidase activity was determined by the FDG method. Mouse or human progeroid cells previously incubated with compound of interest or vehicle were seeded at a density of 2×10^3 or 10^3 cells respectively per well in triplicates in a 96-well plate overnight for attachment, washed, and then fixed for 5 min in 2% formaldehyde and 0.2% glutaraldehyde buffered with PBS. After washing twice in PBS, the fixed cells were incubated in the staining solution [0.1 mM fluorescein di-β-D-galactopyranoside (FDG, Sigma-Aldrich F2756), 3.7 mL 0.2 M citric acid, 6.3 mL 0.4 M Na_2HPO_4 (citrate–phosphate buffer), 1 ml 100 mM potassium ferricyanide, 1 mL 100 mM potassium

ferrocyanide, 0.6 mL 5 M NaCl, 0.2 mL 0.2 M $MgCl_2$, (all from Sigma Aldrich) and 6.2 mL of deionized water in a total volume of 20 mL] in the dark in a humidified incubator at 37 ºC for 24 h without CO_2 supply. Then, an equal volume of the supernatant of each well was transferred to a new 96-well plate for fluorescence measurement. The resultant production of fluorescein was measured using a FluoStar Optima instrument (BMG Labtech) with an excitation of 485 nm and emission at 535 nm. One well containing the reaction mixture without the cells was used as blank for subtracting the background fluorescence.

8. RNA isolation and real time RT-qPCR analysis.

Total RNA was extracted by RNAeasy Mini Kit (Qiagen) according to the manufacturer instructions and subsequently quantified using the NanoPhotometer N60 (Implen). RNA (500 ng) was reverse-transcribed to cDNA using a PrimeScript RT Master Mix (Perfect Real Time) kit (TAKARA). cDNA products were amplified by qPCR using PowerUp SYBR Green Master Mix (Applied Biosystems). All reactions were carried out in triplicate. Quantitative real-time PCR was run in the QuantStudio 7FLEX (Applied Biosystems) system and results were normalized according to β-actin quantification. Specific `in-house´ designed primers (see sequence below) were purchased from Metabion. The threshold cycle (Ct) was determined, and the relative gene expression was

Mouse	
Cdkn2a	Forward: GAGGAAAGCGAACTCGAGGA<br>Reverse: TGCCCATCATCATCACCTGAAT
Actb	Forward: CACTGTCGAGTCGCGTCCA<br>Reverse: CATCCATGGCGAACTGGTGG
Il1a	Forward: CCCATGATCTGGAAGAGACCA<br>Reverse: CAAACTTCTGCCTGACGAGC

expressed as follows: Relative amount , where Ct =Ct (target) – Ct (housekeeping gene) and Δ(ΔCt)=ΔCt (sample)− ΔCt (control).
Primers

Human	
CDKN2A	Forward: GGAGGCCGATCCAGGTCAT<br>Reverse: CACCAGCGTGTCCAGGAAG
CDKN1A	Forward: CTGCCGAAGTCAGTTCCTTGT<br>Reverse: TGACATGGCGCCTCCTCTG
IL6	Forward: AGAGGCACTGGCAGAAAACA<br>Reverse: TCACCAGGCAAGTCTCCTCA
ACTB	Forward: ACAGAGCCTCGCCTTTGCC<br>Reverse: GATATCATCATCCATGGTGAGCTGG

9. Oxygen consumption rates (OCR) measurement.

Cells were seeded at 10K cells per well in XF HS Miniplates (Agilent) in DMEM medium and incubated overnight. The OCRs were measured in DMEM assay medium (10 mM glucose, 1 mM pyruvate, 2 mM glutamine, 2% FBS, at pH 7.4) at 37◦ C using the XF HS Mini extracellular flux analyser (Agilent). Compounds used in the assay include: oligomycin (1.5 μM), FCCP (1 μM), antimycin A (1 μM) and rotenone (1 μM).

10. Cell cycle.

Cells were harvested by trypsin, centrifuged and washed with PBS. Cells pellets were added with 70% (vol/vol) ice-cold ethanol. The mixture was incubated for 15min at 4ºC, centrifuged 2500 rpm, washed with PBS and then re-suspended in 500ul staining buffer (25ug/mL propidium iodide and 100μg/ml DNAse-free RNAse both from Sigma-Aldrich in PBS). The mixture was then allowed to incubate for 30min at 37ºC in the dark and samples were finally analysed by FACScalibur (Becton Dickinson) flow cytometer

11. Transmission electron microscopy.

Cells previously collected in 1.5 mL tube, were washed with PBS and fixed with 2.5% glutaraldehyde and 4% paraformaldehyde in Milloning´s phosphate buffer (41.5 mL 2.26% $NaH_2PO_4 \cdot H_2O$ and 8.5 mL 2.52% NaOH both in milliQ water, pH 7.2-7.4) for 4h at 4ºC. Cells were washed 3 times in Milloning buffer and remained overnight at 4ºC. Cells were incubated for 1 h in 1% OsO_4 at room temperature and then thoroughly washed with milliQ water. Samples were gradually dehydrated in increasing concentrations of acetone, embedded in Spurr's resin (TAAB Laboratories), and polymerized at 70 ºC for 48 h. Thin sections were obtained using an EM UC7 ultramicrotome (Leica) on 200 mesh copper grids and stained with 2% uranyl acetate and lead citrate (Reynolds). The samples were analysed and examined in the transmission electron microscope JEM 1010 at 80 keV.

12. RNA-seq analysis.

Total RNA of HGPS fibroblasts (HGADFN167) and UCM-18142-treated HGPS fibroblasts were extracted for RNA sequencing. The RNA concentration and integrity were assessed using the Tape Station 4150 system (Agilent). The RNA-seq libraries were constructed after following steps: mRNA enrichment, cDNA synthesis, purification, fragment selection and amplification. Sequencing was carried on an Illumina PE150 platform at Haplox company (Hong Kong). All raw reads were mapped to the human genome (Grch38) using the software HISAT2. HTSeq was used to calculate Reads Count and transcripts per kilobase per million reads were computed. A cut-off p value <0.01 and absolute values of log2-fold changes > than 1 or < -1 were used for differential gene expression analysis. The data for this study have been deposited in the European Nucleotide Archive (ENA) at EMBL-EBI under accession number PRJEB100759.

13. Immunoprecipitation.

Co-immunoprecipitation experiment was performed to capture ternary complexes between CRBN and progerin in *Lmna*$^{G609G/G609G}$ fibroblasts treated in the presence of compound **2** at 5 μM for 6 h or 0.1% DMSO using conditions that preserve protein–protein interactions while allowing efficient extraction. Cell lysates were prepared fresh the same day in cold lysis buffer (1% Triton X-100, 0.5% IGEPAL CA-630, 0.25% Sodium deoxycholate, 175 mM NaCl, 25 mM Tris-HCl pH 7.6) supplemented immediately before use with PMSF, protease inhibitor cocktail, and phosphatase inhibitor cocktail (100:1:1:1 ratio), and adjusted to 1 μg/μL protein concentration. Dynabeads Protein G magnetic beads (17 μL; binding capacity ≈8 μg antibody/mg beads; Invitrogen #2998966) were vortexed, magnetically separated, and incubated with 2 μg anti-CRBN antibody (Cell Signalling #D8H3S) in 200 μL PBS containing 0.02% Tween-20 for 60 min at room temperature with orbital rotation. Protein extract adjusted (400 μg total protein) was added to antibody-coupled beads, gently resuspended, and incubated overnight at 4 °C with orbital rotation to allow antigen binding. After incubation, bead complexes were washed three times with 200 μL PBST, resuspended in 30 μL Laemmli

buffer containing 2.5% β-mercaptoethanol, and heated at 95 °C for 5 min prior to magnetic separation and loading of supernatants onto SDS-PAGE gels. Input lysates (~30 μg) were prepared in parallel as Western blot controls.

14. Cellular thermal shift assay.

Progeroid mouse fibroblasts (*Lmna*$^{G609G/G609G}$) were seeded (2x10^6 cells) in 100 mm dishes 24 h before the experiment. Next day, cells were first washed with PBS and then incubated with the proteasome inhibitor (S)-MG132 (20 μM) for 2 h at 37 ºC and 5% CO_2. Then, the cells were incubated with compound **2** at the corresponding concentration or with vehicle for 60 min at 37 ºC and 5% CO_2. Cells were then washed with PBS, detached by treatment with trypsin, and collected by centrifugation at 250*g* and 25 ºC for 5 min, washed with PBS and resuspended in PBS in 0.2 mL microtubes. Then, homogenates or cells were transferred to PCR tubes and heat-shocked for 3 min at the appropriate temperature (37 to 61 ºC) followed by rapid cooling to 25 ºC using a SensoQuest LabCycler Thermocycler (Progen Scientific Ltd). Igepal CA-630 was then added to the samples (final concentration of 1 % v/v), and the samples were thoroughly mixed and denatured by three cycles of freezing/thawing (immersion in liquid nitrogen followed by fast thawing). The non-soluble fraction was separated by centrifugation at 12000*g* for 20 min at 4 ºC and the supernatants were transferred to clean microtubes. Then, Laemmli buffer (Bio-Rad) was added and samples were heated at 100 °C for 10 min and samples were subjected to western blot analysis as described previously.

15. Animal experiments.

All scientific procedures with animals were conformed to EU Directive 2010/63 EU and Recommendation 2007/526/EC, enforced in Spanish law under Real Decreto 53/2013. Animal protocols were approved by the Committee of Animal Experimentation of Universidad Complutense de Madrid and the Animal Protection Area of the Comunidad Autónoma de Madrid (PROEX 114.4/24). Animal studies were carried out in *Lmna*$^{G609G/G609G}$ knock-in mice ubiquitously expressing progerin[19] and control *Lmna*$^{+/+}$ littermates, obtained by breeding *Lmna*$^{G609G/+}$ mice kindly donated by Prof. V. Andrés (CNIC, Spain). Mice were maintained in the animal facility of the Universidad Complutense de Madrid under specific pathogen free conditions.

For pharmacokinetic studies, compound **2** was administered intraperitoneally and blood was collected at the selected time points post-dose (n=2-3 per time point) by cardiac puncture. Blood was allowed to clot at room temperature for 30 min and centrifuged at 4 ºC for 10 min at 10000*g*. The supernatant was transferred to a clean polypropylene tube and stored at -80 ºC until analysis. For analysis, a volume of cold acetonitrile was added to the serum. The sample was incubated in an ice bath for 10 min and centrifuged at 4 ºC for 10 min at 18000*g*. The resulting organic layer was filtered through a polytetrafluoroethylene filter (0.2 μm, 13 mm diameter, Agilent Technologies) and 30 μL of the sample analyzed by liquid chromatography coupled to mass spectrometry multiple reaction monitoring at the UCM's Mass Spectrometry CAI. Separation was performed using a Phenomenex Gemini 5 μm C18 110Å 150x2 mm column (run time 9 min; flow 0.7 mL/min; gradient: 0 min 5% B; 4-7 min 100% B; 7.5 min 5% B; Phase A: water with formic acid 0.1%; Phase B: acetonitrile). The entire LC eluent was directly introduced to an electrospray ionization (ESI) source operating in the positive ion mode for LC MS/MS analysis on a Shimadzu LCMS-8030 triple quadrupole mass spectrometer coupled to

UHPLC with an oven temperature of 31.5 °C. The mass spectrometer ion optics were set in the multiple reaction monitoring mode and the transition selected for quantification was 616.10 > 228.95 (CE: -27 V).

For in vivo treatment, compound **2** was administrated at a concentration of 25 mg/kg diluted in sterile corn oil (Sigma-Aldrich) intraperitoneally three times per week starting at the age of 6 weeks. The same quantity of corn oil was injected to control $Lmna^{G609G/G609G}$ and $Lmna^{+/+}$ mice. Treatments did not produce any apparent damage or stress in mice. The weight of each mouse was registered 2 to 3 times per week during the duration of the treatment.

After 7 weeks of treatment, mice were euthanized by CO_2 inhalation. Immediately after sacrifice, tissues were harvested, frozen in dry ice, and kept at -80 ºC until use. For immunoblot analysis, a portion of the corresponding tissue was homogenized, and the protein concentration was determined. Samples containing equivalent amounts of total protein were then subjected to polyacrylamide gel electrophoresis and analyzed as described above.

PROTAC **2**

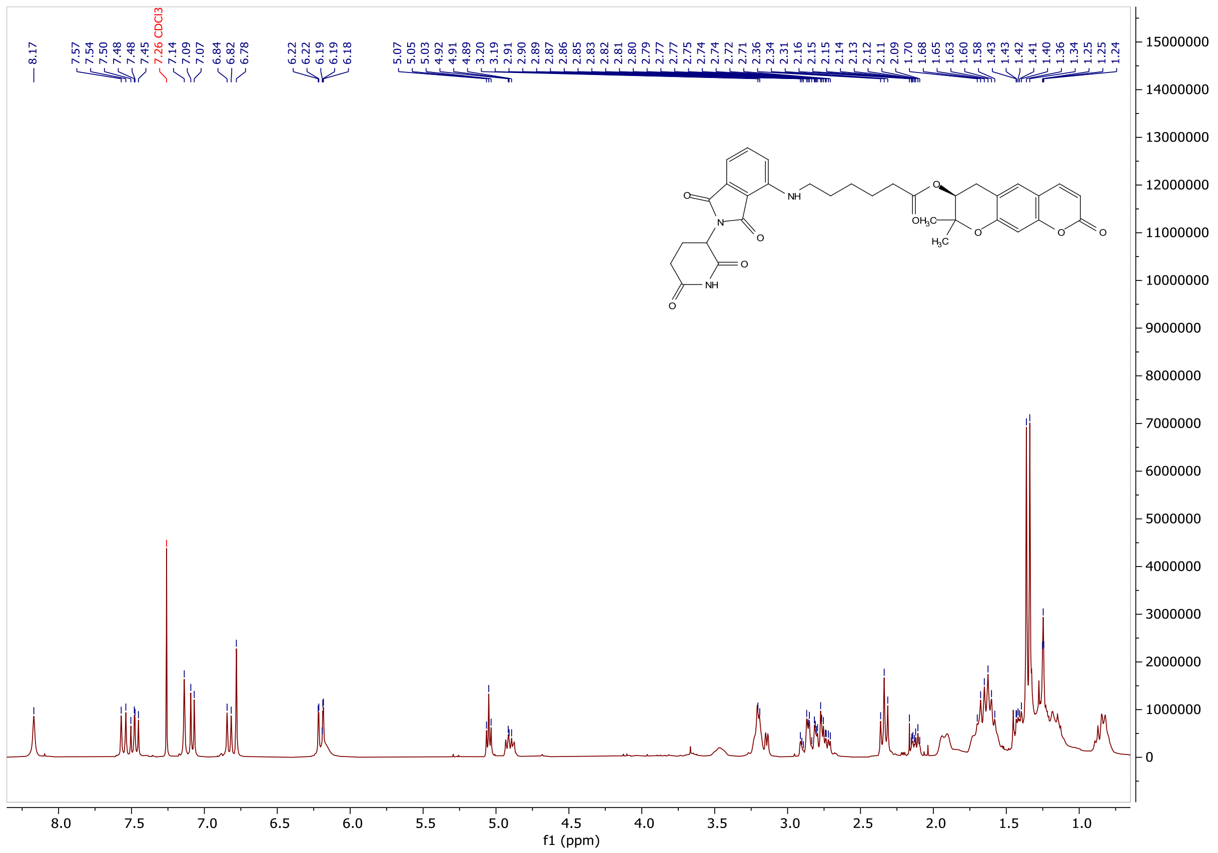

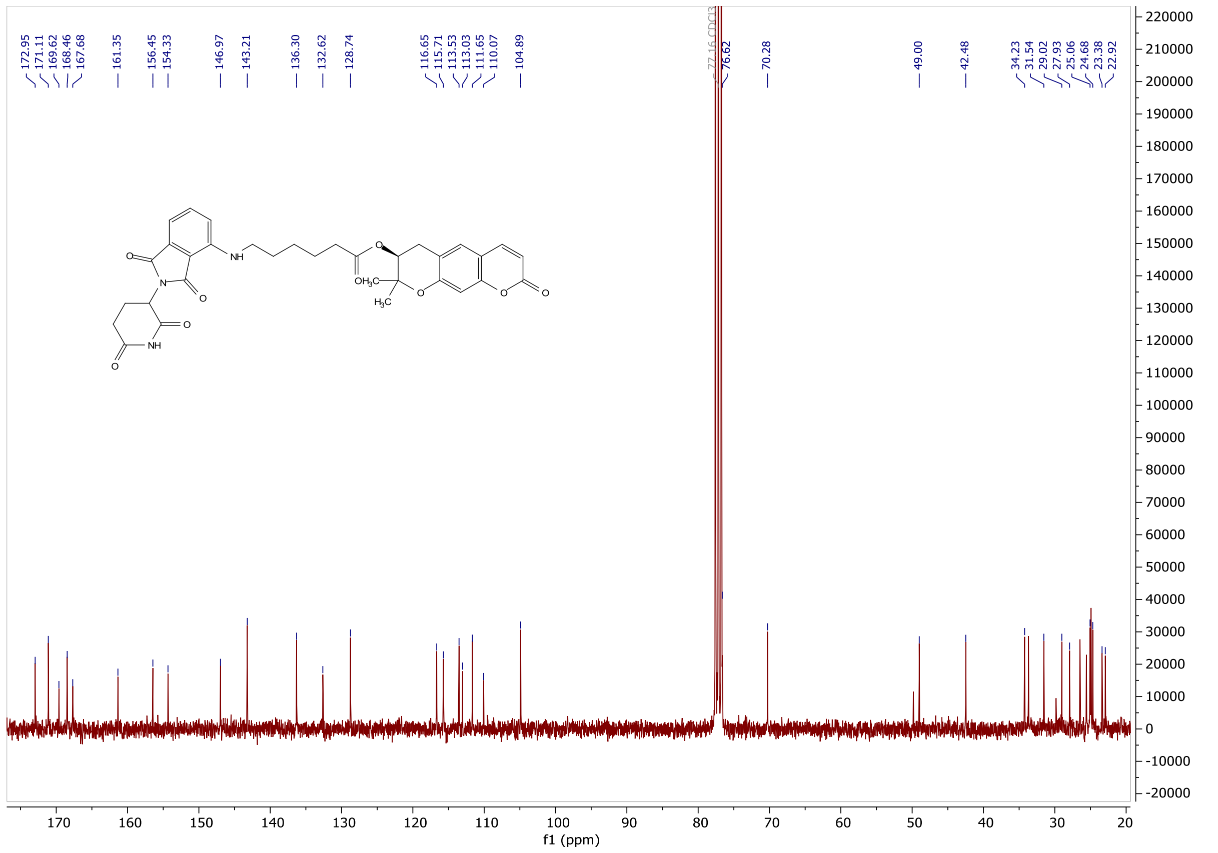

PROTAC **3**

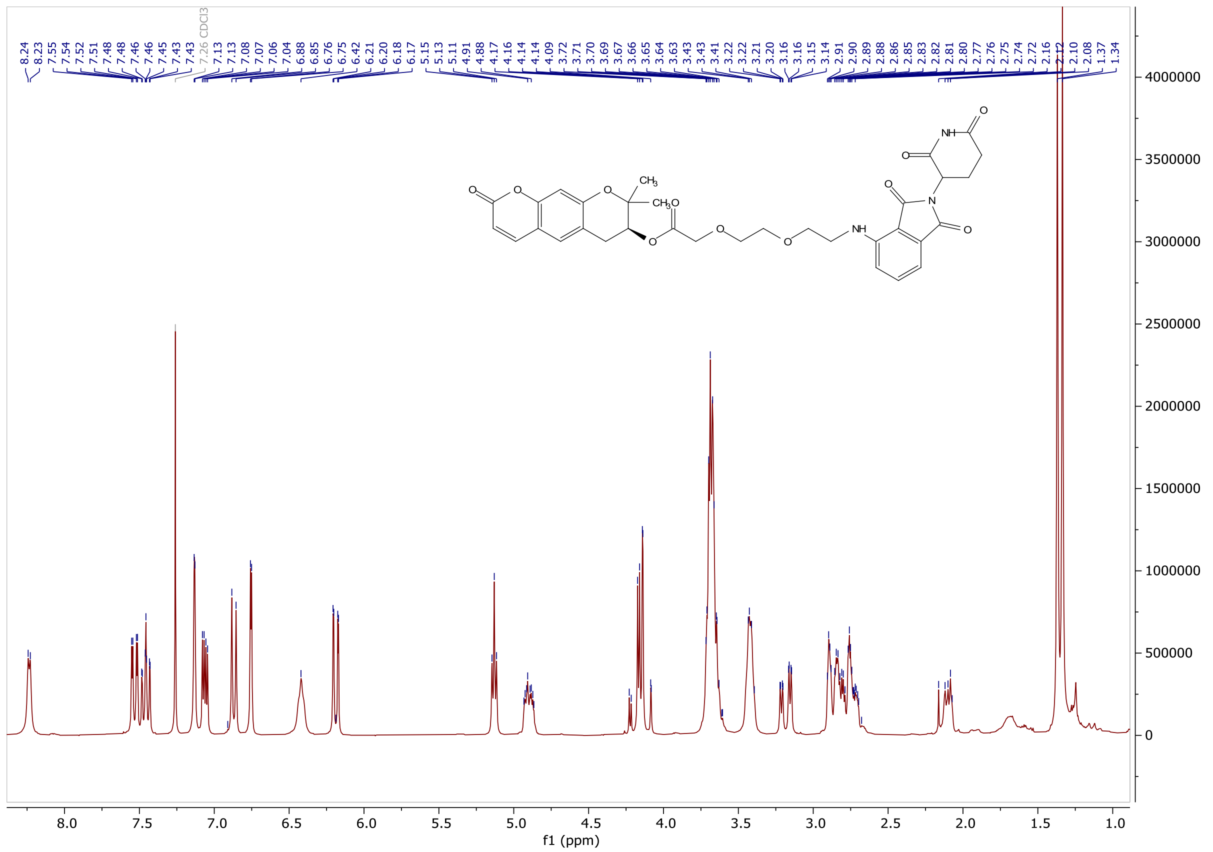

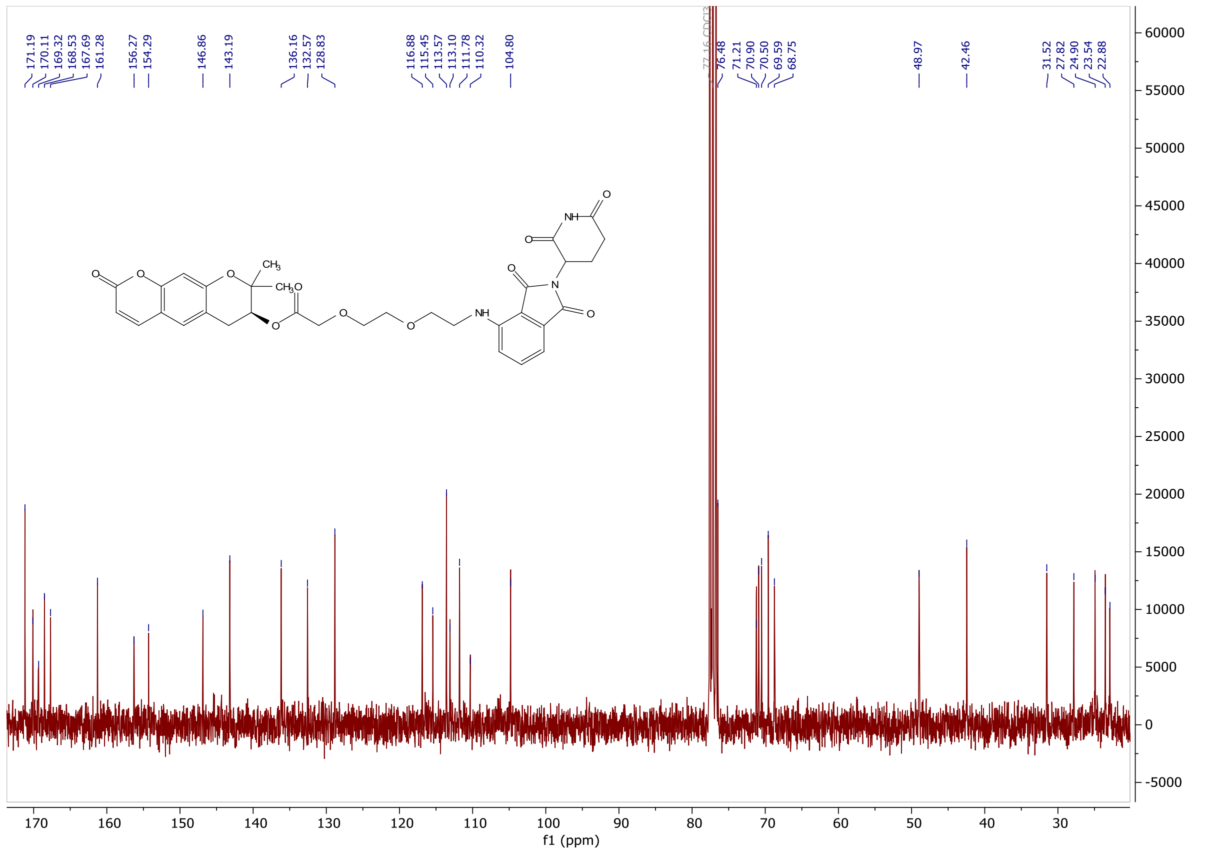

PROTAC **4**

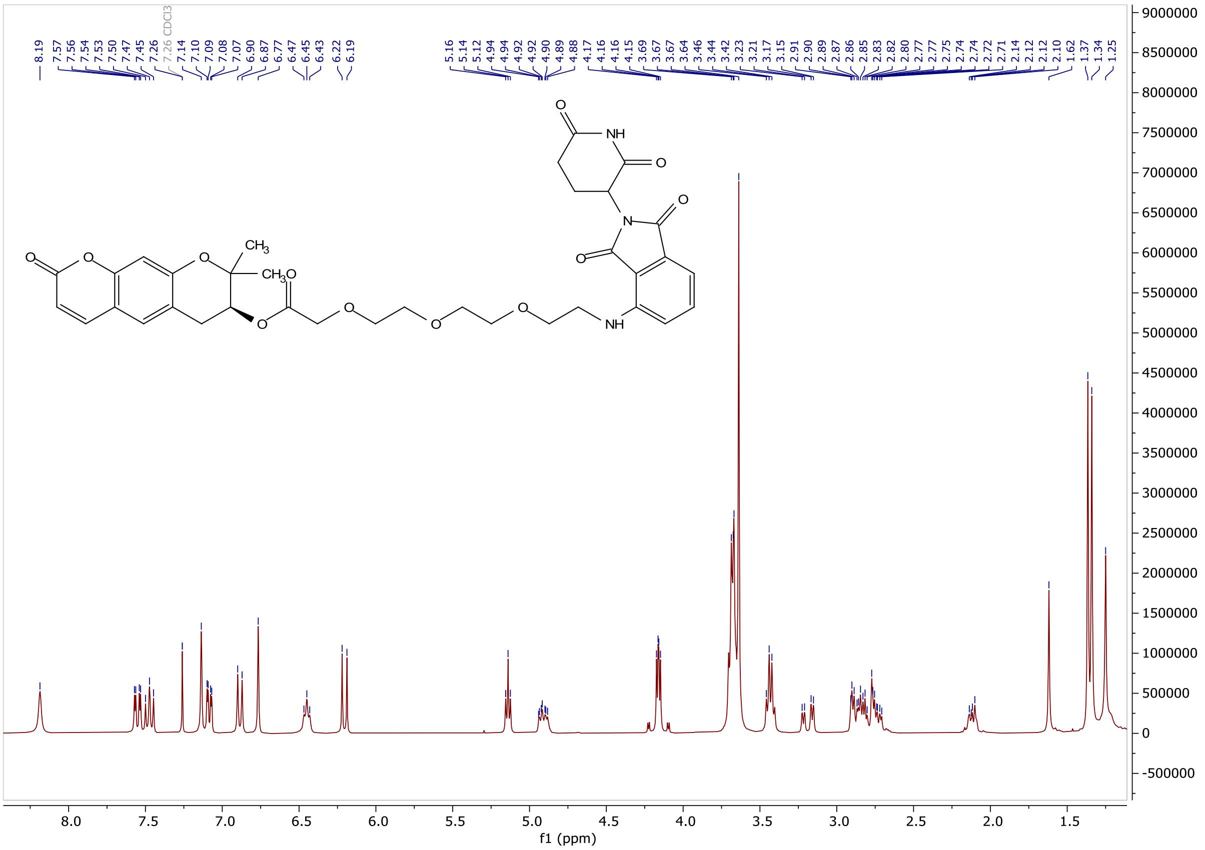

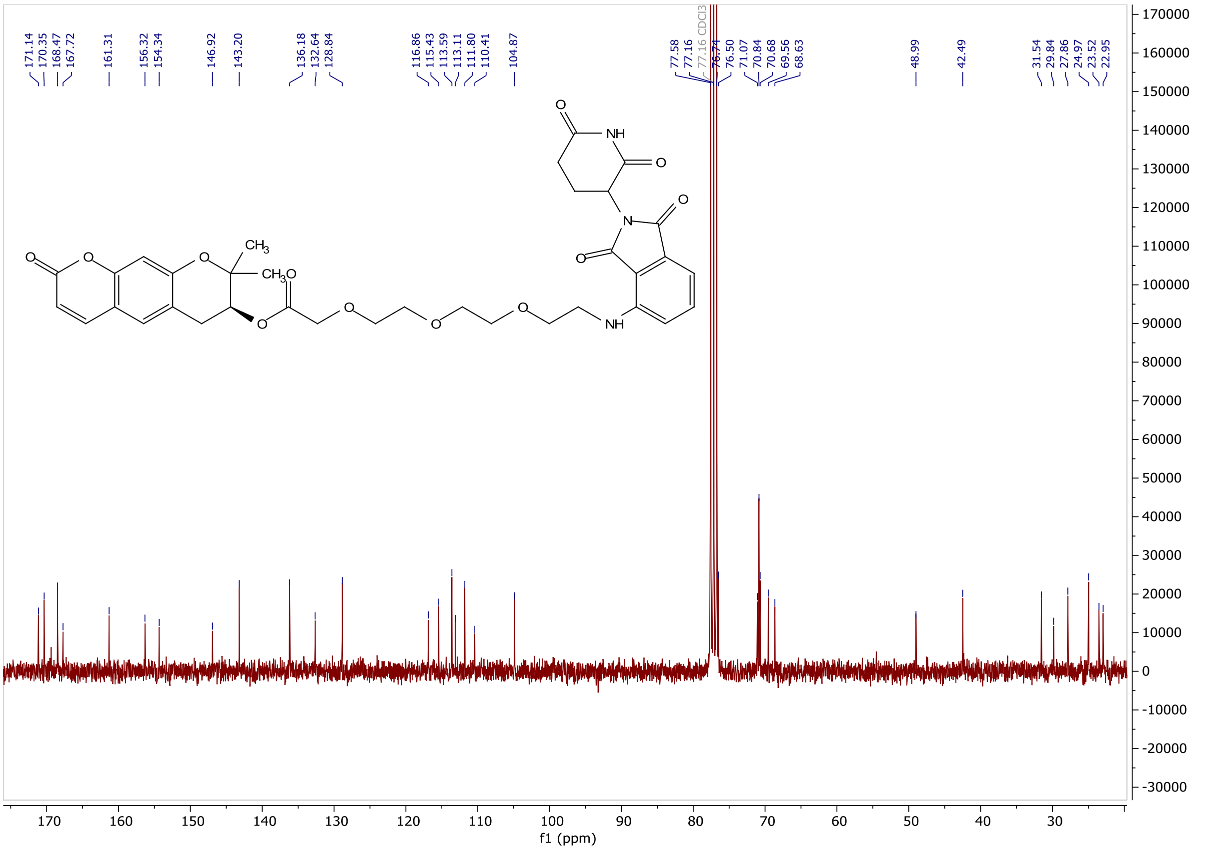

PROTAC **5**

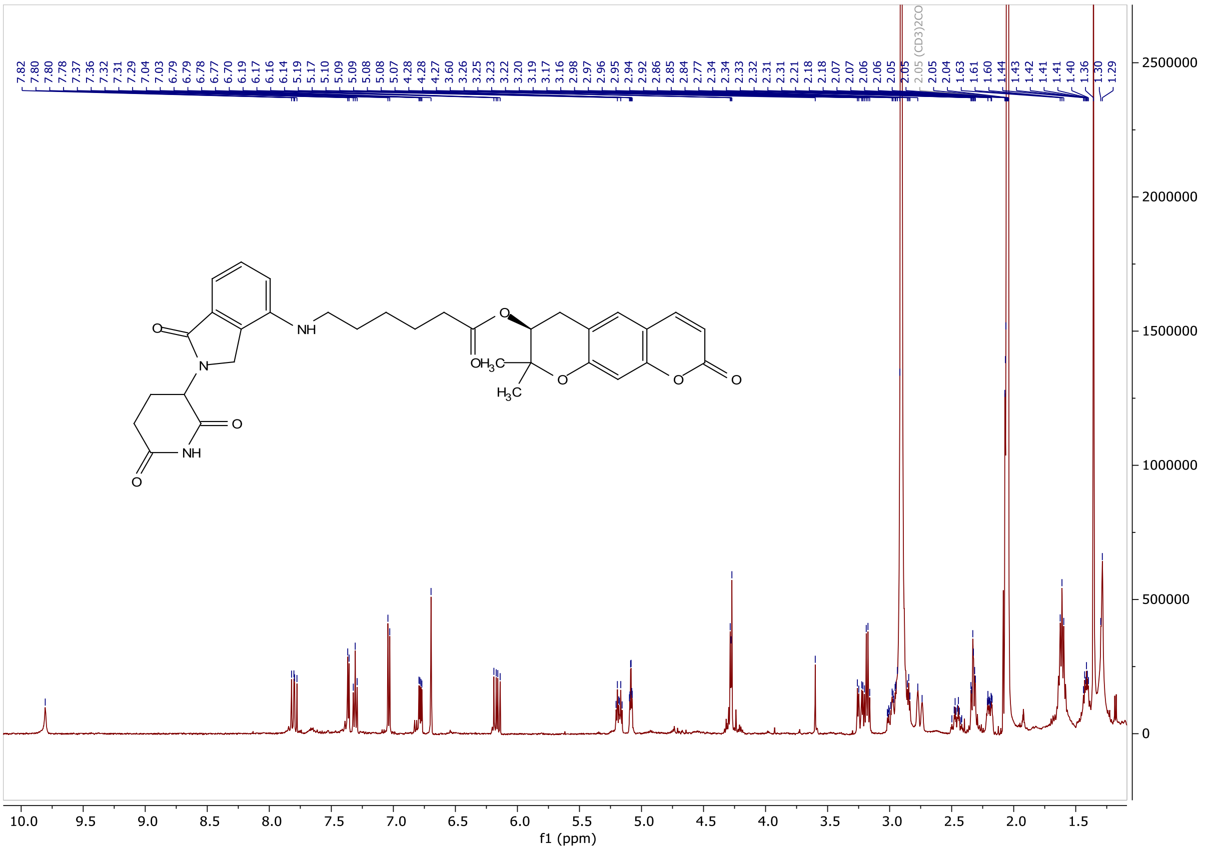

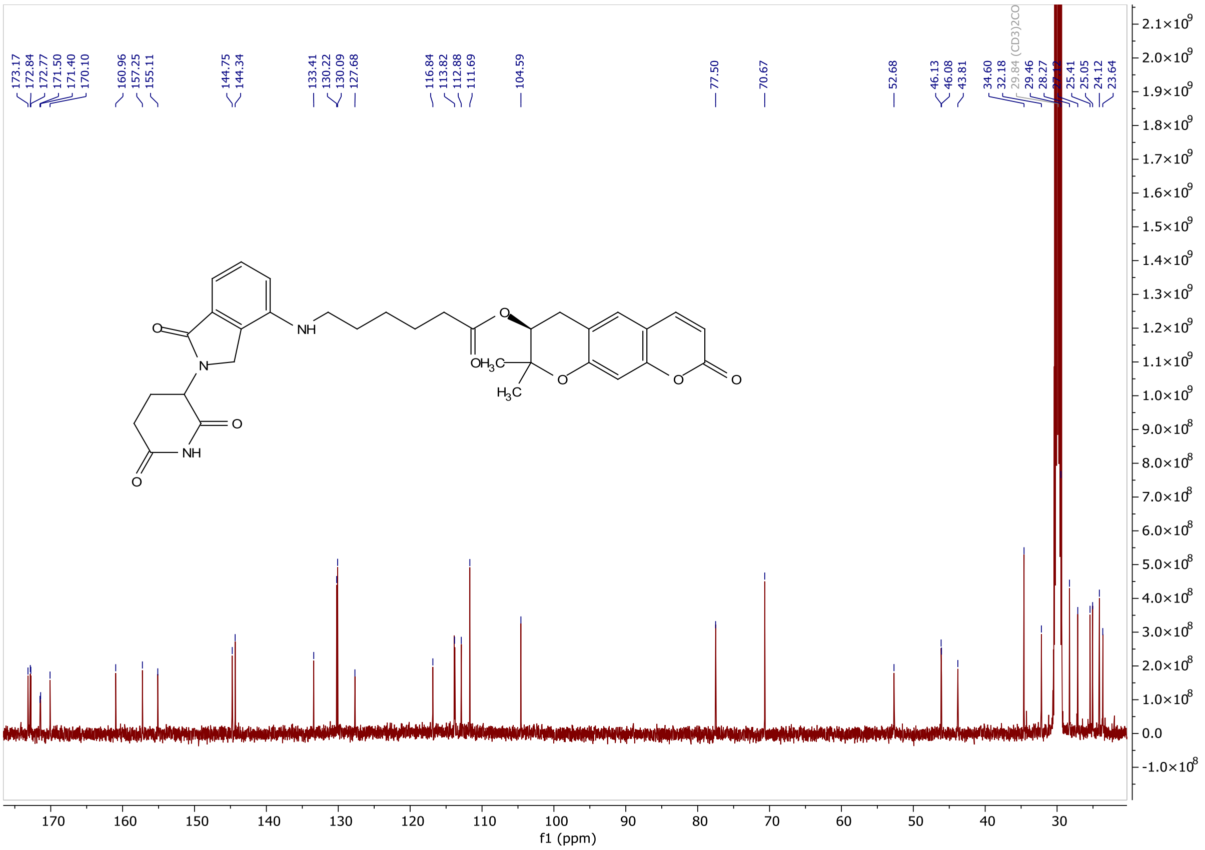

PROTAC **6**

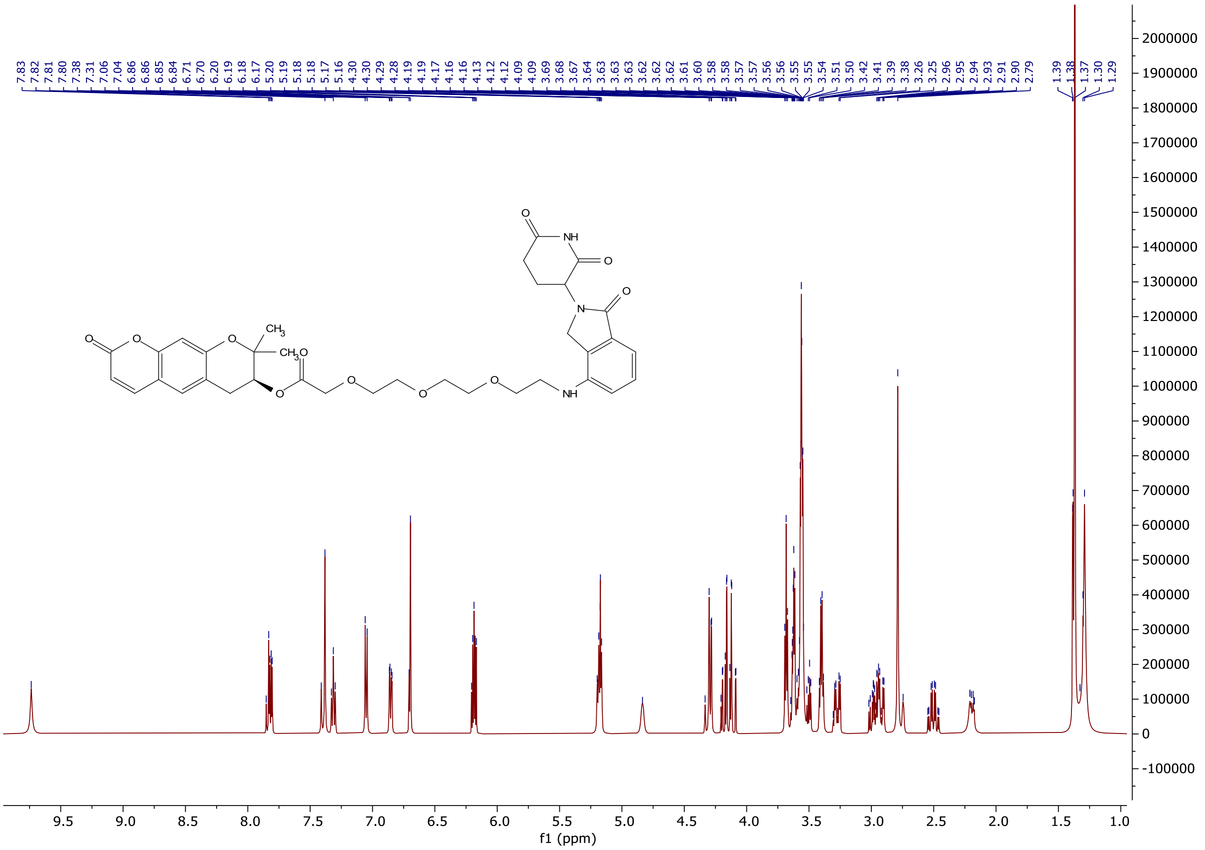
7.83
7.82
7.81
7.80
7.38
7.31
7.06
7.04
6.86
6.86
6.85
6.84
6.71
6.70
6.20
6.19
6.18
6.17
5.20
5.19
5.18
5.18
5.17
5.16
4.30
4.30
4.29
4.28
4.19
4.19
4.17
4.16
4.16
4.13
4.12
4.12
4.09
4.09
3.69
3.68
3.67
3.64
3.63
3.63
3.63
3.62
3.62
3.62
3.61
3.60
3.58
3.58
3.57
3.57
3.56
3.56
3.55
3.55
3.54
3.51
3.50
3.42
3.41
3.39
3.38
3.26
3.25
2.96
2.95
2.94
2.93
2.91
2.90
2.79
1.39
1.38
1.37
1.30
1.29
f1 (ppm)

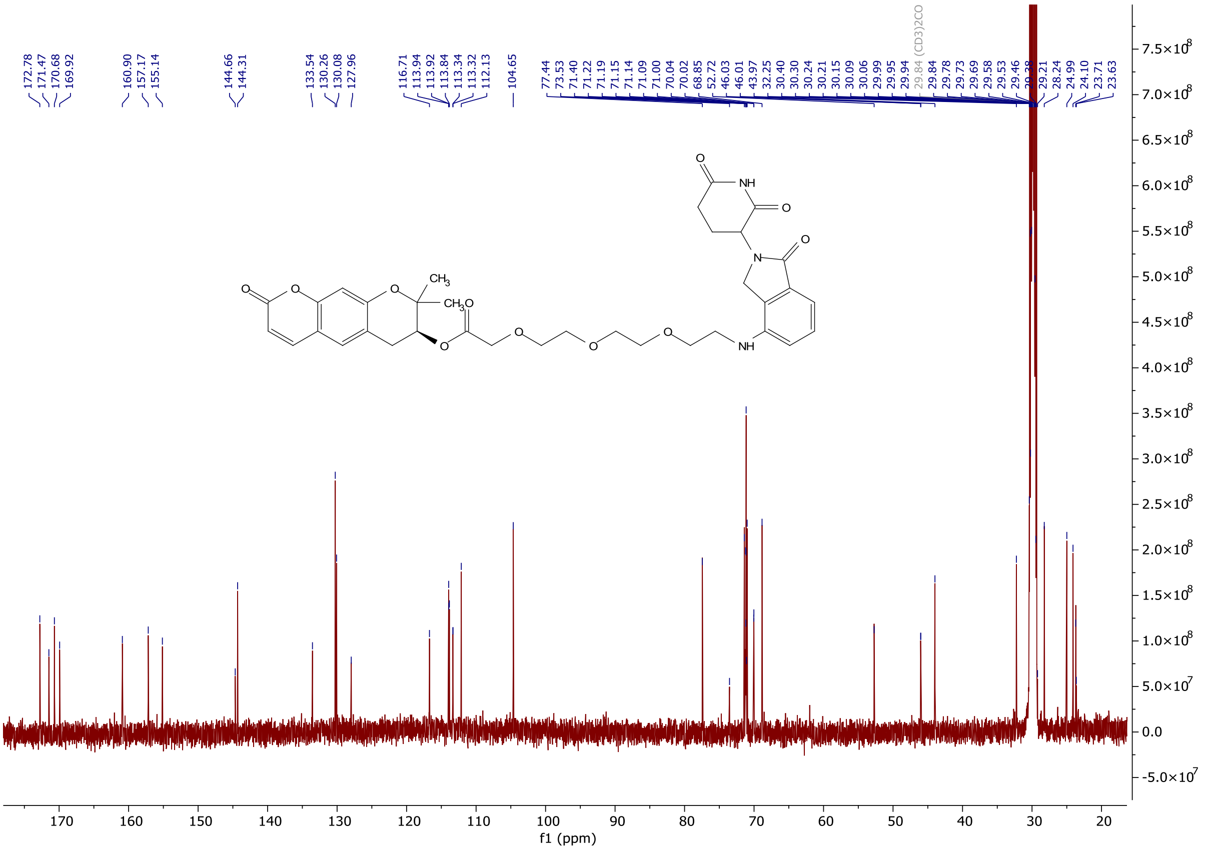
172.78
171.47
170.68
169.92
160.90
157.17
155.14
144.66
144.31
133.54
130.26
130.08
127.96
116.71
113.94
113.92
113.84
113.34
113.32
112.13
104.65
77.44
73.53
71.40
71.22
71.19
71.15
71.14
71.09
71.00
70.04
70.02
68.85
52.72
46.03
46.01
43.97
32.25
30.40
30.30
30.24
30.21
30.15
30.09
30.06
29.99
29.95
29.94
29.84 (CD3)2CO
29.84
29.78
29.73
29.69
29.58
29.53
29.46
29.21
28.24
24.99
24.10
23.71
23.63
f1 (ppm)

PROTAC **7**

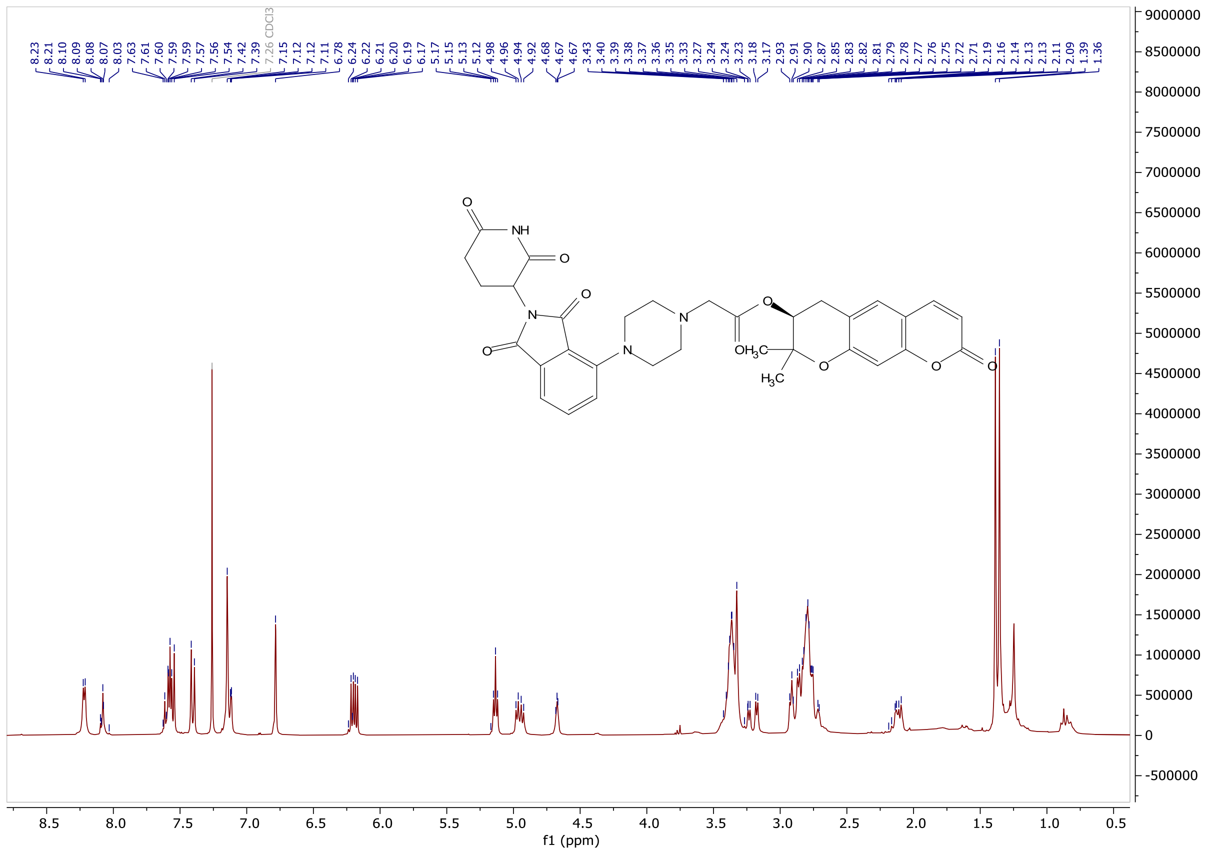

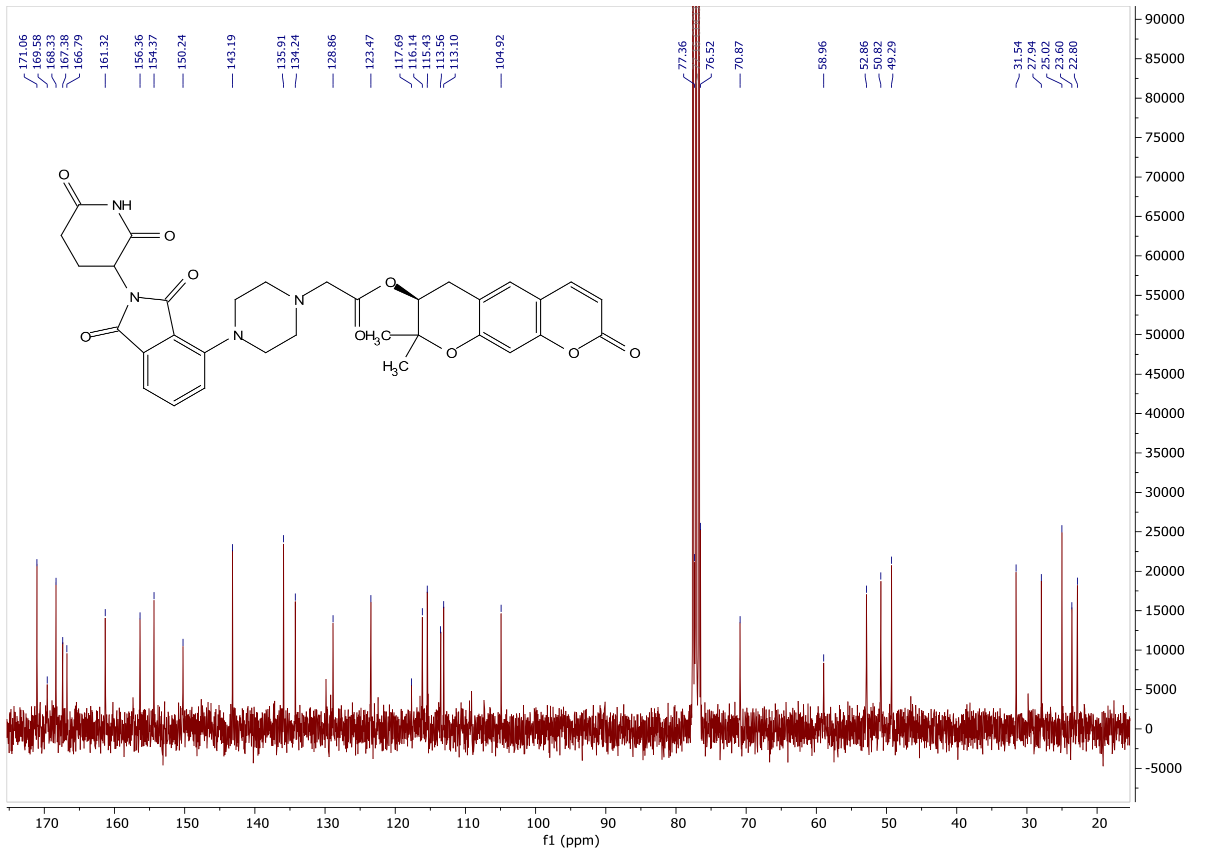

PROTAC **8**

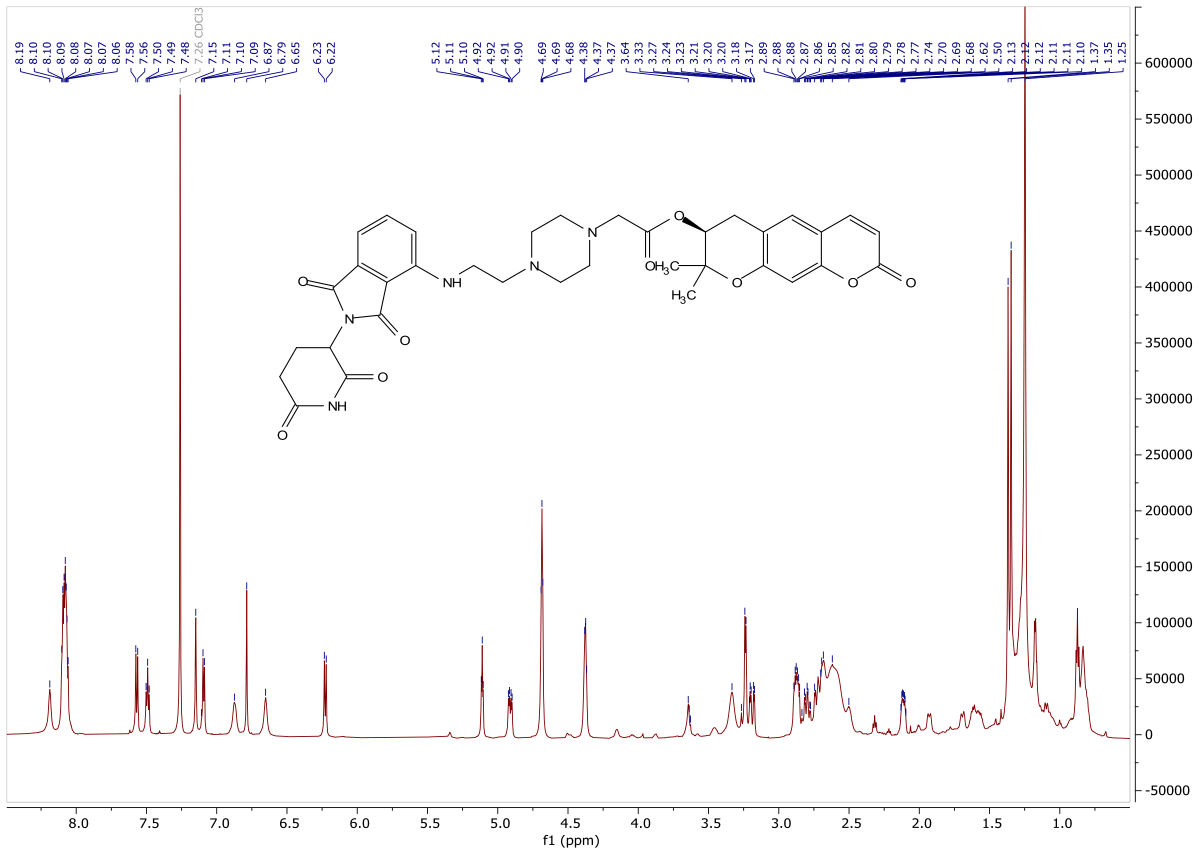

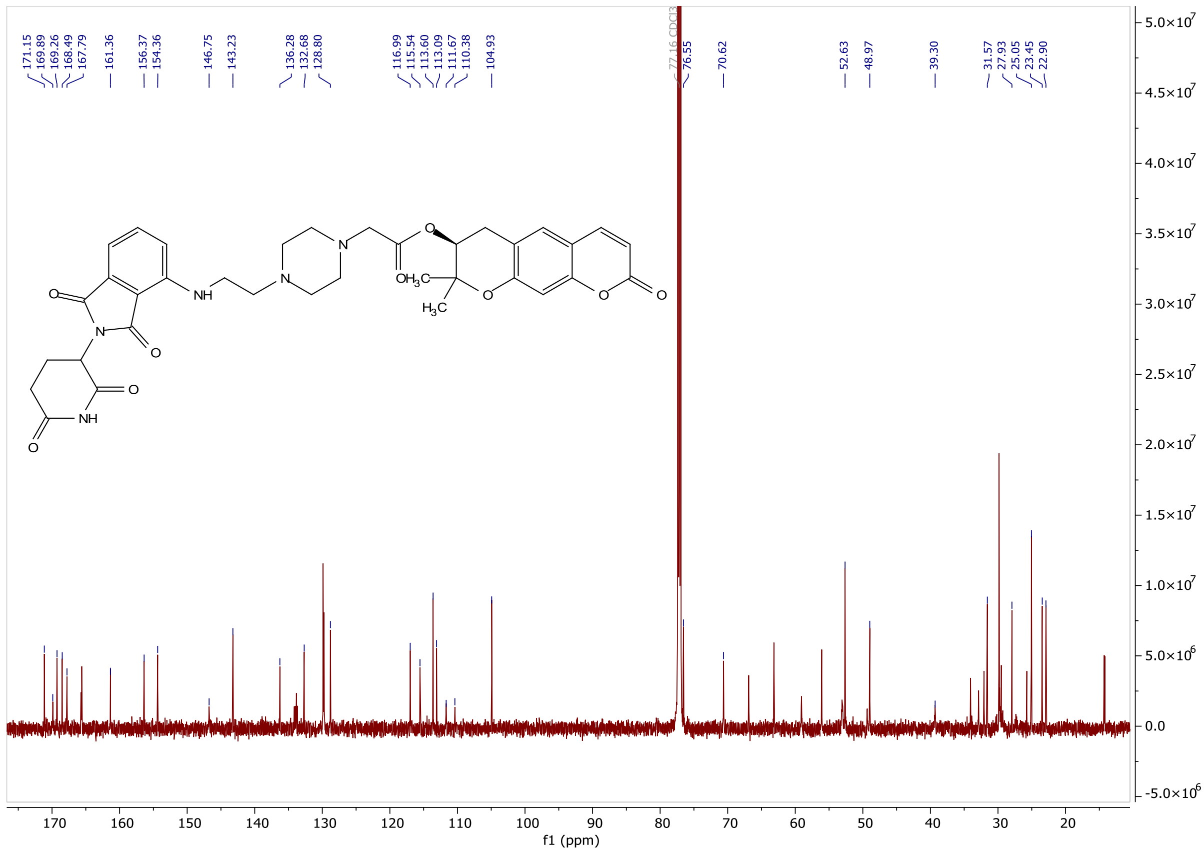

PROTAC **9**

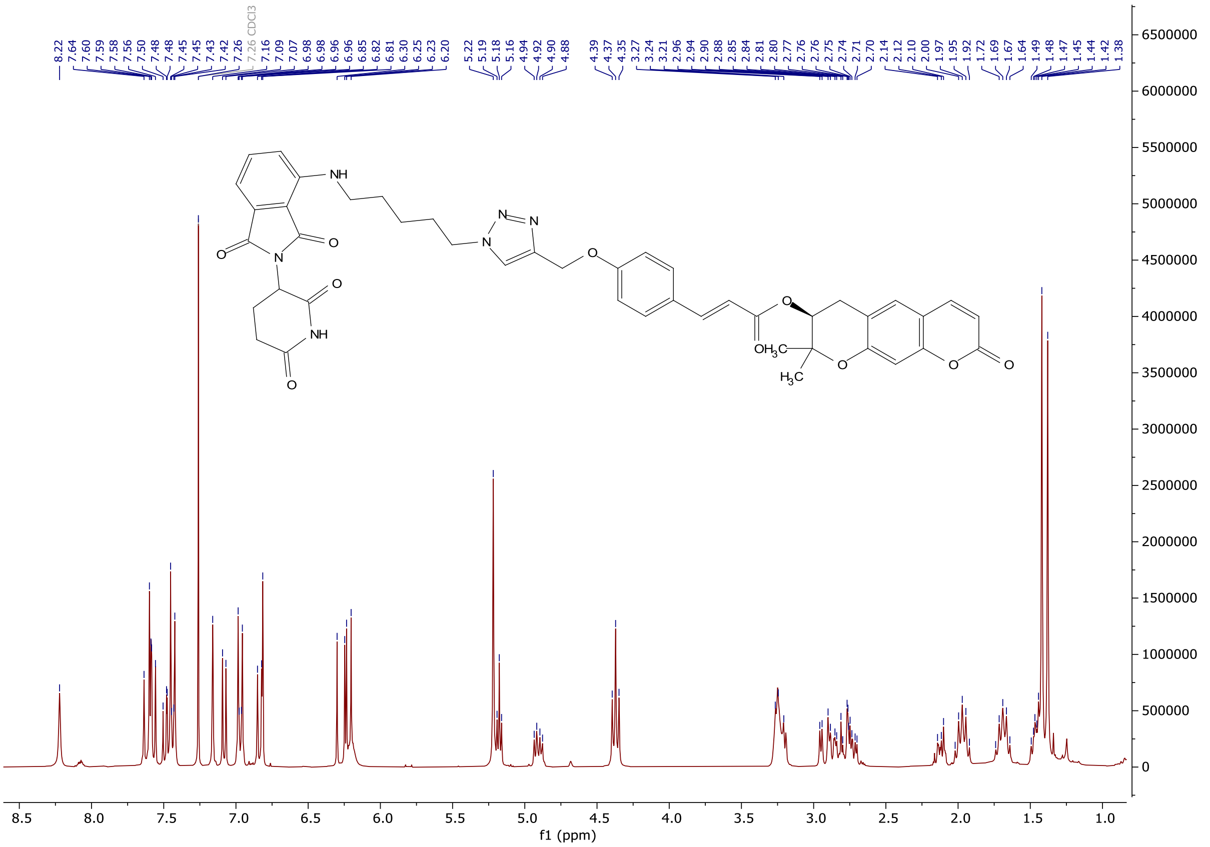

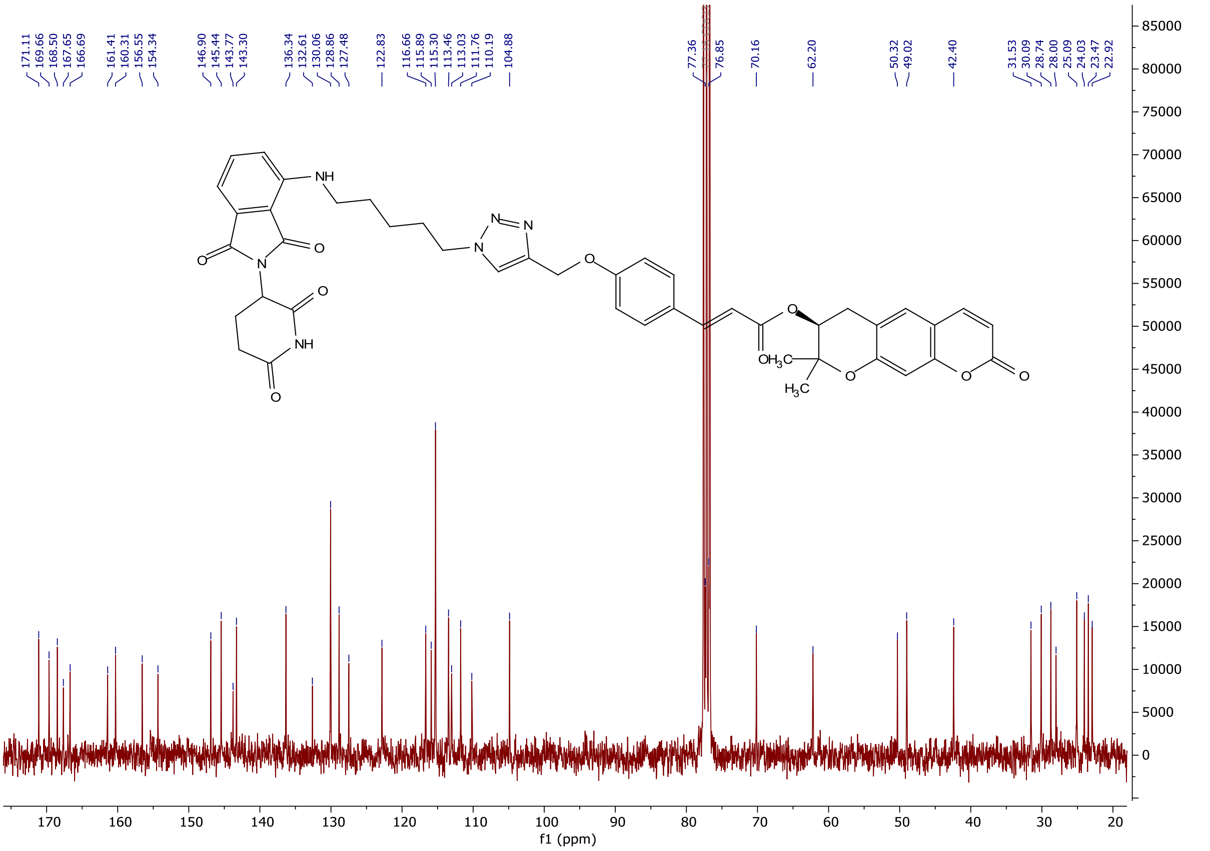

PROTAC **10**

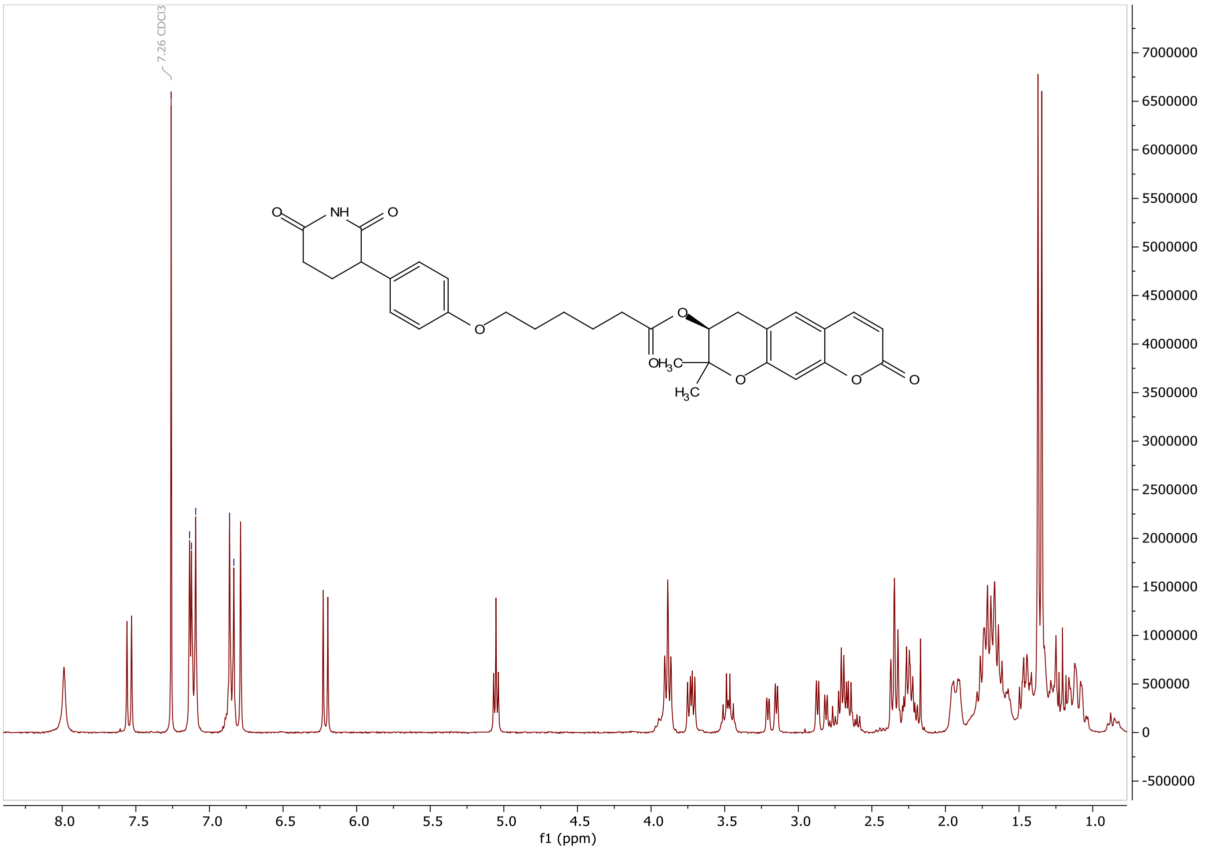

7.26 CDCl3
f1 (ppm)

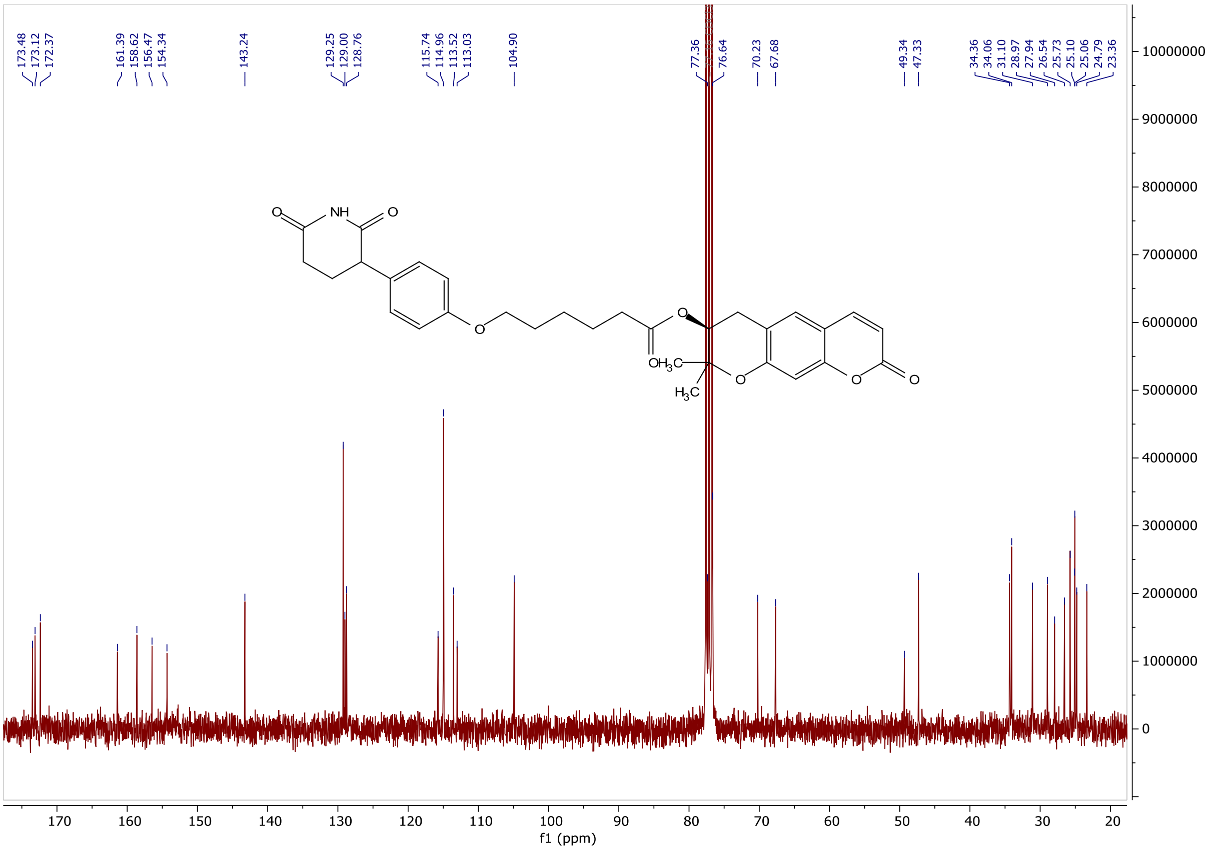

173.48
173.12
172.37
161.39
158.62
156.47
154.34
143.24
129.25
129.00
128.76
115.74
114.96
113.52
113.03
104.90
77.36
76.64
70.23
67.68
49.34
47.33
34.36
34.06
31.10
28.97
27.94
26.54
25.73
25.10
25.06
24.79
23.36
f1 (ppm)

PROTAC **11**

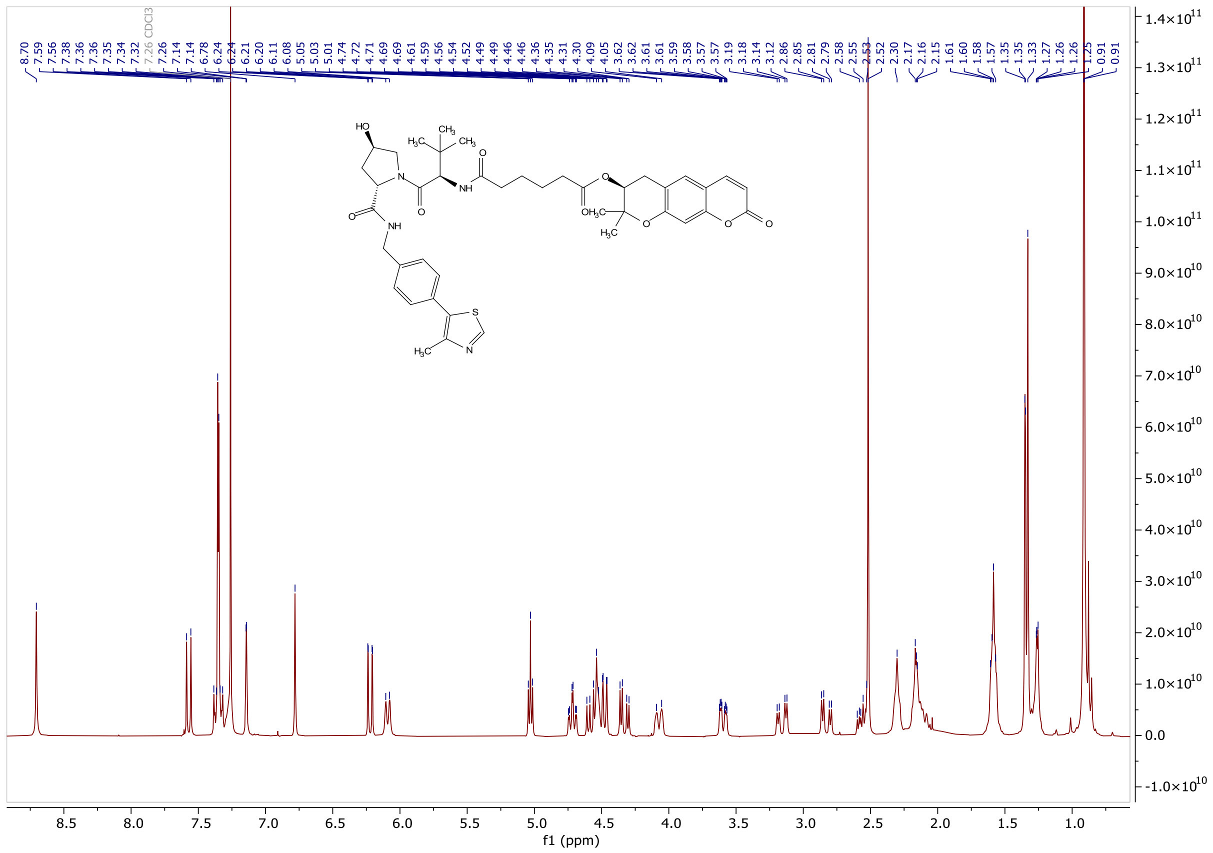

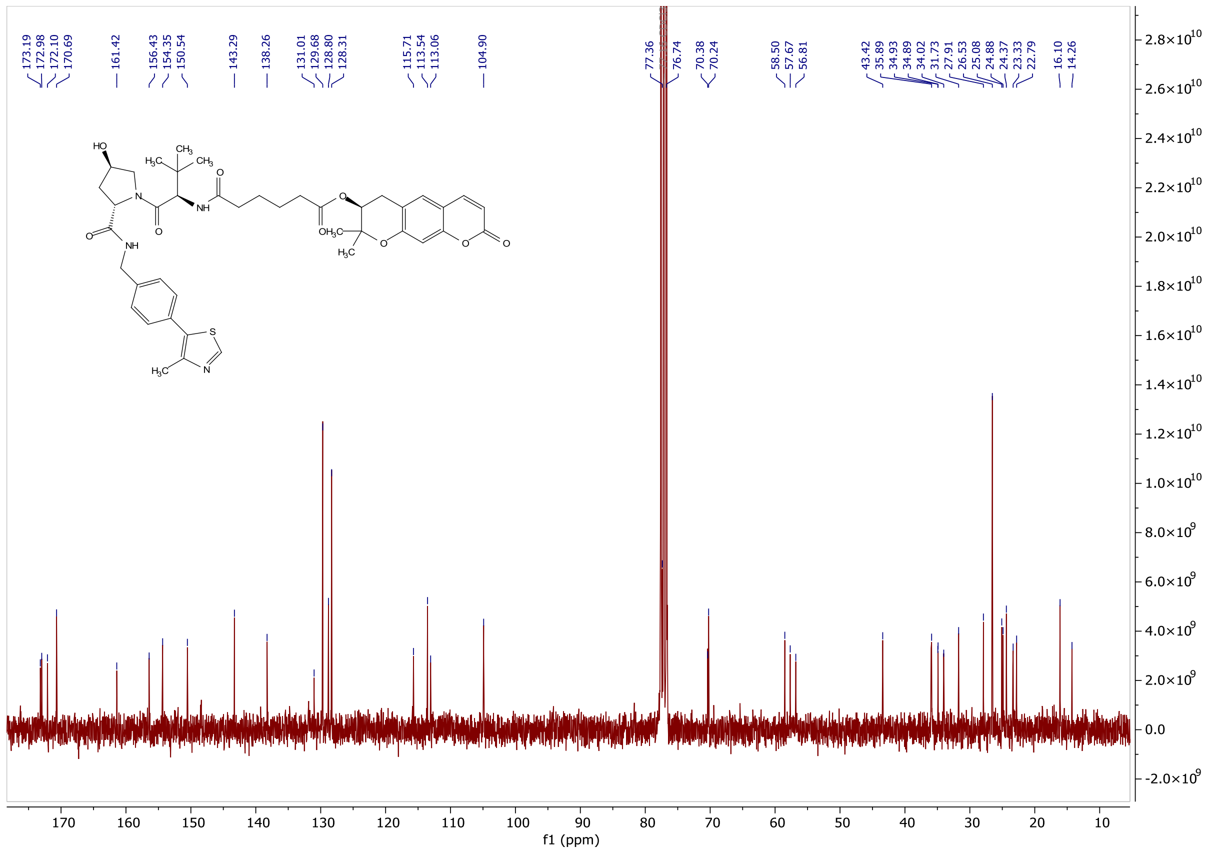

PROTAC **12**

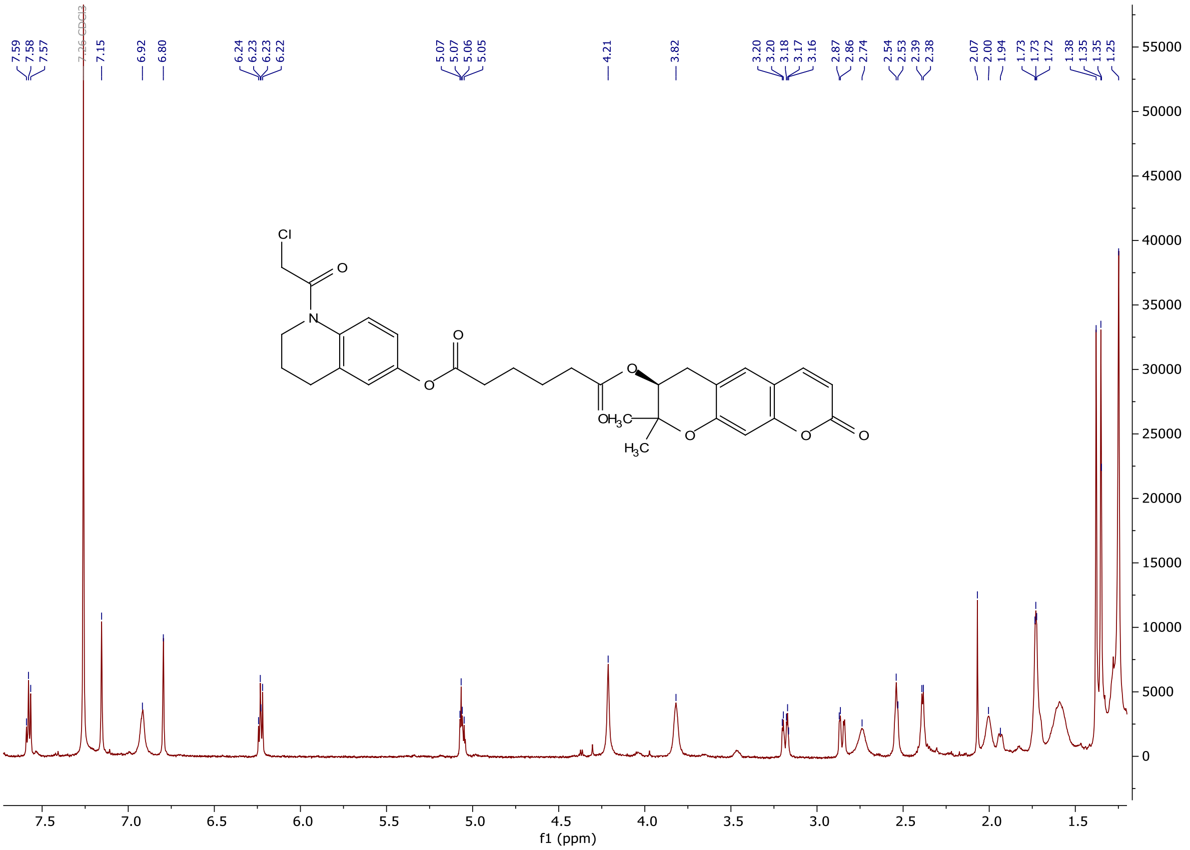
7.59
7.58
7.57
7.26 CDCl3
7.15
6.92
6.80
6.24
6.23
6.23
6.22
5.07
5.07
5.06
5.05
4.21
3.82
3.20
3.20
3.18
3.17
3.16
2.87
2.86
2.74
2.54
2.53
2.39
2.38
2.07
2.00
1.94
1.73
1.73
1.72
1.38
1.35
1.35
1.25
Cl
O
N
O
O
O
O
O
O
H_3C
H_3C
f1 (ppm)

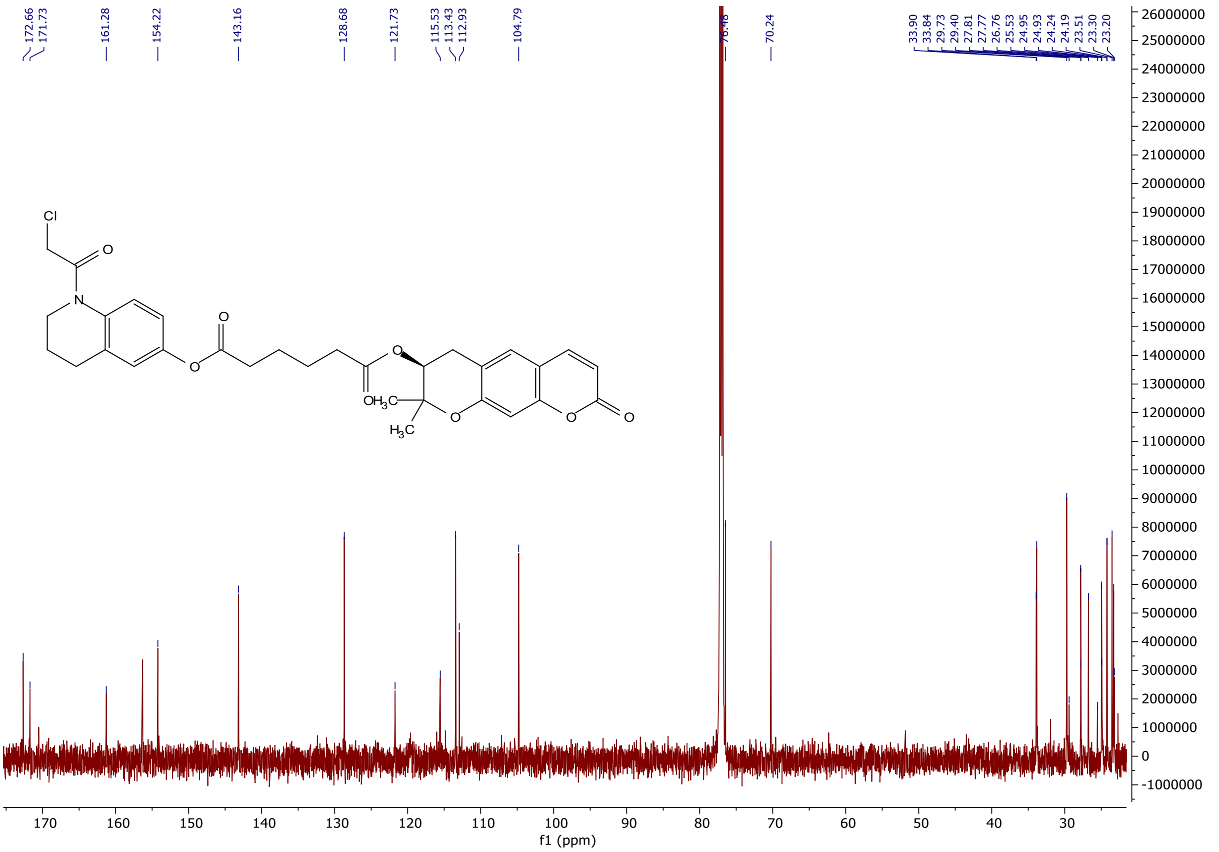
172.66
171.73
161.28
154.22
143.16
128.68
121.73
115.53
113.43
112.93
104.79
76.48
70.24
33.90
33.84
29.73
29.40
27.81
27.77
26.76
25.53
24.95
24.93
24.24
24.19
23.51
23.30
23.20
Cl
O
N
O
O
O
O
O
O
H_3C
H_3C
f1 (ppm)

CRBN-based negative control **13**

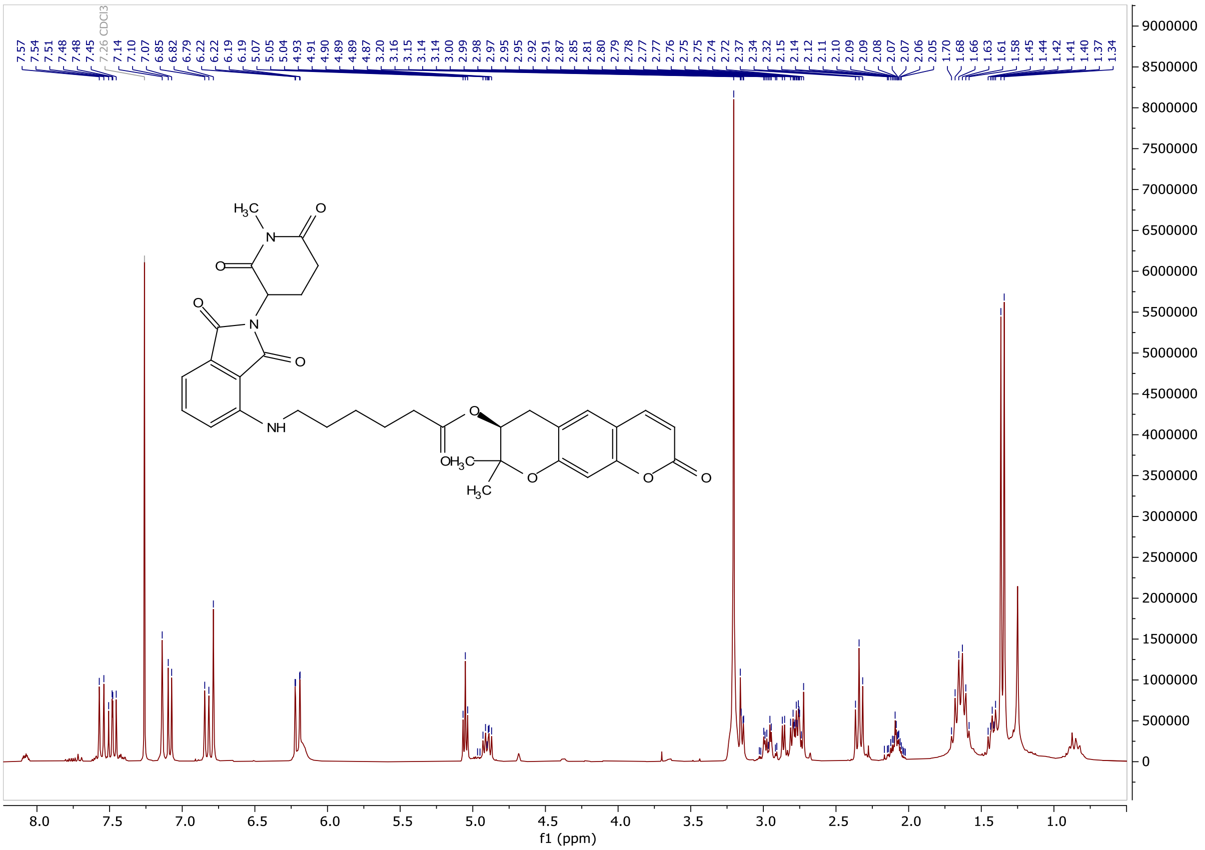

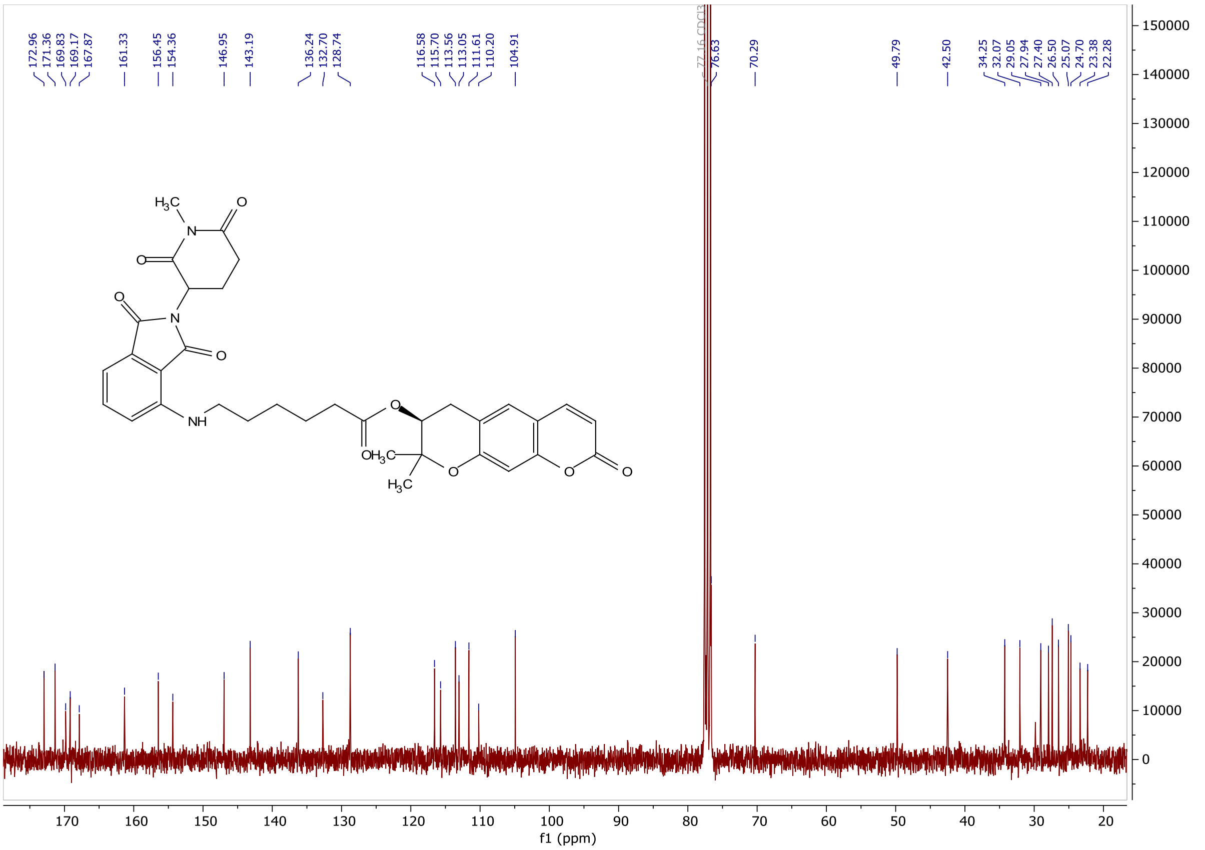

CRBN-based negative control **14**

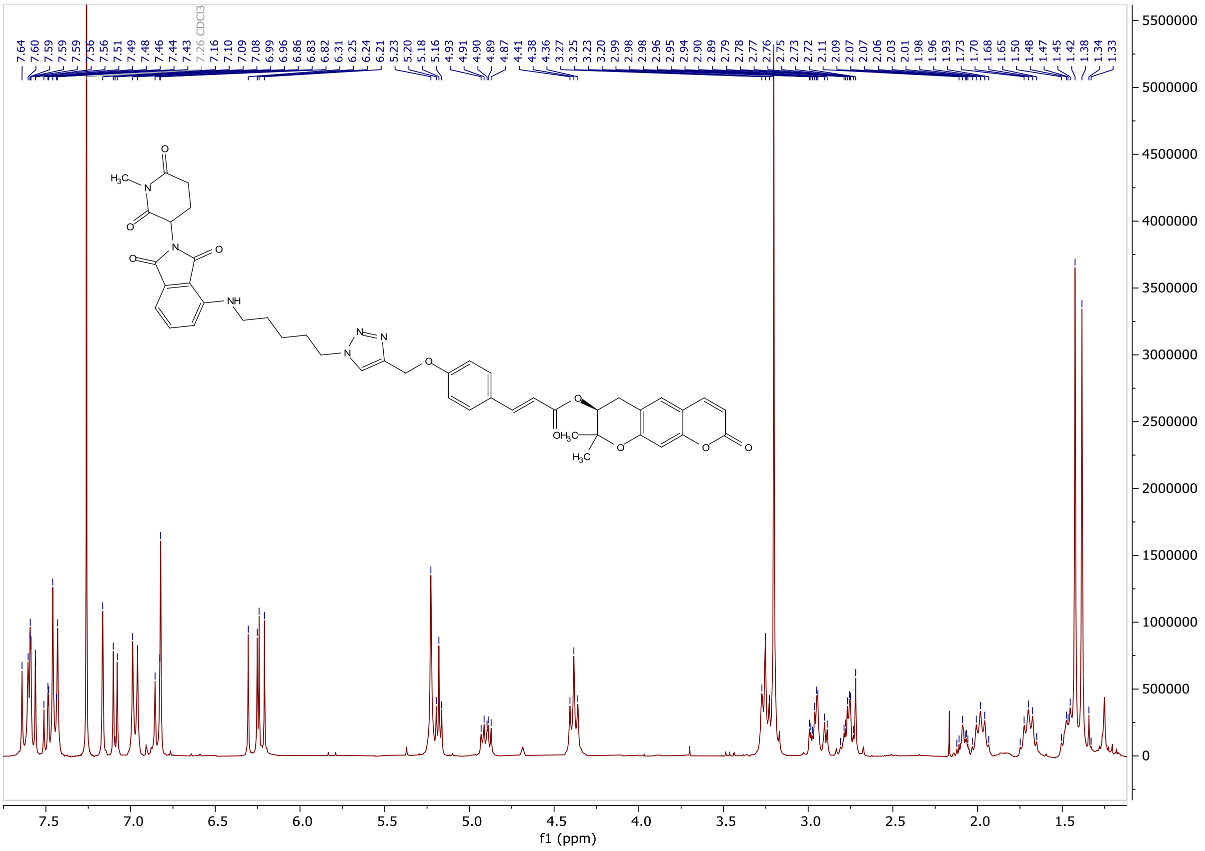

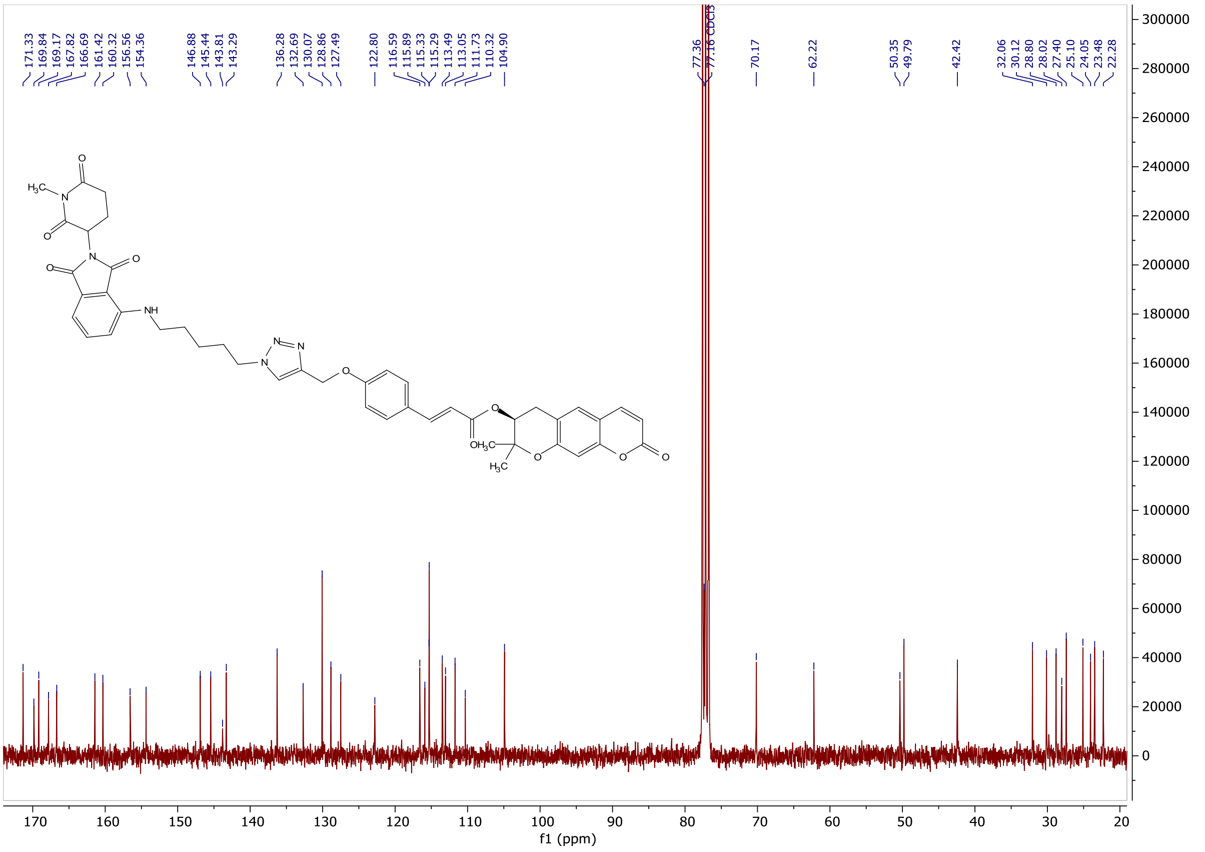

PROTAC **2**

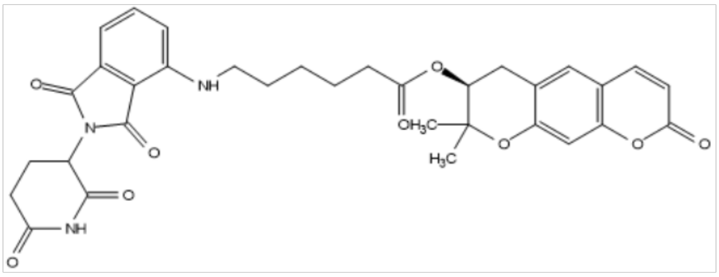

Signal=254.4 nm

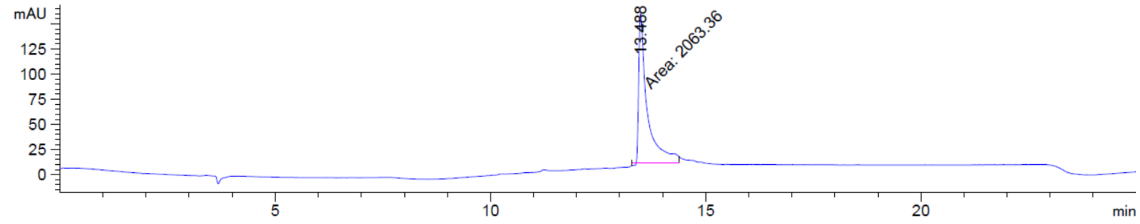

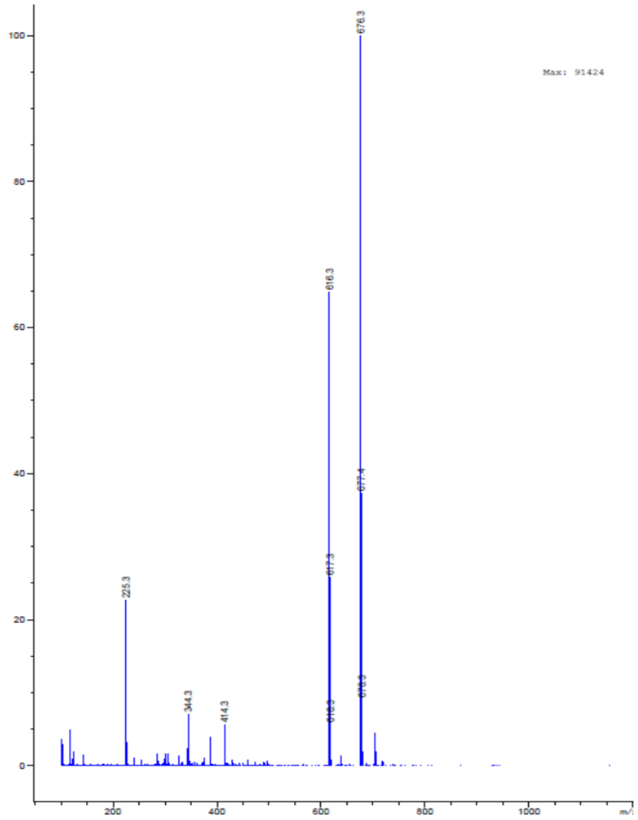

Acquisition Parameter

Source Type	ESI	Ion Polarity	Positive	Set Nebulizer	3.0 Bar
Focus	Active	Set Capillary	4500 V	Set Dry Heater	200 °C
Scan Begin	50 m/z	Set End Plate Offset	-500 V	Set Dry Gas	9.0 l/min
Scan End	1300 m/z	Set Collision Cell RF	1000.0 Vpp	Set Divert Valve	Waste

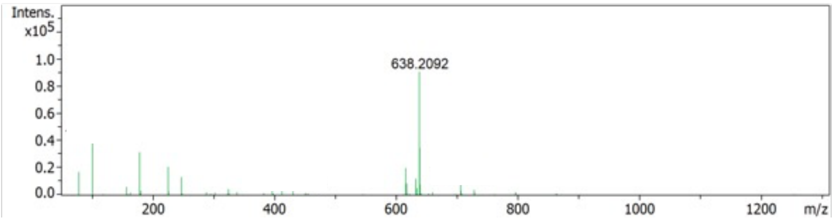

PROTAC **3**

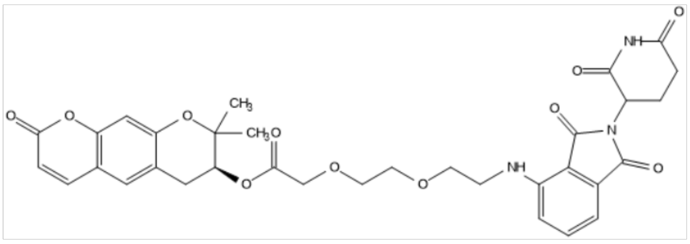

Signal=254.4 nm

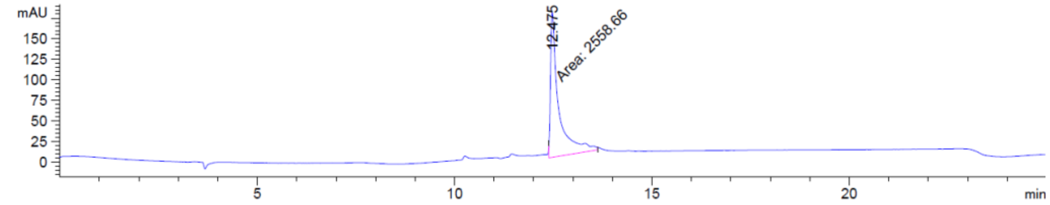

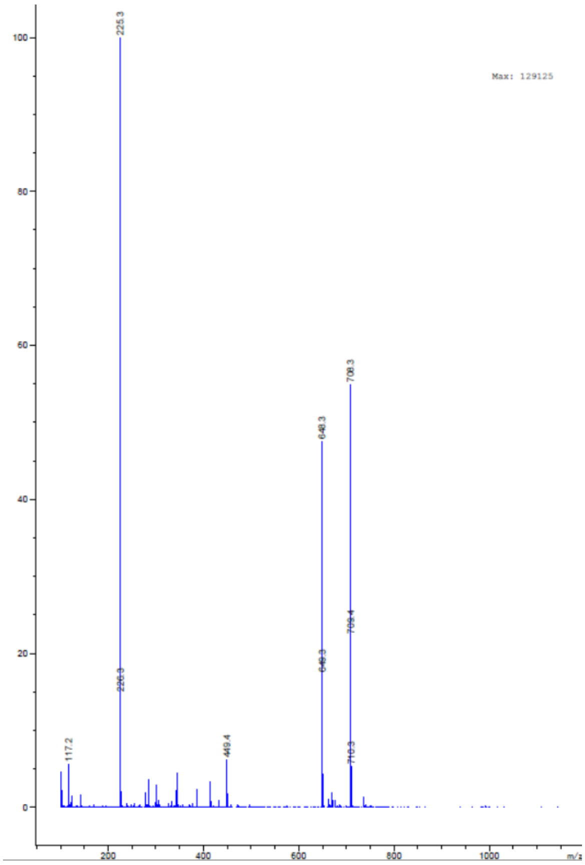

Acquisition Parameter

Source Type	ESI	Ion Polarity	Positive	Set Nebulizer	3.0 Bar
Focus	Active	Set Capillary	4500 V	Set Dry Heater	200 °C
Scan Begin	50 m/z	Set End Plate Offset	-500 V	Set Dry Gas	9.0 l/min
Scan End	1300 m/z	Set Collision Cell RF	1000.0 Vpp	Set Divert Valve	Waste

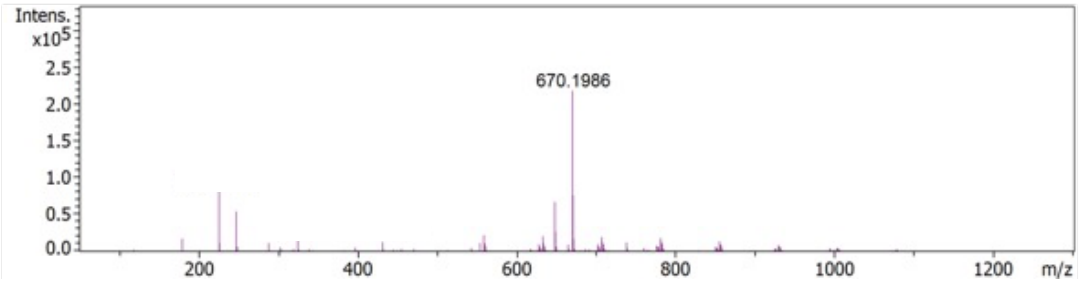

PROTAC **4**

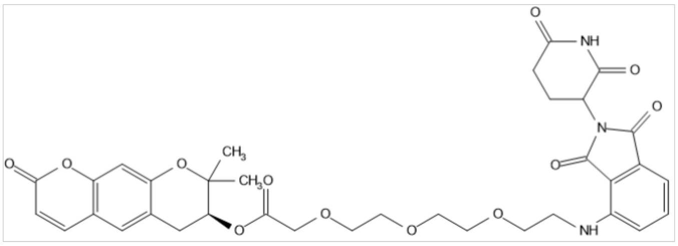

Signal=210.8 nm

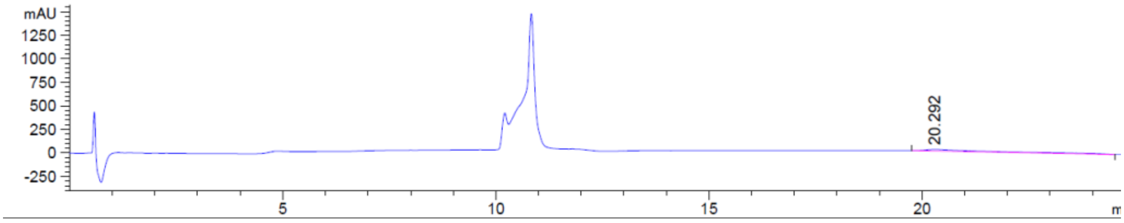

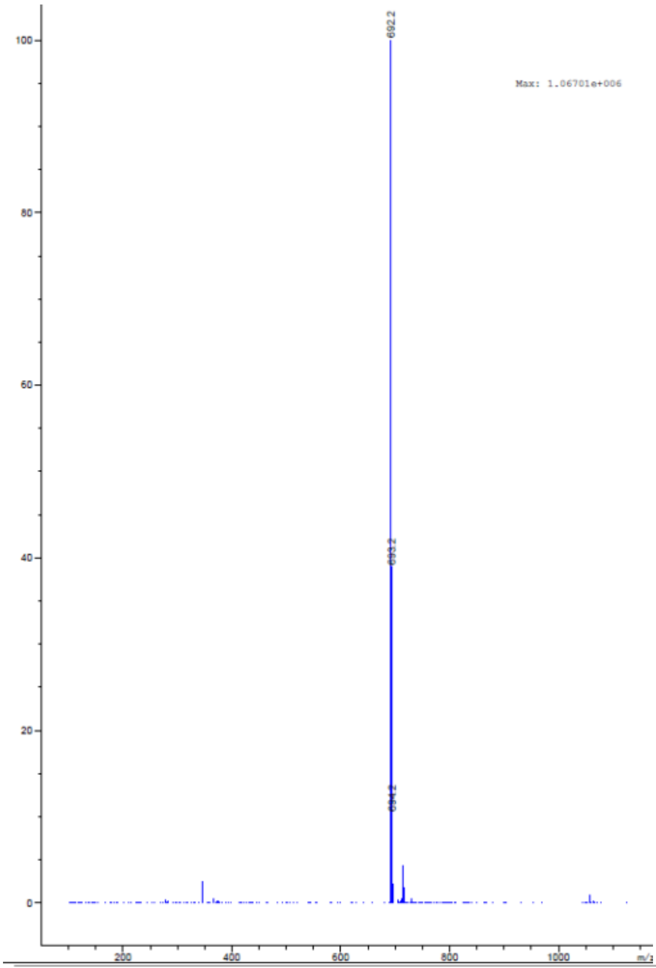

Acquisition Parameter

Source Type	ESI	Ion Polarity	Positive	Set Nebulizer	3.0 Bar
Focus	Active	Set Capillary	4500 V	Set Dry Heater	200 °C
Scan Begin	50 m/z	Set End Plate Offset	-500 V	Set Dry Gas	9.0 l/min
Scan End	1300 m/z	Set Collision Cell RF	1000.0 Vpp	Set Divert Valve	Waste

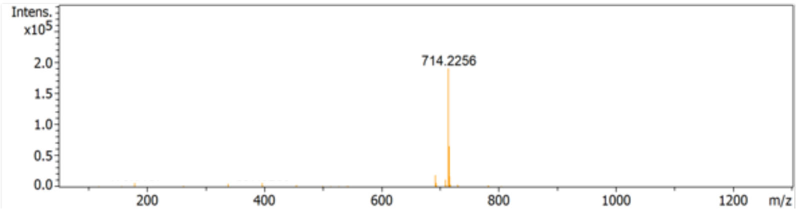

PROTAC **5**

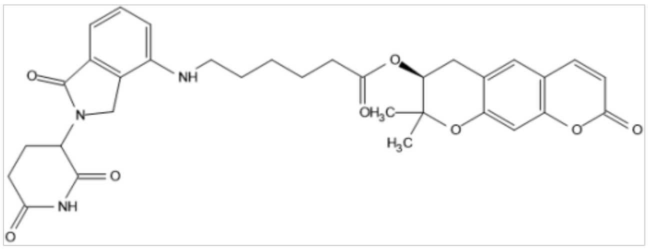

Signal=254.4 nm

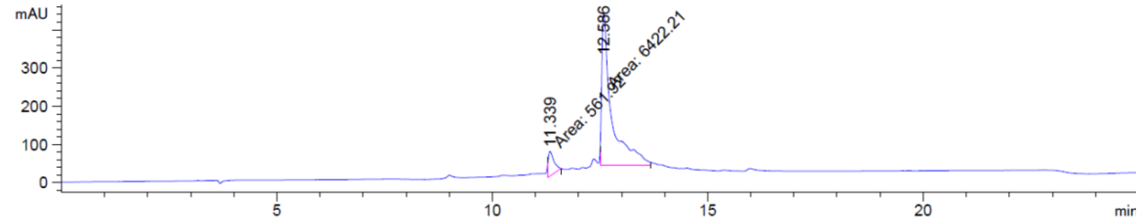

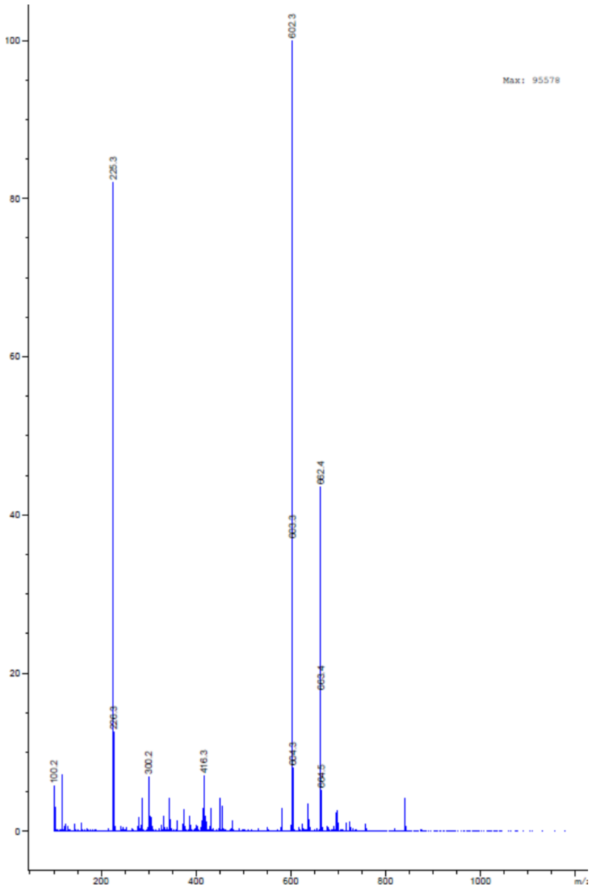

Acquisition Parameter

Source Type	ESI	Ion Polarity	Positive	Set Nebulizer	3.0 Bar
Focus	Active	Set Capillary	4500 V	Set Dry Heater	200 °C
Scan Begin	50 m/z	Set End Plate Offset	-500 V	Set Dry Gas	9.0 l/min
Scan End	1300 m/z	Set Collision Cell RF	1000.0 Vpp	Set Divert Valve	Waste

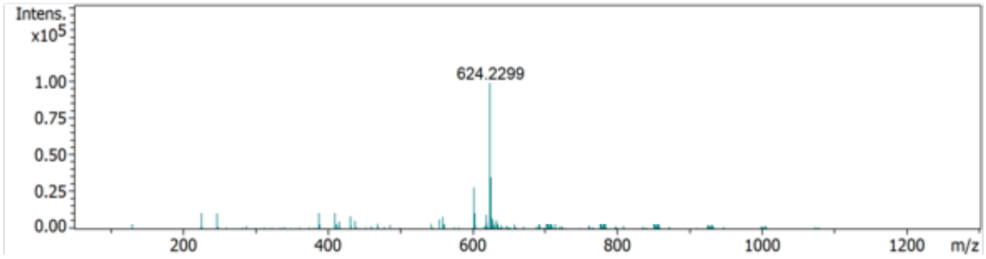

PROTAC **6**

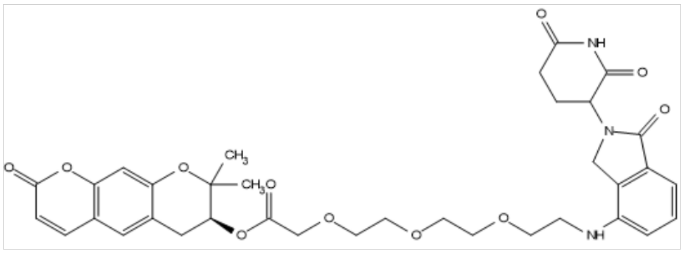

Signal=254.4 nm

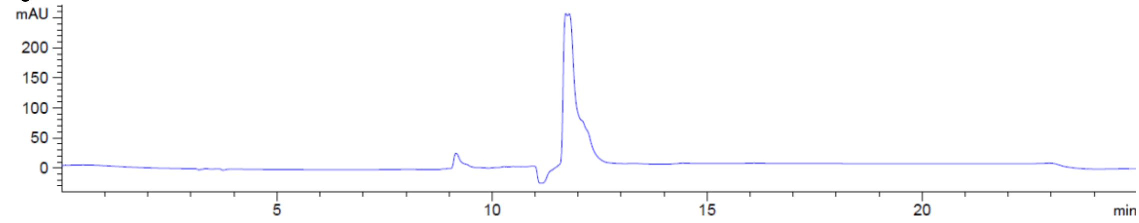

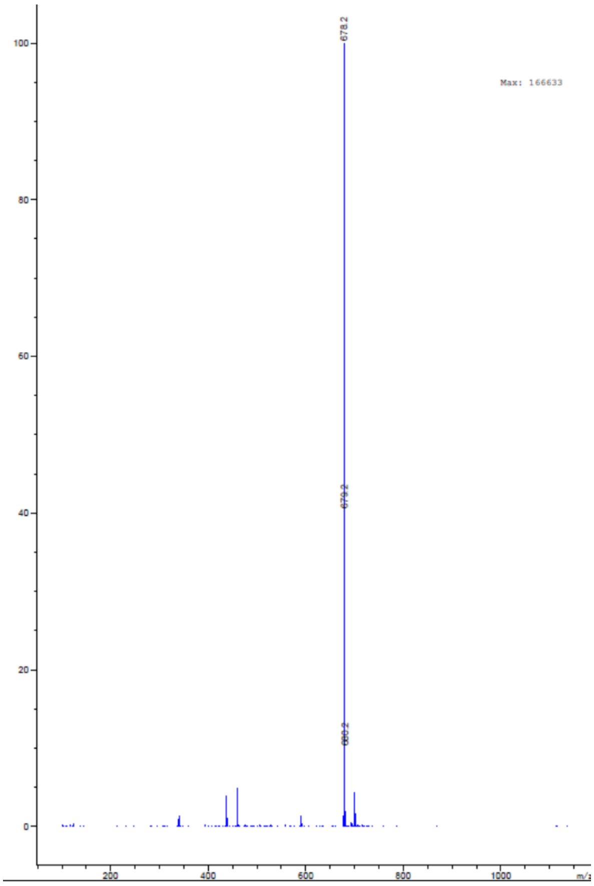

Acquisition Parameter

Source Type	ESI	Ion Polarity	Positive	Set Nebulizer	3.0 Bar
Focus	Active	Set Capillary	4500 V	Set Dry Heater	200 °C
Scan Begin	50 m/z	Set End Plate Offset	-500 V	Set Dry Gas	9.0 l/min
Scan End	1300 m/z	Set Collision Cell RF	1000.0 Vpp	Set Divert Valve	Waste

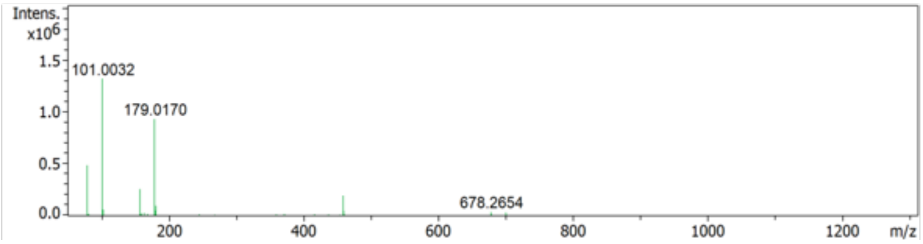

PROTAC **7**

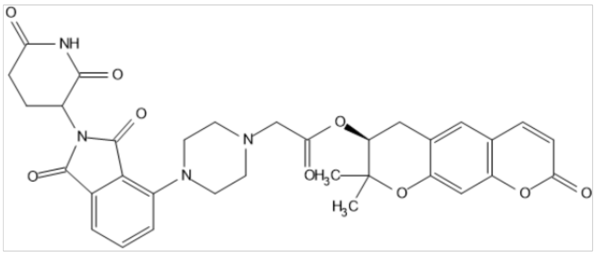

Signal=254.4 nm

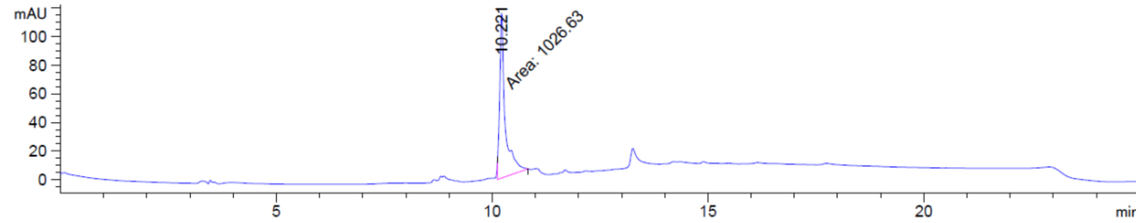

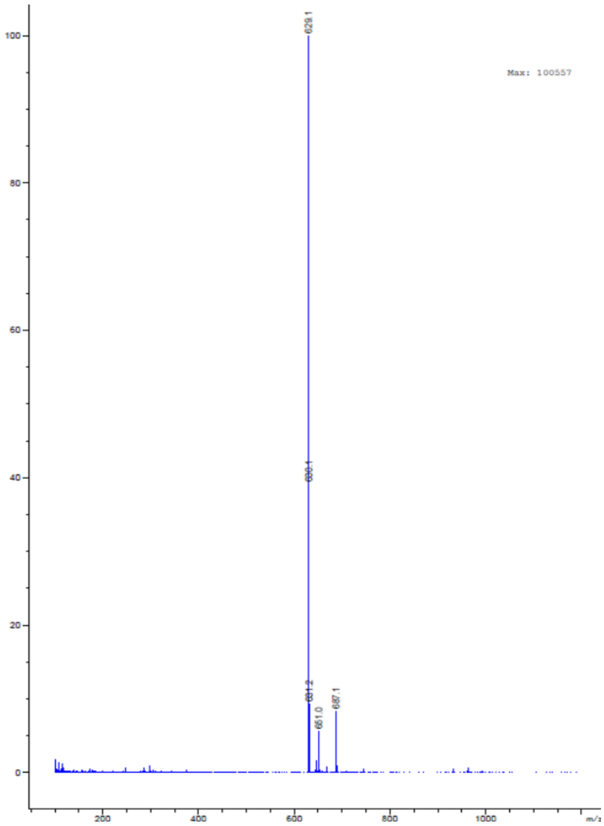

Acquisition Parameter

Source Type	ESI	Ion Polarity	Positive	Set Nebulizer	3.0 Bar
Focus	Active	Set Capillary	4500 V	Set Dry Heater	200 °C
Scan Begin	50 m/z	Set End Plate Offset	-500 V	Set Dry Gas	9.0 l/min
Scan End	1300 m/z	Set Collision Cell RF	1000.0 Vpp	Set Divert Valve	Waste

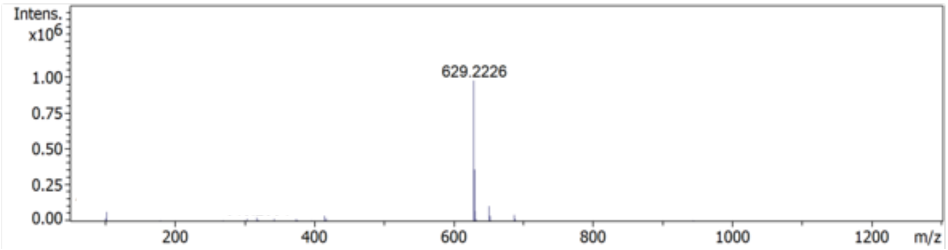

PROTAC **8**

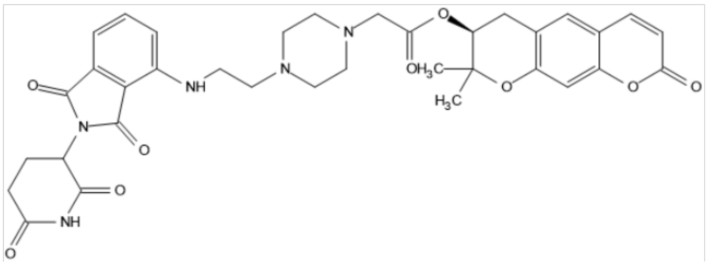

Signal=254.4 nm

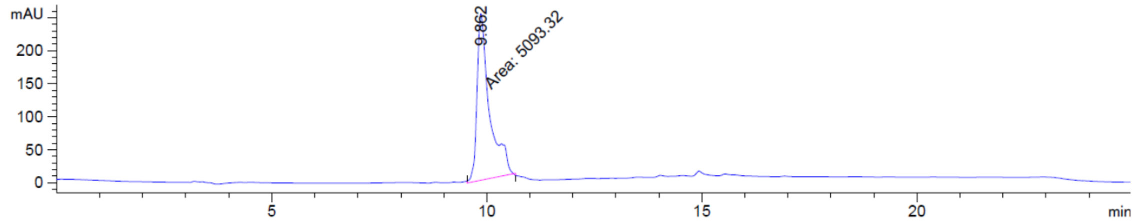

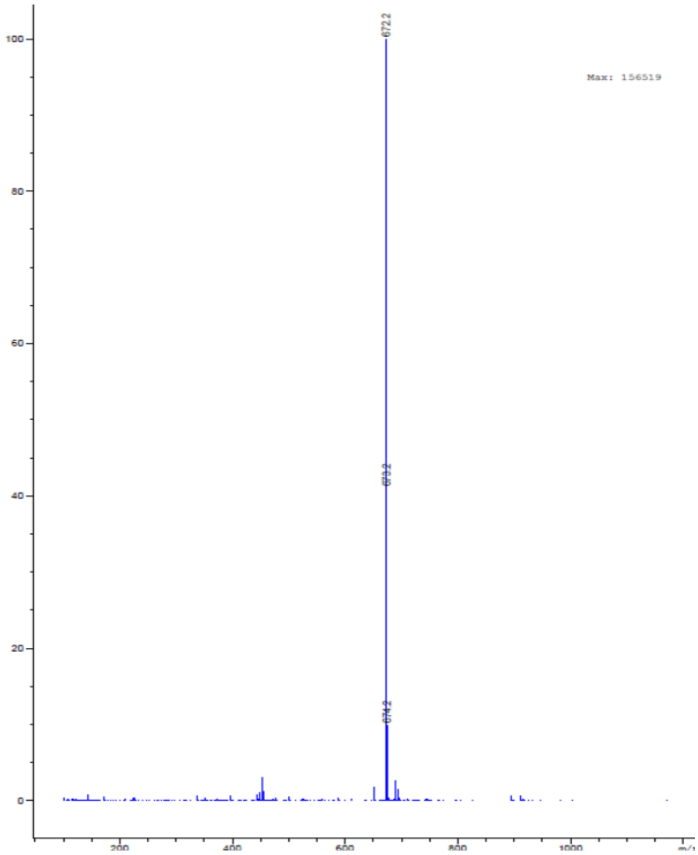

Acquisition Parameter

Source Type	ESI	Ion Polarity	Positive	Set Nebulizer	3.0 Bar
Focus	Active	Set Capillary	4500 V	Set Dry Heater	200 °C
Scan Begin	50 m/z	Set End Plate Offset	-500 V	Set Dry Gas	9.0 l/min
Scan End	1300 m/z	Set Collision Cell RF	1000.0 Vpp	Set Divert Valve	Waste

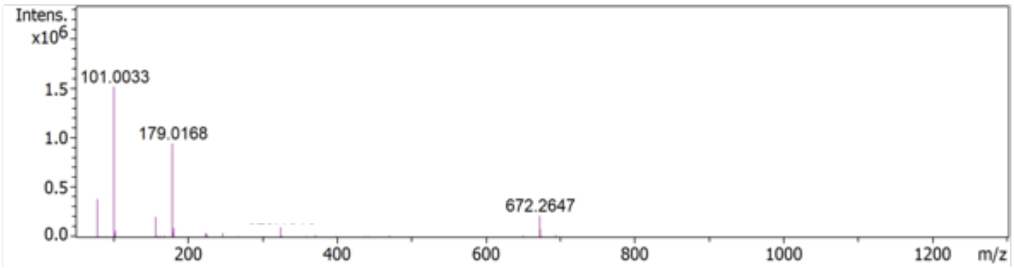

PROTAC **9**

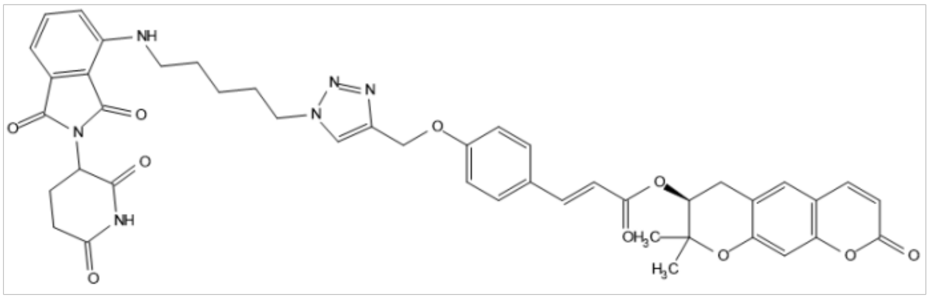

Signal=210.8 nm

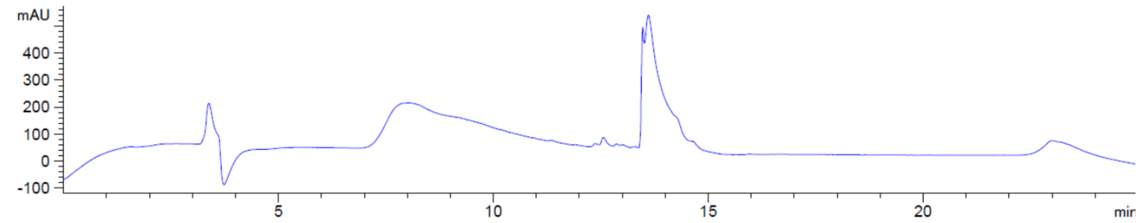

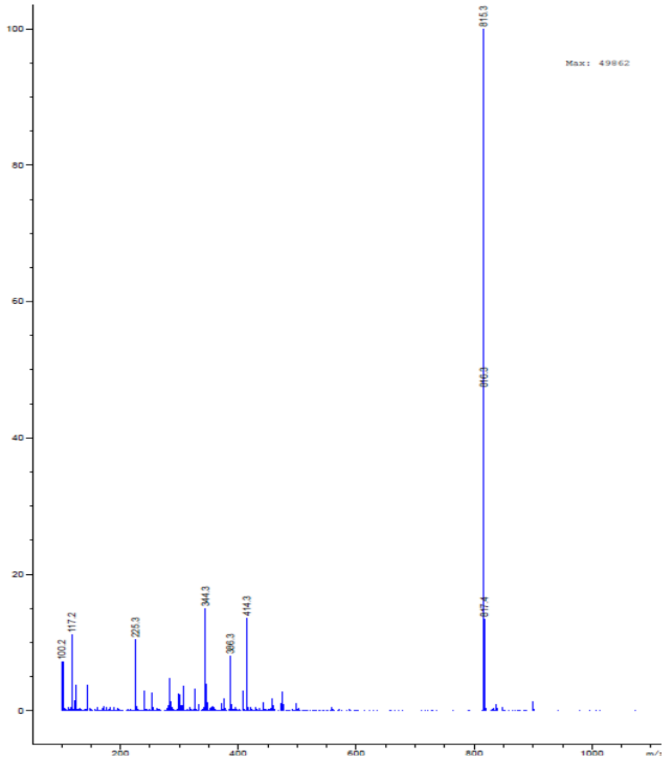

Acquisition Parameter

Source Type	ESI	Ion Polarity	Positive	Set Nebulizer	3.0 Bar
Focus	Active	Set Capillary	4500 V	Set Dry Heater	200 °C
Scan Begin	50 m/z	Set End Plate Offset	-500 V	Set Dry Gas	9.0 l/min
Scan End	1300 m/z	Set Collision Cell RF	1000.0 Vpp	Set Divert Valve	Waste

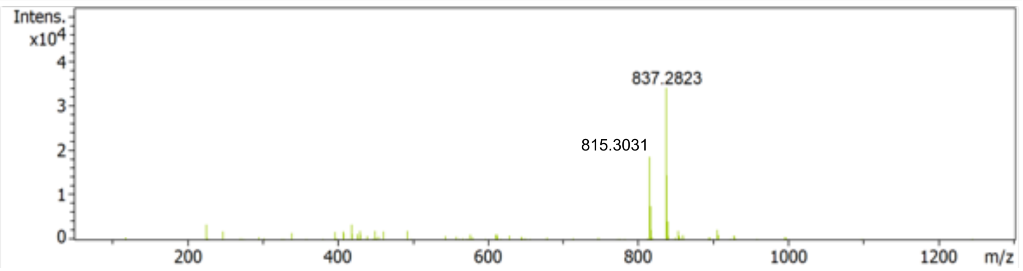

PROTAC **10**

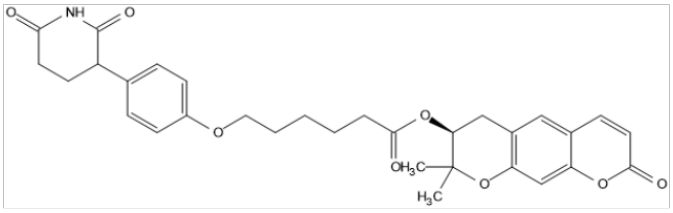

Signal=210.8 nm

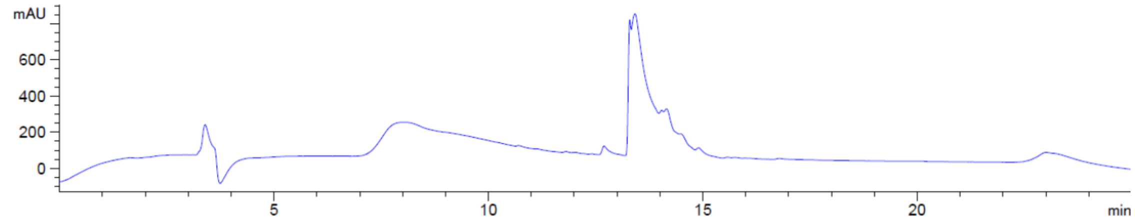

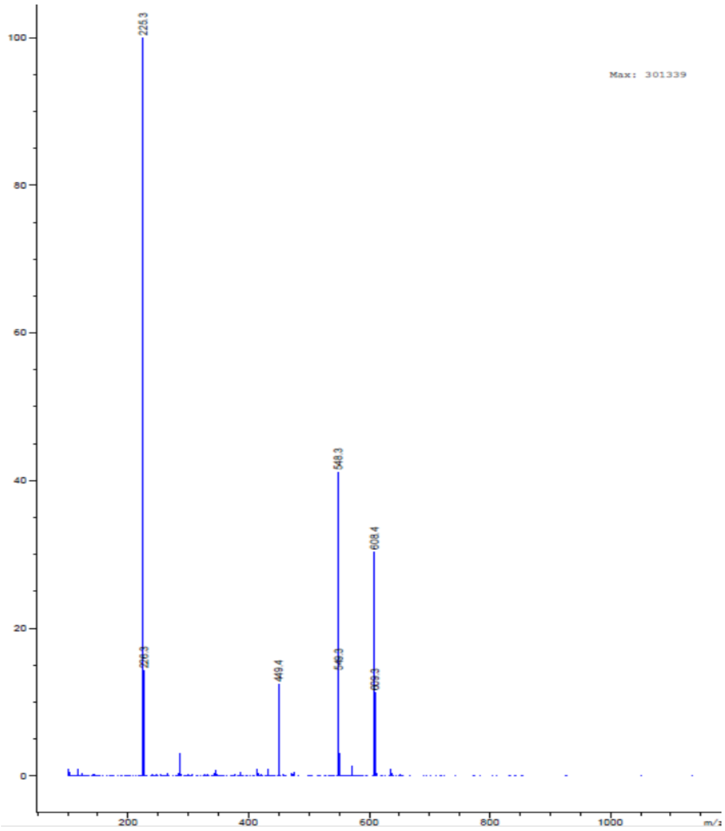

Acquisition Parameter

Source Type	ESI	Ion Polarity	Positive	Set Nebulizer	3.0 Bar
Focus	Active	Set Capillary	4500 V	Set Dry Heater	200 °C
Scan Begin	50 m/z	Set End Plate Offset	-500 V	Set Dry Gas	9.0 l/min
Scan End	1300 m/z	Set Collision Cell RF	1000.0 Vpp	Set Divert Valve	Waste

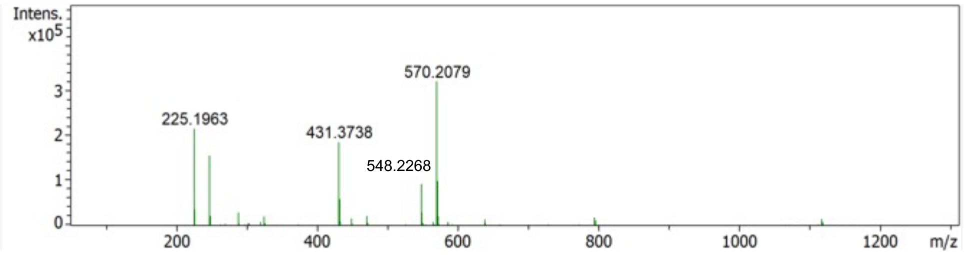

PROTAC **11**

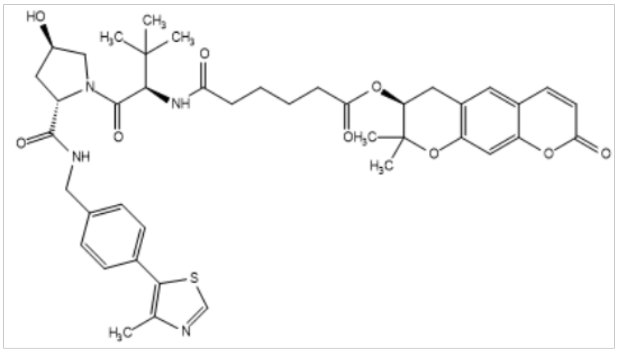

Signal=254.4 nm

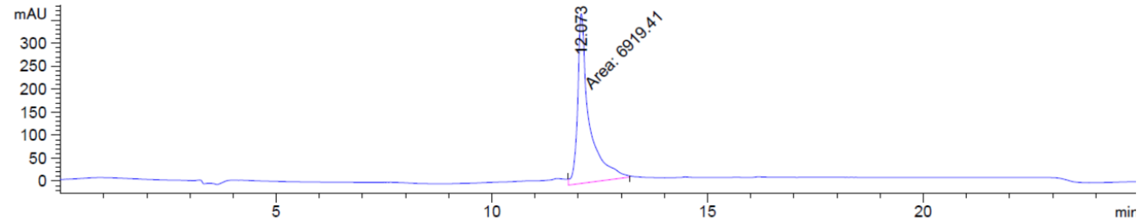

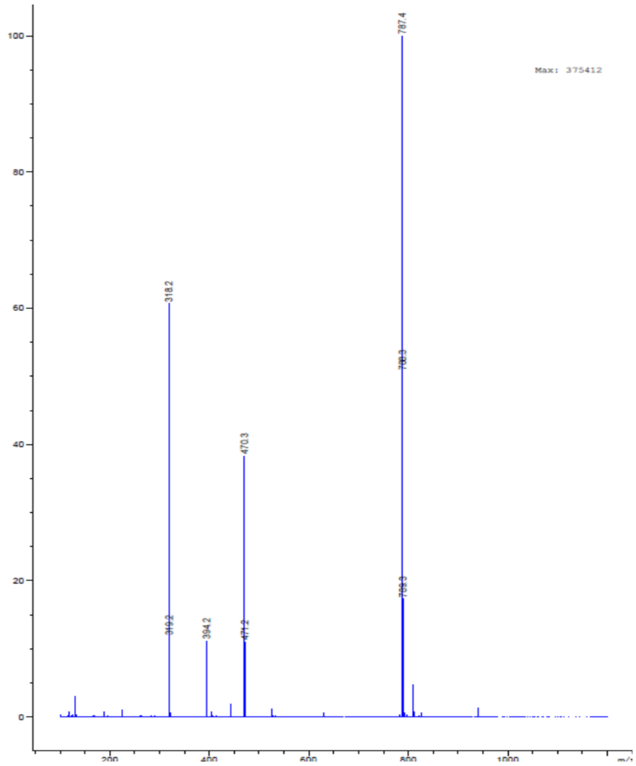

Acquisition Parameter

Source Type	ESI	Ion Polarity	Positive	Set Nebulizer	3.0 Bar
Focus	Active	Set Capillary	4500 V	Set Dry Heater	200 °C
Scan Begin	50 m/z	Set End Plate Offset	-500 V	Set Dry Gas	9.0 l/min
Scan End	1300 m/z	Set Collision Cell RF	1000.0 Vpp	Set Divert Valve	Waste

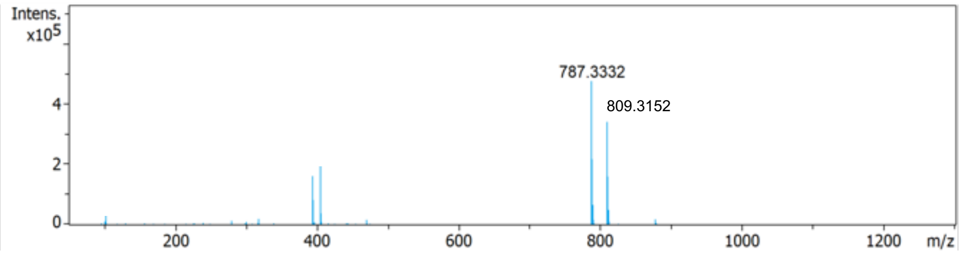

PROTAC **12**

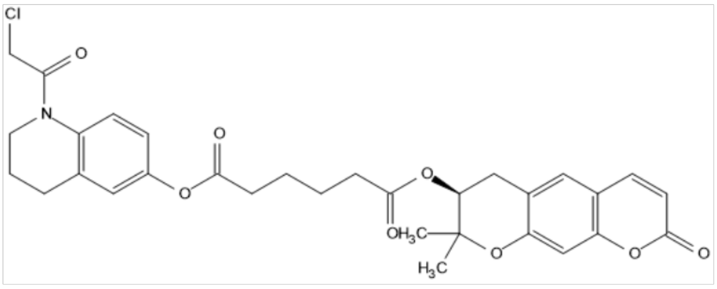

Signal=254.4 nm

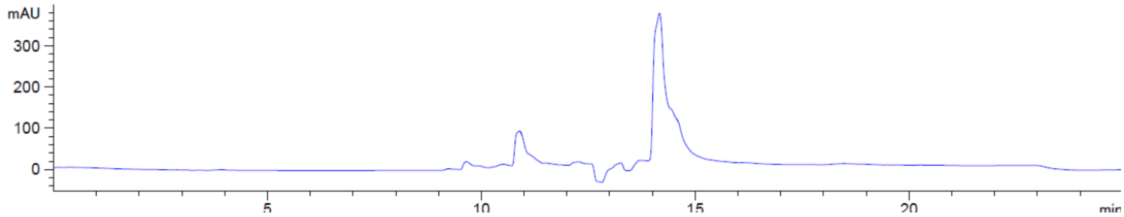

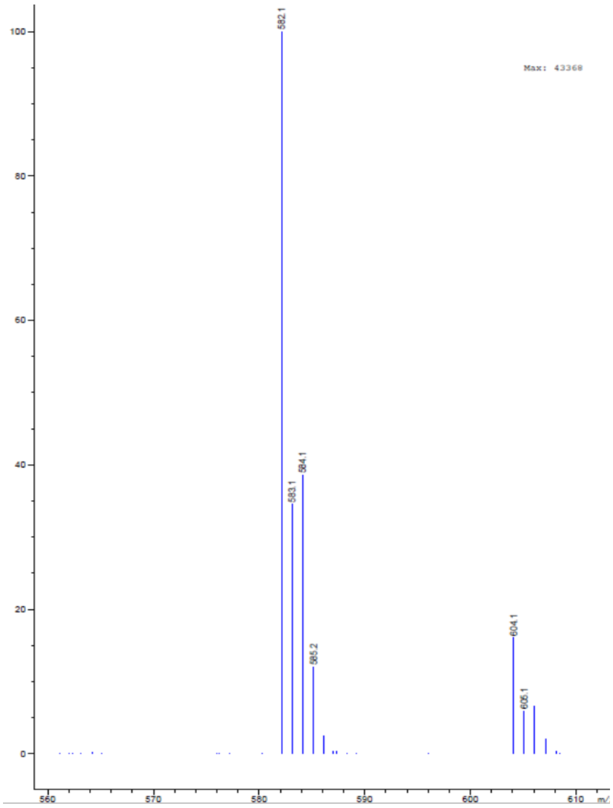

Acquisition Parameter

Source Type	ESI	Ion Polarity	Positive	Set Nebulizer	3.0 Bar
Focus	Active	Set Capillary	4500 V	Set Dry Heater	200 °C
Scan Begin	50 m/z	Set End Plate Offset	-500 V	Set Dry Gas	9.0 l/min
Scan End	1300 m/z	Set Collision Cell RF	1000.0 Vpp	Set Divert Valve	Waste

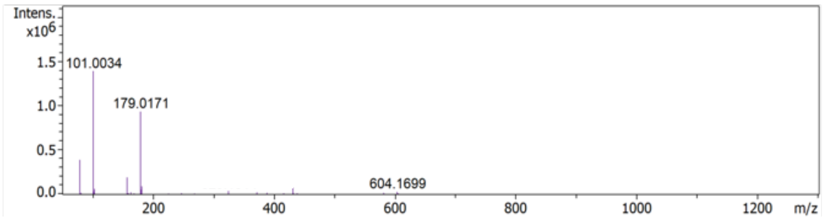

CRBN-based negative control **13**

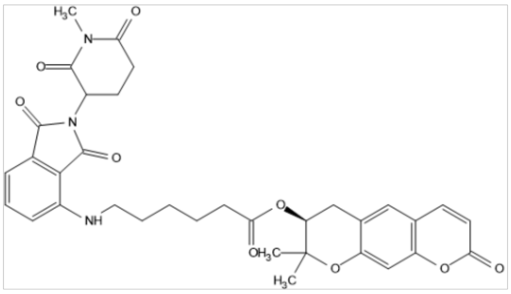

Signal=254.4 nm

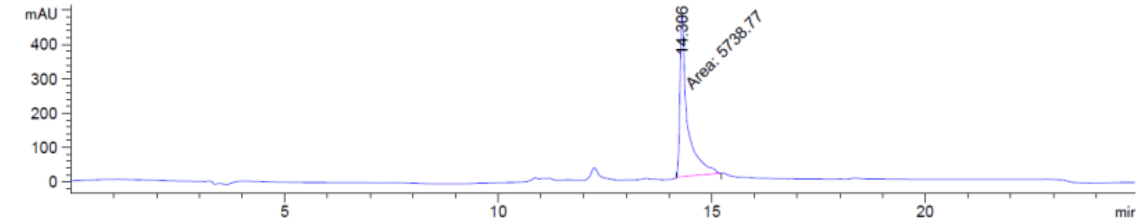

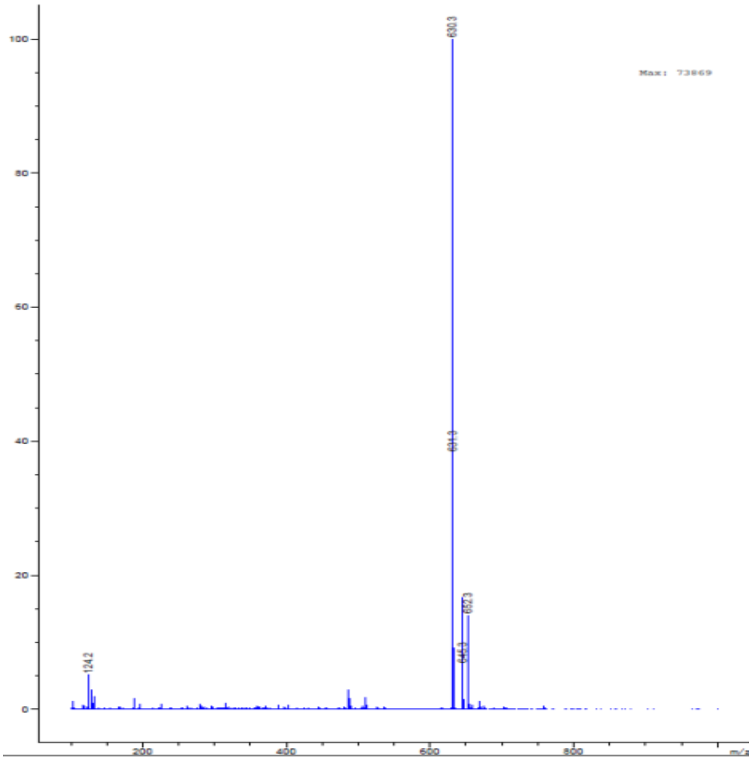

Acquisition Parameter

Source Type	ESI	Ion Polarity	Positive	Set Nebulizer	3.0 Bar
Focus	Active	Set Capillary	4500 V	Set Dry Heater	200 °C
Scan Begin	50 m/z	Set End Plate Offset	-500 V	Set Dry Gas	9.0 l/min
Scan End	1300 m/z	Set Collision Cell RF	1000.0 Vpp	Set Divert Valve	Waste

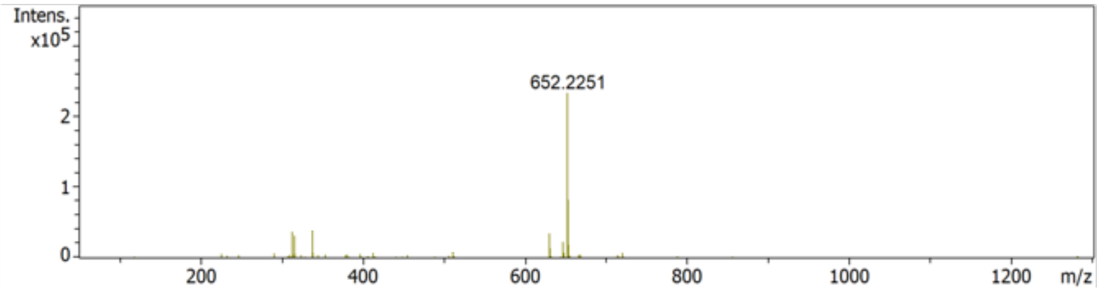

CRBN-based negative control **14**

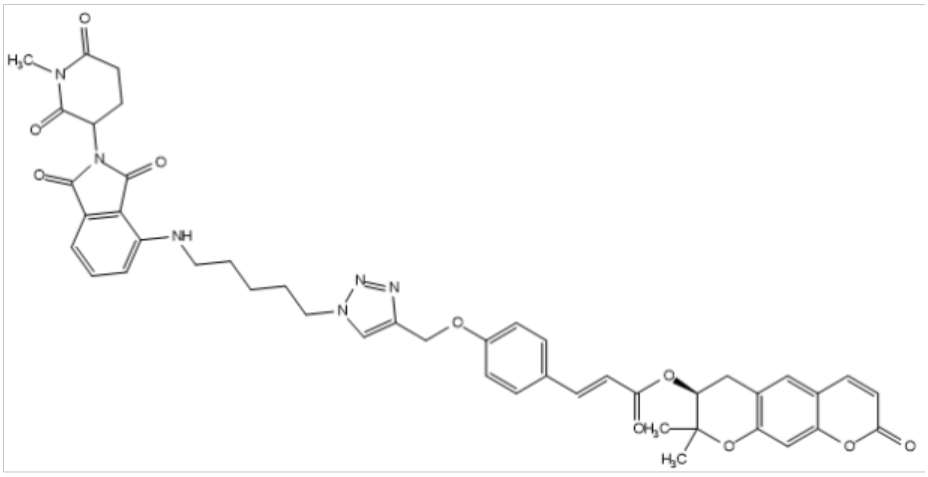

Signal=210.8 nm

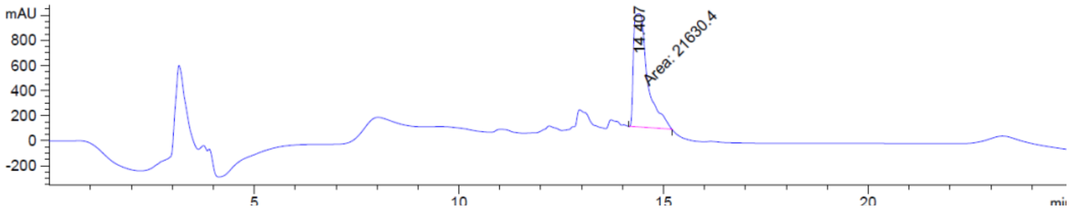

Acquisition Parameter

Source Type	ESI	Ion Polarity	Positive	Set Nebulizer	3.0 Bar
Focus	Active	Set Capillary	4500 V	Set Dry Heater	200 °C
Scan Begin	50 m/z	Set End Plate Offset	-500 V	Set Dry Gas	9.0 l/min
Scan End	1300 m/z	Set Collision Cell RF	1000.0 Vpp	Set Divert Valve	Waste

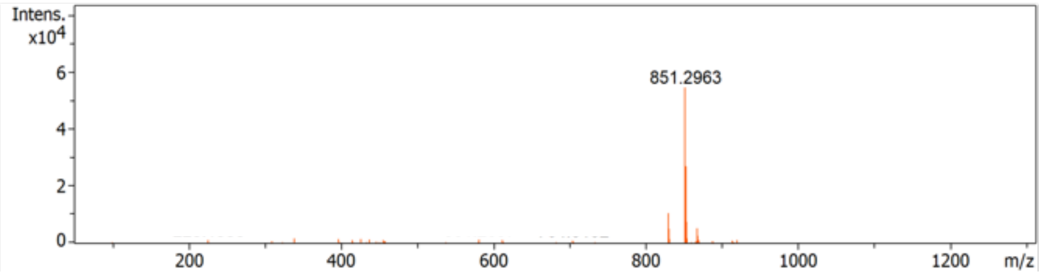

Supplement: Supplementary file 1 — Supporting file: advs74965‐sup‐0001‐SuppMat.pdf [file ADVS-13-e21608-s001.pdf]
